# Mass deworming for improving health and cognition of children in endemic helminth areas: A systematic review and individual participant data network meta‐analysis

**DOI:** 10.1002/cl2.1058

**Published:** 2019-11-20

**Authors:** Vivian A. Welch, Elizabeth Ghogomu, Alomgir Hossain, Alison Riddle, Michelle Gaffey, Paul Arora, Omar Dewidar, Rehana Salam, Simon Cousens, Robert Black, T. Déirdre Hollingsworth, Sue Horton, Peter Tugwell, Donald Bundy, Mary Christine Castro, Alison Elliott, Henrik Friis, Huong T. Le, Chengfang Liu, Emily K. Rousham, Fabian Rohner, Charles King, Erliyani Sartono, Taniawati Supali, Peter Steinmann, Emily Webb, Franck Wieringa, Pattanee Winnichagoon, Maria Yazdanbakhsh, Zulfiqar A. Bhutta, George Wells

**Affiliations:** ^1^ Centre for Global Health Bruyère Research Institute Ottawa Ontario Canada; ^2^ School of Epidemiology Public Health and Preventive Medicine, University of Ottawa Ottawa Ontario Canada; ^3^ Bruyère Research Institute University of Ottawa Ottawa Ontario Canada; ^4^ Cardiovascular Research Methods University of Ottawa Heart Institute Ottawa Ontario Canada; ^5^ Hospital for Sick Children University of Toronto Toronto Ontario Canada; ^6^ Public Health Agency of Canada in the National Public Health Laboratory and Dalla Lana School of Public Health University of Toronto Toronto Ontario Canada; ^7^ South Australian Health and Medical Research Institute University of Adelaide Adelaide Australia; ^8^ London School of Hygiene and Tropical Medicine (LSHTM) London UK; ^9^ Department of International Health Johns Hopkins School of Hygiene and Public Health Baltimore Maryland; ^10^ Big Data Institute, Li Ka Shing Centre for Health Information and Discovery, Nuffield Department of Medicine University of Oxford Oxford UK; ^11^ School of Public Health and Health Systems University of Waterloo Waterloo Ontario Canada; ^12^ Center for Global Health, WHO Collaborating Centre for Knowledge Translation and Health Technology Assessment in Health Equity Bruyère Research Institute Ottawa Ontario Canada; ^13^ Bill & Melinda Gates Foundation London UK; ^14^ Nutrition Center of the Philippines Manila Philippines; ^15^ Medical Research Council/Uganda Virus Research Institute London School of Hygiene and Tropical Medicine Uganda Research Unit Entebbe Uganda; ^16^ Department of Human Nutrition University of Copenhagen Frederiksberg Denmark; ^17^ Institute for Preventive Medicine and Public Health Hanoi Medical University Hanoi Vietnam; ^18^ School of Advanced Agricultural Sciences (SAAS) China Center for Agricultural Policy (CCAP), Peking University Beijing China; ^19^ School of Sport, Exercise and Health Sciences Loughborough University Leicestershire UK; ^20^ GroundWork Fläsch Switzerland; ^21^ Department of Pediatrics University of California La Jolla California; ^22^ Department of Parasitology Leiden University Medical Center Leiden The Netherlands; ^23^ Department Parasitology, Faculty of Medicine Universitas Indonesia Jakarta Indonesia; ^24^ Swiss Tropical and Public Health Institute University of Basel Basel Switzerland; ^25^ Department of Infectious Disease Epidemiology, Faculty of Epidemiology and Population Health London School of Hygiene and Tropical Medicine London UK; ^26^ UMR204 Nutripass Institute de Recherche pour le Développement Montpellier France; ^27^ Community/International Nutrition, Institute of Nutrition Mahidol University Nakhon Pathom Thailand; ^28^ Centre for Global Child Health, The Hospital for Sick Children, Center of Excellence in Women and Child Health Aga Khan University Karachi Pakistan

## Abstract

**Background:**

Soil transmitted (or intestinal) helminths and schistosomes affect millions of children worldwide.

**Objectives:**

To use individual participant data network meta‐analysis (NMA) to explore the effects of different types and frequency of deworming drugs on anaemia, cognition and growth across potential effect modifiers.

**Search Methods:**

We developed a search strategy with an information scientist to search MEDLINE, CINAHL, LILACS, Embase, the Cochrane Library, Econlit, Internet Documents in Economics Access Service (IDEAS), Public Affairs Information Service (PAIS), Social Services Abstracts, Global Health CABI and CAB Abstracts up to March 27, 2018. We also searched grey literature, websites, contacted authors and screened references of relevant systematic reviews.

**Selection Criteria:**

We included randomised and quasirandomised deworming trials in children for deworming compared to placebo or other interventions with data on baseline infection.

**Data Collection and Analysis:**

We conducted NMA with individual participant data (IPD), using a frequentist approach for random‐effects NMA. The covariates were: age, sex, weight, height, haemoglobin and infection intensity. The effect estimate chosen was the mean difference for the continuous outcome of interest.

**Results:**

We received data from 19 randomized controlled trials with 31,945 participants. Overall risk of bias was low. There were no statistically significant subgroup effects across any of the potential effect modifiers. However, analyses showed that there may be greater effects on weight for moderate to heavily infected children (very low certainty evidence).

**Authors' Conclusions:**

This analysis reinforces the case against mass deworming at a population‐level, finding little effect on nutritional status or cognition. However, children with heavier intensity infections may benefit more. We urge the global community to adopt calls to make data available in open repositories to facilitate IPD analyses such as this, which aim to assess effects for the most vulnerable individuals.

## 1 PLAIN LANGUAGE SUMMARY

Mass deworming programmes have little effect on nutritional status and cognitive development on a population level

### 1.1 The Campbell review in brief

The effectiveness and cost‐effectiveness of mass deworming of children to improve child health and other outcomes is debated. This independent analysis reinforces the case against mass deworming at a population‐level, finding little effect on nutritional status or cognition. However, children with heavier intensity infections may benefit more.

### 1.2 What is this review about?

Soil‐transmitted helminthiasis (STH) and schistosomiasis affects over 800 million people. There is ongoing debate about whether mass deworming of children improves child nutritional status and cognitive development in endemic areas.

#### 1.2.1 What studies are included?

Randomised trials of mass deworming for STH (alone or in combination with other drugs or child health interventions) for children aged 6 months to 16 years were eligible if they reported at least one of the following outcomes: growth, haemoglobin, serum ferritin, or cognitive processing or development. Trials had to collect data on baseline STH infection intensity, since the main purpose of this review was to assess effect modification across intensity of infection.

Individual participant data (IPD) was obtained from 19 out of 41 eligible randomised trials. These 19 trials included 31,945 participants and had an overall low risk of bias.

A secondary analysis added new data to the meta‐analysis of STH deworming versus placebo of a previous Campbell review by the same authors. This analysis included 29 randomised trials, with data from two studies which had not published weight gain data and updated effect estimates from three studies based on the data provided by authors.

These studies were conducted in 11 low and middle income countries. Most programmes conducted deworming every 4 months or more frequently. Seven out of 19 studies gave a single dose of deworming. Children were school‐age, with a median of 11 years of age.

### 1.3 Does deworming improve child health and other welfare outcomes?

Mass deworming for STHs compared to placebo probably has little to no effect on nutritional status or cognitive development (moderate certainty evidence). Children with moderate to heavy intensity infections of *Ascaris lumbricoides* or *Trichuris trichiuria* may experience greater weight gain (very low certainty evidence). No other differences in effects were found across age, sex or baseline nutritional status.

Findings are consistent for studies at low risk of bias and for other methodological considerations such as completer analyses. There was no trend in effect according to publication year, baseline *A. lumbricoides* prevalence or *T. trichuria* prevalence in the full dataset of 29 studies. Higher baseline hookworm prevalence was weakly associated with greater effects of STH deworming.

### 1.4 What are the implications of this review for policy makers and decision makers?

This analysis replicates the prior findings of small effects of mass deworming at the population level. In areas where there are children with moderate to heavy intensity infections, which are increasingly uncommon, mass deworming may be beneficial, but this analysis was limited by the small number of children with heavy intensity infections in this sample (<1,000). In areas with light intensity infections, mass deworming programmes probably have very small effects on weight for these children and additional policy options need to be explored to improve child health and nutrition in these areas.

### 1.5 What are the research implications of this review?

This analysis was severely limited by not being able to obtain IPD for many older studies, which may have included children with heavier intensity infections. Greater adoption of calls for open, structured data from trials could maximise the benefit of research to understand effects in the most vulnerable and marginalised populations within these trials.

**Summary of findings table 1: Deworming with any STH drug compared to placebo for children in STH endemic areas**

**Table:** 

Deworming with any STH drug compared to placebo for children in STH endemic areas					
**Patient or population:** Children					
**Settings:** STH endemic areas					
**Intervention:** Deworming with any STH drug					
**Comparison:** Placebo					
**Time:** 4 months or longer					

		Direct evidence		Network meta‐analysis	
Outcomes	No. of participants (studies)	MD (95% CI)	Quality of the evidence (GRADE)	MD (95% CI)	Quality of the evidence (GRADE)
Weight (change in kg)	11,024 (9 studies)	0.05 (−0.02, 0.11)	⊕⊕⊕⊝ moderate^a^	0.01 (−0.08,0.11)	⊕⊕⊕⊝ moderate^a^
Height (change in cm)	11,024 (9 studies)	0.04 (−0.04, 0.11)	⊕⊕⊕⊝ moderate^a^	0.09 (−0.08,0.27)	⊕⊕⊕⊝ moderate^a^
Hemoglobin (change in g/L)	11,024 (9 studies)	0.23 (−0.52, 0.97)	⊕⊕⊕⊝ moderate^a^	0.32 (−0.63,1.26)	⊕⊕⊝⊝ low^a^, ^b^
Cognition	6 studies (5,814 participants)	There was little to no effect on cognitive outcomes measured on various scales for short‐term attention, school achievement, developmental scales			
GRADE Working Group grades of evidence					
**High quality:** Further research is very unlikely to change our confidence in the estimate of effect.					
**Moderate quality:** Further research is likely to have an important impact on our confidence in the estimate of effect and may change the estimate.					
**Low quality:** Further research is very likely to have an important impact on our confidence in the estimate of effect and is likely to change the estimate.					
**Very low quality:** We are very uncertain about the estimate.					

*Note*: Findings based on the analysis of main effects of 19 studies providing individual participant data.

Abbreviations: CI, confidence interval; IPD, individual participant data; STH, soil transmitted helminth.

^a^ Downgraded for study limitations—obtained only a selected sample of IPD from 19 out of 41 eligible studies.

^b^ Downgraded for imprecision.

**Summary of findings table 2: Deworming with praziquantel alone or in combination with any STH deworming compared to placebo for children in STH endemic areas**

**Table:** 

Deworming with Praziquantel alone or in combination with any STH deworming compared to placebo for children in STH endemic areas					
**Patient or population:** Children					
**Settings:** STH endemic areas					
**Intervention:** Deworming with any STH drug and Praziquantel					
**Comparison:** Placebo					
**Time:** 4 months or longer					

		Direct evidence		Network meta‐analysis	
Outcomes	No. of participants (studies)	MD (95% CI)	Quality of the evidence (GRADE)	MD (95% CI)	Quality of the evidence (GRADE)
Weight (change in kg)	2,171 (5 studies)	0.04 (−0.12, 0.20)	⊕⊕⊕⊝ moderate^a^	0.04 (−0.11, 0.19)	⊕⊕⊕⊝ moderate^a^
Height (change in cm)	2,171 (5 studies)	−0.10 (−0.44, 0.25)	⊕⊕⊕⊝ moderate^a^	−0.06 (−0.31, 0.18)	⊕⊕⊕⊝ moderate^a^
Hemoglobin (change in g/L)	2,171 (5 studies)	2.02 (0.93, 3.11)	⊕⊕⊕⊝ moderate^a^	1.85 (0.53, 3.18)	⊕⊕⊝⊝ low^a^, ^b^
GRADE Working Group grades of evidence					
**High quality:** Further research is very unlikely to change our confidence in the estimate of effect.					
**Moderate quality:** Further research is likely to have an important impact on our confidence in the estimate of effect and may change the estimate.					
**Low quality:** Further research is very likely to have an important impact on our confidence in the estimate of effect and is likely to change the estimate.					
**Very low quality:** We are very uncertain about the estimate.					

*Note*: Findings based on the analysis of main effects of 19 studies providing individual participant data.

Abbreviations: CI, confidence interval; STH, soil transmitted helminth.

^a^ Downgraded for study limitations—obtained only a selected sample of IPD from 19 out of 41 eligible studies.

^b^ Downgraded for imprecision.

**Summary of findings table 3: Deworming with any STH drug with iron or micronutrients compared to placebo for children in STH endemic areas**

**Table:** 

Mass deworming with any STH drug with iron or micronutrients compared to placebo for children in STH endemic areas					
**Patient or population:** Children					
**Settings:** STH endemic areas					
**Intervention:** Mass deworming with any STH drug combined with iron or micronutritents					
**Comparison:** Placebo					
**Time:** 4 months or longer					

		Direct evidence		Network meta‐analysis	
Outcomes	No. of participants (studies)	MD (95% CI)	Quality of the evidence (GRADE)	MD (95% CI)	Quality of the evidence (GRADE)
Weight (change in kg)	3,851 (5 studies)	0.00 (−0.07, 0.08)	⊕⊕⊕⊝ moderate^a^	−0.02 (−0.15,0.12)	⊕⊕⊕⊝ moderate^a^
Height (change in cm)	3,851 (5 studies)	0.02 (−0.07, 0.11)	⊕⊕⊕⊝ moderate^a^	−0.03 (−0.27,0.22)	⊕⊕⊕⊝ moderate^a^
Hemoglobin (change in g/L)	3,851 (5 studies)	2.18 (1.02, 3.35)	⊕⊕⊕⊝ moderate^a^	1.98 (0.74,3.21)	⊕⊕⊝⊝ low^a^, ^b^
GRADE Working Group grades of evidence					
**High quality:** Further research is very unlikely to change our confidence in the estimate of effect.					
**Moderate quality:** Further research is likely to have an important impact on our confidence in the estimate of effect and may change the estimate.					
**Low quality:** Further research is very likely to have an important impact on our confidence in the estimate of effect and is likely to change the estimate.					
**Very low quality:** We are very uncertain about the estimate.					

*Note*: Findings based on the analysis of main effects of 19 studies providing individual participant data.

Abbreviations: CI, confidence interval; STH, soil transmitted helminth.

^a^ Downgraded for study limitations—obtained only a selected sample of IPD from 19 out of 41 eligible studies.

^b^ Downgraded for imprecision.

## 2 BACKGROUND

### 2.1 The problem, condition or issue

Soil transmitted (or intestinal) helminths and schistosomes affect millions of children worldwide. There are four species of STH: *A. lumbricoides* (roundworm), *Necator americanus* and *Anyclostoma duodenale* (hookworms), and *Trichuris trichiura* (whipworm). The five species of schistosomes which affect humans include: *Schistosoma mansoni*, *Schistosoma japonicum*, *Schistosoma mekongi*, *Schistosoma intercalatum* (which causes intestinal schistosomiasis) and *Schistosoma haematobium* (which causes urinary schistosomiasis).

Mass deworming is applied widely to reduce the consequences of helminth infection, and there have been numerous studies on the effects of deworming on growth, cognition and learning outcomes in children over the past several decades. Systematic reviews and meta‐analyses based on aggregate results of the effect of mass deworming on health and education outcomes are conflicting with some showing benefit (Croke, Hicks, Hsu, Kremer, & Miguel, 2016; Hall, Hewitt, Tuffrey, & de, 2008) and others not (Taylor‐Robinson, Maayan, Soares‐Weiser, Donegan, & Garner, 2015; Welch et al., 2017). Debate has ensued about whether these conflicting results are due to the influence of variations in effect across individual‐level characteristics such as whether children are infected or not and intensity of infection (Bundy, Kremer, Bleakley, Jukes, & Miguel, 2009; Hotez et al., 2007; Montresor et al., 2015) as well as setting characteristics such as the sanitation environment and rapidity of reinfection (Campbell et al., 2016).

### 2.2 The intervention

Mass deworming for STH infection and schistosomiasis is recommended one to four times per year in order to reduce worm burden in endemic areas in the updated World Health Organization guidelines, depending on prevalence of worm infection (WHO, 2017). These updated WHO guidelines cite the Campbell and Cochrane systematic reviews on deworming which both concluded there was little to no effect of deworming on child health outcomes which included growth, anaemia and cognitive outcomes (Taylor‐Robinson et al., 2015; Welch et al., 2016). Mass deworming can be applied to school‐aged children or whole communities. Selective treatment of infected individuals is rarely done due to the high cost of screening for infection.

The drugs used include albendazole, mebendazole, levamisole, ivermectin and piperazine for STH infection and praziquantel for schistosomiasis. These drugs are usually provided as pills, are inexpensive and can be administered by schoolteachers or parents. The drugs are considered to have few minor and transient side effects, such as gastrointestinal discomfort, headache, nausea, dizziness, oedema, myalgia and vomiting (WHO, 2017).

Mass deworming is sometimes accompanied by iron, micronutrient or food supplementation in order to correct nutritional deficiencies that may have been caused by worm infections (de Gier, Campos Ponce, van de Bor, Doak, & Polman, 2014; Friis et al., 2003; Nga et al., 2009; Rajagopal, Hotez, & Bundy, 2014; Taylor, Jinabhai, Kleinschmidt, & Jogessar, 2001). In addition, water and sanitation measures may be implemented with mass deworming to reduce exposure and transmission of infections.

### 2.3 How the intervention might work

Even with heavy infections, the nutritional requirements of intestinal worms relative to their human hosts are small. The harm to child welfare is expected to be caused by three factors: (a) malabsorption, (b) tissue damage and bleeding and (c) loss of appetite (Crawley, 2004). STH infections may cause malabsorption of nutrients in their hosts because of damage to the gastrointestinal surfaces. Hookworm infections are associated with anaemia, thought to be due to hookworm feeding on host tissue and to bleeding when they move from one site to another (Hall et al., 2008). Intestinal infections may also lead to reduced appetite which may negatively influence both growth and attention in school.

**Figure 1:**
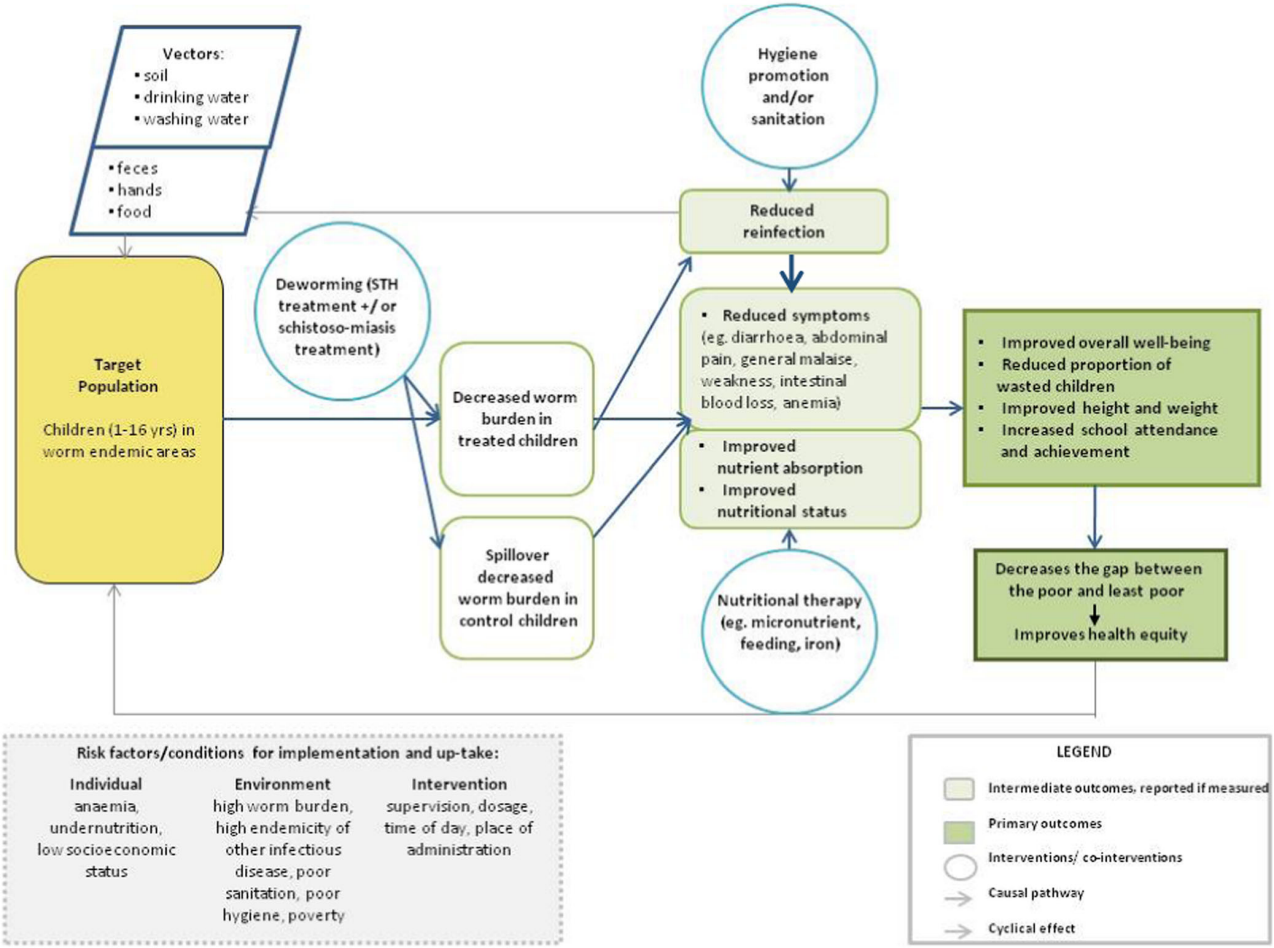
Logic model for deworming effects

Deworming drugs are over 90% effective at reducing the worm load in individuals and are expected to reduce the prevalence of worm infection in the community as well as the intensity of infection in individuals (Figure 1). Reducing the prevalence and intensity of infection is expected to improve child nutritional status due to the mechanisms described above of reducing blood loss, reducing damage to gastrointestinal surfaces and improving appetite. Improved nutritional status and appetite are expected to improve attention in school and cognitive outcomes. Some have argued that deworming alone is insufficient to improve child health outcomes since the nutritional deficiencies caused by infections must be corrected with food and/or micronutrients (Hall et al., 2008).

Many potential effect modifiers have been described in the literature. Younger children may have a greater impact of deworming since they are smaller in size and the impact of infections may be greater on them (Hall et al., 2008). Girls may benefit less from deworming if they have lower school attendance (thus, not receiving deworming given at school) and if there is preferential distribution of food or other resources at home which could influence child welfare. Children who are stunted for age at three years of age may not be able to benefit as much in terms of growth. Conversely, children who are underweight may benefit more from deworming than those of normal weight (Hall et al., 2008). It is expected that benefits of deworming would only accrue to those who are infected, and even more so to those with heavier infection intensity (Hall et al., 2008). Low socioeconomic status is expected to be correlated with other features such as exposure to repeat intestinal infections, including those that cause diarrhoea, and thus children with lower socioeconomic status may not achieve as much benefit as less poor children.

Reinfection is expected to depend on the prevalence and intensity of infection as well as environmental factors such as the water and sanitation environment and hygiene practices in the community.

### 2.4 Why it is important to do the review

A recent Campbell systematic review and network meta‐analysis (NMA) by members of our team (V. W., P. T., G. A. W., E. G., Z. B.), with 47 randomised trials and >1 million children, found little to no overall effect on growth, attention and school attendance (Welch et al., 2016). With NMA, we were able to explore the size of effect with different types and frequency of drugs and their combination with food or micronutrients; none of which contributed to larger effects. Our review also did not find larger effects in subgroups of children at the aggregate level across characteristics such as age, baseline nutritional status, prevalence or intensity of infection that have been postulated to be important (Welch et al., 2016). These analyses were conducted at the study level, rather than using data for each individual child, which limits the power to detect effect modification by individual participant characteristics. This review was therefore unable to identify whether mass deworming was more effective for children with certain characteristics. There was substantial unexplained heterogeneity between studies, with some studies finding larger effects than others, and no single individual‐level, setting‐level or methodology characteristic explaining this variation. Thus, we concluded that our analysis of effect modifiers was limited by the aggregate level data.

Our previous review was conducted using NMA, which allowed the comparison of treatments which had not been directly compared in head‐to‐head trials. NMA also allowed for the assessment of the role of multicomponent interventions (such as deworming combined with other parasite control interventions, food or micronutrients). Because there are several drugs used for mass deworming, this allowed the assessment of heterogeneity related to the type of drug, frequency and use of concomitant interventions.

IPD meta‐analysis has been called the “gold standard” in meta‐analyses for exploring individual level characteristics and their association with effects (Stewart, 1995). Advantages of IPD meta‐analysis include improving data quality, enabling standardisation of outcomes, clarifying risk of bias and increasing the power to assess the interaction of participant characteristics with effect size (Dagne, Brown, Howe, Kellam, & Liu, 2016; Stewart et al., 2015). Furthermore, IPD analysis can explore the size and direction of differences in effect, thus assessing whether there is a greater benefit for some participants (Early Breast Cancer Trialists' Collaborative Group, 1990). Another advantage of IPD is that they usually require an international collaborative effort, involving trial authors, who may help to identify more relevant trials, and also contribute to an agreed analysis plan and shared understanding of the results.

While failure to obtain some datasets may lead to selection bias if there are systematic reasons why some studies do not provide full data, methods have been developed to combine IPD with aggregate data (when IPD is not available for some studies) in NMA (Donegan, Williamson, D'Alessandro, & Smith, 2012; Sutton, Kendrick, & Coupland, 2008).

We decided in collaboration with several authors of primary trials that there would be value in conducting an IPD meta‐analysis to explore the question of whether mass deworming is more effective for subgroups of children defined by characteristics such as infection intensity or status, age or nutritional status. This understanding could help to develop targeted strategies to reach these children better with deworming and guide policy regarding deworming.

## 3 OBJECTIVES

The primary objective is to use IPD NMA to explore whether the effects of different types and frequency of deworming drugs as well as their combination with food or micronutrients on anaemia, cognition and growth vary with child‐level and study‐level characteristics (see Table 1), specifically: intensity of infection (as assessed by egg count), infection status (including species of worm), age, nutritional status, socioeconomic status and sanitation environment.

**Table 1:** Potential effect modifiers at child‐level and environment level

Child‐level	Environment^a^
Age	Population level prevalence
Sex	Population level intensity
Nutritional status	Water and sanitation environment
Infection status	
Socioeconomic status	
Intensity of infection (including type of worm and duration of infection)	

^a^ Environment‐level factors were not entered into the same model as individual‐level modifiers because these factors are likely multicollinear. Instead, we planned to explore these factors with sensitivity analysis.

## 4 METHODOLOGY

The protocol was registered with the Campbell Collaboration (Welch et al., 2016) and reported according to the preferred reporting items for systematic reviews and meta‐analyses for protocols (PRISMA‐P; Moher et al., 2015). Results of the review are reported using the PRISMA of individual patient data (PRISMA‐IPD) Statement (Stewart et al., 2015) and the PRISMA for network meta‐analyses (PRISMA‐NMA).

### 4.1 Criteria for including and excluding studies

We included studies which met the following eligibility criteria:

#### 4.1.1 Types of study designs

We included randomised and quasirandomised trials. For the purpose of determining whether specific individual‐level and environment‐level characteristics are associated with greater effects of deworming, there is sufficient evidence from over 70 randomised trials with over 100,000 children to include only randomised and quasirandomised trials. We included studies reported in abstract form at a conference as well as unpublished studies. We sought full datasets from all studies and carried out the same methods for data checking and quality for all studies.

#### 4.1.2 Types of participants

Children aged 6 months up to 16 years. We excluded studies with <100 participants because of the time and effort required for each dataset and the information gained from smaller studies would be small compared to larger datasets. We did not exclude studies on the basis of attrition rate from the study.

#### 4.1.3 Types of interventions

Mass deworming using any drugs for STH or schistosomes with or without cointerventions such as food, micronutrients, iron or hygiene interventions. Eligible drugs include (but are not limited to) albendazole, praziquantel, levamisole, ivermectin, diethyl carbamazine, pyrantel, piperazine, metrifonate, hycanthone and tetramisole.

We included studies with combined approaches to parasite elimination such as albendazole and praziquantel. Also, because deworming may be used in combination with iron, food or hygiene promotion, we included studies with multiple component interventions.

Studies were included with placebo, control, or other active interventions (e.g., vitamin A, iron, hygiene promotion) as comparators.

As NMA depends on the assumption of transitivity (that participants could be randomised to any one of the treatments; Salanti, 2012), we planned to conduct two evidence networks of jointly randomizeable interventions of drugs given for two indications. First, we assessed the evidence network of interventions given for STH which includes different frequencies of albendazole, mebendazole, levamisole, pyrantel, piperazine, ivermectin and tetramisole with or without micronutrients or food. These are considered jointly randomizable because they are given for the same indication, and many have been compared in multiarm trials (Salanti, 2012).

Secondly, we considered the evidence network of interventions given for schistosomiasis (praziquantel, metrifonate, hycanthone) with or without micronutrients or food.

#### 4.1.4 Types of outcome measures

The primary health outcomes were change from baseline in: weight (kg), height (cm), plasma ferritin, cognition and haemoglobin (g/L). We included studies which measured weight, haemoglobin, plasma ferritin, cognition or height. Cognition could be measured using scales that measured development (e.g., Raven's matrices) or tests that assessed attention using digit recall.

We did not exclude on the basis of reported outcomes since some measured outcomes may not be reported in trial reports or abstracts.

We used the available data on age and sex to calculate height for age, weight for age and weight for height for children <5 years using the 2006 child growth standards (using WHO software Anthro version 3.2.2) and body mass index (BMI) for age for children aged five or older using the WHO Reference 2007 (using WHO AnthroPlus software).

Effects on infection intensity and status were assessed as secondary outcomes. Adverse effects of deworming were assessed in prior systematic reviews as minor and uncommon and the results are not contested, thus we did not assess adverse effects in this review.

Since the primary objective of this systematic review is to assess effect modification, particularly as it relates to infection status and intensity, we excluded studies that did not measure baseline infection prevalence of at least one of the STH or schistosomes.

#### 4.1.5 Duration of follow‐up

For weight and height, we included data from studies >4 months in duration because we considered this as a minimum duration to observe differences in growth based on clinical expertise of nutritionists on the team and decided a priori. However, for haemoglobin and ferritin status, changes may occur sooner, so study duration was not be used as an exclusion criterion. While infection status and infection intensity are affected much sooner than this, these are not primary outcomes of interest since there is no question that deworming drugs reduce infection load. We assessed infection intensity and status at baseline as indicators of the force of infection in the population. We collected data at each available time‐point and aimed to explore study duration as a covariate in the model.

#### 4.1.6 Types of settings

The settings included any area where STH or schistosomes were described as endemic.

### 4.2 Search strategy

We adapted the search strategy used for a previous Campbell review by members of our team (Welch et al., 2016) and updated the search to March 27, 2018. See search strategy in Appendix. We searched in the following databases: MEDLINE, CINAHL, LILACS, Embase, the Cochrane Library, Econlit, Internet Documents in Economics Access Service (IDEAS), Public Affairs Information Service (PAIS), Social Services Abstracts, Global Health CABI and CAB Abstracts.

We searched the System for Information on Grey Literature in Europe (SIGLE)‐ended in 2005. We searched websites of relevant organisations such as the World Bank, World Food Program and International Food Policy Research Institute, as per the prior Campbell review (Welch et al., 2016).

We also contacted authors of studies and members of our advisory board for any unpublished studies or grey literature reporting eligible studies. We checked reference lists of relevant studies and reviews.

Titles and abstracts were screened in duplicate by two reviewers. We pilot‐tested the screening criteria at both title and abstract screening stage and full text stage. We used the PRISMA flow diagram to report eligibility of studies. We retrieved full text of all studies which passed this first level screening. The full text review was also done in duplicate by two reviewers, and agreement was reached by consensus. Disagreements were resolved by consultation with a third reviewer. No language limits were applied. The research team had expertise in English, Portuguese, French and Spanish, and translation would have been sought if studies were found in other languages.

### 4.3 Description of methods used in primary research

Randomized controlled trials (RCTs) of deworming include two‐arm trials as well as factorial trials, with children allocated either individually or by cluster‐randomisation (e.g., by village or school).

### 4.4 Details of study coding categories

Details of the populations, interventions, comparators, outcomes and study design were extracted in duplicate by two reviewers, using a pretested form, designed for a previous Campbell review on deworming for children (Welch et al., 2016). This extraction includes details about the context, setting and environment, as well as sociodemographic details, and details about the frequency, delivery method and dose of interventions.

Two independent reviewers appraised each study with the Cochrane risk of bias tool which assesses selection bias, performance bias, detection bias, attrition bias and reporting bias (Cochrane Handbook; Higgins, Altman, & Sterne, 2011). Disagreements were resolved by discussion or consultation with a third reviewer.

We appraised the GRADE certainty for each outcome for each comparison by two independent reviewers, using the GRADE approach for NMA (Puhan et al., 2014). GRADE certainty (quality) “reflects our confidence that the estimates of the effect are correct. In the context of recommendations, quality reflects our confidence that the effect estimates are adequate to support a particular recommendation. “Quality as used in GRADE means more than risk of bias and so may also be compromised by imprecision, inconsistency, indirectness of study results, and publication bias.” (Balshem et al., 2011). The two reviewers discussed ratings and reached consensus. Disagreements were resolved by consulting a third reviewer.

We developed a summary of findings table for each main comparison to show the effects for the outcomes of weight, height and haemoglobin, along with the quality of evidence (using GRADE certainty).

### 4.5 Statistical procedures and conventions

Data were prepared into a Microsoft Excel spreadsheet with the same fields for every study. Since we only included RCTs, we considered the missing values for each variable as missing at random (MAR) based on observed data (Joshi, Royuela, & Zamora, 2013).

We used multiple imputation to impute the missing values for baseline and outcome variables based on the assumption of MAR (Bell, Fiero, Horton, & Hsu, 2014; Groenwold, Moons, & Vandenbroucke, 2014; Jakobsen, Gluud, Wetterslev, & Winkel, 2017) and created five complete datasets using Proc MI in SAS9.4/STAT (SAS Institute Inc., Cary, NC). All model estimates and standard errors were obtained by fitting the model to each of these five imputed datasets and aggregating results across them using Rubin's Rule which incorporates uncertainty due to imputation. Proc MIANALYZE in SAS 9.4/STAT was used to obtain the aggregation of estimates across imputed datasets.

Descriptive characteristics of each study are presented, with details on the child characteristics, environment, worm species, prevalence, and intensity of infection, geographic location, interventions, comparator and outcomes and risk of bias assessment.

We accounted for clusters (such as villages, schools or households) as nested within each study.

We analysed IPD datasets to check for comparability with the primary published papers. We calculated the standardised difference between the published data and the IPD received from authors for baseline characteristics and baseline outcome assessment. For endline, we replicated the effect measures reported in study publications and calculated the standardised difference between the IPD received and the study report (Austin, 2009).

As with our previous Campbell review, we used a two‐step process to meta‐analysis. We conducted pairwise analyses for each comparison of interest by entering all IPD data into a multilevel model, with each study as one cluster. We expected considerable heterogeneity between studies for each outcome based on our Campbell review; therefore, we used a random effects model. We assessed mean differences in change from baseline for weight (kg), height (cm) and haemoglobin (g/L). We intended to assess plasma ferritin (mcg/L) but too few studies reported this outcome (seven studies with 6,318 participants). The Advisory board, based on clinical and methodological expertise, decided that there were insufficient studies to conduct effect modification analyses and that basic random effects meta‐analysis could be misleading.

For cognition, we analysed measures of motor and cognitive development separately. We analysed measures of attention separately from developmental outcomes. We did not combine different measures of cognition.

We accounted for clustering as above by nesting clusters within studies. We decided on a set of predefined covariates with advice from our advisory board and coauthors. We accounted for the covariates of sex, age, infection intensity for each type of agent, socioeconomic status, maternal education and baseline nutritional status in the model. We assessed heterogeneity using visual inspection of forest plots for pairwise analyses as well as statistical tests of heterogeneity (*I* ^2^).

We conducted NMA with IPD, using a frequentist approach for random‐effects NMA. The covariates were identified by the Study Advisory Group, namely: age, sex, baseline nutritional status (weight and height), haemoglobin and infection intensity. The effect estimate chosen was the mean difference for the continuous outcome of interest. The general linear mixed model (GLMM) follows a normal distribution using a mixed linear regression model. Random effect GLMM was conducted with two random effects considered in the model: random effect “trial” accounts for the response variables of patients within a given trial being correlated; and random effect “Patient's clusters” which accounts for the correlation of responses between any two patients from the same clusters (such as villages, schools or households) within a given trial. We expected to have a connected network of trials to allow direct and indirect comparisons based on our Campbell review and NMA (see Figure 12, full evidence network for weight; Welch et al., 2016). We used the GLIMMIX procedure in SAS 9.4/STAT (SAS Institute Inc.) for the GLMM NMA, considering models that account for multiarm trials and adjust for the covariates identified. Results are summarised as point estimates with 95% confidence intervals (CIs).

### 4.6 Assessment of clinical and methodological heterogeneity within treatment comparisons

Within GLM, the explanatory model included covariates at the study level (e.g., methodological quality) and participant characteristics (anaemia, nutritional status, infection intensity, age, sex). We constructed forest plots for unadjusted direct treatment comparisons and adjusted treatment comparisons and assessed heterogeneity by visual inspection. Any study level or participant‐level covariates that were statistically significant would have been analysed using subgroup analyses. We compared participant characteristics and trial methodology in tables.

### 4.7 Assessment of transitivity across treatment comparisons

Transitivity cannot be assessed statistically. With IPD, we have more opportunity to account for and model heterogeneity. As proposed by Salanti (2012), we used IPD to assess the distribution of the child‐level effect modifiers from Table 1 in each comparison to assess the plausibility of the transitivity assumption (Salanti, 2012). As above, transitivity is considered plausible since the treatments in each model (STH and schistosomiasis, respectively) are provided for the same indication and many of the treatments and their cointerventions have been included in multiarm trials (as shown by the prior review by our team: Welch et al., 2016).

### 4.8 Assessment of statistical heterogeneity

For this IPD NMA, we assumed equal variances across comparisons within network. This assumption was tested using the Levene test.

### 4.9 Assessment of statistical inconsistency

Inconsistency in an NMA is defined as a disagreement between the direct estimates (from direct comparisons of treatments) and indirect estimates (which are derived from the network comparisons). With GLMM we were unable to perform a test for model fit and consistency therefore we assessed underlying assumptions about consistency between indirect and direct evidence by comparing direct effect estimates with NMA effect estimates (direct and indirect evidence).

### 4.10 Publication bias

A funnel plot would have been plotted for comparisons and outcomes with >10 studies. We used Egger's test for asymmetry and visual inspection to assess the presence of publication bias and/or selective reporting in the entire corpus of randomised trials of deworming versus control for children (which includes some studies that were not eligible due to missing baseline data on infection intensity and some studies which were eligible but did not provide data).

We did not rank interventions because there is controversy as to the utility of ranking.

### 4.11 Subgroup analyses

Provided sufficient data was available to inform the evidence network, subgroup analyses were conducted to assess effects across both child‐level as well as environment‐level characteristics. We compared the results of models with subgroup analyses by assessing the size of quantitative or qualitative differences in effects, the statistical significance of tests for interactions, assessing between‐study variance and assessing the goodness of fit of the models using the likelihood ratio.

The following child and environment level effect modifiers were planned:

Child level:

- Individual‐level intensity of infection with *ascaris, trichuris* and hookworm (across four levels of none, light, moderate and heavy, using the WHO cutoffs for each helminth (http://apps.who.int/iris/bitstream/10665/44671/1/9789241548267_eng.pdf)
- Stunting (HAZ>  −2.0, HAZ < −2.0 to −3.0, HAZ < −3.0)
- Undernutrition (defined by WAZ cutoffs for children <5 years of age (http://apps.who.int/iris/bitstream/10665/44129/1/9789241598163_eng.pdf?ua=1) of WAZ > −2.0, WAZ < −2.0 to −3.0, WAZ < −3.0) and by BMI for age (BAZ) cutoffs for children aged 5 years or older available at http://www.who.int/growthref/who2007_bmi_for_age/en/) using BAZ > −2.0, BAZ < −2.0 to −3.0, BAZ < −3.0),
- Anaemia (using WHO cutoffs by age and altitude of nonanaemic, mild, moderate and severe, http://www.who.int/vmnis/indicators/haemoglobin.pdf)
- Age (<5 and ≥5 years of age)
- Sex (male/female)
- Socioeconomic status: socioeconomic status is measured in different ways in studies (e.g., questionnaires, asset indices, quintiles). We planned to assess whether the measurement of socioeconomic status could be compared across study settings and time. We decided this was not possible therefore we did not do a planned sensitivity analysis with children in the poorest tertile.

Before conducting subgroup analyses, we assessed the distribution of each variable. If there were insufficient children in some categories, the levels were combined (see results).

We planned to assess socioeconomic status of household or parents and maternal education as effect modifiers, but data was insufficient (see results).

Environment level:

- Study level sanitation and hygiene environment, as reported by studies was assessed to consider whether environments can be classified according to consistent system
- Study‐level prevalence (using WHO cut‐offs for each worm‐type, as above)
- Study‐level intensity of infection (using WHO cut‐offs for each worm‐type, as above)

As noted in Table 1, environment level characteristics were not entered into the model. They were assessed by sensitivity analyses.

We expected poor reporting on these details in the articles based on our prior Campbell review, but some studies may have collected information on this at the study level that were not reported in the paper publications. We assessed whether there was sufficient data on the geographic location and date of the studies to assess study‐level prevalence generated by the Global Atlas of Helminth Infections.

### 4.12 Sensitivity analyses

Provided sufficient data was available to inform the evidence network, we conducted sensitivity analyses to assess robustness of results when restricted to studies at low risk of bias for sequence generation, allocation concealment and blinding of participants. We assessed whether results were robust to excluding imputed data (i.e., complete case analysis). We assessed sensitivity to restricting to studies published in 2008 or later (last 10 years).

Data were housed at a secure data warehouse at the Bruyère Research Institute, following the personal health information act. Data were transferred to SAS as a common platform for all studies, using a common data dictionary. V. W. checked IPD data for consistency immediately upon receiving datasets. For example, we checked for outlier individuals (e.g., with ages outside of eligibility criteria, duplicate participant IDs, unrealistic date ranges). We compared the IPD from authors with the aggregate data reported in the articles. Any missing or unusual data were flagged for discussion with the trial author or statistician by V. W. We asked for clarification from the authors to establish reasons for the errors, and corrected them if possible. Any requests for authors were discussed when the data was provided, such as clarification of trial risk of bias, conduct or eligibility criteria. We ran the same statistical analysis as the authors to check for consistency with the published paper (Stewart et al., 2015).

We requested statements of ethics approval from each study. No studies were identified that did not receive ethics approval. We requested that all data be transferred without any identifiers.

### 4.13 Treatment of qualitative research

We did not include qualitative research.

## 5 RESULTS

The results of this review are reported according to the PRISMA‐IPD and PRISMA‐NMA reporting guidelines (checklists in Table S1).

### 5.1 Search results

We searched all databases up to March 27, 2018. We also retrieved in full text all 299 primary studies included in eight previous reviews (Danso‐Appiah, Olliaro, Donegan, Sinclair, & Utzinger, 2013; Grimes et al., 2014; Kramer, Zhang, Sinclair, & Olliaro, 2014; Salam, Haider, Humayun, & Bhutta, 2015; Salam, Maredia, Das, Lassi, & Bhutta, 2014; Strunz et al., 2014; Taylor‐Robinson et al., 2015; Welch et al., 2016).

**Figure 2:**
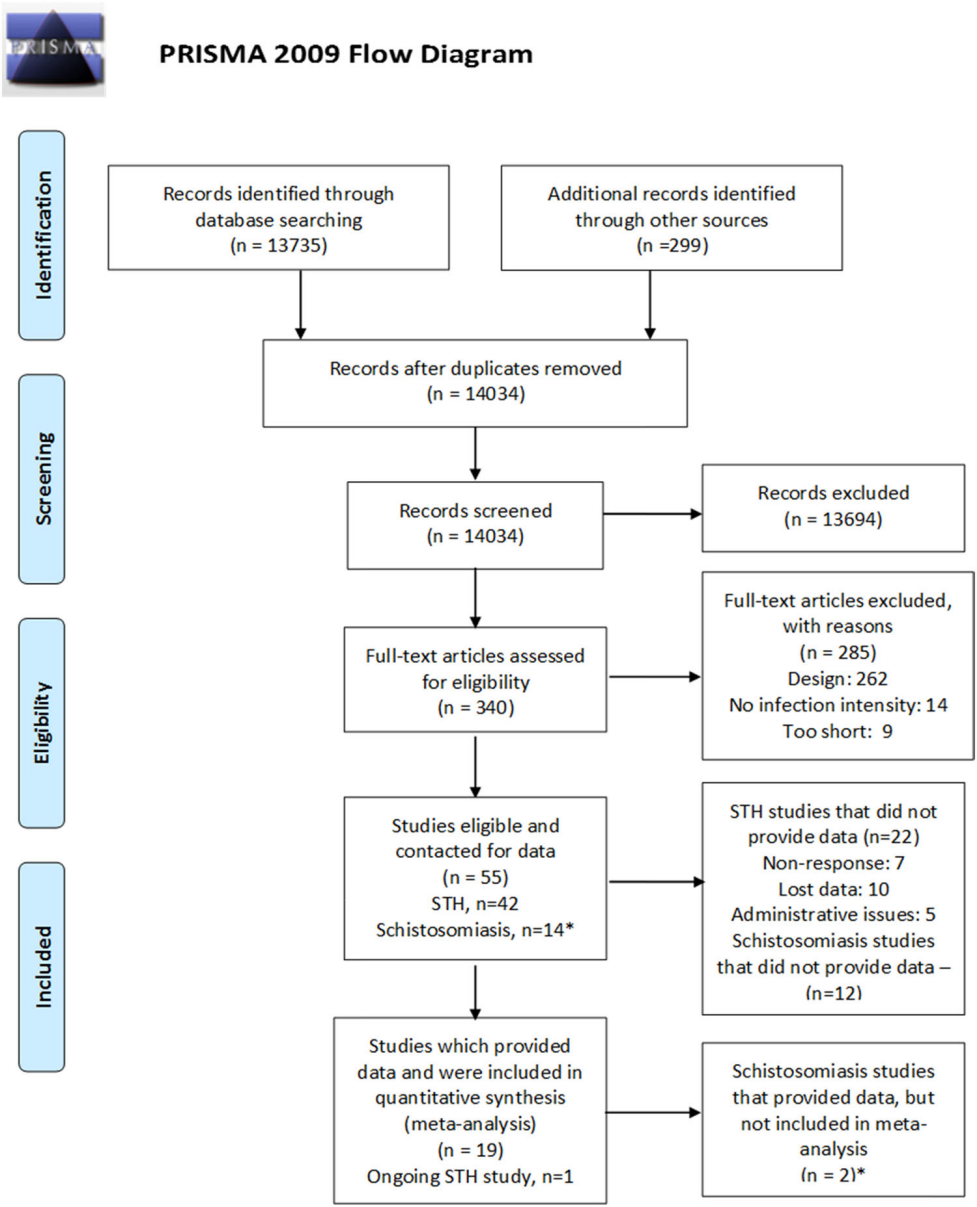
PRISMA flow diagram. *Number of STH studies and schistosomiasis studies adds to 56 (not 55 as in the figure) since one study (Olds et al., 1999) is counted as both STH deworming and schistosomiasis deworming because it is a factorial trial. STH, soil‐transmitted helminthiasis

We screened 14,034 records for inclusion. We screened 340 studies in full‐text. We assessed 41 studies of deworming for STH and 14 studies of schistosomiasis treatment as eligible for inclusion. One study included treatments for both STH and schistosomiasis (Olds et al., 1999), and is included in both counts (Figure 2).

A total of 285 studies were excluded because they did not meet eligibility criteria, due to lack of infection intensity data (*n* = 14), <3 months (*n* = 9) and wrong study design (*n* = 262; Table S3). We identified one ongoing study of albendazole (Table S4).

### 5.2 Contacting authors and yield of studies

We contacted first authors of all eligible studies by email outlining the study purpose and inviting them to join the Deworming Collaborative. If there was no reply, contact with all authors was then made by email or using other contact information such as Researchgate and twitter. Authors were contacted in their language if possible (French, Portuguese).

**Figure 3:**
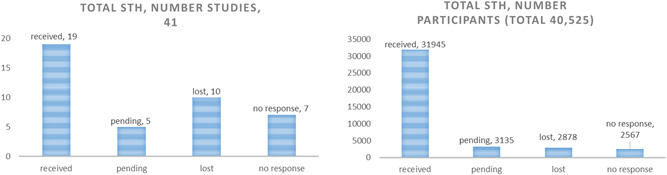
Yield of STH studies and participants. STH, soil‐transmitted helminthiasis

For STH, we received complete data from 19 out of 41 published studies (46%) (Tables S5), which represented 79% of all children included in those eligible STH trials (Figure 3).

**Figure 4:**
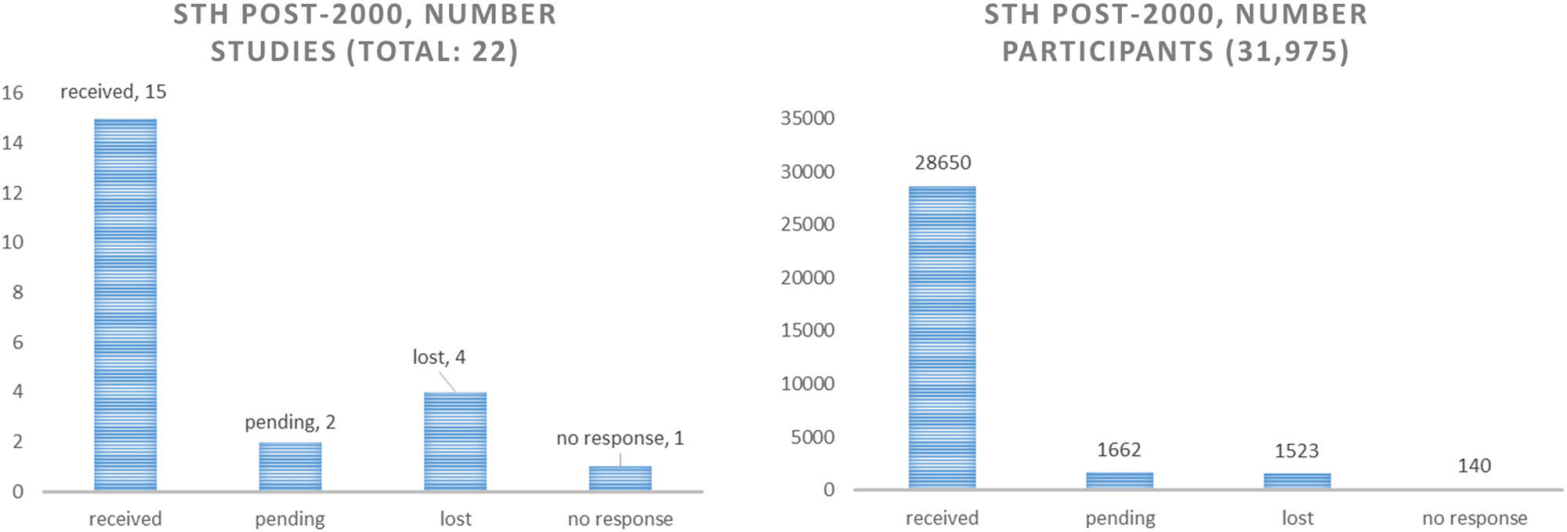
STH yield of studies and participants for studies post‐2000. STH, soil‐transmitted helminthiasis

The retrieval of data was better for studies conducted after 2000, with a yield of 15 out of 22 published studies (68%) and 90% of participants randomised to eligible studies (Figure 4).

**Figure 5:**
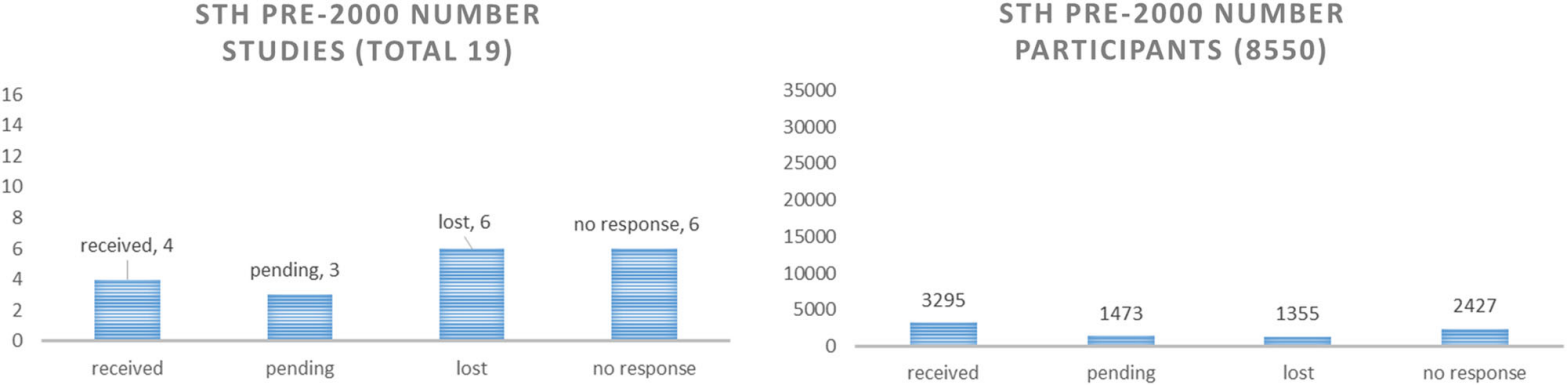
STH studies pre‐2000. STH, soil‐transmitted helminthiasis

For studies conducted before 2000, we received only four out of 19 studies (21%), and 39% of participants randomised (Figure 5).

**Figure 6:**
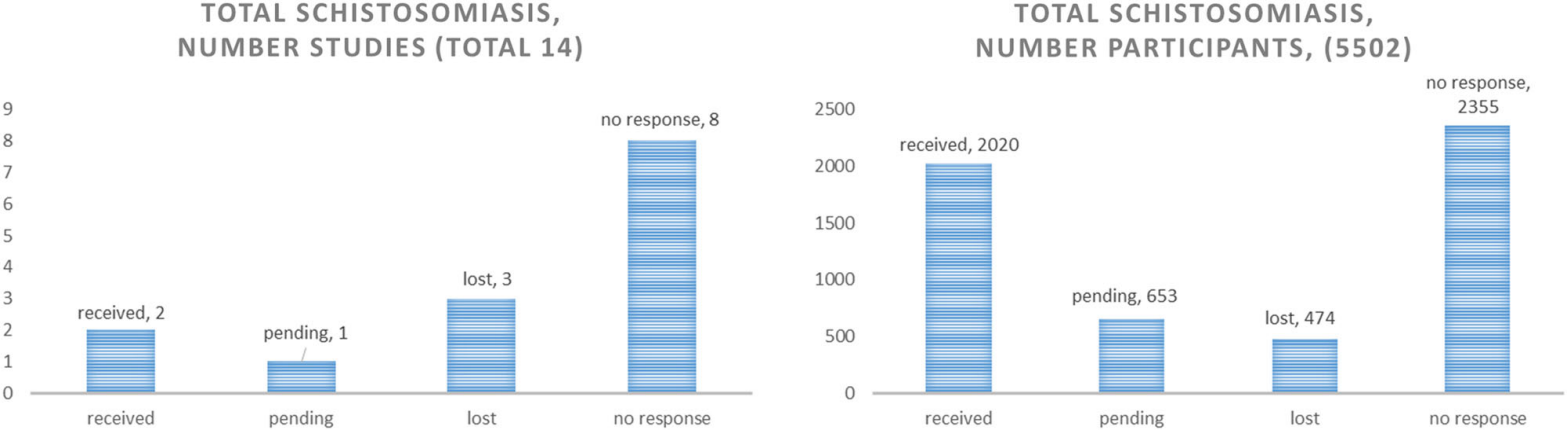
Schistosomiasis study yield

For schistosomiasis, we received data from only two out of 14 studies (14%) (Table S6), representing 37% of participants randomised to eligible studies (Figure 6). We decided not to pursue an analysis of schistosomiasis studies because of the risk of misleading results with an inadequate representation of available studies.

All study authors who provided data signed a data transfer agreement (Appendix 2).

### 5.3 Characteristics of studies

#### 5.3.1 Studies contributing data: Settings, participants, size of studies

The 19 studies which provided data were conducted in Tanzania (Beasley et al., 1999; Beasley, 1995; Stoltzfus et al., 1997, 2004), Sri Lanka (Ebenezer et al., 2013), Kenya (Friis et al., 2003; Miguel & Kremer, 2004; Olds et al., 1999), Vietnam (Hall, Hanh, Farley, Quynh and Valdivia, 2006, Le Huong, Brouwer, Nguyen, Burema & Kok, 2007; Nga et al., 2009), China (Liu et al., 2017; Yap et al., 2014), Cote d'Ivoire (Rohner et al., 2010), Bangladesh (Rousham and Mascie‐Taylor, 1994), Indonesia (Wiria et al., 2013), Nigeria (Kirwan et al., 2009), Uganda (Ndibazza et al., 2012) and the Philippines (Solon et al., 2003) (Tables 2 and S7).

**Table 2:** Description of included studies which provided individual participant data on STH deworming

Study	Country	Design	Type of cluster (number of clusters)	Sample size (number of children per cluster)	Age (years)	Interventions	Frequency, how often dewormed	Duration	Compliance with STH deworming
Beasley et al. (1999)	Tanzania	RCT SAT		357	7–12	1. Albendazole + praziquantel 2. Placebo	5 months	5 months	Not reported
Beasley (1995) (thesis)	Tanzania	RCT SAT		217	6–15	1. Albendazole + praziquantel + iron 2. Placebo	4 months	4 months	100% (full course of 10 doses of iron given on a daily basis was received over 4 weeks due to absenteeism)
Ebenezer (2013)	Sri Lanka	cRCT MDA	Schools (100)	1,570 (20)	9.5	1. Mebendazole + iron tablet 2. Placebo	6 months	6 months	94–98% with deworming, 80% with iron tablets
Friis (2003)	Kenya	RCT MDA		915	8–18	1. Albendazole + praziquantel 2. Albendazole + praziquantel + micronutrient tablet 3. Micronutrient tablet 4. Placebo	8 months	8 months	Not reported
Huong (2007)	Vietnam	RCT MDA		426	7.2	1. Mebendazole + unfortified noodles 2. Mebendazole + iron fortified noodles 3. Mebendazole + iron tablet 4. Iron‐fortified noodles 5. Placebo + unfortified noodles	3 months	6 months	93–99% consumed noodles and iron tablets
Liu (2017)	China	cRCT MDA	Townships (112)	2,179 (20)	10.6	1. Albendazole 2. Control (no deworming)	6 months	12 months	52% fully compliant, 76% took at least one out of two albendazole pills in both rounds
Ndibazza et al. (2012)	Uganda	RCT MDA		2,016	1.52	1. Albendazole 2. Placebo	3 months	45 months	19–22% received all 16 doses (over 5 years)
Nga (2009)	Vietnam	RCT MDA		510	6–8	1. Albendazole + unfortified biscuit 2. Albendazole + micronutrient fortified biscuit 3. Micronutrient fortified biscuit 4. Placebo + unfortified biscuit	4 months	4 months	94% compliant with consuming biscuits
Olds (1999)	Kenya	RCT MDA		371	10.5	1. Albendazole 2. Praziquantel 3. Albendazole + praziquantel 4. Placebo	6 months	12 months	Not reported
Rohner (2010)	Cote d'Ivoire	RCT MDA		311	6–14	1. Iron fortified biscuits 2. Albendazole + praziquantel 3. IPT 4. Albendazole + praziquantel + iron fortified biscuits 5. Albendazole + praziquantel + IPT 6. IPT + iron fortified biscuits 7. Albendazole + praziquantel + IF + IPT 8. Placebo + unfortified biscuits	3 months	6 months	94% for anthelminthics
Solon (2003)	Philippines	RCT MDA		831	9.9	1. Albendazole + unfortified beverage 2. Albendazole + micronutrient fortified beverage 3. Micronutrient fortified beverage 4. Placebo + unfortified beverage	4 months	4 months	Not reported
Stoltzfus et al. (1997)	Tanzania	cRCT MDA	Schools (12)	4,034	10.5	1. Mebendazole (2/year) 2. Mebendazole (3/year) 3. Placebo	6 months 4 months	12 months	Not reported
Stoltzfus et al. (2004)	Tanzania	cRCT MDA	Households (451)	463	6–59 months	1. Mebendazole (high) 2. Mebendazole (high) + iron supplement 3. Iron supplement 4. Placebo	3 months	12 months	Not reported
Yap (2014)	China	RCT SAT		194	9–12	1. Albendazole 2. Placebo	6 months	6 months	92%, field investigators directly observed consumption
Kirwan (2009)	Nigeria	RCT MDA		1,367	1–4	1. Albendazole 2. Placebo	4 months	14 months	Not reported. Consumption was directly observed
Hall (2006)	Vietnam	cRCT MDA	Schools (80)	2,916	6.8	1. Albendazole + vitamin A 2. Placebo + vitamin A	6 months	12 months	Not reported
Miguel (2004)	Kenya	cRCT MDA	Schools (75)	15,881	6–18	1.Albendazole 2. Albendazole + praziquantel 3. Control	3 months	3 months	96%
Rousham (1994)	Bangladesh	cRCT MDA	Villages (7)	124	2–6	1. Mebendazole 2. Placebo	2 months	12 months	Not reported
Wiria (2013)^a^	Indonesia	cRCT MDA	Households (954)	1,854	2–16	1. Albendazole 2. Placebo	3 months	21 months	78% with antheminthic

Abbreviation: MDA, mass drug administration; RCT, randomized controlled trial; SAT, screen and treat; STH, soil‐transmitted helminthiasis.

^a^ Of 4004 people randomized, only 1854 met our age range (6 months to 16 years), and of those 1854, only 738 had baseline data for weight and height.

Three of these studies were screen and treat (SAT) studies: Yap et al. (2014), Beasley et al. (1999) and Beasley (1995). We decided to include these in the model since our model is designed to adjust for infection intensity.

Seven studies were cluster RCTs, with the unit of randomisation as the household (Stoltzfus et al., 2004; Wiria et al., 2013), the village (Liu et al., 2017), and school (Ebenezer et al., 2013; Hall et al., 2006; Miguel & Kremer, 2004; Stoltzfus et al., 1997). The study duration ranged from 4 to 45 months. The median sample size was 486 (range, 124–15,881). Interventions included albendazole, mebendazole, praziquantel at different frequencies, iron supplements, micronutrient tablets and food (noodles or biscuits only) or beverages fortified with micronutrient and/or iron. The median frequency of deworming was every 4 months (range, 2–8 months).

The median age of children in the studies was 10.8 years of age at enrolment (interquartile range, 8.8–13.0) according to IPD. For nutritional status, 16% of the children included in these studies were below −2 for BMI for age, 33% were stunted and 50% were anaemic (Table 3). The prevalence of infection was 45% for *A. lumbricoides* (31% light infections <4,999 eggs per gram of stool [epg], 13% moderate infection intensity from 5,000 to 49,999 epg and 1% heavy infection intensity with >49,999 epg), 52% for *T. trichiura*(38% light intensity <1,000 epg, 14% moderate between 1,000 and 9,999 epg and 0% heavy >10,000 epg) and 45% for hookworm (38% light intensity <2,000 epg, 5% moderate between 2,000 and 3,999 epg and 2% heavy >4,000 epg).

**Table 3:** Characteristics of studies with <50% missing data which were included in the main model

Variable and subgroups	Beasley 99	Beasley TAN	Ebenezer	Friis	Huong	Liu	Nga	Olds 1999	Rohner	Solon	Stolfuz 2004	Stolfuz 1997	Yap	Ndibazza	Sample size
BMI for age (*z* score)															
<−2 (≤−2)	16	17	626	77	102	141	62	41	17	146	12	926	5	66	2,254
>−2	339	200	945	838	324	2,038	448	330	293	685	451	2,665	189	1,950	11,695
	355	217	1,571	915	426	2,179	510	371	310	831	463	3,591	194	2,016	
Height for age (*z* score)															
<−2 (≤−2)	162	67	462	124	129	607	143	102	76	329	221	1,771	113	299	4,605
>−2	193	150	1,109	791	297	1,572	367	269	234	502	242	1,820	81	1,717	9,344
	355	217	1,571	915	426	2,179	510	371	310	831	463	3,591	194	2,016	
Hookworm (epg)															
0‐No	30	35	1,379	411	388	2,162	483	49	135	625	205	214	75	1,514	7,705
1–384	85	70	107	451	21	17	3	176	141	206	256	1,034	98	501	3,166
>384	242	112	85	53	17		24	146	35		2	2,346	21	1	3,084
	357	217	1,571	915	426	2,179	510	371	311	831	463	3,594	194	2,016	
*Trichuris trichiura* (epg)															
0‐No	103	118	1,368	499	113	1,661	236	79	283	528	126	136	11	1,472	6,733
1–288	131	62	168	376	87	497	143	172	27	301	325	736	94	542	3,661
>288	123	37	35	40	226	21	131	120	1	2	12	2,722	89	2	3,561
	357	217	1,571	915	426	2,179	510	371	311	831	463	3,594	194	2,016	
*Ascaris lumbricoides* (epg)															
0‐No	175	188	1,157	788	138	1,508	183	244	284	385	253	959	13	1,462	7,737
1–1,776	11	6	145	50	21	621	19	45	26	363	209	1,175	15	420	3,126
>1,776	171	23	269	77	267	50	308	82	1	83	1	1,460	166	134	3,092
	357	217	1,571	915	426	2,179	510	371	311	831	463	3,594	194	2,016	
Anyworm^a^ (epg)															
0			1,077	217	50	1,268	101	23	129	306	88	12		1,448	4,719
1	173	164	291	639	111	902	184	233	179	514	375	1,682	35	563	6,045
2	184	53	203	59	265	9	225	115	3	11		1,900	159	5	3,191
	357	217	1,571	915	426	2,179	510	371	311	831	463	3,594	194	2,016	
Anaemia^b^															
No 2	247	132	347	299	53	361	127	264	214	318	440	2,785	13	1,380	6,980
Yes 1	110	85	1,224	616	373	1,818	383	107	97	513	23	809	181	636	6,975
	357	217	1,571	915	426	2,179	510	371	311	831	463	3,594	194	2,016	
Age															
<5 years											430	2		2,016	2,448
>5 years	355	137	1,571	915	426	2,179	510	371	311	831	30	3,592	194		11,422
	355	137	1,571	915	426	2,179	510	371	311	831	460	3,594	194	2,016	
Sex															
Male 1	204	71	812	447	211	1,173	243	219	184	434	246	1,864	94	1,041	7,243
Female 0	153	66	759	468	215	1,006	267	152	127	387	217	1,730	100	975	6,622

Abbreviations: epg, eggs per gram of stool; STH, soil‐transmitted helminthiasis.

^a^ “Anyworm” is a variable indicating children with no detected STH infection of any type of STH, light intensity using WHO cut‐offs for each type of STH, or moderate or heavy infection intensity for any type of STH.

^b^ Anaemia cut points defined on the basis of age and sex using WHO guidelines.

Additional child and setting charateristics for the 19 studies with <50% missing data are in Table S2.

#### 5.3.2 Characteristics of STH deworming studies which did not provide data

Characteristics of studies that did not provide data are shown in Table S8. The main difference in these studies is their year of publication since we were more successful at obtaining data from more recent studies.

#### 5.3.3 Compared to the 2016 aggregate data Campbell review

Seventeen studies which were included in our prior Campbell review (Welch et al., 2016) were excluded because they were not randomised or quasirandomised trials (*n* = 2), had no baseline infection intensity data (*n* = 15). These studies are summarised in Table S9.

#### 5.3.4 Aggregate effect estimates of studies not providing IPD

**Figure 7:**
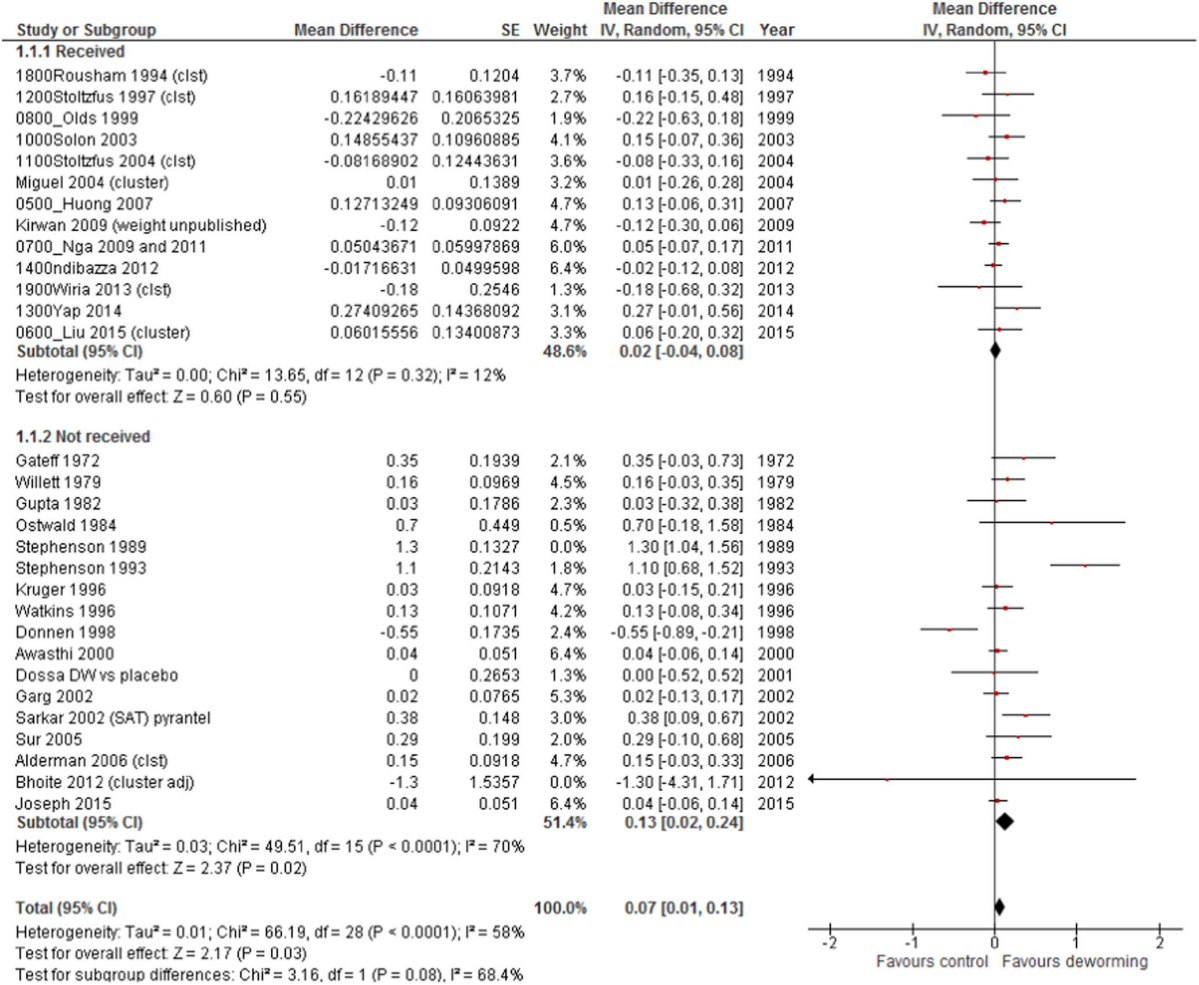
Weight gain (kg) for STH versus placebo for studies providing IPD and studies not included in the IPD analysis. IPD, individual participant data; STH, soil‐transmitted helminthiasis

We compared the effect estimates of the studies which were eligible but did not provide data, those that provided data and those which were not eligible (no infection intensity, too small or too short). Results for STH deworming versus placebo for weight gain (kg) are shown in Figure 7. As can be seen on visual inspection, two studies had much larger effects on weight gain than any others (Stephenson, Latham, Adams, Kinoti, & Pertet, 1993; Stephenson, Latham, Kurz, Kinoti, & Brigham, 1989). The heterogeneity with both of these studies included was 90% as assessed by *I* ^2^, suggesting that statistical combining of these studies is inappropriate. As in our previous Campbell review, we removed outliers to assess contribution to *I* ^2^. Removing Stephenson (1989), which we earlier assessed as having imbalance in baseline covariates which may have influenced results (Welch et al., 2016), resulted in an *I* ^2^ of 71%, which we considered acceptable for statistical pooling, following the Cochrane Handbook guidance (Higgins et al., 2011). The test for interaction of effect was not statistically significant (*p* = .10).

Details for height and haemoglobin for STH versus placebo are shown in Appendix 3, comparison 1. The interaction test for subgroup effects was not statistically significant for any of these outcomes. However, the studies which were not included were older (with 8/16 published before 2000) compared to the studies which provided IPD (3/13 post‐2000). Also, the size of effect was larger for studies which did not provide IPD or were not eligible for weight gain with an effect of 0.07 kg (95% CI: 0.01, 0.13).

### 5.4 Feasibility of conducting IPD meta‐analysis

We judged that we had insufficient data to conduct analysis of the studies of deworming for schistosomiasis since we received only two studies out of 13 eligible for analysis, and this represented 36% of participants randomised to eligible studies.

For deworming for STH, we received IPD from 19 studies out of 41 considered eligible (46%) and 31,945 out of 40,525 participants randomised (79%). We considered this was sufficient data to pursue IPD meta‐analysis.

### 5.5 Quality of studies

**Figure 8:**
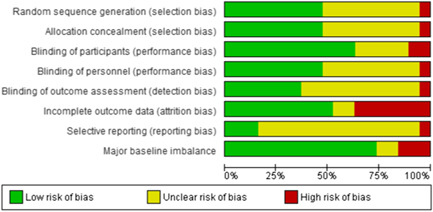
Risk of bias graph for 19 studies that provided data

**Figure 9:**
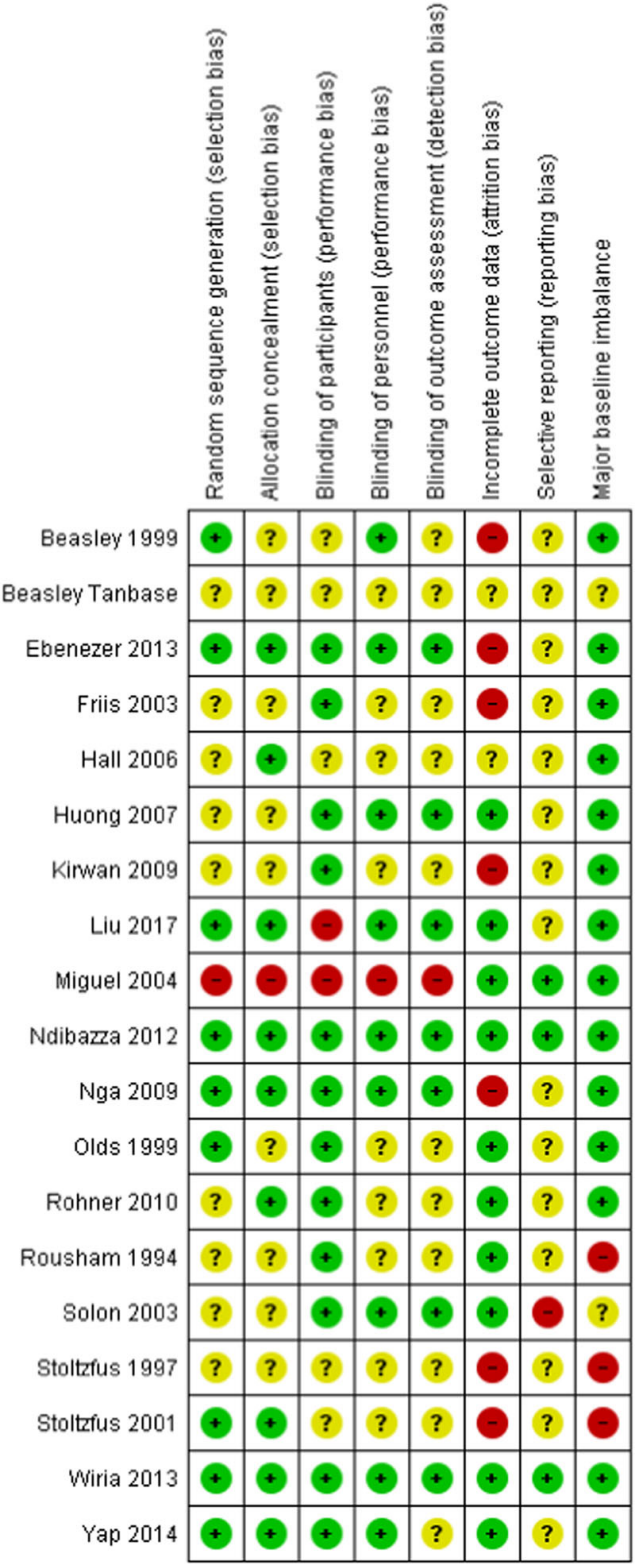
Risk of bias summary for 19 studies that provided data

Overall, there was low risk of selection and performance bias in 47% (9 of 19) studies. 47% (9 studies) had unclear risk of bias due to lack of detail on allocation method or method of blinding. Overall, there was a high risk of attrition bias in 37% (seven studies) of the included studies. Attrition bias was judged high risk due to loss to follow‐up of >20% of participants in these studies. Detection bias could not be assessed in 58% (11 studies) of the studies and selective reporting could not be assessed in 79% (15 studies) due to insufficient information. No major baseline imbalance was found in 74% (14 studies) of the studies, judged according to the description of baseline characteristics (Figures 8 and 9).

**Figure 10:**
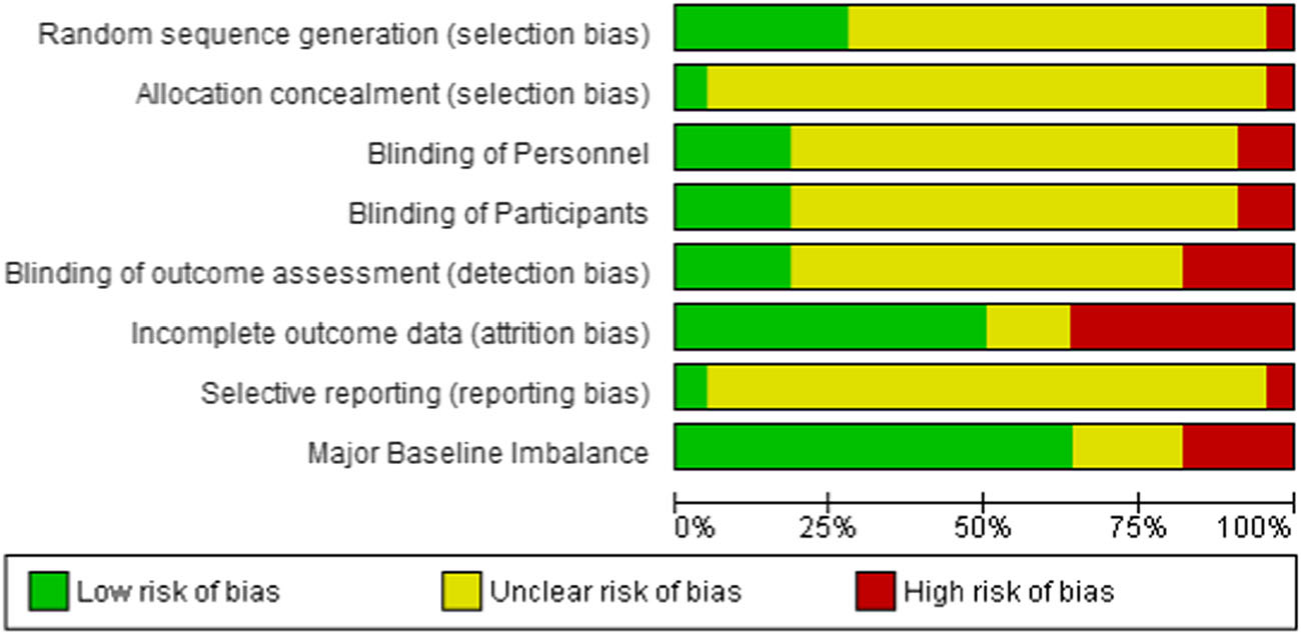
Risk of bias graph for 22 studies that did not provide data

**Figure 11:**
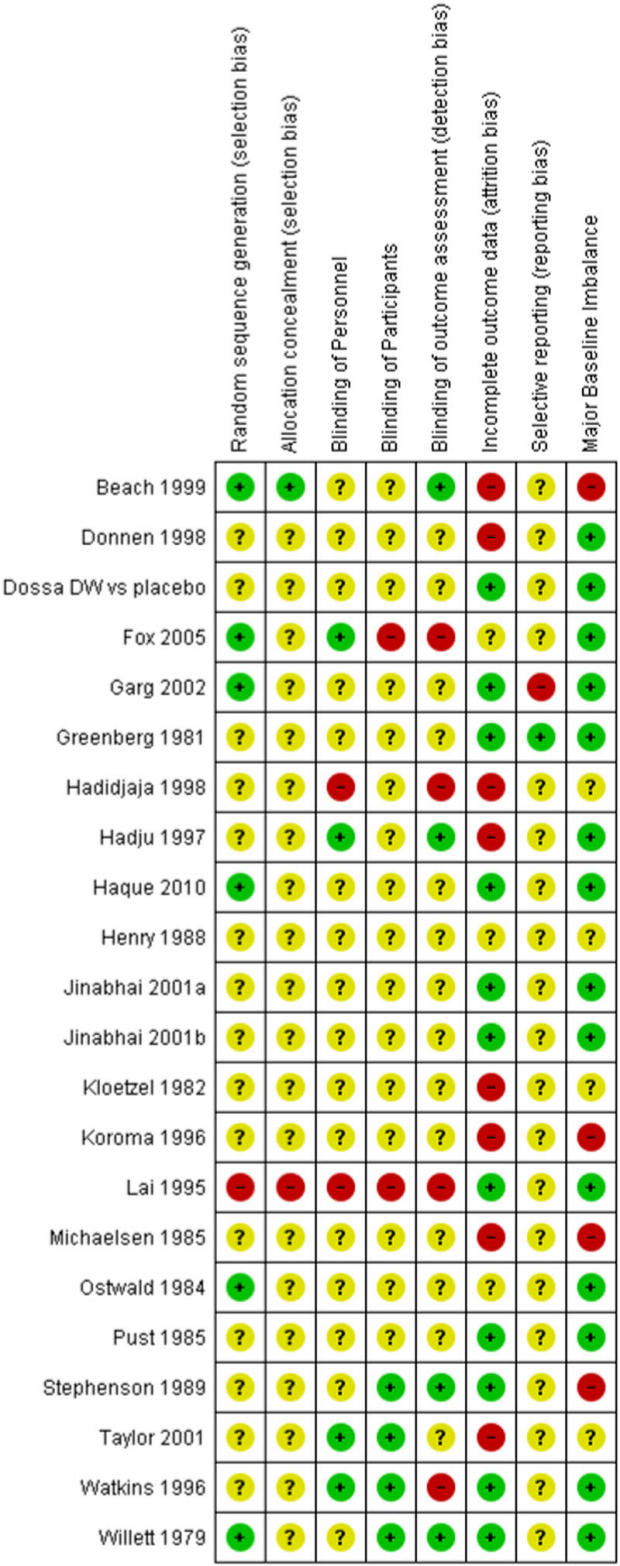
Risk of bias for studies not providing data

The overall risk of bias was similar for studies for which we were unable to obtain data except for selection bias which was low risk in only 4.5% (1 of 22 studies) and unclear in 91% (22 studies) and blinding of personnel which was low risk in 18% (four studies) and unclear in 72% (16 studies) due to lack of description of the method of allocation or blinding (Figures 10 and 11).

### 5.6 Preparation, replication, imputation, measurement and estimation

As described in the methods, we followed four steps to prepare, replicate, impute and calculate anthropometric *Z* scores.

#### 5.6.1 Preparation: missingness analysis

Of the 19 studies that met this review's inclusion criteria, 14 studies were missing <50% of data for outcomes and covariates at baseline and endline, and were included in the main analysis (Table 4). For the studies included in the main analysis, there was an average of 4% missing data at baseline (range, 0–42%), and an average of 9% missing data at endline (range, 0–31%). Five studies (Hall et al., 2006; Kirwan et al., 2009; Miguel & Kremer, 2004; Rousham & Mascie‐Taylor, 1994; Wiria et al., 2013) were missing more than 50% of outcome or covariate data at baseline or endline, and were included in the complete case analysis only. Wiria et al. (2013), Hall et al. (2006), and Miguel and Kremer (2004) were missing more than 50% of data for all STH counts at baseline. Hall et al. (2006) and Rousham and Mascie‐Taylor (1994) did not collect haemoglobin at baseline nor endline. Wiria et al. (2013) and Kirwan et al. (2009) were missing more than 50% of data on all outcome variables at endline. Miguel and Kremer (2004) was missing height measures for all study participants at baseline, and haemoglobin measures for all participants at endline.

**Table 4:** Number and percentage of missing values at baseline and endline of outcome variables and covariates by eligible study

		Baseline								Endline		
Studies	*N*	Age (%)	Sex (%)	*Ascaris lumbricoides* epg (%)	*Trichuris trichiura* epg (%)	Hookworm epg (%)	Hb (%)	Weight (%)	Height (%)	Hb (%)	Weight (%)	Height (%)
Ebenezer (2013)	1,579	48 (3.0)	26 (1.6)	114 (7.2)	114 (7.2)	114 (7.2)	50 (3.2)	9 (0.6)	9 (0.6)	207 (13.1)	219 (13.9)	205 (13.0)
Friis (2003)	915	0	0	0	0	0	212 (23.2)	134 (14.6)	133 (14.5)	282 (30.8)	224 (24.5)	222 (24.3)
Huong (2007)	426	0	8 (0.2)	0	0	0	0	0	0	16 (3.8)	16 (3.8)	16 (3.8)
Liu et al. (2017)	2,180	1 (0.0)	1 (0.0)	610 (28.0)	573 (26.3)	905 (41.5)	1 (0.0)	2 (0.0)	2 (0.0)	139 (6.4)	134 (6.1)	134 (6.1)
Nga (2009)	510	0	0	0	0	0	0	0	0	38 (7.5)	28 (5.5)	28 (5.5)
Olds (1999)	371	0	0	0	0	0	0	0	0	44 (11.9)	44 (11.9)	44 (11.9)
Rohner (2010)	635	130 (20.5)	46 (7.2)	45 (7.1)	45 (7.1)	45 (7.1)	44 (6.9)	24 (3.8)	27 (4.3)	81 (12.8)	84 (13.2)	2 (0.3)
Solon (2003)	831	0	0	159 (19.1)	159 (19.1)	159 (19.1)	28 (3.4)	0	0	28 (3.4)	0	0
Yap (2014)	194	0	0	0	0	0	0	0	0	0	0	0
Beasley et al. (1999)	357	0	0	0	0	0	0	1 (0.0)	1 (0.0)	56 (15.7)	56 (15.7)	56 (15.7)
Beasley Tanbase	217	0	0	0	0	0	9 (4.1)	5 (2.3)	5 (2.3)	24 (11.1)	24 (11.1)	24 (11.1)
Stoltzfus (1997)	4,034	440 (11.0)	192 (4.8)	598 (14.8)	598 (14.8)	597 (14.8)	431 (10.7)	432 (10.7)	431 (10.7)	719 (17.8)	722 (17.9)	723 (17.9)
Stoltzfus (2004)	463	0	0	15 (3.2)	15 (3.2)	15 (3.2)	0	0	0	35 (7.6)	35 (7.6)	35 (7.6)
Ndibazza et al. (2012)	2,016	341 (16.9)	0	534 (26.5)	534 (26.5)	534 (26.5)	388 (19.2)	343 (17.0)	359 (17.8)	449 (22.3)	434 (21.5)	448 (22.2)
Wiria (2013)	1,854	0	0	1,277 (68.9)	1,041 (56.1)	1,277 (68.9)	1,629 (87.9)	1,116 (60.2)	1,116 (60.2)	1,425 (76.9)	1,186 (64.0)	1,186 (64.0)
Kirwan (2009)	1,367	0	14 (0.01)	102 (0.07)	101 (0.07)	100 (0.07)	57 (0.04)	9 (0.01)	12 (0.01)	953 (69.7)	873 (63.9)	883 (64.6)
Hall (2006)	2,916	256 (0.09)	0	2,295 (78.7)	2,295 (78.7)	2,295 (78.7)	2,916 (100.0)	256 (0.09)	256 (0.09)	2,916 (100.0)	256 (0.09)	256 (0.09)
Miguel (2004)	15,901	2,820 (17.7)	2,771 (17.4)	14,007 (88.1)	14,007 (88.1)	14,007 (88.1)	15,015 (94.4)	2,774 (17.4)	15,901 (100.0)	15,901 (100.0)	6,541 (41.1)	6,542 (41.1)
Rousham (1994)	124	1 (0.01)	1 (0.01)	29 (23.4)	29 (23.4)	29 (23.4)	124 (100.0)	2 (0.02)	3 (0.02)	124 (100.0)	6 (0.05)	6 (0.05)

#### 5.6.2 Replication

Replication of the published study results was conducted for all 19 eligible studies. The standardised differences between the published and replication results were at or below 0.10 for all outcome measures and covariates at baseline and endline, with the exception of two measures from the Ebenezer et al. (2013) study (baseline haemoglobin and baseline age) (Table 5). The average standardised difference between published study results and replication results at baseline and endline were 0.014 (range, −0.13–0.15) and 0.015 (range, −0.09–0.07), respectively. For every study, there was at least one instance where the standardised difference could not be calculated at baseline or endline because the published results did not report the covariate or outcome measure in question (indicated as “NA” in Table 6).

**Table 5:** Standardised differences between published and reproduced results for baseline outcome measures and covariates by eligible study

Studies	Hb	Weight	Height	Age	Sex	*Ascaris lumbricoides* epg	Hookworm epg	*Trichuris trichiura* epg
Beasley et al. (1999)	0.00	NA	NA	NA	NA	0.00	0.00	0.00
Beasley Tanbase	NA	NA	NA	NA	NA	NA	NA	NA
Ebenezer (2013)^a^	0.00, **0.15**	NA	NA	**−0.13**, 0.00	NA	NA	NA	NA
Friis (2003)	−0.06, 0.007	−0.02, 0.025	−0.05, 0.04	0.00, 0.004	NA	NA	NA	NA
Huong (2007)	−0.08, 0.02	−0.10, 0.04	−0.01, 0.00	−0.02, 0.04	NA	NA	NA	NA
Liu et al. (2017)	0.00	0.00	0.00	0.00	0.00	0.00	0.00	0.00
Ndibazza et al. (2012)	NA	NA	NA	0.00	0.00	NA	NA	NA
Nga (2009)	0.00, 0.01	−0.01, 0.00	−0.01, 0.01	0.00	NA	NA	NA	NA
Olds (1999)	NA	NA	NA	NA	NA	NA	NA	NA
Rohner (2010)	−0.02, 0.01	NA	NA	−0.06, 0.00	NA	NA	NA	NA
Solon (2003)	−0.01, 0.00	0.00	0.00	−0.02, 0.02	NA	NA	NA	NA
Stoltzfus (1997)	NA	0.00	0.00	0.00	0.00	0.00	0.00	0.00
Stoltzfus (2004)	−0.08, 0.00	NA	NA	−0.09, 0.03	NA	NA	NA	NA
Yap (2014)	−0.01, 0.02	0.00	−0.01, 0.00	−0.02, 0.03	NA	0.00	0.00	0.00
Kirwan (2009)	NA	NA	NA	0.00	0.00	0.00	0.00	0.00
Hall (2006)	NA	0.00	0.00	0.00	NA	0.00	0.00	0.00
Miguel (2004)	NA	NA	NA	NA	NA	0.00	0.00	0.00
Rousham (1994)	NA	0.00	0.00	NA	NA	NA	NA	NA
Wiria (2013)	NA	0.00	0.00	NA	NA	NA	NA	NA

Abbreviation: epg, eggs per gram of stool.

^a^ Standardised differences between published and reproduced results > 0.10.

**Table 6:** Standardised differences between published and reproduced results for endline outcome measures by eligible study

Studies	Hb	Weight	Height
Beasley et al. (1999)	−0.09, 0	NA	NA
Beasley (1995)	NA	NA	NA
Ebenezer (2013)	0.00	NA	NA
Friis (2003)	−0.006, 0.069	NA	NA
Huong (2007)	0.00	NA	NA
Liu et al. (2017)	0.00	0.00	0.00
Ndibazza et al. (2012)	−0.02, 0.00	0.00	−0.02
Nga (2009)	0.00	NA	NA
Olds (1999)	NA	NA	NA
Rohner (2010)	−0.002, 0.01	NA	NA
Solon (2003)	−0.049, 0.008	−0.058, −0.047	−0.06, −0.03
Stoltzfus (1997)	0.00	0.00	0.00
Stoltzfus (2004)	0.00	NA	NA
Yap (2014)	0.00, 0.002	−0.003, 0.007	−0.007, −0.005
Kirwan (2009)	NA	NA	NA
Hall (2006)	NA	NA	NA
Miguel (2004)	NA	NA	NA
Rousham (1994)	NA	NA	NA
Wiria (2013)	NA	0.00	0.00

#### 5.6.3 Imputation

We used multiple imputation for missing data at baseline and endline and created five completed datasets.

#### 5.6.4 Measurement and estimation

Two studies (Ebenezer et al., 2013 and Yap et al., 2014) required adjustments to haemoglobin measures due to high altitude. The altitude correction method applied was:

$${\text{Hb}}_{\text{sea}‐\text{level}}={\text{Hb}}_{\text{measured}}-3.44\times (e^{\left(0.\;000\;633\times \text{Alt}\right)}-1),$$

where Hb_sea level_ stands for the concentration after adjustment, and Alt for altitude (m) (Dirren, Logman, Barclay, & Freire, 1994). The mean altitude for each village was applied to each child in the village.

BMI for age, weight for age and height for age were calculated as described above, using Anthro Software.

Distribution curves for effect modifier variables were prepared to confirm there were sufficient numbers of children in each prespecified level to conduct subgroup analyses (Supporting Information Figures). For individual‐level intensity of infection (any helminth, *Ascaris*, hookworm, *Trichuris*), the distribution curves showed that there were insufficient numbers of children with a high intensity level of any helminth infection to justify the use of the WHO cutoffs for each helminth, as originally planned. Consequently, tertiles for the distributions were calculated and used to define the levels of the subgroup analyses to assess the gradient of effect for each helminth and for infection intensity (any helminth). The cutoffs used for BMI‐for‐age *z* score, weight‐for‐age *z* score, and height‐for‐age *z* scores were adjusted to include only two levels (≤−2*SD*, >−2*SD*) to accommodate the lack of children with extreme scores at either end of the distribution. Anaemia status was adjusted to two levels (not anaemic, anaemic)^1^ ^1^ for the same reason.

### 5.7 Effect of deworming on infection intensity

The effect of deworming on infection intensity was assessed for each study and each type of STH infection and found to be variable across studies (see Appendix 4, Forest plots, comparisons 9 and 10). When sorted according to year of publication, there was no visual trend of greater infection prevalence between the years of publication of these studies from 1999 to 2015. The complete case analysis (only including children with complete data on infection prevalence) were comparable to results conducted using multiple imputation for baseline prevalence.

Six studies had relative risk reduction of *A. lumbricoides* infection prevalence 20% or more when compared to the placebo group in *A. lumbricoides* prevalence at endline (Beasley et al., 1999; Friis et al., 2003; Le Huong et al., 2007; Nga et al., 2009; Stoltzfus et al., 1997, 2004) and these were included in a sensitivity analysis to assess whether greater impact on *A. lumbricoides* prevalence was associated with greater effects on growth or haemoglobin.

One study did not assess endline infection intensity (Liu et al., 2017).

### 5.8 NMA‐IPD model development

We planned our analysis model *a priori* based on consultation with the advisory group and our research team to consider study design elements, outcomes, covariates and effect modifiers.

#### 5.8.1 Changes to analysis model

The effect on plasma ferritin levels was not assessed because only seven studies measured this outcome (Beasley et al., 1999; Beasley, 1995; Le Huong et al., 2007; Nga et al., 2009; Rohner et al., 2010; Stoltzfus et al., 1997, 2004) (Table 7).

**Table 7:** Comparison of original analysis plan and actual model employed

	Planned	Actual
Design	Study Clusters Treatment arms Number of 6‐month periods	Study Clusters Treatment arms
Outcomes	Change in weight (kg) Change in height (cm) Change in haemoglobin (g/L) Change in cognition Change in plasma ferritin	Change in weight (kg) Change in height (cm) Change in haemoglobin (g/L) Change in cognition (by study only)
Covariates	Age Sex *Ascaris lumbricoides* epg count Hookworm epg count *Trichuris trichiura* epg count Haemoglobin Height‐for‐age BMI‐for‐age (5 years and older) Weight‐for‐height (under 5 years) Socioeconomic status Maternal education	Age Sex *A. lumbricoides* epg count Hookworm epg count *T. trichiura* epg count Haemoglobin Height‐for‐age BMI‐for‐age (all ages)
Effect modifiers	Weight‐for‐age *z* score (<−3*SD*, −3*SD*, −2*SD*, >−2*SD*) Height‐for‐age *z* score (<−3*SD*, −3*SD*, −2*SD*, >−2*SD*) *A. lumbricoides* intensity (0, light, moderate, heavy) Hookworm intensity (0, light, moderate, heavy) *T. trichiura* intensity (0, light, moderate, heavy) Any helminth infection intensity (0, light, moderate, heavy) Anaemia status (none, mild, moderate, severe) Age (1–5, >5 years) Sex (female, male)	BMI‐for‐age *z* score (≤−2*SD*, >−2*SD*) Height‐for‐age *z* score (≤−2*SD*, >−2*SD*) *A. lumbricoides* intensity (tertiles) Hookworm intensity (tertiles) *T. trichiura* intensity (tertiles) Any helminth infection intensity (tertiles) Anaemia status (not anaemic, anaemic) Age (1–5, >5 years) Sex (female, male)

Abbreviation: BMI, body mass index; epg, eggs per gram of stool.

Six out of 14 studies (Ebenezer et al., 2013; Liu et al., 2017; Nga et al., 2009; Rohner et al., 2010; Solon et al., 2003; Stoltzfus et al., 1997) measured effects on cognition outcomes. However, the specific measures and methods used to assess cognition varied by study. At the December 2017 meeting of the review investigators and advisors (London, UK), it was decided to assess cognition (using measures for attention and development) on a study‐by‐study basis. Where measures were described with the same name (e.g., working memory in Liu et al., 2017; Ndibazza et al., 2012; Nga et al., 2009), the Advisory Group recommended not combining results across studies since the translation and different contexts of the studies could influence the tool's application. Cognitive measures were categorised as related to: (a) short‐term attention (e.g., digit recall), (b) scholastic performance (e.g., math, language tests) or (c) developmental outcomes (e.g. motor development, Raven's index).

An insufficient number of eligible studies included measures for maternal education and socioeconomic status, and the specific measures used varied by study (Table 8). Five studies (Ebenezer et al., 2013; Liu et al., 2017; Ndibazza et al., 2012; Nga et al., 2009; Yap et al., 2014) included a measure for maternal education, and seven studies (Beasley et al., 1999; Beasley, 1995; Liu et al., 2017; Ebenezer et al., 2013; Ndibazza et al., 2012; Nga et al., 2009; Yap et al., 2014) included a measure for socioeconomic status. Given the limited number of studies and the variability in measures, these measures were not included as covariates in the model.

**Table 8:** Maternal education, socioeconomic status, and cognition measures by eligible study

Studies	Maternal education	Socioeconomic status	Cognition
Beasley (1999)	NA	House made of concrete (yes, no) Owns home (yes, no) Owns a sewing machine (yes, no) Owns a radio (yes, no)	NA
Beasley (1995)	NA	House made of concrete (yes, no) Flushing toilet (yes, no) Owns home (yes, no) Owns a sewing machine (yes, no) Owns a bike (yes, no) Owns a radio (yes, no)	NA
Liu et al. (2017)	Mother has attended secondary school (yes, no)	Individual level Boarding at school Ethnicity Household level Number of siblings Number of pieces of durable assets Parents who are working migrants (yes, no) Mother has attended secondary school (yes, no) Father has attended secondary school (yes, no) Dirt floor (yes, no) Dirt‐based latrines (yes, no)	Working memory index (digit span, letter numbering sequencing) Processing speed index (coding, symbol search)
Ebenezer (2013)	Total number of years in school	Poor (yes, no) Father's total number of years in school	Math scores Tamil scores Single digit attention scores Double digit attention scores
Ndibazza et al. (2012)	Level of education (none, primary, secondary, tertiary)	Household SES scored (1–6) based on building materials of the home, number of rooms, and items owned	General cognitive abilities (block design, picture vocabulary scale) Cognitive flexibility (Wisconsin card sort test) Measure of attention (picture search) Working memory (sentence repetition, verbal fluency, counting span, running memory) Motor abilities (coin box, balancing on one leg) Measure of planning (Tower of London) Measures of inhibition (tap once tap twice; shapes task)
Nga (2009)	Level of education (illiterate, primary, secondary, high school, college)	Percentage of households classified as “poor”	Raven's coloured progressive matrices test Working memory (digit span forward, digit span backward) Processing speed index (coding, block design) Math scores Vietnamese language scores
Rohner	NA	NA	Raven score Coding total Symbols total Target marking errors Target marking time
Solon (2003)	NA	NA	Verbal ability Quantitative ability Nonverbal ability Total cognition
Stoltzfus (2004)	NA	NA	Language development (18 items) Motor development (20 items)
Yap (2014)	Parents' level of education (literate, primary, secondary, above)	Parents' level of education (literate, primary, secondary, above) Household income source (agriculture, teacher/government official, own business, worker)	NA

Abbreviation: SES, socioeconomic status.

The weight‐for‐height *z* score for children under 5 years that was originally planned as a covariate was replaced by BMI‐for‐age on the recommendation of the Advisory Group to avoid collinearity between weight‐for‐age and height‐for‐age *z* scores.

Indicators for water and sanitation were not included as effect modifiers because not all studies described water and sanitation conditions (see Table 2—Characteristics of Included Studies). The studies that did provide descriptions did not do so in a quantifiable way that would allow comparison.

#### 5.8.2 Evidence network and feasibility assessment for NMA

**Figure 12:**
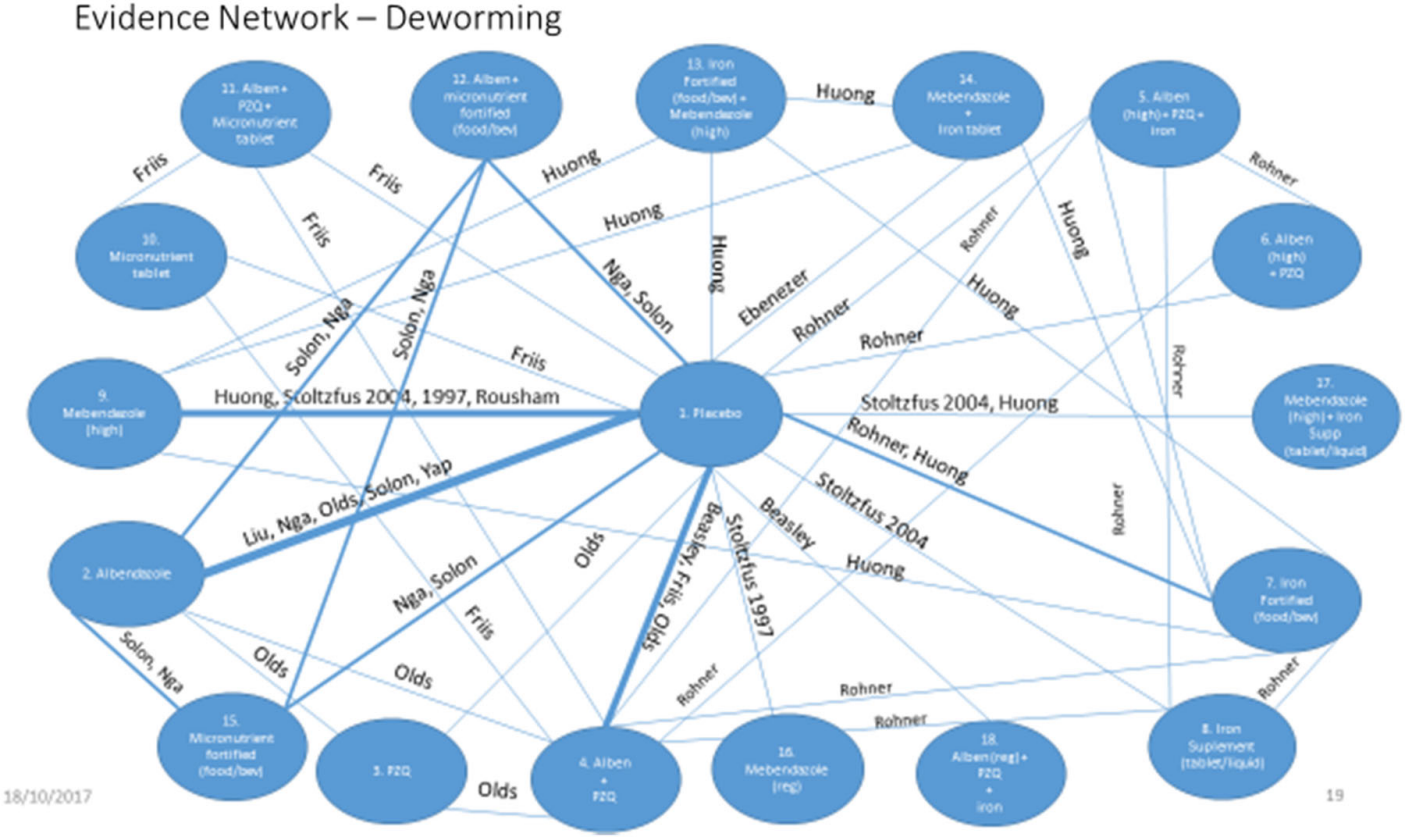
Full evidence network

The full evidence network included 18 nodes (Figure 12) due to different types of deworming (e.g., albendazole, mebendazole and praziquantel), cointerventions (e.g., micronutrients) and frequency of deworming. We considered the control arm of two studies as equivalent to placebo (Liu and Miguel).

### 5.9 Evidence network refinement

We ran the NMA‐IPD for the full network with 18 nodes, as above. We excluded five studies with >50% missing data since we could not impute missing data and adjusted analyses would be biased due to the amount of missing data. We decided to include these five studies in a “complete case” sensitivity analysis.

The results with the full evidence network were presented at a meeting of the Advisory Group in June 2017 (Table S13: full network—14 studies, 18 nodes). The Advisory Board decided that the full network was too complicated to allow meaningful interpretation for policy decisions.

The effects on weight gain, height gain and haemoglobin were not statistically significant for the two types of STH deworming drugs: albendazole and mebendazole. Since the WHO guidelines on deworming (WHO, 2017) do not distinguish between the choice of mebendazole or albendazole on the grounds of nutritional effects, the Advisory board decided to collapse across type of STH drug.

With respect to praziquantel, results showed larger effects on haemoglobin for all treatment comparisons which included praziquantel (e.g., Albendazole + praziquantel effect on haemoglobin was 2.03 g/L, 95% CI: 0.56, 3.50) compared to effect of albendazole alone on haemoglobin of 0.16 g/L, 95% CI: −0.86, 1.17). Praziquantel is given in areas where schistosomiasis is endemic, and schistosomiasis is known to have effects of chronic inflammation in the intestines, liver or urogeneital system which are linked to anaemia and malnutrition (Colley, Bustinduy, Secor, & King, 2014). Thus, the Advisory board decided to collapse all treatments which included any type of praziquantel into one treatment node.

With respect to micronutrients, in the analysis of 18 nodes, we found overlapping 95% CIs for iron fortified food or beverages (−0.04 kg, 95% CI: −0.35, 0.27), iron tablets (−0.32 kg, 95% CI: −0.69, 0.06), micronutrient tablets (0.03 kg, 95% CI: −0.26, 0.32) or micronutrient fortified food/beverage (0.04 kg, 95% CI: −0.17, 0.24) on weight gain. Also, there were no differences in haemoglobin effects (Table S13). Thus, the Advisory board decided to collapse all iron and micronutrients into a single node.

Based on evidence of efficacy of micronutrients and iron on childhood anaemia and iron deficiency (De‐Regil, Jefferds, Sylvetsky, & Dowswell, 2011), the Advisory Board agreed that all micronutrients and iron could be collapsed together into one node. Similarly STH deworming combined with any micronutrient or iron was collapsed into a treatment node.

This revised, collapsed network was run, with separate nodes for frequency of STH deworming to further explore the importance of frequency in a network with eight nodes. The Advisory Group recommended combining all helminth deworming drugs into one node for high frequency dosage, and a second node for regular frequency dosage. The rationale for keeping frequency separate was that more frequent deworming could provide more constant levels of lower worm burden.

**Figure 13:**
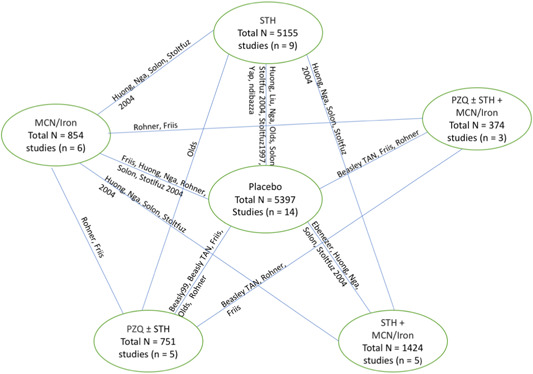
Final collapsed evidence network. *Five studies with >50% missing data are not shown in this figure. Four of these included STH deworming versus placebo (Wiria, Kirwan, Miguel, Rousham) and one assessed STH deworming + micronutrients versus micronutrients (Hall et al., 2006). STH, soil‐transmitted helminthiasis

At the November 2017 meeting of investigators and the Advisory Group, the results for high versus regular frequency of STH deworming were presented and considered not different (e.g weight gain for STH deworming at high frequency was −0.01 kg, 95% CI: −0.13, 0.10) and for regular frequency weight gain was 0.13 kg (95% CI: 0.04, 0.22)) hence the network was further reduced to six nodes (Figure 13).

Since the results may be influenced by these decisions about collapsing across nodes, the Advisory Board and research team decided to also analyse the full network as a sensitivity analysis.

#### 5.9.1 Assessing feasibility of NMA with IPD

##### Assumptions of transitivity and consistency

Transitivity was considered plausible because we assessed the distribution of child‐level effect modifiers across studies, and found similar distributions across studies for all covariates (Figures 2–13). In addition, we found that the distribution of effect modifiers was balanced across comparisons (Table S17). As shown by the evidence network, the treatments are given for the same indication and compared in the same studies, with numerous connected nodes in the full network (with 18 nodes) as well as the collapsed evidence network (six nodes; Table 9).

**Table 9:** Comparison of node constitution in full network, June 2017 collapsed network and November 2017 network model

	Full network	Collapsed network1 (8 nodes) Jun 2017 Advisory Group meeting	Collapsed network2 (6 nodes) November 2017 Advisory Group meeting
1	Placebo or control	Placebo or control	Placebo or control
2	Albendazole	STH deworming with any drug at regular frequency	STH deworming with any drug
3	Praziquantel	STH deworming with any drug at high frequency	Any STH deworming combination with praziquantel
4	Albendazole with praziquantel	Any STH deworming combination at regular frequency with praziquantel with or without iron or micronutrients	Any STH deworming combination with praziquantel with iron or micronutrients
5	Albendazole (high) with praziquantel with iron	Any STH deworming combination at high frequency with praziquantel with or without iron or micronutrients	Any STH deworming with micronutrients or iron
6	Albendazole (high) with praziquantel	Any STH deworming at regular frequency with micronutrients or iron	Micronutrients or iron alone
7	Iron fortified (food/beverage)	Any STH deworming at high frequency with micronutrients or iron	
8	Iron supplement (tablet/liquid)	Micronutrients or iron alone	
9	Mebendazole (high)		
10	Micronutrient tablet		
11	Albendazole with praziquantel with micronutrient tablet		
12	Albendazole with micronutrient fortified (food/beverage)		
13	Mebendazole (high) with iron fortified (food/beverage)		
14	Mebendazole with iron tablet		
15	Micronutrient fortified (food/beverage)		
16	Mebendazole (regular)		
17	Mebendazole (high) with iron supplement (tablet/liquid)		
18	Albendazole (regular) with praziquantel with iron		

Abbreviation: STH, soil‐transmitted helminthiasis.

Methodological and clinical heterogeneity was considered appropriate for pooling by considering the settings, population characteristics and interventions. Statistical heterogeneity within each treatment comparison was tested by constructing forest plots for each direct comparison. Heterogeneity as measured by the *I* ^2^ statistic was <75% for all direct comparisons (Appendix 3, Forest plots).

### 5.10 Funnel plot

As above, the only comparison with >10 studies providing IPD was STH deworming versus placebo, with nine studies with sufficient data for multiple imputation of missing data, and four studies with >50% missing data (Kirwan et al., 2009; Miguel & Kremer, 2004; Rousham & Mascie‐Taylor, 1994; Wiria et al., 2013).

As planned, we constructed a funnel plot to assess the presence of publication bias. To do this, we included all studies of STH versus placebo from our previous Campbell review of deworming (Welch et al., 2016) to compare the received data with the data which was either not received or ineligible (due to lack of baseline infection intensity data).

**Figure 14:**
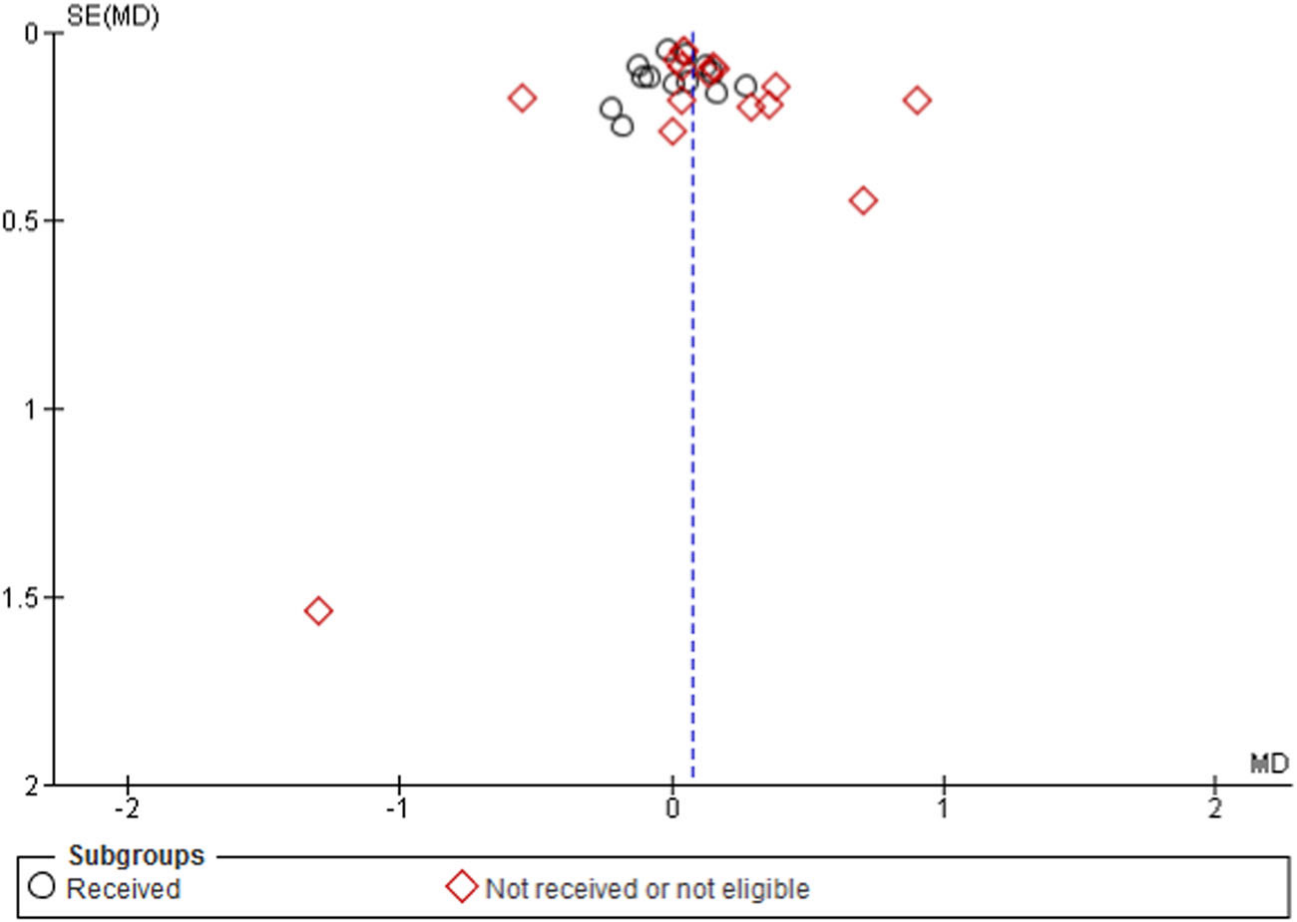
Funnel plot for weight gain (kg) for all studies of STH deworming versus placebo. STH, soil‐transmitted helminthiasis

The funnel plot of STH deworming versus placebo for the studies for which we received data (circles) shows that the studies we received include both positive and negative studies (Figure 14). The studies which were not received had larger effects on weight gain and were smaller (diamonds).

The Egger test for publication bias on the aggregate data of the entire sample (*n* = 30 studies) was not statistically significant (*p* = .249) for small study effects.

### 5.11 Main effects

This section provides the overall results on our four primary outcomes: weight, height, haemoglobin and cognition, using the collapsed evidence network, which we decided was the most clinically sensible and policy‐relevant. The results are based on studies with a median duration of 7 months (ranging from 4 to 45 months).

These findings are summarised in three summary of findings tables.

Following this section, we describe effect modifier analyses for each planned effect modifier for each outcome of interest.

A road map of all analyses is described in Table S10. Results for main effects of NMA with IPD for the base case are in Table S11.

#### 5.11.1 Weight

##### Base case IPD‐NMA analysis

**Figure 15:**
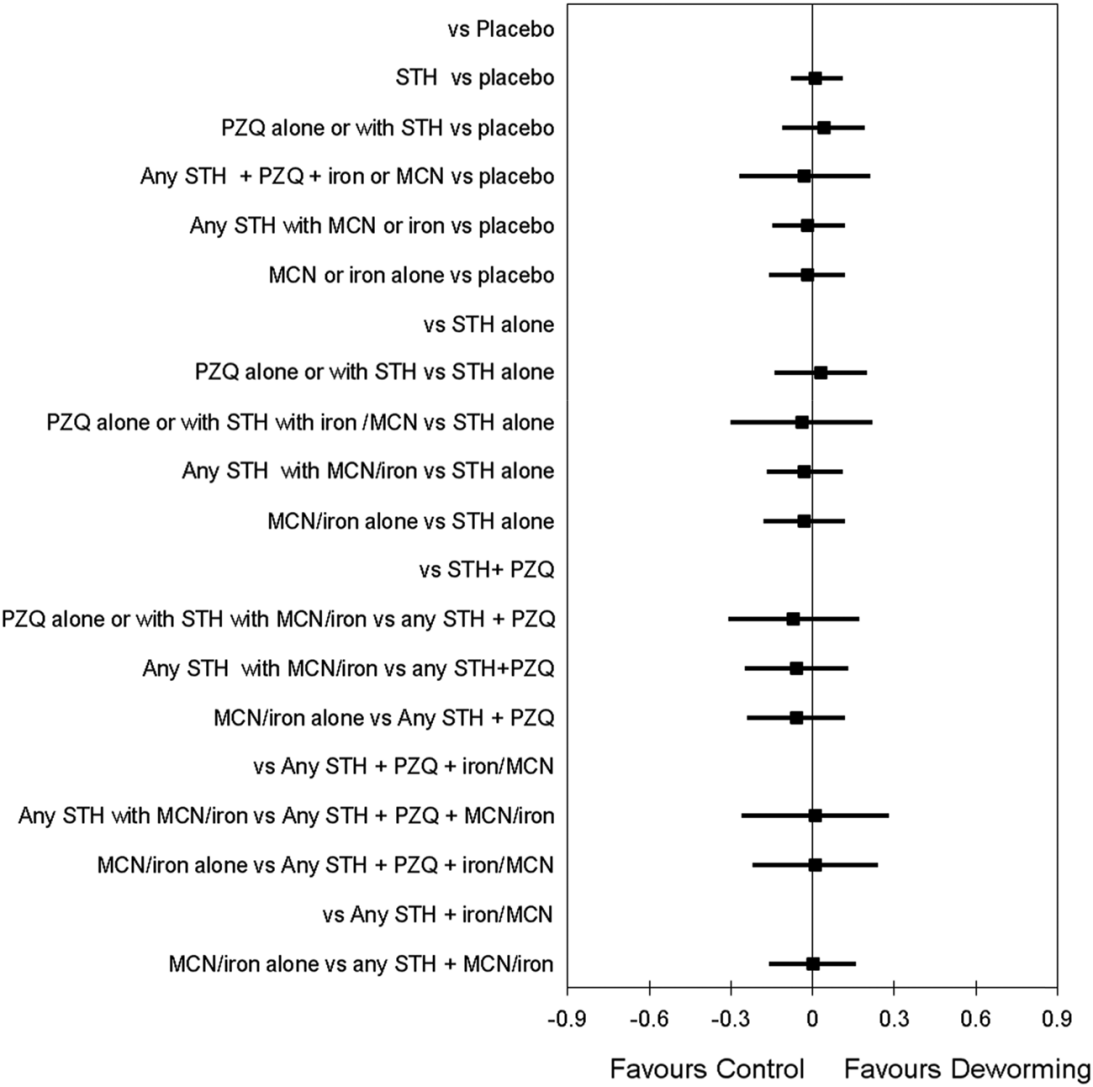
Weight gain (kg), base case, collapsed network, adjusted for covariates

There were no statistically significant effects on weight gain (kg) for any of the deworming combinations compared to placebo. For STH deworming versus placebo, the effect on weight gain was 0.01 kg (95% CI: −0.08, 0.11; Figure 15).

The head‐to‐head comparisons of deworming treatment combinations produced results that were consistent in direction and size with the results of the treatment versus placebo comparisons.

##### Direct evidence‐aggregate and IPD

For each comparison, we compared the IPD‐NMA result with the results for the direct evidence from study results pooled at the aggregate level (adjusted for covariates) and the direct evidence pooled using IPD (adjusted for covariates).

**Figure 16:**
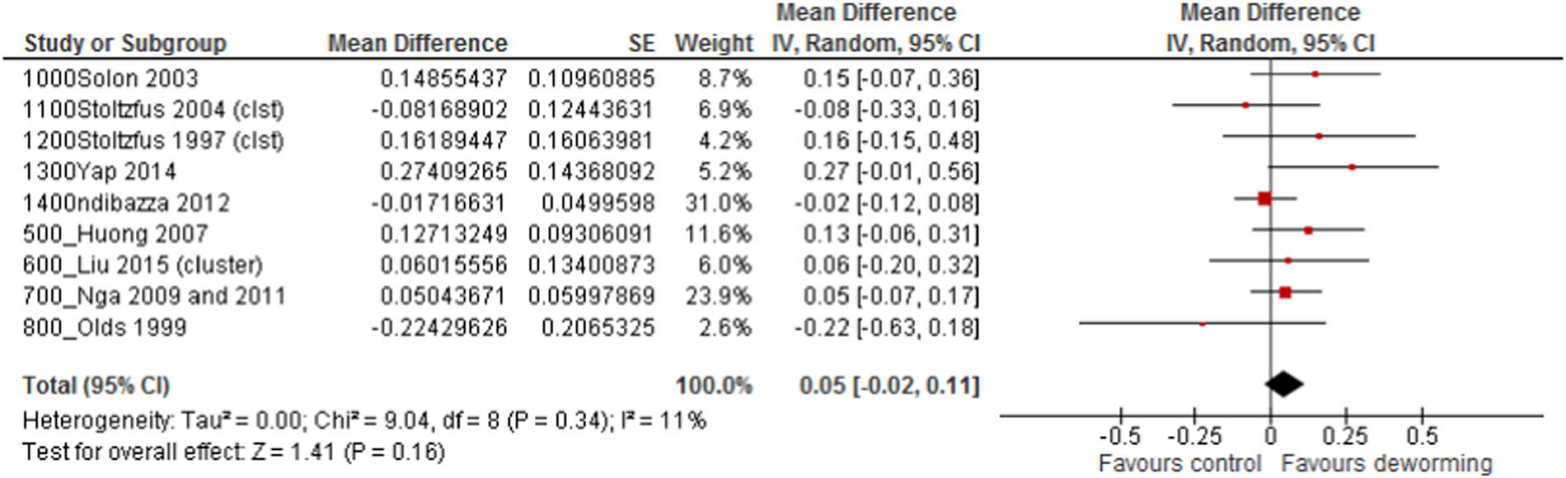
Weight gain (kg), aggregate level direct evidence for STH versus placebo. STH, soil‐transmitted helminthiasis

In all cases, the effect estimates from direct evidence were of similar size and direction as the IPD‐NMA indirect + direct effect estimates (Table 10), and the heterogeneity of direct comparisons was below an *I* ^2^ of 75% (Table 11). The forest plot for one comparison (deworming for STH vs. placebo) is shown in Figure 16.

**Table 10:** Comparison of direct and indirect evidence for weight gain (kg) for STH versus placebo

Analysis	Effect estimate of STH vs. placebo, weight gain (kg)
Aggregate‐direct adjusted	0.05 (−0.02, 0.11), *I* ^2^ = 11%
IPD‐direct adjusted	0.013 (−0.088, 0.115)
IPD‐NMA‐Direct + indirect evidence (adjusted)	0.01 (−0.08,0.11)

Abbreviation: IPD, individual participant data; STH, soil‐transmitted helminthiasis.

**Table 11:** Heterogeneity of direct evidence comparisons

Comparisons	Effect estimate and heterogeneity of direct evidence (pooled at aggregate level)
STH deworming vs. placebo	0.05 (−0.02, 0.11), *I* ^2^ = 11%, 9 studies
PZQ alone or with STH vs. placebo	0.04 (−0.12, 0.20) *I* ^2^ = 0%, 5 studies
PZQ with MCN/iron vs. placebo	0.13 (−0.28, 0.54), *I* ^2^ = 67%, 3 studies
STH deworming with MCN/iron vs. placebo	0.00 (−0.07, 0.08), *I* ^2^ = 0%, 5 studies
MCN/iron vs. placebo	0.01 (−0.07, 0.09), *I* ^2^ = 6%, 6 studies

Abbreviations: MCN, micronutrients; PZQ, praziquantel; STH, soil‐transmitted helminthiasis.

The effect estimates for all other comparisons are shown in Appendix 3 with details for each study for each comparison (Appendix 3).

##### Sensitivity analyses

There were no qualitative (different directions of effects) nor quantitative (different sizes of effects) differences in the analyses conducted with no covariates (unadjusted analyses; Table S12). For example, the unadjusted effect on weight gain was 0.01 (−0.08, 0.11) for STH versus placebo.

The results of a complete case analysis with the same 14 studies from the base case, where missing data were not imputed, was congruent with the main effects described above (Table S12). For example, the effect on weight gain for STH deworming versus placebo was 0.03 kg (95% CI: −0.07, 0.13).

The complete case analysis with an additional five studies (Hall et al., 2006; Kirwan et al., 2009; Miguel & Kremer, 2004; Rousham & Mascie‐Taylor, 1994; Wiria et al., 2013) which had too much missing data (>50%) for multiple imputation was congruent with our base case analysis. For example, the effect on weight for STH deworming versus placebo was 0.01 kg (95% CI: −0.11, 0.12).

Analysis of the NMA model restricted to studies at low risk of bias yielded similar results (Table S12) for weight gain (kg) for STH versus placebo (0.01 kg, 95% CI: −0.10, 0.12). However, there were larger effects for praziquantel with or without STH deworming versus placebo (0.17 kg, 95% CI: −0.28, 0.62) compared to 0.04 kg (−0.11 to 0.19) in base case) or praziquantel with STH deworming and micronutrients or iron (0.34 kg, 95% CI: −0.10, 0.78) compared to −0.03 kg, 95% CI: −0.27, 0.21). These latter comparisons also had wider CIs due to smaller numbers of participants.

We assessed the full evidence network with 18 nodes with our 14 base case studies, with multiple imputation for missing data and adjusted for covariates as a sensitivity analysis to allow comparison of separate drugs such as albendazole and mebendazole at different frequencies to our main model findings with the collapsed network. These analyses had wider CIs (Table S12). In this analysis, we found some larger effects on weight gain than in the collapsed model. For example, the effect of mebendazole twice per year versus placebo was 0.25 kg (95% CI: −0.37, 0.86). For praziquantel alone versus placebo, the effect was 0.18 kg (95% CI: −0.19, 0.56) compared to 0.04 kg (−0.11, 0.19) in the base case. None of these effects were statistically significant.

One study had very precise results and received a lot of weight in the meta‐analyses for weight and height gain (Nga et al., 2009). We conducted a sensitivity analysis without this study and found the same effect on weight gain for STH versus placebo (0.01 kg, 95% CI: −0.08, 0.11). Other effect sizes were also of a similar magnitude and direction as the base case.

As described above, there was variation in effect of deworming on infection prevalence at endline. We conducted a senstivity analysis restricted to studies which were more effective at reducing infection prevalence, defined as a a relative risk of 0.80 or lower when compared to the placebo group in *A. lumbricoides* prevalence at endline (Beasley et al., 1999; Friis et al., 2003; Le Huong et al., 2007; Nga et al., 2009; Stoltzfus et al., 1997, 2004). The results of this sensitivity analysis show that for STH deworming versus placebo, the effect on weight gain was 0.08 kg, 95% CI (−0.10, 0.26), whereas our basecase analysis findings were 0.10 kg (95% CI: −0.08, 0.11).

##### Comparison of effect sizes for weight gain (kg) between received data and studies that were not included in the analysis

We assessed whether the effects on weight gain were similar for these studies which did not provide data (either because they did not provide it or because they did not meet eligibility criteria) to the studies which did provide data (Appendix 3). For the STH versus placebo comparison, using aggregate data, the effect size for the studies for which we received data was 0.02 kg (95% CI: −0.04, 0.08) (13 studies, *I* ^2^ 12%) compared to an effect size for the studies we did not receive of 0.13 kg (95% CI: 0.01, 0.25) (*n* = 15 studies, *I* ^2^ 71%). The interaction test for subgroup differences was not statistically significant (*p* = .10). The pooled effect of all 28 studies was 0.07 kg, 95% CI (0.00, 0.13).

The above analysis only includes studies which randomised STH alone compared to a placebo or control arm. Studies with vitamin A, iron or praziquantel as cointerventions are not included in this analysis since we decided that STH deworming and cointerventions should be considered as separate nodes. The latter analysis omits one study (Stephenson et al., 1989) which was also omitted from our previous meta‐analysis (Welch et al. 2016). In our previous systematic review, we identified baseline imbalance in the Stephenson 1989 study for hookworm prevalence (95% vs. 79%) and infection intensity (1,183 epg vs. 394 epg for the control group). This baseline imbalance is larger than expected by chance and may have biased the study to find larger effects since sicker children were in the intervention group. The effect was 1.3 kg greater weight gain with a single dose of albendazole (400 mg) compared to placebo after 6 months. When included in our analysis, the *I* ^2^ was 89%, suggesting pooling is inappropriate. There may have been other factors related to this study which led to a larger weight gain than seen in any of the other 28 studies of STH deworming versus placebo. The Stephenson et al. (1993) study conducted in the same area in Kenya found an effect of 1.1 kg on weight gain of a single dose of Albendazole over 8.2 months.

#### 5.11.2 Height

##### Height‐base case analysis

**Figure 17:**
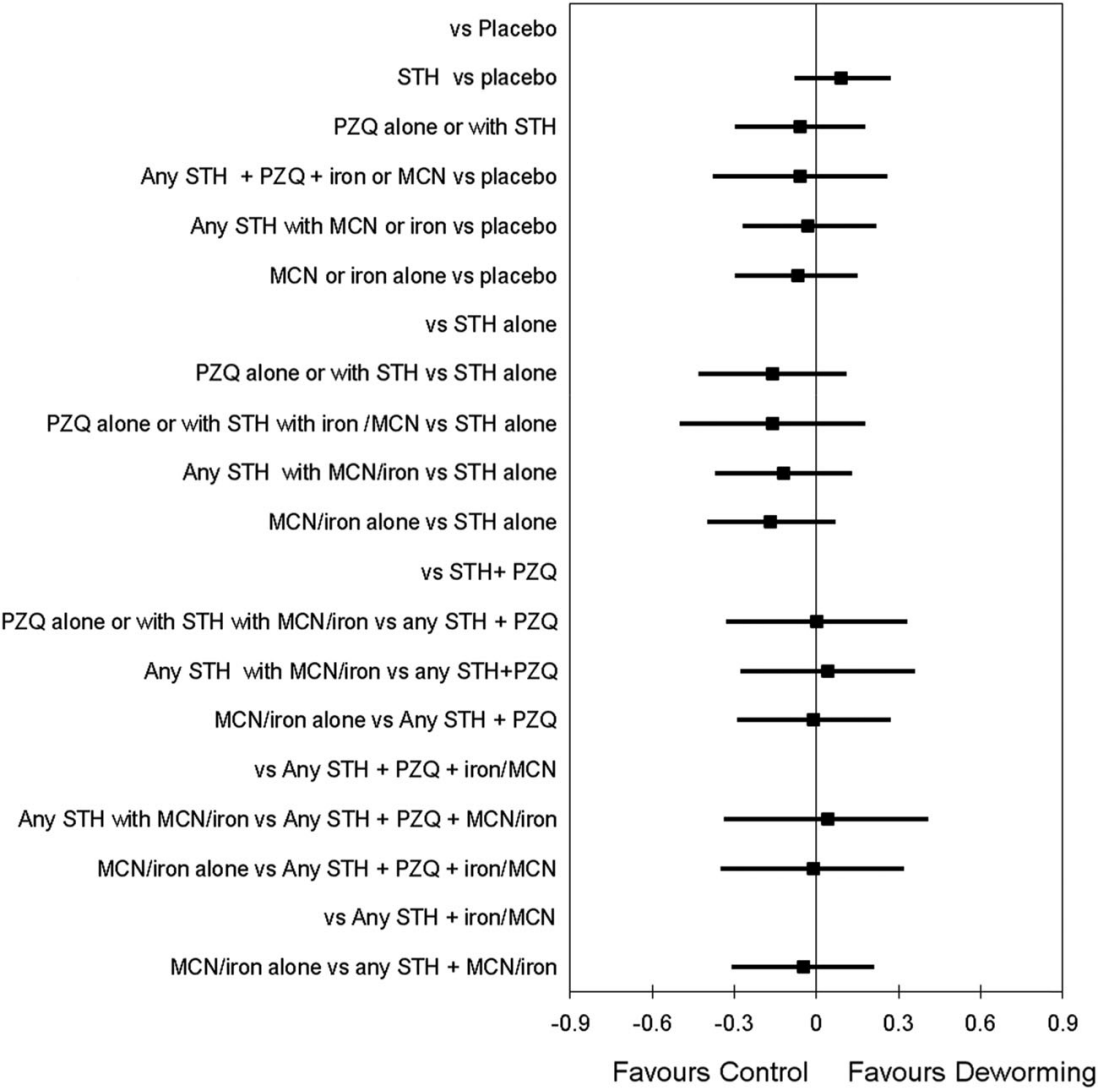
Height gain (cm), base case, collapsed network, adjusted for covariates

The effect on height gain for STH deworming versus placebo was 0.09 cm (95% CI: −0.08, 0.27). The effects for the other comparisons were of similar magnitude (Figure 17).

The head‐to‐head comparisons of STH deworming treatment combinations produced results that were consistent in expected direction and size with the results of the treatment versus placebo comparisons.

##### Direct evidence‐aggregate and IPD

Comparison of the analyses of height gain for STH versus placebo for aggregate data, IPD direct estimates and IPD‐NMA estimates are congruent in size and direction of effect (Table 12).

**Table 12:** Comparison of direct and indirect estimates for STH versus placebo for height gain

Analysis	Effect estimate of STH vs. placebo, height (cm)
Aggregate‐direct adjusted	0.04 (−0.04, 0.11), *I* ^2^ = 11%
IPD‐direct adjusted	0.010 (−0.10, 0.30)
IPD‐NMA‐direct + indirect evidence (adjusted)	0.09 (−0.08, 0.27)

Abbreviation: IPD, individual participant data; NMA, network meta‐analysis; STH, soil‐transmitted helminthiasis.

The forest plots for each direct evidence comparison were of acceptable heterogeneity to carry out NMA (Table 13).

**Table 13:** Direct evidence, assessment of heterogeneity for height gain

Comparisons	Effect estimate and heterogeneity of direct evidence (pooled at aggregate level)
STH deworming vs. placebo	0.05 (−0.02, 0.11), *I* ^2^ = 11%, 9 studies
PZQ alone or with STH vs. placebo	0.04 (−0.12, 0.20) *I* ^2^ = 0%, 5 studies
PZQ with MCN/iron vs. placebo	0.13 (−0.28, 0.54), *I* ^2^ = 67%, 3 studies
STH deworming with MCN/iron vs. placebo	0.00 (−0.07, 0.08), *I* ^2^ = 0%, 5 studies
MCN/iron vs. placebo	0.01 (−0.07, 0.09), *I* ^2^ = 6%, 6 studies

Abbreviation: MCN, micronutrients; PZQ, praziquantel; STH, soil‐transmitted helminthiasis.

##### Sensitivity analyses

There were no qualitative (different directions of effects) nor quantitative (different sizes of effects) differences in any of the sensitivity analyses including: (a) unadjusted analyses, (b) complete case (unadjusted), (c) studies at low risk of bias, (d) full model with 18 nodes and (e) complete case with additional five studies that had too much missing data to be included in the adjusted models (Tables S12 and S13).

As an example, the effect sizes for the STH versus placebo comparison for height gain are in the table below (Table 14).

**Table 14:** Sensitivity analyses for STH versus placebo

Sensitivity analysis	STH vs. placebo
Base case	0.09 (−0.08,0.27)
Unadjusted analyses	0.09 (−0.08,0.27)
Complete case (unadjusted), 14 studies	0.06 (−0.24,0.35)
Studies at low risk of bias,	0.11 (−0.11,0.33)
Full model with 18 nodes, 14 studies	Albendazole 2/year vs. placebo: 0.09 (−0.09,0.28) Mebendazole 2/year vs. placebo: 0.13 (−1.47,1.74)
Complete case with additional five studies	0.06 (−0.19,0.31)

Abbreviation: STH, soil‐transmitted helminthiasis.

##### Comparison of effect sizes for height gain (cm) between received data and studies that did not provide data

We assessed whether the effects on height gain were similar for these studies which did not provide data (either because they did not provide it or because they did not meet eligibility criteria) to the studies which did provide data (Appendix 3). The test for interaction for subgroup difference was not statistically significant (*p* = .25), with an effect on height gain for studies for which we received data of 0.04 cm (95% CI: −0.04, 0.12) compared to an effect of 0.24 cm (95% CI: −0.01, 0.30). The pooled effect on height gain across these 28 studies for STH deworming versus placebo was 0.09 cm (95% CI: 0.01, 0.17). This analysis included four studies of SAT (Sarkar, Anwar, Biswas, & Mannan, 2002; Simeon et al., 1995; Tee, Lee, Noorizan, Noori, & Raj, 2013; Yap et al., 2014) since we decided to include these studies since our model adjusts for infection intensity. In this aggregate level analysis, there is no adjustment for infection intensity.

#### 5.11.3 Haemoglobin

##### Haemoglobin base case analyses

**Figure 18:**
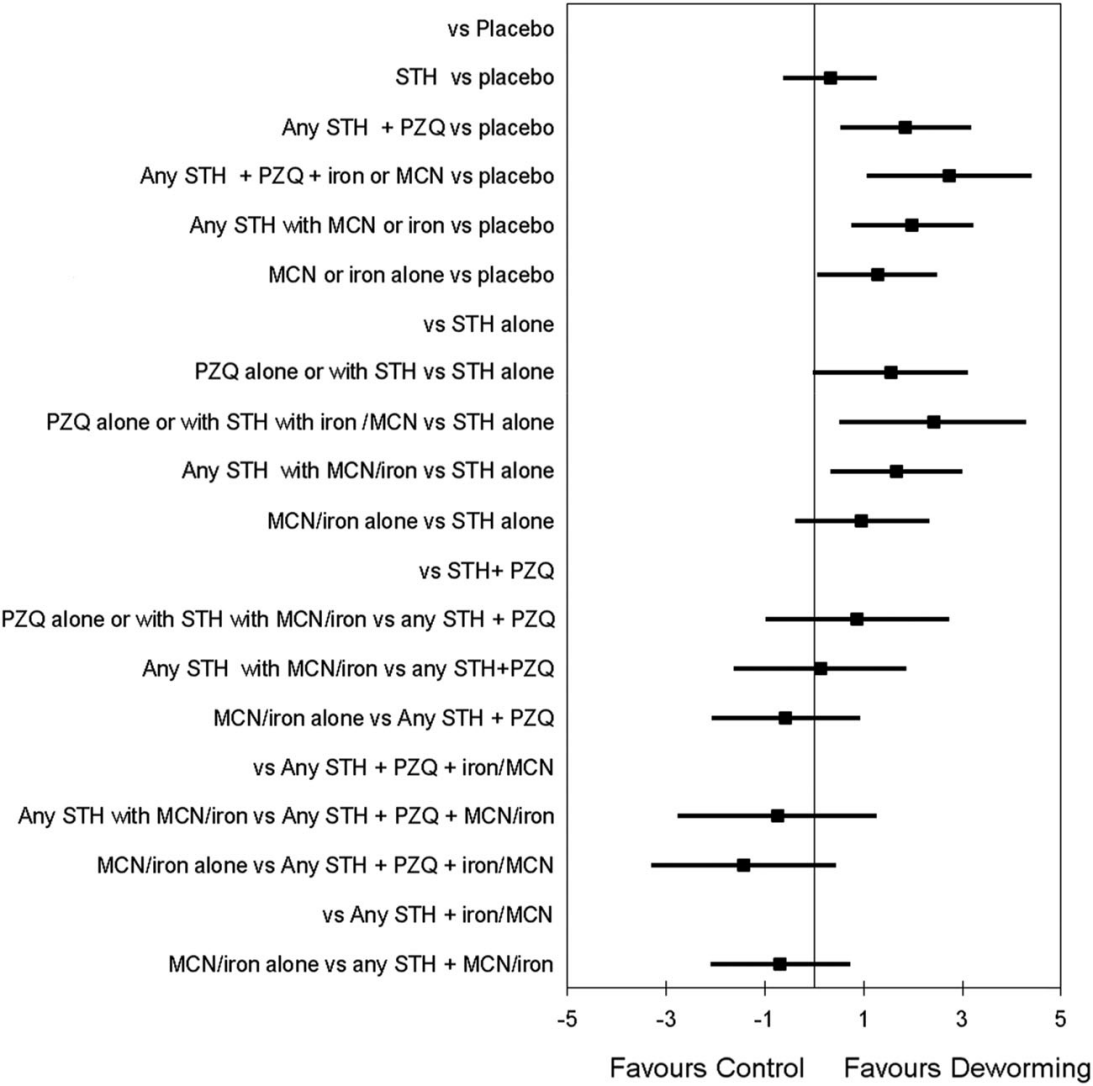
Change in haemoglobin (g/L), base case collapsed network, adjusted for covariates

The effect of STH deworming alone versus placebo was 0.32 g/L (95% CI: −0.63, 1.26) (Figure 18). Deworming for schistosomiasis with or without STH deworming increased haemoglobin by 1.85 g/L (95% CI: 0.53, 3.18) versus placebo. Deworming for schistosomiais with or without STH deworming and micronutrients or iron increased haemoglobin by 2.72 g/L (95% CI: 1.05, 4.40) compared to placebo. Deworming for STH combined with iron and/or micronutrients increased haemoglobin by 1.98 g/L (95% CI: 0.74, 3.21) compared to placebo. The effect of micronutrients and/or iron versus placebo was 1.28 g/L (95% CI: 0.07, 2.49). This latter effect must be interpreted with caution since there are many other studies of micronutrient and iron supplementation in children that are not included in this review.

##### Direct evidenceaggregate and IPD

When comparing the three effect sizes of aggregate direct evidence, IPD direct evidence and IPD‐NMA direct and indirect evidence, we show the results below for the STH versus placebo and STH + micronutrients/iron comparisons to placebo, showing similar size and direction of effects for both comparisons (Table 15).

**Table 15:** Direct and indirect evidence for haemoglobin STH versus placebo

Analysis	Effect size STH vs. placebo	STH+MCN/iron vs. placebo
Direct‐aggregate	0.23 (−0.52, 0.97) *I* ^2^ 0%	2.18 (1.02, 3.35), *I* ^2^ 18%
Direct IPD	0.22 (−0.74,1.19)	1.76 (0.41,3.11)
IPD‐NMA direct and indirect	0.32 (−0.63,1.26)	1.98 (0.74,3.21)

Abbreviation: IPD, individual participant data; MCN, micronutrients; NMA, network meta‐analysis; STH, soil‐transmitted helminthiasis.

Analysis of the direct evidence of aggregate data from studies confirmed heterogeneity <75% for all comparisons (Appendix 3).

##### Sensitivity analyses

All sensitivity analyses were congruent with these main findings including the complete case model (14 studies, six nodes, unadjusted), complete case with additional five studies (unadjusted) and the unadjusted 14 study model (Table 16).

**Table 16:** Sensitivity analyses for haemoglobin

Sensitivity analysis	STH vs. placebo	STH+MCN/iron vs. placebo
Base case	0.32 (−0.63,1.26)	1.98 (0.74,3.21)
Unadjusted analyses	0.32 (−0.63,1.26)	1.98 (0.74,3.21)
Complete case (unadjusted), 14 studies	0.30 (−0.69,1.29)	3.18 (1.28,5.09)
Studies at low risk of bias,	0.07 (−1.05,1.19)	2.48 (0.57,4.39)
Full model with 18 nodes, 14 studies	Albendazole 2/year: 0.16 (−0.86, 1.17) Mebendazole 2/year: −0.10 (−6.61, 6.41)	Alben 2/year + foritifed beverage: 0.69 (−1.09, 2.48) Meben 2/year+ iron: 2.73 (−0.06, 5.51)
Complete case with additional five studies	0.37 (−0.77,1.51)	1.83 (0.05,3.61)
Studies with greater impact on infection prevalence	0.72 (−1.06,2.50)	2.74 (0.95,4.52)

Abbreviation: MCN, micronutrients; STH, soil‐transmitted helminthiasis.

Analysis of the six studies with greatest impact on *A. lumbricoides* prevalence at endline (relative risk of 0.80 or greater when compared to placebo) found an effect of 2.74 g/L (95% CI: 0.95, 4.52) of STH deworming with micronutrients or iron compared to placebo.

##### Comparison of effect sizes for haemoglobin (g/L) between received data and studies that did not provide data

We assessed whether the effects on haemoglobin were similar for these studies which did not provide data (either because they did not provide it or because they did not meet eligibility criteria) to the studies which did provide data (Appendix 3). The test for interaction for subgroup differences for STH deworming versus placebo was not statistically significant (*p* = .33). The effect size was 0.05 g/L (95% CI: −0.02, 0.11) compared to an effect size of studies for which data was not received of 0.00 (95% CI: −0.05, 0.06). Also, when sorted by year of publication, there was no pattern in effect size based on the year in which the study was published.

#### 5.11.4 Cognition

Six studies provided IPD data on cognition outcomes (Ebenezer et al., 2013; Liu et al., 2017; Nga et al., 2009; Rohner et al., 2010; Solon et al., 2003; Stoltzfus et al., 2004). These were analysed separately for each study.

The baseline means and range of minimum and maximum scores at baseline are given below to aid in interpreting the effect sizes observed (Table 17).

**Table 17:** Baseline cognition measures for each study

Studies	Outcome	Mean	Minimum	Maximum
Ebenezer	Single digit attention score	12.2	0.0	20.0
	Double digit attention score	7.2	0.0	20.0
	Math score	34.4	0.0	100.0
	Tamil language score	43.2	0.0	100.0
Liu	Processing speed index	86.2	45.0	138.0
	Working memory index	78.6	45.0	147.0
	TIMSS *z* score	0.0	−2.4	2.1
Nga	Raven score	16.4	0.0	35.0
	Digit forward	7.0	2.0	9.0
	Digit back	2.9	0.0	8.0
	Block score	11.9	0.0	47.0
	Code score	31.3	1.0	55.0
Stoltzfus04	Language skills	10.2	0.0	18.0
	Motor skills	13.7	0.0	20.0
Solon	Verbal ability	7.5	0.0	16.0
	Quantitative ability	7.4	0.0	12.0
	Nonverbal ability	2.7	0.0	7.0
	Total cognition	17.6	2.0	34.0
Rohner	Raven score	13.3	1.0	22.0
	Coding total	18.0	3.0	43.0
	Symbols total	8.6	1.0	20.0
	Target marking errors	0.1	0.0	8.0
	Target marking time	45.5	20.0	105.0

Nga et al. (2009) found that digit forward was 0.38 (95% CI: 0.06, 0.71) units higher for albendazole + fortified biscuit compared to unfortified biscuit, and that digit forward was also improved for fortified biscuit alone compared to unfortified biscuit (0.57, 95% CI: 0.25, 0.88). All other outcomes had nonsignificant effects (see Table 18).

**Table 18:** Studies providing cognition data

Study	Treatment	Comparator	Cognition outcome	EE (no covariates)	95% LCI	95% UCI	EE (IPD‐NMA covariates)	95% LCI	95% UCI	EE Diff. (NMA cov. − No cov.)
Short‐term attention										
**Ebenezer**	Mebendazole + iron	Placebo	Single digit attention score	−0.11	−1.08	0.87	−0.11	−1.09	0.87	0.00
**Ebenezer**	Mebendazole + iron	Placebo	Double digit attention score	−0.05	−0.92	0.82	−0.07	−0.95	0.80	0.02
**Liu**	Albendazole	Placebo	Processing speed index	0.84	−0.39	2.07	0.90	−0.35	2.15	−0.06
**Liu**	Albendazole	Placebo	Working memory index	0.45	−0.51	1.42	0.50	−0.47	1.47	−0.04
**Nga**	Albendazole	Placebo	Digit forward	0.26	−0.05	0.58	0.27	−0.04	0.59	−0.01
**Nga**	Albendazole	Placebo	Digit back	0.08	−0.20	0.36	0.13	−0.14	0.41	−0.05
**Nga** ^a^	Albendazole + micronutrients	Placebo	Digit forward	**0.38**	**0.06**	**0.71**	**0.39**	**0.06**	**0.71**	0.00
**Nga**	Albendazole + micronutrients	Placebo	Digit back	−0.07	−0.36	0.22	−0.06	−0.34	0.22	−0.01
**Nga** ^a^	Micronutrients	Placebo	Digit forward	**0.57**	**0.25**	**0.88**	**0.59**	**0.27**	**0.90**	−0.02
**Nga**	Micronutrients	Placebo	Digit back	−0.04	−0.32	0.24	0.00	−0.28	0.28	−0.04
Rohner	Albendazole + praziquantel	Placebo	Coding total	0.24	NA	NA	0.53	−2.36	3.42	−0.29
Rohner	Albendazole + praziquantel + iron	Placebo	Coding total	−2.37	NA	NA	−2.20	−5.04	0.64	−0.17
Rohner	Iron fortified food or beverage	Placebo	Coding total	−0.51	NA	NA	−0.12	−3.07	2.83	−0.39
Rohner	Albendazole + praziquantel	Placebo	Symbols total	−0.01	NA	NA	0.74	−1.22	2.70	−0.75
Rohner	Albendazole + praziquantel + iron	Placebo	Symbols total	−0.55	NA	NA	0.06	−1.86	1.97	−0.60
Rohner	Iron fortified food or beverage	Placebo	Symbols total	0.18	NA	NA	0.97	−1.05	2.98	−0.78
Rohner	Albendazole + praziquantel	Placebo	Target marking errors	−0.07	NA	NA	−0.08	−0.44	0.28	0.01
Rohner	Albendazole + praziquantel + iron	Placebo	Target marking errors	0.11	NA	NA	0.09	−0.26	0.45	0.02
Rohner	Iron fortified food or beverage	Placebo	Target marking errors	−0.03	NA	NA	−0.02	−0.38	0.35	−0.01
Rohner	Albendazole + praziquantel	Placebo	Target marking time	−2.10	NA	NA	−2.81	−10.80	5.18	0.71
Rohner	Albendazole + praziquantel + iron	Placebo	Target marking time	1.11	NA	NA	0.55	−7.26	8.36	0.56
Rohner	Iron fortified food or beverage	Placebo	Target marking time	4.06	NA	NA	4.28	−3.85	12.40	−0.21
School achievement (math and language)										
**Ebenezer**	Mebendazole + iron	Placebo	Math score	2.30	−0.24	4.84	2.31	−0.19	4.81	−0.01
**Ebenezer**	Mebendazole + iron	Placebo	Tamil language score	−1.37	−3.79	1.06	−1.34	−3.73	1.06	−0.03
**Liu**	Albendazole	Placebo	TIMSS *z* score	−0.03	−0.11	0.05	−0.03	−0.11	0.05	0.00
**Nga**	Albendazole	Placebo	Block score	1.14	−0.59	2.88	1.03	−0.71	2.77	0.11
**Nga**	Albendazole	Placebo	Code score	−0.01	−2.20	2.18	0.14	−2.05	2.32	−0.15
**Nga**	Albendazole + micronutrients	Placebo	Block score	−0.71	−2.52	1.10	−0.63	−2.45	1.19	−0.08
**Nga**	Albendazole + micronutrients	Placebo	Code score	1.19	−0.98	3.35	1.21	−0.95	3.37	−0.02
**Nga**	Micronutrients	Placebo	Block score	−0.53	−2.28	1.21	−0.58	−2.34	1.18	0.05
**Nga**	Micronutrients	Placebo	Code score	1.28	−0.94	3.51	1.38	−0.84	3.61	−0.10
Solon	Albendazole	Placebo	Verbal ability	−0.01	−0.61	0.60	−0.02	−0.63	0.58	0.01
Solon	Albendazole	Placebo	Quantitative ability	0.04	−0.41	0.48	0.01	−0.44	0.45	0.03
Solon	Albendazole	Placebo	Nonverbal ability	0.08	−0.31	0.47	0.09	−0.30	0.48	−0.01
Solon	Albendazole + micronutrients	Placebo	Verbal ability	0.38	−0.18	0.94	0.38	−0.18	0.94	0.00
Solon	Albendazole + micronutrients	Placebo	Quantitative ability	0.06	−0.37	0.49	0.05	−0.38	0.48	0.01
Solon	Albendazole + micronutrients	Placebo	Nonverbal ability	−0.10	−0.47	0.27	−0.08	−0.46	0.29	−0.02
Solon	Micronutrient	Placebo	Verbal ability	0.15	−0.39	0.70	0.11	−0.44	0.66	0.04
Solon	Micronutrients	Placebo	Quantitative ability	0.17	−0.27	0.61	0.16	−0.28	0.60	0.01
Solon	Micronutrients	Placebo	Nonverbal ability	−0.11	−0.51	0.29	−0.08	−0.48	0.31	−0.03
Development										
**Nga**	Albendazole	Placebo	Raven score	0.05	−1.04	1.14	0.00	−1.06	1.07	0.05
**Nga**	Albendazole + micronutrients	Placebo	Raven score	0.19	−0.93	1.31	0.14	−0.96	1.24	0.06
**Nga**	Micronutrients	Placebo	Raven score	0.82	−0.26	1.90	0.80	−0.26	1.86	0.02
Rohner	Albendazole + praziquantel	Placebo	Raven score	0.89	NA	NA	0.98	−1.07	3.02	−0.08
Rohner	Albendazole + praziquantel + iron	Placebo	Raven score	−1.04	NA	NA	−1.24	−3.23	0.75	0.20
Rohner	Iron fortified food or beverage	Placebo	Raven score	−1.44	NA	NA	−1.40	−3.47	0.67	−0.04
**Stoltzfus04**	Iron	Placebo	Language skills	0.17	−1.03	1.38	0.41	−0.77	1.59	−0.24
**Stoltzfus04**	Iron	Placebo	Motor development	0.01	−0.08	0.09	−0.01	−0.09	0.07	0.02
**Stoltzfus04**	Mebendazole (high)	Placebo	Language skills	0.06	−1.13	1.25	0.31	−0.87	1.50	−0.26
**Stoltzfus04**	Mebendazole (high)	Placebo	Motor development	0.02	−0.06	0.11	0.00	−0.09	0.09	0.02
**Stoltzfus04**	Mebendazole (high) + iron	Placebo	Language skills	0.36	−0.77	1.49	0.60	−0.52	1.73	−0.24
**Stoltzfus04**	Mebendazole (high) + iron	Placebo	Motor development	0.01	−0.07	0.10	0.01	−0.08	0.09	0.01
Solon	Albendazole + micronutrients	Placebo	Total cognition	0.34	−0.51	1.19	0.35	−0.50	1.21	−0.01
Solon	Albendazole + micronutrients	Placebo	Total cognition	0.11	−0.85	1.06	0.07	−0.89	1.04	0.03
Solon	Micronutrients	Placebo	Total cognition	0.21	−0.66	1.09	0.19	−0.69	1.06	0.03

Abbreviation: cov., covariate; IPD, individual participant data; LCI, lower confidence interval; NMA, network meta‐analysis; UCI, upper confidence interval.

^a^ Bold values are statistically significant differences between the intervention and placebo groups.

### 5.12 Effect modifier analyses

We conducted subgroup analyses across each of the nine factors that were deemed important by our advisory group.

There were insufficient numbers of children with moderate and high intensity infections (as defined using the WHO cut‐offs) to run the NMA model. Thus, we decided with our advisory board to use three categories based on the distribution to assess whether there is a gradient in effect across infection intensity. Light and moderate infection intensity cutoffs were defined by the median infection intensity in children who were infected across the whole population of children in all 14 studies in the base case.

To further assess whether there was a gradient in effect size across infection intensity, we conducted subgroup analysis for the comparison with the most available data (STH deworming vs. placebo). In this comparison of direct evidence from trials, 15% of children had moderate or heavy infections according to WHO cut‐offs for ascaris, *T. trichiura* and hookworm.

#### 5.12.1 BMI for age as effect modifier

##### Weight

Tests for interaction across BMI for age were not statistically significant for weight gain across any comparison (Figures S13 and Table S18).

##### Height

Tests for interaction were not statistically significant across BMI for age for height gain in cm for any comparison (Figure S14; Tables S15 and S18).

##### Haemoglobin

There were no statistically significant subgroup differences across BMI for age for change in haemoglobin for any comparison (Figure S15 and Table S15).

##### Cognition

The test for interaction was not statistically significant across BMI for age for cognition for any comparison (Table S16).

#### 5.12.2 Height for age‐as effect modifier

##### Weight

The test for interaction was not statistically significant across levels of height for age for weight gain for any comparison (Figure S16 and Table S18).

##### Height

The test for interaction was not statistically significant across levels of height for age for height gain for any comparison (Figure S17 and Table S18).

##### Haemoglobin

The test for interaction was not statistically significant across levels of height for age for change in haemoglobin for any comparison (Figure S18 and Table S18).

##### Cognition

When cognition for each of six studies (Ebenezer, Liu, Nga, Rohner, Solon and Stoltzfus 2004) was analysed according to subgroups of height for age of stunted (<−2 HAZ) or not stunted (≥−2.0 HAZ), there were no statistically significant effects on cognition, except for three comparisons. In these comparisons, albendazole combined with fortified biscuits resulted in better improvement of −0.85 units on digit forward (95% CI: −1.52, −0.18) compared to −0.20 (95% CI: −0.57, 0.17) for children who were not stunteda. In the same study (Nga), fortified biscuits resulted in improvement of digit forward of −0.54 (9% CI: −0.90 to −0.18) for children with normal HAZ (>−2), and similar improvement in stunted children (−0.78 digit forward units, 95% CI: −1.42, −0.14) (Table S16).

#### 5.12.3 Sex, as an effect modifier

##### Weight

The test for interaction for subgroup effects was not statistically significant across sex for weight gain for any comparison (Figure S19 and Table S16).

##### Height

The test for interaction for subgroup effects was not statistically significant across sex for height gain for any comparison (Figure S20 and Table S16).

##### Haemoglobin

The test for interaction for subgroup effects was not statistically significant across sex for change in haemoglobin for any comparison (Figure S21 and Table S16).

##### Cognition

Tests for interaction for subgroup effects across sex were not statistically significant for cognition for any outcome measure or any comparison (Table S16).

#### 5.12.4 Age, as effect modifier

##### Weight

Tests for interaction for subgroup effects across age were not statistically significant for weight gain for any comparison (Figure S22 and Table S16).

##### Height

The relatively small number of participants <5 years of age led to wide CIs for estimates in this age group. Tests for interaction for subgroup effects across age were not statistically significant for height gain for any comparison (Figure S23 and Table S16).

##### Haemoglobin

Some comparisons did not have any children <5 years of age. Tests for interaction for subgroup effects across age were not statistically significant for change in haemoglobin for any comparison (Figure S24 and Table S16).

##### Cognition

Studies that reported cognition outcomes did not have children <5 years.

#### 5.12.5 *A. lumbricoides*, as effect modifier

We conducted two analyses because of the limited number of children with moderate or heavy intensity infections:

1) NMA with IPD using cutoffs based on the distribution of intensity in the sample of three levels, and
2) Direct evidence analysis with IPD using WHO cutoffs for intensity of infection.

##### Weight

For the NMA‐IPD, tests for interaction for subgroup effects across *A. lumbricoides* intensity were not statistically significant for weight gain for the NMA. When using cut‐offs for intensity of infection for *A. lumbricoides* based on the median distribution across three levels: none detected, lighter intensity (1–1,776 epg), and higher intensity (>1,776 epg), the effect for children with higher intensity was 0.08 kg (95% CI: −0.13, 0.29) (Table S15).

For the analysis of STH deworming versus placebo using direct evidence only for weight across three levels of *A. lumbricoides* infection using WHO cutoffs: none detected, light (1–4,999 epg) and moderate/heavy (≥5,000 epg), the interaction test for subgroup effects was not statistically significant. The effects for children with moderate or heavy intensity of *A. lumbricoides* infection was 0.12 kg (−0.05, 0.28) which is higher than the effect for those with no detected infection (−0.01 kg (95% CI: −0.11, 0.09) or those with light infection intensity (0.04 kg, 95% CI: −0.07, 0.15) (Figure S25).

**Figure 19:**
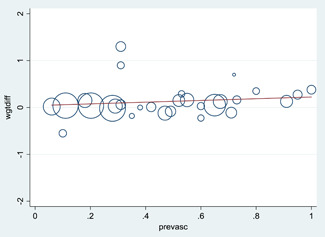
Meta‐regression according to prevalence of *Ascaris lumbricoides* for difference in weight gain at aggregate level

In order to explore the role of *A. lumbricoides* prevalence further, we conducted a meta‐regression according to prevalence of *A. lumbricoides* at the study level using aggregate data for all 30 studies available with STH deworming versus placebo. We chose this comparison since it is the comparison with the most data. The results yielded a coefficient of 0.18 (*SE*, 0.24), *p* = .455, 95% CI: −0.313, 0.68) with an adjusted *R* ^2^ of −2.97% (proportion of between‐study variance explained by prevalence of ascaris). These results indicate that *A. lumbricoides* prevalence was not a significant predictor of the effectiveness of deworming (Figure 19).

##### Height

Tests for interaction for subgroup effects across *A. lumbricoides* intensity were not statistically significant for height gain for the NMA. When using cut‐offs for intensity of infection for *A. lumbricoides* based on the median distribution across three levels: none detected, lighter intensity (1–1,776 epg), and higher intensity (>1,776 epg), the effect modification for children with higher intensity was 0.04 cm (95% CI: −0.22, 0.30) (Table S15).

For the posthoc direct evidence analysis of STH deworming versus placebo for height gain across three levels of *A. lumbricoides* infection, using WHO cutoffs: none detected, light (1–4,999 epg) and moderate/heavy (≥5,000 epg), the interaction test for subgroup effects was not statistically significant. The effect for children with moderate or heavy intensity of *A. lumbricoides* infection was 0.07 cm (95% CI: −0.07, 0.22) (Figure S26).

##### Haemoglobin

Tests for interaction for subgroup effects across *A. lumbricoides* intensity were not statistically significant for change in haemoglobin for the NMA. When using cut‐offs for intensity of infection for *A. lumbricoides* based on the median distribution across three levels: none detected, lighter intensity (1–1,776 epg), and higher intensity (>1,776 epg), the effect modification for children with higher intensity was 0.48 g/L (95% CI: −0.69, 1.66) (Table S15).

For the posthoc analysis of direct evidence of STH deworming versus placebo for haemoglobin across three levels of *A. lumbricoides* infection, using WHO cutoffs: none detected, light (1–4,999 epg) and moderate/heavy (≥5,000 epg), the interaction test for subgroup effects was not statistically significant. The effect for children with moderate or heavy intensity of *A. lumbricoides* infection was 0.44 g/L (95% CI: −2.49, 1.60) (Figure S27).

##### Cognition

Tests for interaction for subgroup effects across *A. lumbricoides* intensity were not statistically significant for single digit attention scores, math scores, Tamil language scores, processing speed index, working memory index, TIMSS *z* score, digit forward, digit back, block score and code score (Table S16).

#### 5.12.6 Hookworm, as effect modifier

Two analyses were conducted: (a) NMA using cutoffs based on the distribution of intensity in the sample of three levels and (b) direct evidence analysis using WHO cutoffs for intensity of infection.

##### Weight

Tests for interaction for subgroup effects in the NMA across hookworm intensity were not statistically significant for weight gain for any comparison. When using cut‐offs for intensity of infection for hookworm based on the median distribution across three levels: none detected, lighter intensity (1–384 epg), and higher intensity (>384 epg), the effect modification for children with higher intensity was 0.16 kg (95% CI: −0.13, 0.46) (Table S15).

For the direct evidence, posthoc analysis using random effects pairwise meta‐analysis of STH deworming versus placebo for weight gain across three levels of hookworm infection using WHO cutoffs: none detected, light (1–1,999 epg) and moderate/heavy (≥2,000 epg), the interaction test for subgroup effects was not statistically significant. The effect for children with moderate or heavy intensity of hookworm infection was −0.53 kg (95% CI: −2.09, 1.03) (Figure S28).

**Figure 20:**
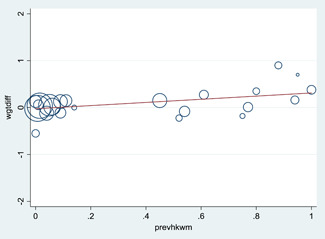
Meta‐regression of hookworm prevalence at aggregate level for 23 studies with data on STH versus placebo. STH, soil‐transmitted helminthiasis

To further assess the role of prevelance of hookworm, we conducted meta‐regression using aggregate level data for 23 studies with data on hookworm prevalence for the comparison of STH deworming versus placebo. The proportion of variance explained is 54%, *p* = .014, showing a positive relationship of weight gain with hookworm infection prevalence (Figure 20).

##### Height

Tests for interaction for subgroup effects across hookworm intensity were not statistically significant for height for any comparison in the NMA. When using cut‐offs for intensity of infection for hookworm based on the median distribution across three levels: none detected, lighter intensity (1–384 epg), and higher intensity (>384 epg), the effect modification for children with higher intensity was 0.20 cm (95% CI: −0.13, 0.52) (Table S15).

For the direct evidence, posthoc analysis of STH deworming versus placebo for height gain across three levels of hookworm infection, using WHO cutoffs: none detected, light (1–1,999 epg) and moderate/heavy (≥2,000 epg), the interaction test for subgroup effects was not statistically significant. The effect for children with moderate or heavy intensity of hookworm infection was −0.17 cm (95% CI: −0.52, 0.18) (Figure S29).

##### Haemoglobin

Tests for interaction for subgroup effects across hookworm intensity were not statistically significant for change in haemoglobin for any comparison in the NMA. When using cut‐offs for intensity of infection for hookworm based on the median distribution across three levels: none detected, lighter intensity (1–384 epg) and higher intensity (>384 epg), the effect modification for children with higher intensity was 3.58 g/L (95% CI: 0.13, 7.02) (Table S15).

For the direct evidence, posthoc analysis of STH deworming versus placebo for haemoglobin across three levels of hookworm infection using WHO cutoffs: none detected, light (1–1,999 epg) and moderate/heavy (≥2,000 epg), the interaction test for subgroup effects was not statistically significant. The effect for children with moderate or heavy intensity of hookworm infection was −0.56 g/L (95% CI: −6.39, 5.27) (Figure S30).

##### Cognition

Tests for interaction for subgroup effects across hookworm intensity were not statistically significant for any comparison for single digit attention scores, math scores, Tamil language scores, processing speed index, working memory index, TIMSS *z* score, digit forward, digit back, block score and code score (Table S16).

#### 5.12.7 *T. trichiura*, as effect modifier

We conducted two analyses: (a) NMA using cutoffs based on the distribution of intensity in the sample of three levels and (b) direct evidence analysis using WHO cutoffs for intensity of infection.

##### Weight

Tests for interaction for subgroup effects across *T. trichiura* intensity were not statistically significant for weight gain for any comparison in the NMA. When using cut‐offs for intensity of infection for *T. trichiura* based on the median distribution across three levels: none detected, lighter intensity (1–288 epg), and higher intensity (>288 epg), the effect modification for children with higher intensity was 0.17 kg (95% CI: −0.06, 0.41) (Table S15).

For the direct evidence, posthoc analysis of STH deworming versus placebo for weight gain across three levels of *T. trichiura* infection using WHO cutoffs: none detected, light (1–999 epg) and moderate/heavy (≥1,000 epg), the interaction test for subgroup effects was not statistically significant. However, the effect for children with moderate or heavy intensity of *T. trichiura* infection was 0.11 kg (−0.14, 0.35) which was higher than for those with no detected infection (Figure S31).

##### Height

Tests for interaction for subgroup effects across *T. trichiura* intensity were not statistically significant for height for any comparison in the NMA models. When using cut‐offs for intensity of infection for *T. trichiura* based on the median distribution across three levels: none detected, lighter intensity (1–288 epg), and higher intensity (>288 epg), the effect modification for children with higher intensity was 0. 07 cm (−0.02, 0.34) (Table S15).

For the direct evidence, posthoc analysis of STH deworming versus placebo for height gain across three levels of *T. trichiura* infection using WHO cutoffs: none detected, light (1–999 epg) and moderate/heavy (≥1,000 epg), the interaction test for subgroup effects was not statistically significant. The effect for children with moderate or heavy intensity of *T. trichiura* infection was −0.17 cm (−0.52, 0.18) (Figure S32).

##### Haemoglobin

Tests for interaction for subgroup effects across *T. trichiura* intensity were not statistically significant for haemoglobin for any comparisonmin the NMA models. When using cut‐offs for intensity of infection for *T. trichiura* based on the median distribution across three levels: none detected, lighter intensity (1–288 epg), and higher intensity (>288 epg), the effect modification for children with higher intensity was 1.33 g/L (−1.14, 3.81) (Table S15).

For the direct evidence, posthoc analysis of STH deworming versus placebo for change in haemoglobin across three levels of *T. trichiura* infection using WHO cutoffs: none detected, light (1–999 epg) and moderate/heavy (≥1,000 epg), the interaction test for subgroup effects was not statistically significant. The effect for children with moderate or heavy intensity of *T. trichiura* infection was 0.33 g/L (−2.99, 3.65) (Figure S33).

##### Cognition

Tests for interaction for subgroup effects across *T. trichiura* intensity were not statistically significant for any comparison for single digit attention scores, math scores, Tamil language scores, processing speed index, working memory index, TIMSS *z* score, digit forward, digit back, block score and code score (Table S16).

#### 5.12.8 Any helminth infection, as effect modifier

##### Weight

Tests for interaction for subgroup effects across a composite category of intensity of infection for any parasite were not statistically significant for weight gain for any comparison in the NMA‐IPD model (Figure S34 and Table S15).

##### Height

Tests for interaction for subgroup effects across a composite category of intensity of infection for any parasite were not statistically significant for height gain for any comparison (Figure S35and Table S15).

##### Haemoglobin

Tests for interaction for subgroup effects across a composite category of intensity of infection for any parasite were not statistically significant for change in haemoglobin for any comparison (Figure S36and Table S15).

##### Cognition

Tests for interaction for subgroup effects across a composite category of intensity of infection for any parasite were not statistically significant for any comparison for single digit attention scores, math scores, Tamil language scores, processing speed index, working memory index, TIMSS *z* score, digit forward, digit back, block score and code score (Table S16).

#### 5.12.9 Anaemia as an effect modifier

##### Weight

Tests for interaction for subgroup effects across anaemia were not statistically significant for weight gain for any comparison (Figure S37 and Table S15).

##### Height

Tests for interaction for subgroup effects across anaemia were not statistically significant for height gain for any comparison (Figure 38 and Table S15).

##### Haemoglobin

**Figure 21:**
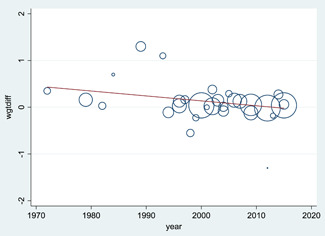
Metaregression according to year of publication for difference in weight gain (kg)

Tests for interaction for subgroup effects across anaemia were not statistically significant for change in haemoglobin for any comparison (Table S15; Figures 21 and S39).

##### Cognition

Tests for interaction for subgroup effects across anaemia were not statistically significant for any comparison for single digit attention scores, math scores, Tamil language scores, working memory index, TIMSS *z* score, digit forward, digit back, block score and code score (Table S16).

#### 5.12.10 Year of publication

We planned to restrict our IPD‐NMA to studies conducted 2008 or later. However, we decided that it would be more informative to conduct a meta‐regression using aggregate data according to year of publication to include older studies for which we were unable to obtain individual participant datasets.

This analysis shows a negative association, with a greater effect in older studies which was not statistically significant (*p* = .05) and explained 7.88% of the variance between studies. The graph shows a concentration of more recent studies with smaller effects on weight gain.

### 5.13 Comparison with other recent systematic reviews for STH deworming versus placebo

We compared our findings for weight gain, height gain and haemoglobin and cognition to a Cochrane review (Taylor‐Robinson et al., 2015) and prior Campbell review, which both used aggregate level data (Table 19).

**Table 19:** Comparison of results with other recent systematic reviews and meta‐analyses

Review	Taylor‐Robinson et al. (2015), Regular frequency STH deworming (2/year) vs. placebo	Welch et al. (2016), Albendazole 2/year vs. placebo	Welch et al. (2018) STH deworming vs. placebo
Weight gain	0.08 kg (95% CI: −0.11, 0.27)	0.09 kg (95% CI: −0.04, 0.2),	IPD‐NMA: 0.01 kg (95% CI: −0.08, 0.11) Aggregate level, all studies (received, not received and ineligible due to lack of baseline infection intensity: 0.07 (95% CI: 0.01, 0.13) random effects
Height gain	0.02 cm (95% CI: −0.14, 0.17)	0.07 cm (95% CI: −0.1, 0.24 cm),	0.09 cm (95% CI: −0.08, 0.27)
Change in haemoglobin	0.02 g/dl, (95% CI: −0.08, 0.04)	Not pooled, concluded there were effects only when combined with micronutrients, iron or praziquantel	0.32 g/L (95% CI: −0.63, 1.26).
Cognition	Little to no effect	0.23 points on a 100 point scale (95% CI: −0.6, 0.14)	Little to no effect

Abbreviation: IPD, individual participant data.

Also, the Welch et al. 2016 review assessed the relationship of aggregate data with prevalence of each type of helminth infection, and found no relationship using two different methods. The findings of this systematic review and IPD‐NMA are in agreement with this, using IPD‐NMA effect modification tests for subgroup effects, and aggregate data subgroup analysis as well as meta‐regression across prevalence of ascaris. Unlike our prior systematic review, we did find a statistically significant relationship with hookworm prevalence and effect on weight gain.

We also compared our findings to Croke et al. meta‐analysis (Croke et al., 2016; Table 20). This comparison contains 34 possible effect estimates from 33 studies of STH deworming versus placebo. The meta‐analysis by Croke et al. does not include 13 estimates which we have included, two because they are SAT studies (Sarkar et al., 2002; Yap et al. 2014). The remaining studies, it is unclear why they were excluded since no table of excluded studies is provided. We excluded four studies where STH was combined with micronutrients or iron and compared to micronutrients or iron. This is because of our decision to keep this as a separate treatment comparison. The table below shows that our effect estimate is lower than Croke et al. for both fixed and random effects. Even when we conduct a sensitivity analysis, adding the four studies of STH + micronutrients versus micronutrients, we still have a lower effect estimate than Croke et al. (2016) with a random effects meta‐analysis of 0.10 kg (95% CI: 0.03, 0.17) and fixed effects 0.07 (95% CI: 0.04, 0.10).

**Table 20:** Comparison of Croke et al. (2016) with data from this review and Welch et al. 2016 systematic review

			Welch et al. (2016, 2018)		Croke et al. (2016)	
	Effect estimate and 95% CI	Sample size (children)^a^	Weight in meta‐analysis (%)	Aggregate data estimate	Weight in meta‐analysis (%)	Effect estimate and 95% CI
Included in both, with same estimate and SE	**Alderman** 2006 **(cluster)**	**1,3055**	4.10	0.15 (−0.03, 0.33)	6.02	0.15 (−0.02, 0.33)
	**Dossa vs. placebo 2001**	**65**	1.90	0.00 (−0.52, 0.52)	0.68	0.00 (−0.52, 0.52)
	**Gateff** 1972	**140**	2.70	0.35 (−0.03, 0.73)	2.81	0.35 (0.09, 0.60)
	**Gupta** 1982	**39**	2.90	0.03 (−0.32, 0.38)	1.56	0.03 (−0.32, 0.37)
	**Joseph** 2015	**440**	4.70	0.04 (−0.06, 0.14)	18.40	0.04 (−0.06, 0.14)
	**Ostwald** 1984	**45**	0.90	0.70 (−0.18, 1.58)	0.24	0.70 (−0.18, 1.58)
	**Stephenson** 1993	**93**	2.40	0.90 (0.54, 1.26)	1.41	0.90 (0.54, 1.26)
	**Watkins** 1996	**110**	4.10	0.13 (−0.08, 0.34)	4.27	0.13 (−0.08, 0.34)
	**Willett** 1979	**137**	4.10	0.16 (−0.03, 0.35)	6.67	0.16 (−0.01, 0.33)
Included in both, same effect, different SE	**Sur** 2005	**341**	2.60	0.29 (−0.10, 0.68)	6.11	0.29 (0.12, 0.47)
Included in both, different point estimates	**Awasthi** 2000	**387**	4.70	0.04 (−0.06, 0.14)	8.29	−0.05 (−0.20, 0.10)
	**Donnen** 1998	**117**	2.90	−0.55 (−0.89, −0.21)	1.73	−0.45 (−0.78, −0.12)
	**Kruger** 1996	**91**	3.90	0.03 (−0.15, 0.21)	0.93	−0.38 (−0.82, 0.06)
	**Liu** 2017 **(cluster)**	**1,028**	3.50	0.06 (−0.20, 0.32)	2.27	0.03 (−0.25, 0.31)
	**Miguel** 2004 **(cluster)**	**9,360**	3.70	0.01 (−0.26, 0.28)	0.25	−0.75 (−1.63, 0.10)
	**Ndibazza** 2012	**715**	4.70	−0.02 (−0.12, 0.08)	5.80	0.01 (−0.17,0.19)
	**Wiria** 2013 **(cluster)**	**310**	2.00	−0.18 (−0.68, 0.32)	0.24	0.19 (−0.69, 1.06)
Studies included by Welch et al., not by Croke	**Bhoite 2012 (cluster)**	**153**	0.10	−1.30 (−4.31, 1.71)		Not included
	**Garg** 2002	**181**	4.40	0.02 (−0.13, 0.17)		Not included
	**Huong** 2007	**426**	4.10	0.13 (−0.06, 0.31)		Not included
	**Kirwan** 2009 **(weight unpublished)**	**494**	4.10	−0.12 (−0.30, 0.06)		Not included (unpublished)
	**Nga** 2009 **and** 2011	**122**	4.60	0.05 (−0.07, 0.17)		Not included
	**Olds** 1999	**91**	2.50	−0.22 (−0.63, 0.18)		Not included (unpublished)
	**Rousham** 1994 **(cluster)**	**124**	3.70	−0.11 (−0.35, 0.13)		Not included
	**Sarkar** 2002 **(SAT)**	**41**	3.30	0.38 (0.09, 0.67)		Not included, screen and treat
	**Solon** 2003	**831**	3.90	0.15 (−0.07, 0.36)		Not included (unpublished)
	**Stoltzfus** 2004 **(cluster)**	**463**	3.40	−0.08 (−0.33, 0.16)		Not included
	**Stoltzfus** 1997 **(cluster)**	**1,054**	3.10	0.16 (−0.15, 0.48)		Not included
	**Yap** 2014	**95**	3.40	0.27 (−0.01, 0.56)		Not included (screen and treat)
Studies included by Croke, not by Welch et al.	**Awasthi** 1995 (2013)	3,712 (50 clusters)	–	Not included STH + vitamin A vs. vitamin A	2.19	0.98 (0.69, 1.27)
	**Awasthi** 2001	1,672 (124 clusters)	–	Not included, STH + vitamin A vs. vitamin A	11.35	0.17 (0.04, 0.30)
	**Dossa** 2001 **(with iron)**	64	–	not included, since iron in both arms	14.09	0.00 (−0.27, 0.27)
	**Hall** 2006	2,659 (80 schools)	–	Not included, STH + vitamin A vs. vitamin A	14.09	0.05 (−0.06, 0.14)
	**Stephenson** 1989	150	–	Not included due to baseline imbalance in hookworm		Not included
	**Totals**			0.05 (0.02, 0.09) fixed effects		0.11 (0.07, 0.15) (fixed effects model
		**17,428**		0.07 (0.01, 0.13) random effects		0.13 (0.03, 0.24) random effects

Abbreviation: SAT, screen and treat; STH, soil‐transmitted helminthiasis.

^a^ Bold values are sample sizes for studies that are included in both reviews.

Our primary analysis was deworming versus placebo (without cointerventions) because it was the closest match to our NMA.

#### 5.13.1 Notes for those with different point estimates and standard errors

Awasthi and Pande (2001): We used 1 year data reported in Taylor‐Robinson, Maayan, Soares‐Weiser, Donegan, and Garner (2012) systematic review. Croke et al. (2016) used 2 year data.

Donnen et al. (1998): we used adjusted estimates reported by Donnen et al. (1998). Croke et al. (2016) used unadjusted estimates provided to the Cochrane authors.

Kruger et al. (1996): Kruger et al. (1996) randomised children to anthelminthic therapy (albendazole 400 mg once at baseline and once 5 months later) versus placebo tablets. In addition, three schools received fortified soup and two received unfortified soup. Taylor‐Robinson et al. (2015) and Croke et al. (2016) evaluated the effect of anthelminthics for the children who did not receive fortified soup to avoid the confounding effect of iron (*n* = 74). Since we considered that the fortified soup and unfortified soup were not randomised, we collapsed across the fortified and unfortified soup conditions, calculating an overall effect for all children randomised to anthelminthics versus placebo (*n* = 178).

Liu et al. (2017): We used IPD data provided by the authors. Croke et al. (2016) used published mean changes.

Miguel and Kremer (2004): We used data from the public use files for the 1st year of comparison, where we considered Group 1 as STH deworming and Groups 2 and 3 as control. It is not clear which data were used by Croke et al., but they may have had access to different datasets, or used data from the 2nd year of the study.

Ndibazza et al. (2012): We used data from IPD provided by the authors. Croke et al. used mean changes provided by the authors to Welch et al. for the 2016 review.

Wiria et al. (2013): Croke et al. (2016) used a table provided by the authors with baseline weight, and weight at 9 and 21 months, then calculated a change score and associated variance. We were able to replicate this table from the authors. However, we used IPD change score for children with complete data at baseline and 9 months to calculate a change score.

Studies not included in Welch et al. analysis: We did not include four studies in this analysis since they are a comparison of deworming combined with vitamin A versus vitamin A (Awasthi et al., 2008; Awasthi & Pande, 2001; Hall et al., 2006) or STH deworming + iron versus iron (Dossa et al., 2001). Because we used a NMA approach, these studies are included in the node for STH + micronutrients or iron compared to micronutrients or iron. In a sensitivity analysis, we included these studies to assess the influence on our results, and our random effects meta‐analysis was 0.10 kg (95% CI: 0.03, 0.17). Note: these studies are included in the NMA‐IPD presented in this paper if they had baseline infection intensity and provided data (that is, Hall et al. (2006) was included).

## 6 DISCUSSION

### 6.1 Summary of main results

This IPD NMA and systematic review reinforces findings from previous meta‐analyses and the 2017 WHO guidelines on mass deworming that STH deworming alone is insufficient to improve population level child health and cognitive outcomes based on moderate certainty evidence. When we add data obtained for this IPD analysis from unpublished results on weight gain to studies included in our prior meta‐analysis, the overall effect across 25 studies of deworming compared to placebo is 0.07 kg (−0.01, 0.13). This effect size is comparable to other published systematic reviews and meta‐analyses.

A central issue in deworming debates has been the difficulty of detecting effects when the majority of the population has light or no detectable infection. Our review is unique in its ability to assess effect modification using IPD. Effect modification analyses across intensity of infection, using WHO cutoffs, suggest that deworming may slightly increase weight in children with moderate to heavy intensity infections of *A. lumbricoides* or *T. trichiura* (very low certainty).

At the population level, for weight gain, we found little effect for STH deworming versus placebo with our NMA IPD results (0.01 kg; 95% CI: −0.08, 0.11) with moderate quality evidence, and results for all other comparisons were similar. For height, we found little effect on height (0.09 cm; 95% CI: −0.08, 0.27) with moderate quality evidence and similar effects across all comparisons. For haemoglobin, we found little effect of deworming for STH (0.32, 95% CI: −0.63, 1.26) (low certainty). Deworming with praziquantel resulted in an increase in haemoglobin compared to placebo of 1.85 g/L (95% CI: 0.53, 3.18). Similarly, deworming with praziquantel combined with iron or micronutrients increased haemoglobin (2.72 g/L, (95% CI: 1.05, 4.40), iron or micronutrients with STH deworming increased haemoglobin (1.98 g/L, (95% CI: 0.74, 3.21) compared to placebo (low certainty evidence). For cognition, studies reported no effects of any types of deworming on short term attention measures such as digit forward or processing speed or measures of child development.

Subgroup analysis were considered at very low certainty due to imprecision of results. There were no statistically significant subgroup effects across age, sex, infection intensity for any type of STH infection using median distribution of intensity, BMI for age, height for age or anaemia (moderate certainty). For subgroup analysis using WHO cutoffs and direct evidence, children with moderate to heavy *A. lumbricoides* or hookworm infection, deworming may slightly increase weight but not height or haemoglobin compared to children with no detected infection or light infection intensity (very low certainty evidence).

These findings were robust to sensitivity analyses across risk of bias and effectiveness of studies at reducing infection prevalence as well as differences in the model structure, adjustment for covariates, risk of bias and the use of multiple imputation for missing data.

Clinical importance of weight and height for STH deworming needs to be put in the context of the children in these studies, where 33% of children were stunted, 54% were anaemic, 41% were infected with hookworm, 48% with *A. lumbricoides*, 53% with trichuris and 73% were over 5 years of age. According to WHO growth standards, weight gain for children aged 7 years is approximately 2 kg in 12 months. Therefore, the 95% CI observed in our analysis of −0.08 to 0.11 kg for STH versus placebo is equivalent to −4% to +5.5% relative to the expected weight gain over this time period for these children. This is much smaller than effects of other nutritional programmes such as schoolfeeding which increases weight gain by about 0.39 kg annually (Kristjansson et al., 2007). We are moderately certain that further research will not change this estimate. Uncertainty arises since we were unable to obtain data from all published trials.

Clinical importance of haemoglobin effects need also to be considered in light of the average haemoglobin level of these children, and the settings in which they live. Almost half (46%) of our sample was anaemic, defined as haemoglobin below 115 g/L. STH deworming alone compared to placebo had small effects on haemoglobin (0.32 g/L) but STH deworming combined with micronutrients or iron compared to placebo had larger effects (1.98 g/L). as a comparison, iron supplementation alone for children <12 years of age was found to increase haemoglobin by approximately 5 g/L (De‐Regil et al., 2011).

We were successful in retrieving 14% (2/14 studies) for deworming for schistosomiasis. As a result, we were unable to conduct our planned analysis of deworming for schistosomiasis.

### 6.2 Overall completeness and applicability of evidence

For STH deworming, we received data for 19 studies with 31,945 participants of an eligible 41 studies with 40,132 participants for studies of STH deworming. Fifteen of the 19 studies were published in the last 15 years.

We had sufficient participants in each level of our planned effect modifier analyses to run all of our planned effect modification analyses using the base case evidence network. Although there were no statistically significant differences across infection intensity levels, these were limited due to the paucity of children with moderate to heavy infection intensity in our sample (<13%). Furthermore, the upper CIs included potentially important effects of up to 460 g for weight and 7 g/L for haemoglobin. In direct evidence comparisons using WHO cutoffs, we also did not find statistically significant interaction across intensity of infection, however, there were larger effects on weight gain for children with moderate or heavy intensity infections of *A. lumbricoides* or *T. trichuria*. For prevalence of *A. lumbricoides* and hookworm, we also assessed whether there was a relationship between prevalence and effects on weight using meta‐regression for aggregate data for all studies with weight data for STH deworming versus placebo. These analyses did not show a relationship with *A. lumbricoides* prevalence at the study level. There was an association of higher hookworm prevalence with effect on weight. These meta‐regressions must be interpreted with caution since they are using data at the aggregate level (Debray et al., 2018).

We conducted an extensive search of electronic databases, with advice from the Campbell Collaboration International Development Group information scientist. We screened 16,613 articles and updated this search to March 27, 2018. We report the systematic review according to the reporting guidelines for IPD meta‐analysis (PRISMA‐IPD) and network‐meta‐analysis (PRISMA NMA).

We published and followed an a priori protocol (Welch et al., 2018). Our systematic review and IPD analysis was approved by the Research Ethics Boards at SickKids and Bruyere Research Institute. We developed a data sharing agreement that was signed by all studies that contributed data. Study authors were invited to join the Investigators' Collaborative, participate in meetings and contribute to the final report. Our process to developing the evidence network was driven by consultation with our expert Advisory board which included statistical, parasitology and nutrition expertise. We tested our assumptions, model structure and statistical methods using sensitivity analyses.

The studies were conducted in a range of low and middle income countries in settings with predominantly poor sanitation with a range of prevalence of STH in children aged from 6 months to 17 years. The prevalence of *A. lumbricoides* infections in our base case sample (14 studies with sufficient data for multiple imputation) was 52% (range, 9–93%), hookworm 45% (range, 1–94%) and *T. trichiura* 52% (range, 9–96%). Less than 15% of children had an infection intensity considered moderate or heavy for *A. lumbricoides*, *T. trichiura* or hookworm, according to the WHO criteria in our sample. Our dataset included 2,448 children <5 years of age in our main models (18% of the sample).

Our data suggest that there is publication bias in the deworming literature with failure to report growth data since we obtained weight and height data from eight studies which had not previously reported these (Beasley et al., 1999; Beasley, 1995; Friis et al., 2003; Ebenezer et al., 2013; Kirwan et al., 2009; Le Huong et al., 2007; Rohner et al., 2010; Solon et al., 2003). We also report cognition data that was not previously published from one study (Rohner et al., 2010). Given our findings of selective outcome reporting, it is still possible that there are additional older studies with negative findings.

We compared the effect sizes observed in the studies that we retrieved to those which were excluded (due to missing baseline infection intensity) or which we were unable to obtain from the trial authors (due to lost datasets, administrative hurdles or nonresponse from the authors). We found that the test for interaction for subgroup differences was not statistically significant for weight, height or haemoglobin, but the effect on weight was higher in the studies which were not obtained, which were mostly older studies.

For schistosomiasis deworming, we received only two of 14 eligible studies. We decided that meta‐analysis of these two studies would be misleading and did not pursue IPD meta‐analysis for schistosomiasis deworming. We did include nodes in our evidence network for combinations of schistosomiasis deworming and STH deworming, but these had relatively fewer studies and participants.

Small amounts of calories were provided in three studies in the form of unfortified or fortified biscuits (Nga et al., 2009), noodles (Le Huong et al., 2007) or beverage (Solon et al., 2003). In each of these studies, the comparator groups received the unfortified food or beverage. We did not identify any studies that looked at providing substantive meals or snacks with deworming. Thus, we cannot draw conclusions on the effects of deworming when combined with feeding programmes in comparison to not providing feeding.

### 6.3 Quality of the evidence

We included only RCTs. About 40% of trials did not provide enough information to assess adequacy of randomisation and allocation concealment. We considered the included studies were at overall low risk of bias. The quality of evidence as assessed using the GRADE framework was moderate across all outcomes and comparisons for the main effects. Quality of evidence was downgraded because of uncertainty about selective reporting bias across the evidence base, and the fact that we were not able to obtain data from all eligible studies. Subgroup effect analyses were judged at very low certainty due to imprecision and inability to obtain all eligible studies.

Sensitivity analyses across adequacy of allocation concealment were congruent with our main findings for weight, height and haemoglobin for all comparisons.

### 6.4 Limitations and potential biases in the review process

One limitation of this review is that we did not receive data from all eligible studies. We compared published results of the studies received for STH deworming versus placebo with the studies that were not received and those that were not eligible to assess the potential influence of these missing studies on our findings. The test for interaction was not statistically significant but the effect on weight gain overall was larger in studies that were not received, which limits the ability of this analysis to assess the overall, population level effects. However, this should not affect the effect modification analyses since these are based on individual level covariates. There was no trend in effect size or direction across the year of publication for weight, height or haemoglobin.

The assumptions of transitivity and consistency were assessed and considered plausible by assessing distribution of effect modifiers, assessing within comparison heterogeneity in direct evidence and by comparing direct and indirect evidence.

Another limitation is that different diagnostic tools with different measuring properties including Kato‐Katz, polymerase chain reaction (PCR) and other techniques, were used for assessing infection intensity across the studies and may lead to measurement error. Only one study used PCR, and we used its infection intensity estimates in analyses with other studies, recognising that there may be differences in sensitivity of these tests.

Cognitive outcomes are measured using diverse tools and some are translated for use in these studies. For this reason we presented each cognitive outcome for each study separately without combining them in a meta‐analysis. This limits the ability to combine results across studies thus these analyses are under‐powered for cognitive outcomes.

We were unable to assess effect modification for infection intensity using the WHO cutoffs for moderate or heavy intensity using our NMA model because <15% of children in our sample met criteria for being moderately to heavily infected, thus the models failed to converge when we used the WHO cutoffs for moderate and heavy intensity infection. To further investigate the importance of infection intensity, we conducted subgroup analysis using the WHO cutoffs for each infection type (none detected, light and moderate/heavy) for the direct evidence of STH deworming versus placebo. The test for interaction for subgroup effects across infection intensity for STH deworming versus placebo for weight, height or haemoglobin was not statistically significant. However, the effect of deworming was higher for weight gain for children with moderate/heavy infection of *A. lumbricoides* and *T. trichuria*, compared to children with light intensity infections or no detected infection. These subgroup analyses were considered very low certainty evidence due to imprecision.

The study durations were short with a median duration of 12 months (ranging from 4 to 45 months) and this may have limited our ability to detect changes in height or weight gain. However, since two of the earlier studies mentioned previously with large effects on weight (Stephenson et al., 1989, 1993) were only 6 and 8 months in duration, we consider that the study durations of these studies was sufficient to assess differences in weight gain. It is unlikely that these study durations are sufficient to assess differences in linear growth. Single dose trials of short duration may not be able to detect positive effects due to high re‐infection rates in endemic areas.

In our collapsed model, we collapsed across frequency of deworming which limits our ability to assess whether high frequency STH deworming is more effective than regular frequency deworming. As described above, our preliminary models with frequency of administration as separate nodes did not show differences in effects on weight, height or haemoglobin between high frequency and regular frequency deworming.

Two studies in Kenya have shown large effects on weight gain of 1 kg or more (Stephenson et al., 1989, 1993). The reason for these large effects is unclear. Analysis of heterogeneity led us to exclude the Stephenson et al. (1989) study due to baseline imbalance in a prior systematic review (Welch et al., 2016). The conditions in which those two trials were carried out may have been different from other trials, including characteristics such as intensity of infection, sanitation, and participant and investigator adherence to protocols. However, 25 other studies are available on STH versus placebo, and when all are combined, the overall effect in our analyses is 70 g.

The older studies of deworming suggested stronger effects on nutrition and other health outcomes than we have found in our analysis. Given that stunting is associated with adverse health and cognitive outcomes that implied (since deworming drugs are inexpensive) that deworming is cost‐effective. However, our study would cast doubt on this, since at moderate levels of infection, we could not discern significant impacts on key nutrition outcomes such as stunting and wasting. Our systematic review cannot predict outcomes and cost‐effectiveness for chemoprophylaxis where infection is severe, since we had <2% of our sample with heavy intensity infections.

Our study did not look at school attendance which has been used for previous cost‐effectiveness analysis of deworming. There has been an intense debate on this topic where an independent replication identified smaller benefits than previously thought (Aiken, Davey, Hargreaves, & Hayes, 2015; Hargreaves, Aiken, Davey, & Hayes, 2015; Hicks, Kremer, & Miguel, 2015). Also, our prior systematic review found an average effect on school attendance of 1% (95% CI: −1, 3%) (Welch et al., 2016). We also identified problems with the methods of measuring school attendance in these studies. The implication is that the cost‐effectiveness/cost benefit of deworming on the basis of school attendance is not proven.

The exclusion of studies with <100 participants may lead to small study bias. However, only three studies had <100 participants; including them would not affect the main analyses or effect modification analyses.

### 6.5 Agreements and disagreements with other studies or reviews

A Cochrane review (Taylor‐Robinson et al., 2015) and Campbell review (Welch et al., 2016) on mass deworming for children both concluded there was little to no effect on weight and height for STH deworming. The effects observed in Taylor‐Robinson et al. (2015) were a mean difference of 0.08 kg (95% CI: −0.11, 0.27) on weight, a mean difference of 0.02 cm (95% CI: −0.14, 0.17) on height and a mean difference of 0.02 g/dL, 95% CI: −0.08, 0.04) on haemoglobin for regular treatment, and little to no effect on formal tests of cognition (Taylor‐Robinson et al., 2015). In Welch et al. (2016), the effects of Albendazole twice per year were 0.09 kg (9%CI: −0.04, 0.2), 0.07 cm (95% CI: −0.1, 0.24 cm), short term cognition −0.23 points on a 100 point scale (95% CI: −0.6, 0.14). Haemoglobin effects were not combined across studies in Welch et al., but the individual study results are consistent with our finding that there are robust effects on haemoglobin only when iron, micronutrients or praziquantel are combined with STH deworming.

Our findings for STH deworming versus placebo for height, cognition and haemoglobin are similar to these two prior reviews. Our IPD‐NMA effect on weight gain of 0.01 kg (95% CI: −0.08, 0.11) is lower than these reviews, and is likely due to not being able to retrieve data from all eligible studies. Our meta‐regression of year of publication and weight gain did not show a statistically significant effect of year of publication, but this must be interpreted with caution since metaregression suffers from low power and was based on aggregate data. The smaller effect seen in our analysis may be related to publication bias in the previous reviews since we obtained unpublished data which is known to be associated with negative findings (defined as smaller effects or nonstatistically significant; Hopewell, Loudon, Clarke, Oxman, & Dickersin, 2009) and that we did not receive data from all available studies.

Our finding on weight gain with our IPD‐NMA of 0.01 kg is considerably smaller than in the meta‐analysis by Croke et al. (2016) on weight gain (http://www.nber.org/papers/w22382.pdf), which found an average overall effect on weight gain of 0.134 kg (95% CI: 0.031, 0.236). When comparing our analyses of direct evidence from all studies with STH deworming versus placebo (including both studies for which we received IPD and studies which did not contribute IPD), we also found a smaller effect size than Croke et al. of 0.07 kg (95% CI: 0.01, 0.13) on weight with random effects. Our finding that there were no subgroup effects across infection intensity or association of effect size with prevalence do not agree with Croke et al.'s findings that the effect on weight was higher for studies with >20% prevalence (0.148 kg, 95% CI: 0.039, 0.2225558). Much of the difference in our findings across prevalence and intensity of infection may be due to the fact that NMA‐IPD has better power to detect subgroup differences than aggregate level subgroup analyses or meta‐regression (Dagne et al., 2016).

## 7 AUTHORS' CONCLUSIONS

### 7.1 Implications for policy

The policy implications are that deworming alone is insufficient to achieve improvements in population‐level growth, nutritional status and cognition. Based on the totality of evidence from three prior systematic reviews and new data from IPD previously unpublished, average effects of mass deworming on child nutritional status and cognition are small at the population level (moderate certainty). Effects are higher for children with moderate to heavy intensity infections thus mass deworming may be beneficial in areas with heavy intensity infections. In areas with predominantly lighter infections, effects are smaller thus policymakers and programmers need to explore other policy options to improve child health and nutrition in these areas.

### 7.2 Implications for research

IPD analyses such as this have greater power to investigate effect modifiers, but are currently limited by the time and resources needed to seek data from all eligible studies and the limited availability of such data. There is an urgent need for open data from all research studies. Our analyses were limited by obtaining only 46% of eligible studies, mostly conducted in the last 15 years, which could be mitigated by having all data from prospectively registered trials available in open data repositories, as called for by the Alltrials campaign.

The quality of evidence is rated as moderate for our findings, mainly due to the possibility of selective reporting and publication bias in the body of literature. Further research to obtain additional unpublished data on growth and cognition could change our findings. For schistosomiasis deworming, we were unable to obtain the majority of studies, thus we did not carry out these analyses.

Further short‐term studies of STH deworming in lightly infected populations are not likely to change the certainty or sizes of effects observed in this systematic review or in other systematic reviews of deworming.

Ideally, in the design of studies, duplicate methods to measure exposure and outcome in a reliable way would be important. For example, future studies could use more sensitive diagnostic tools (e.g., PCR). Also, for cognition, proper cultural translations and validation of measurement tools are important.

## ROLES AND RESPONSIBILITIES

V. W., E. G., A. H., A. R., and G. A. W. had full access to the data and take responsibility for the accuracy of data analyses. Concept and design: Z. B., M. G., S. C., V. W., G. A. W., and P. A.: Horton, Black, Tugwell, Hollingsworth, Holland. Acquisition of data: V. W., E. G. and C. M. Acquisition of data: V. W., E. G. and C. M. Drafting of manuscript: V. W. and E. G. Critical revision of content: all authors. Statistical analysis: V. W., A. H. and A. R., O. D. Obtained funding: Z. B., M. G., G. A. W., and V. W.

## SOURCES OF SUPPORT

This review is funded by the Bill and Melinda Gates Foundation (funding reference number: OPP1140742).

## DECLARATIONS OF INTEREST

Michelle Gaffey, Robert Black, Deidre Hollingsworth, Sue Horton, Paul Arora, Alison Riddle, Rehana Salam, Simon Cousens, Omar Dewidar have no conflict of interest, financial or otherwise that may influence judgments made in this review.

Celia Holland is a co‐author and principal investigator on a randomised trial of deworming: Kirwan et al. 2009.

Vivian Welch, Elizabeth Ghogomu, Alomgir Hossain, Zulfi Bhutta, Peter Tugwell and George Wells are authors of the Campbell systematic review and NMA of mass deworming for children (Welch et al. 2016).

Vivian Welch is editor‐in‐chief of the Campbell Collaboration.

## PLANS FOR UPDATING THE REVIEW

This review will be updated if funds become available.

## Supporting information

Supplementary information

## Footnotes

1 Anaemia cutoffs were sex‐ and age‐specific as per the WHO's *Haemoglobin concentrations for the diagnosis of anaemia and assessment of severity* (World Health Organization, 2013).
