# Supplementary material for: Mass deworming for improving health and cognition of children in endemic helminth areas: A systematic review and individual participant data network meta‐analysis
Source: Campbell Syst Rev. 2019 Nov 20;15(4):e1058. doi: 10.1002/cl2.1058 (PMC8356492; doi:10.1002/cl2.1058)
Supplement: Supplementary file 1 — Supplementary information [file CL2-15-e1058-s001.docx]

# Additional Figures and tables

Additional figures 1: *A Lumbricoides*  frequency, across all studies


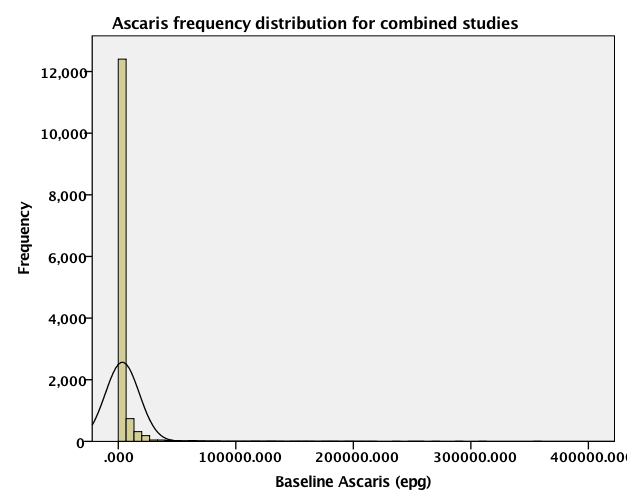


Additional figures 2: *A Lumbricoides*  distribution for each study


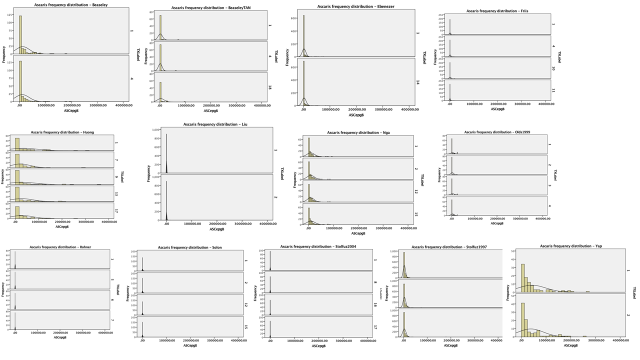


Additional figures 3: Hookworm frequency across all studies


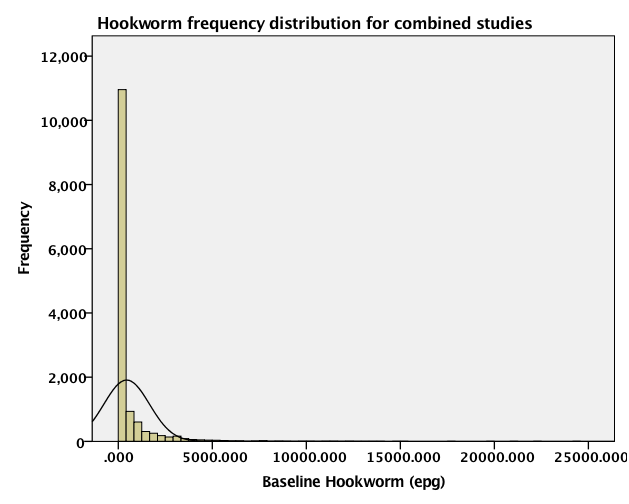


Additional figures 4: Hookworm frequency for each study


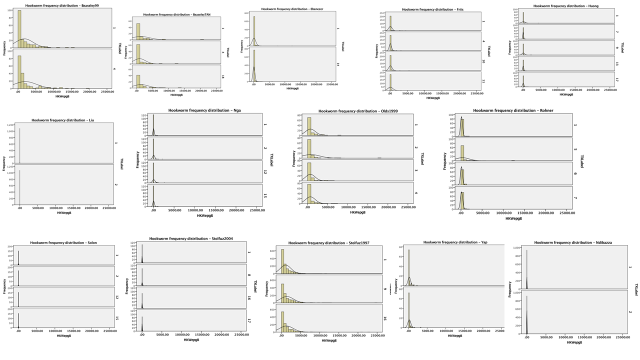


Additional figures 5: *T. Trichiura*frequency across all studies


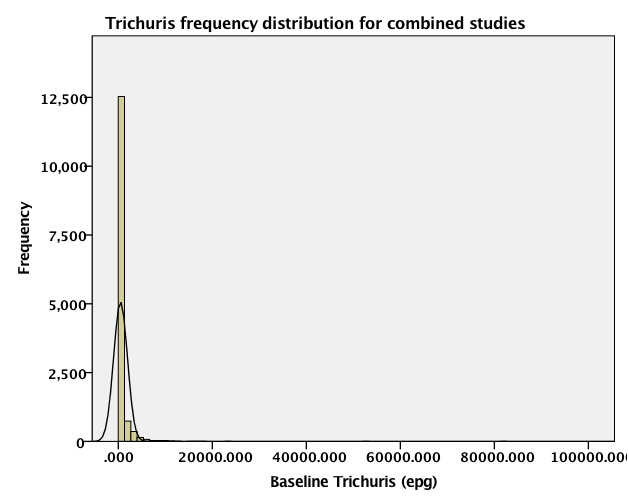


Additional figures 6: *T. Trichiura*frequency for each study


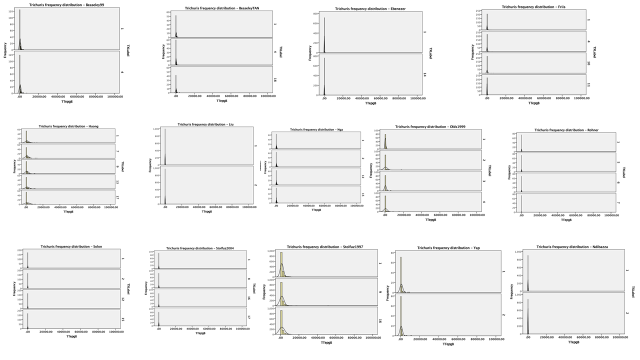


Additional figures 7: BMI for age frequency across all studies


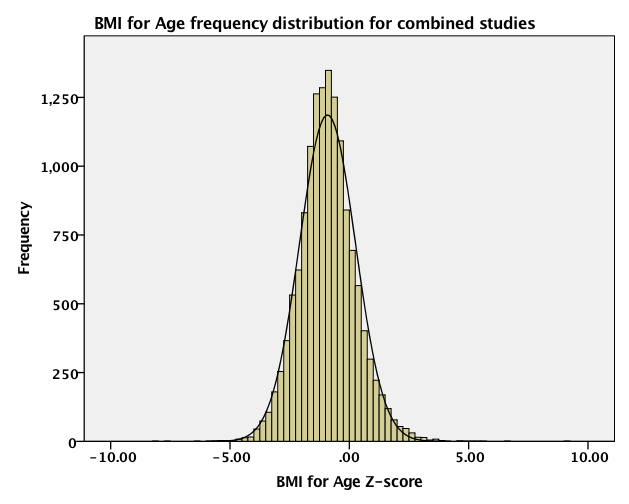


Additional figures 8: BMI for age for each study distribution


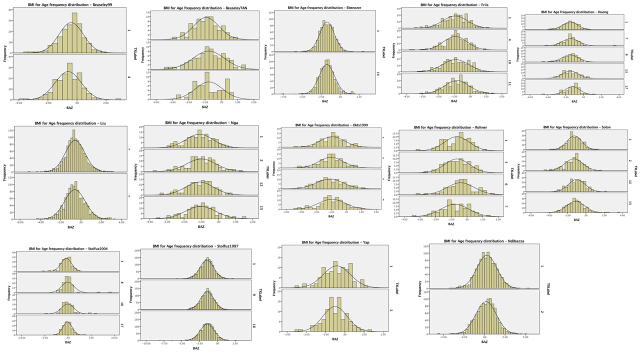


Additional figures 9: Height for age frequency distribution across all studies


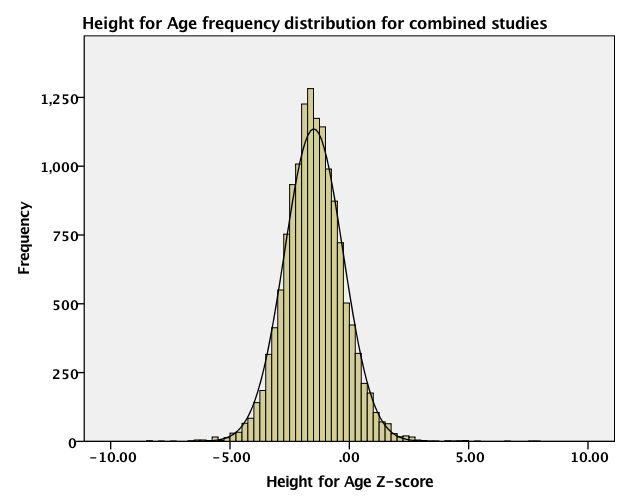


Additional figures 10: Height for age distribution across each study


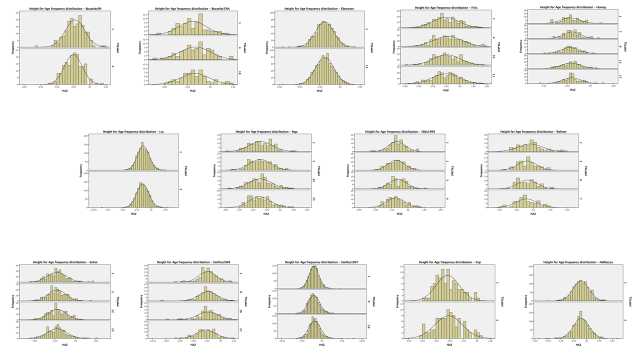


Additional figures 11: Haemoglobin frequency distribution across all studies


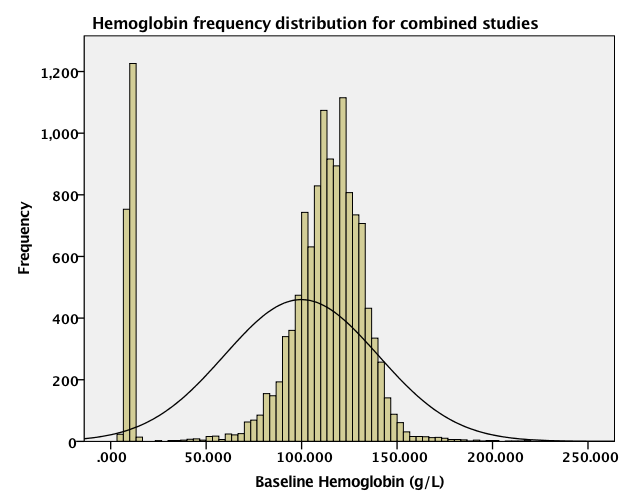


Additional figures 12: Haemoglobin frequency distribution for each study


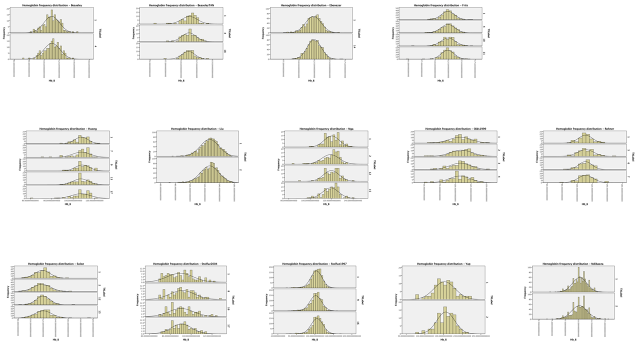


Additional figures 13: Weight gain (kg), BMI for age as effect modifier

Additional figures 14: Height gain, with BMI for age as effect modifier

Additional figures 15: Haemoglobin, with BMI for age as effect modifier


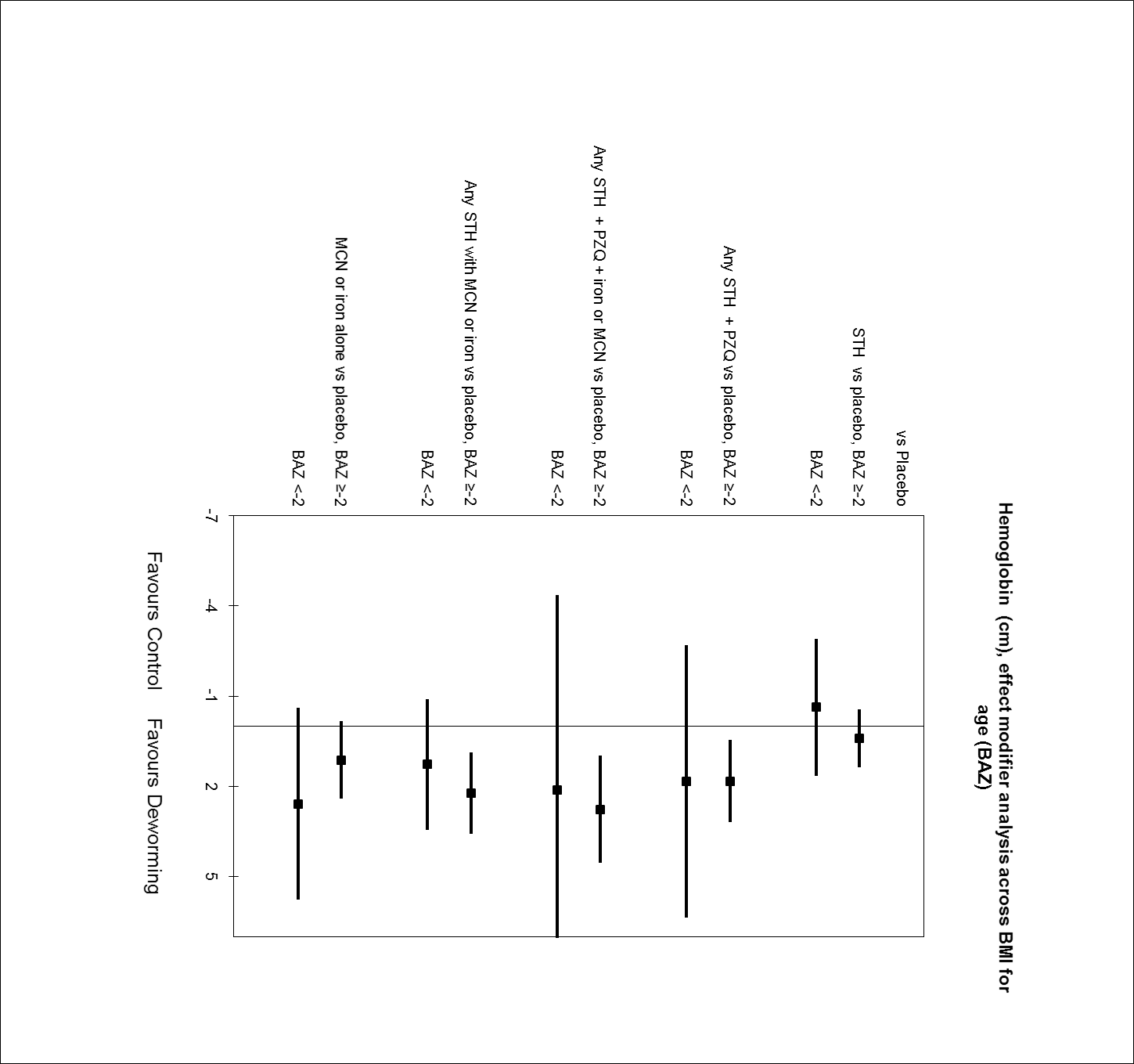


Additional figures 16: Weight gain (kg) with height for age as effect modifier


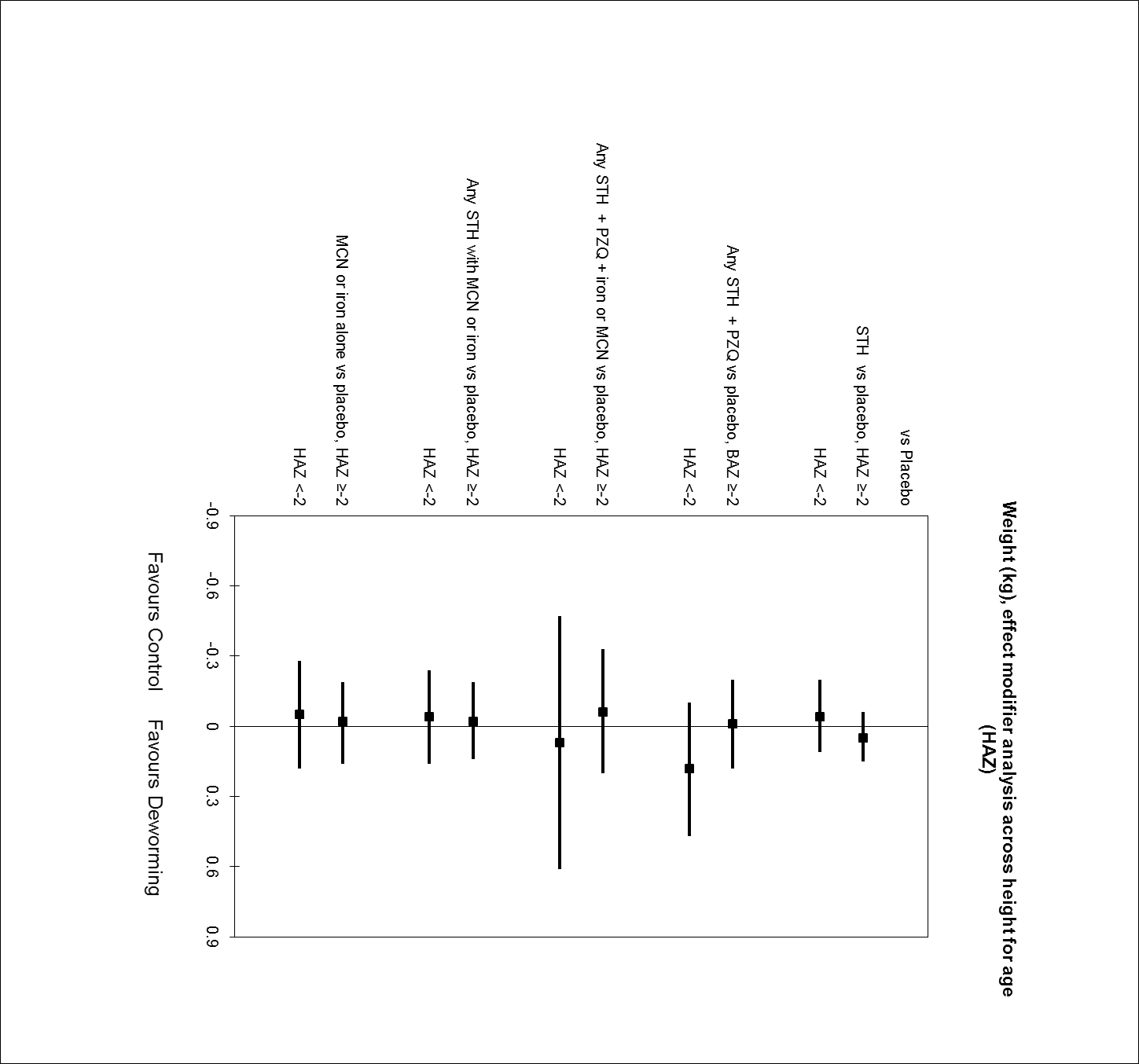


Additional figures 17: Height gain (cm), with HAZ as effect modifier


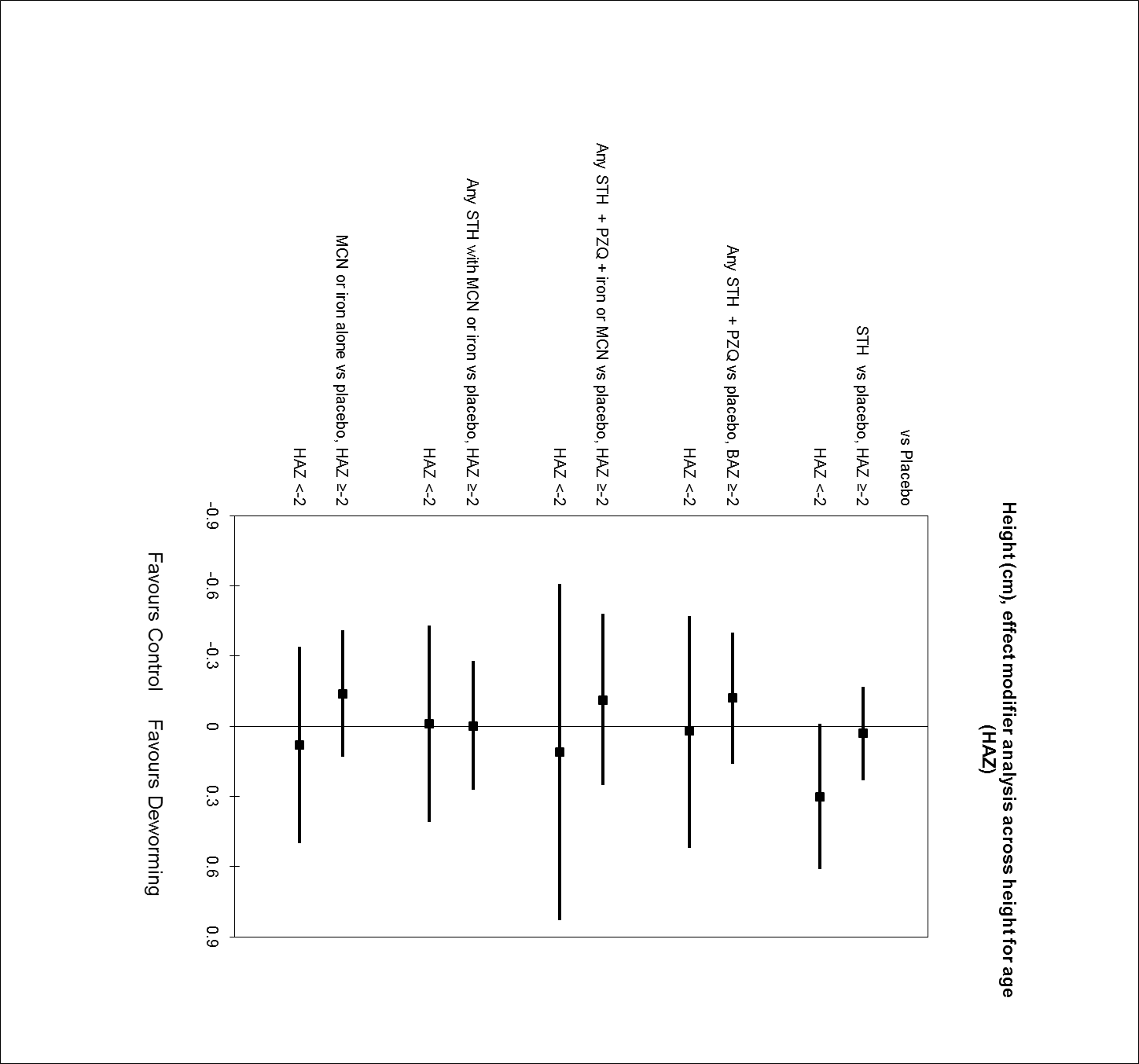


Additional figures 18: Change in haemoglobin (g/L), with HAZ as effect modifier


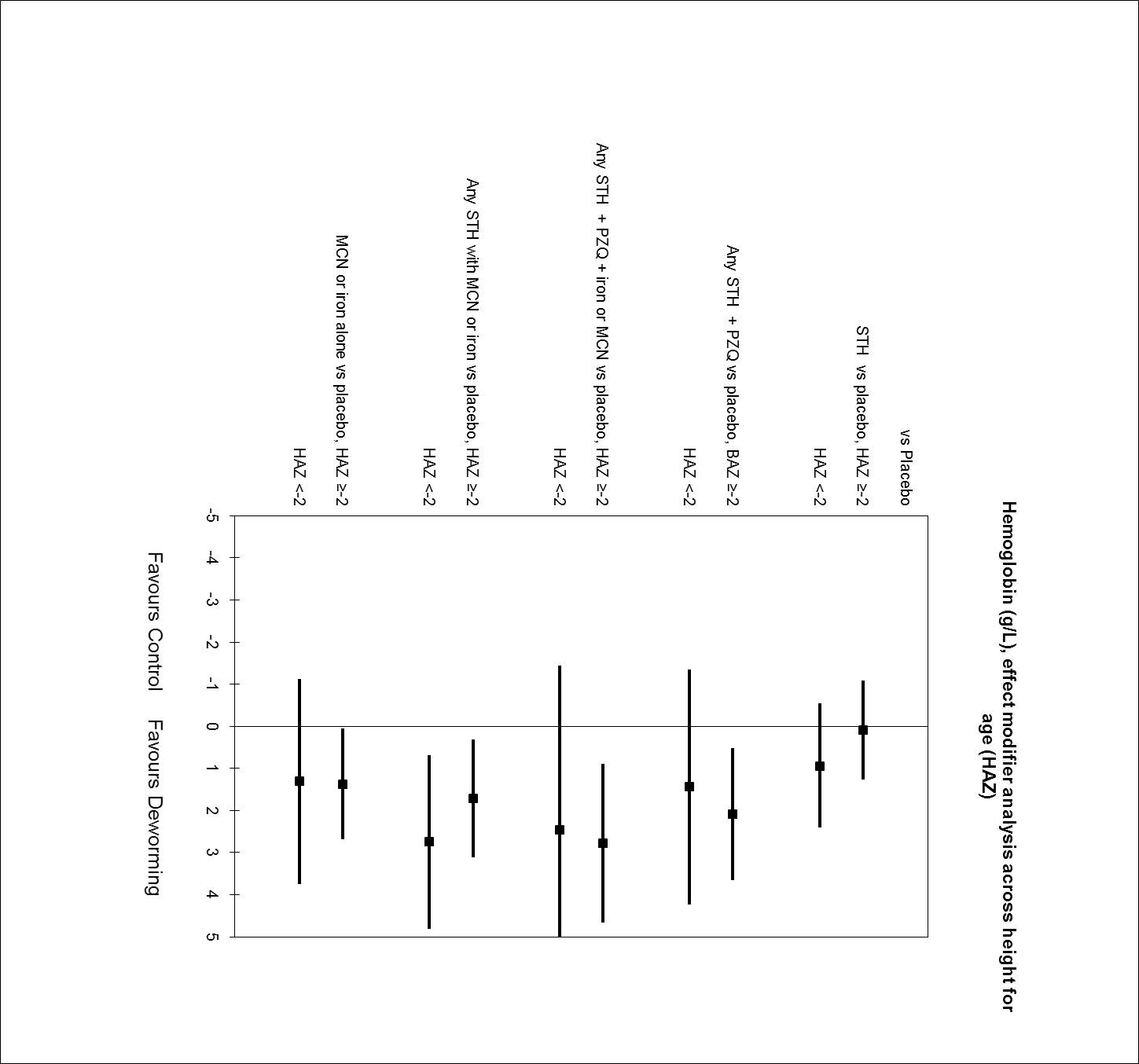


Additional figures 19: Weight gain (kg), with sex as effect modifier


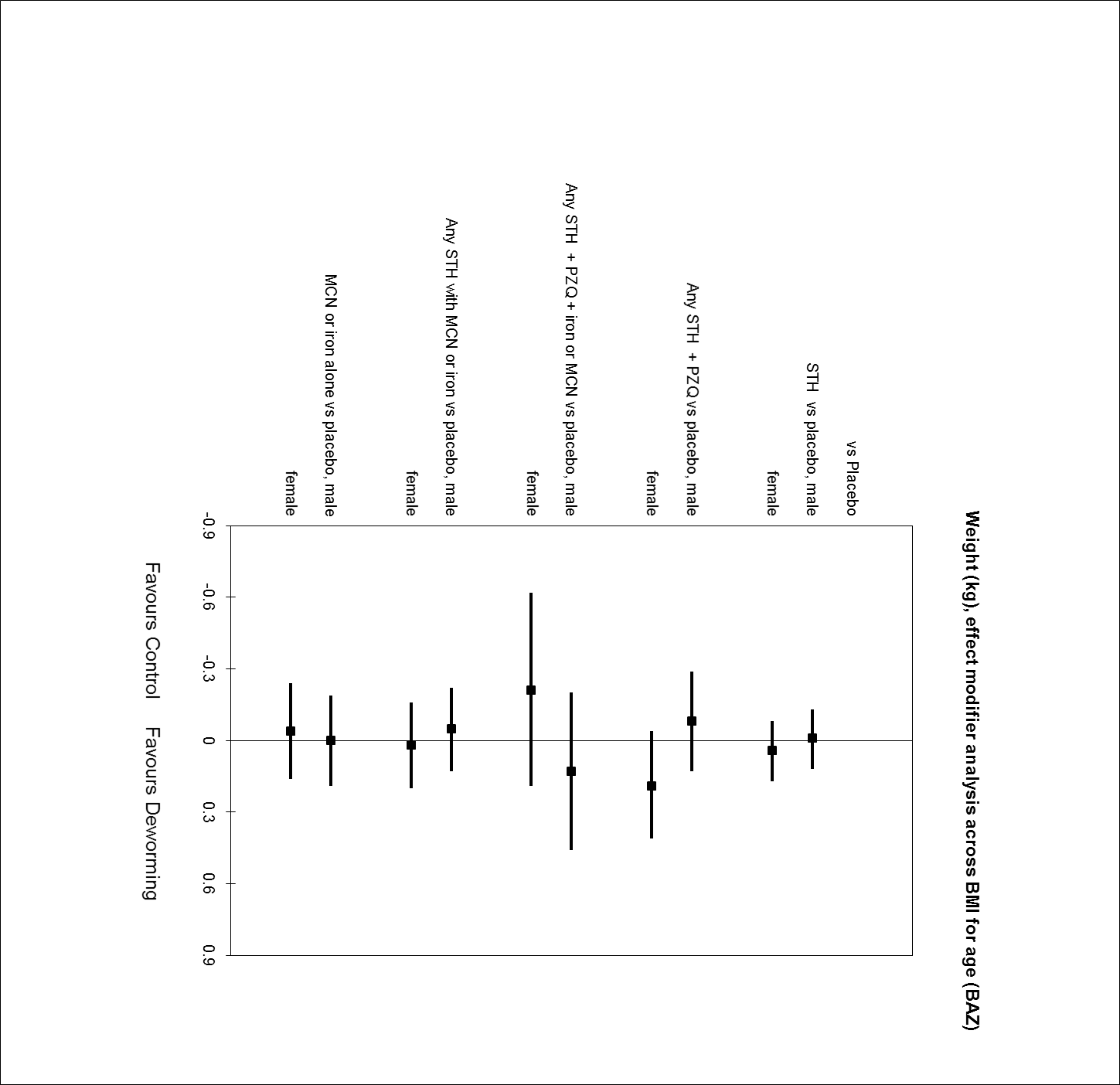


Additional figures 20: Height gain (cm) with sex as effect modifier


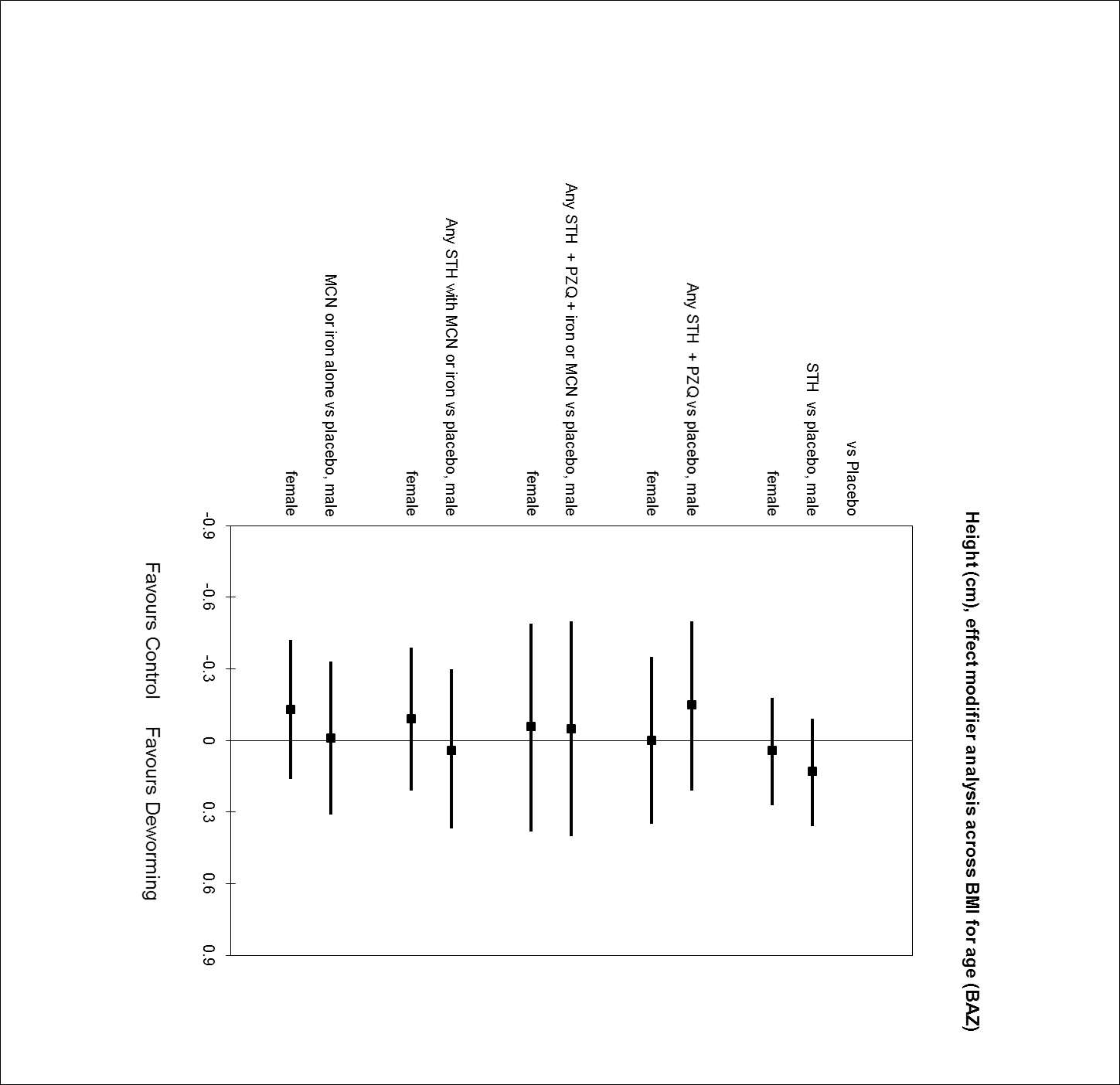


Additional figures 21: Change in haemoglobin (g/L), with sex as effect modifier


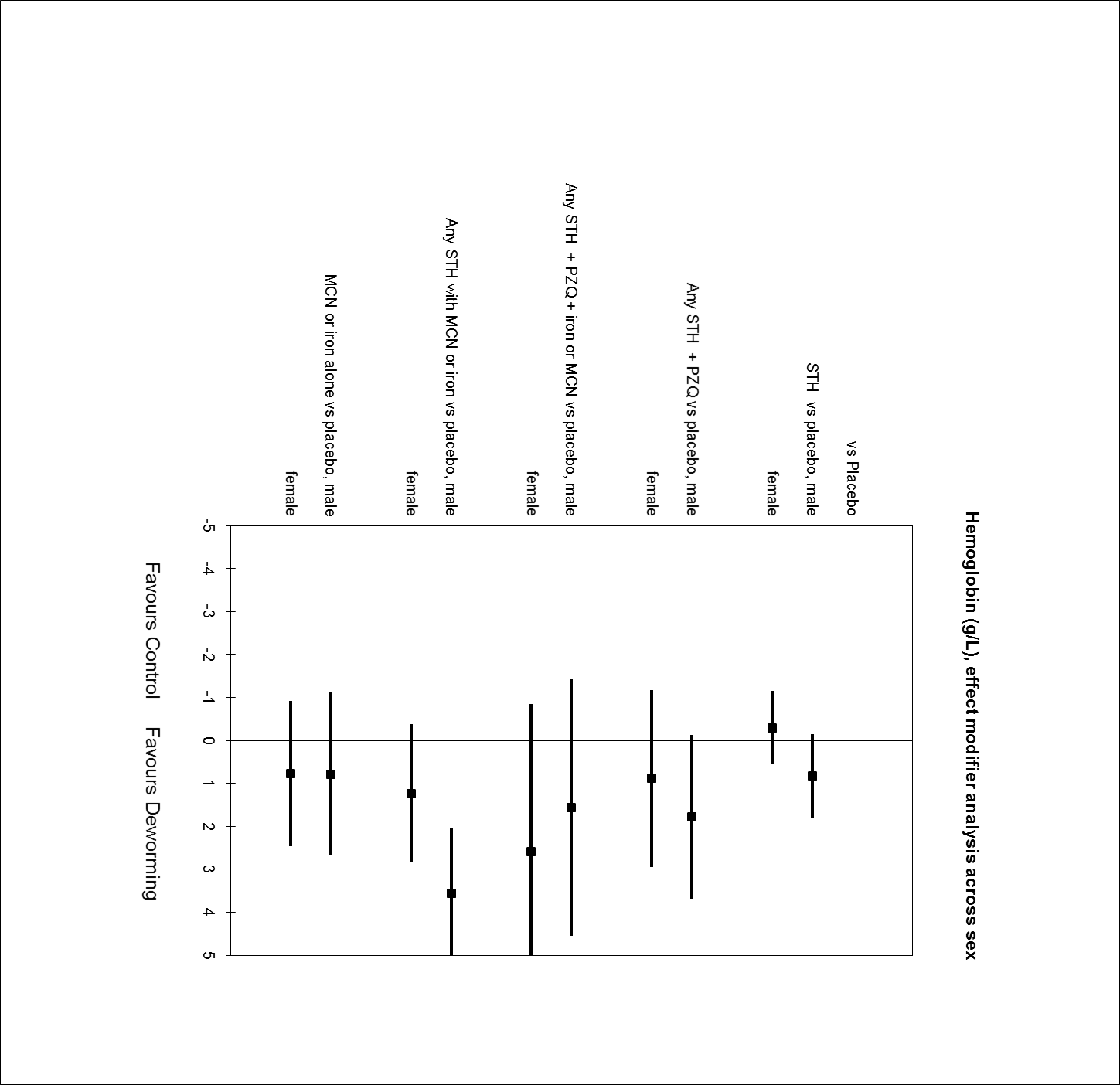


Additional figures 22: Weight gain (kg), Age as effect modifier (for <5 years)


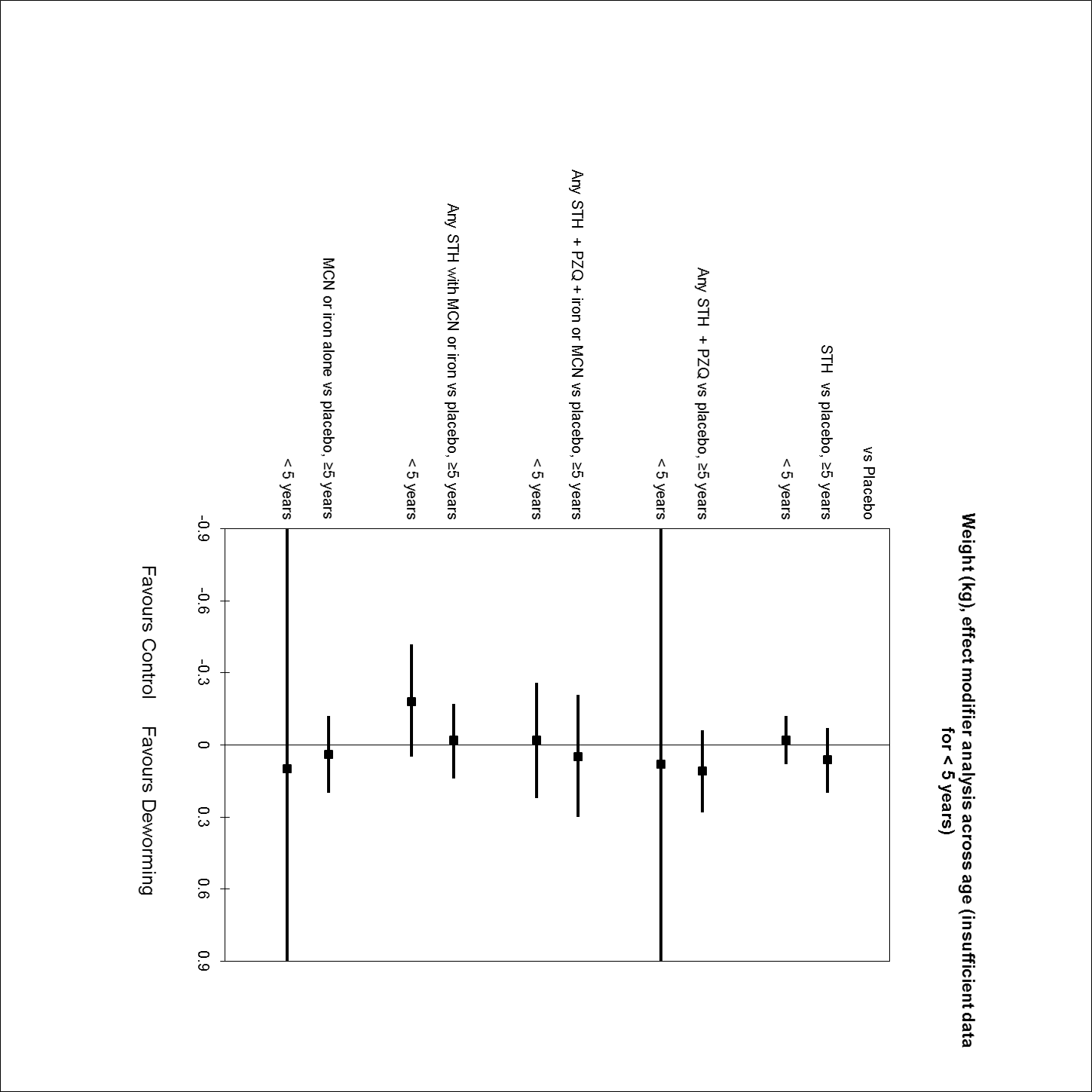


Additional figures 23: Height gain (cm), with age as effect modifier (for <5 years)

Additional figures 24: Change in haemoglobin (g/L), with age as effect modifier (for < 5 years)


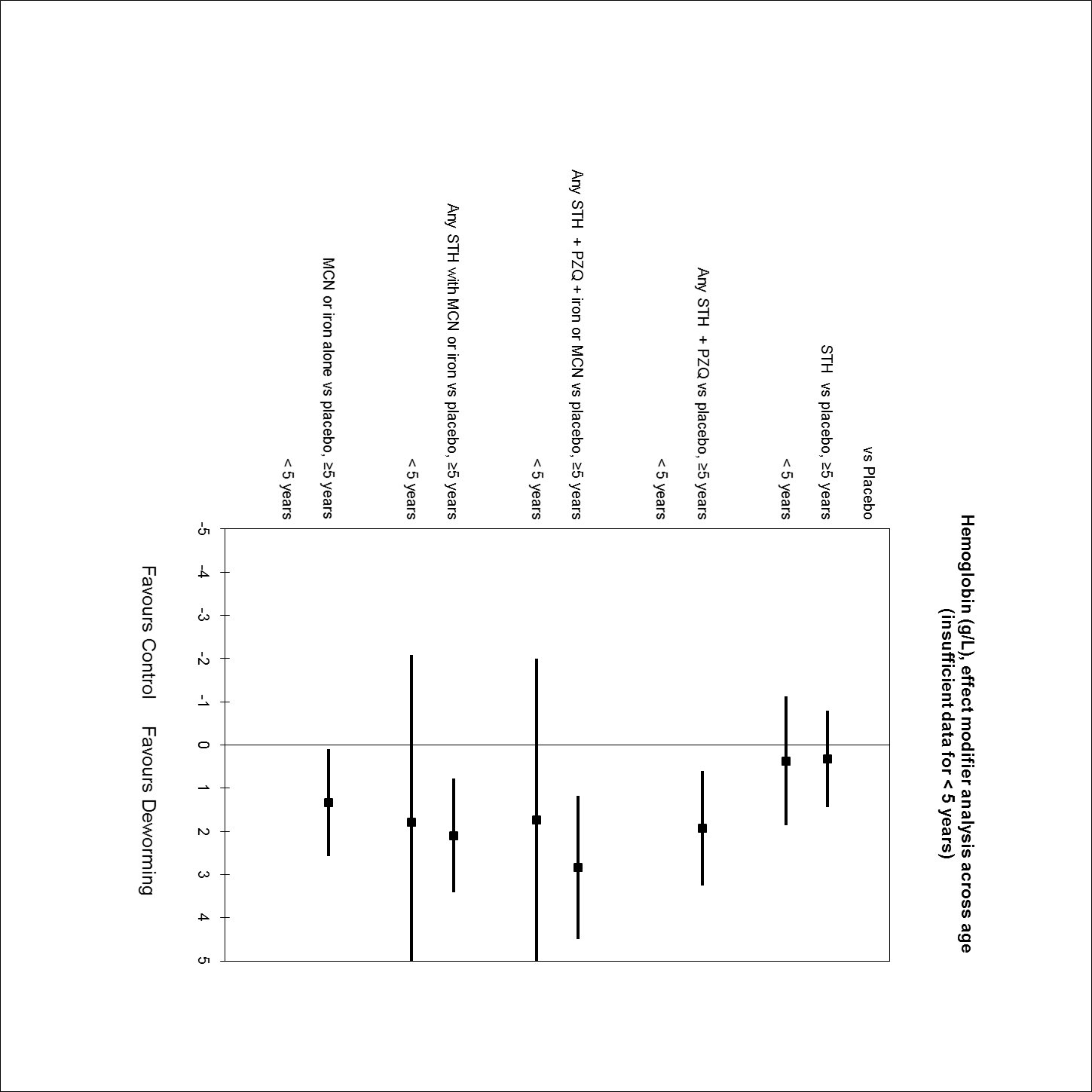


Additional figures 25: Direct evidence, Weight gain (kg), with *A Lumbricoides*  infection intensity using WHO cut-offs as effect modifier


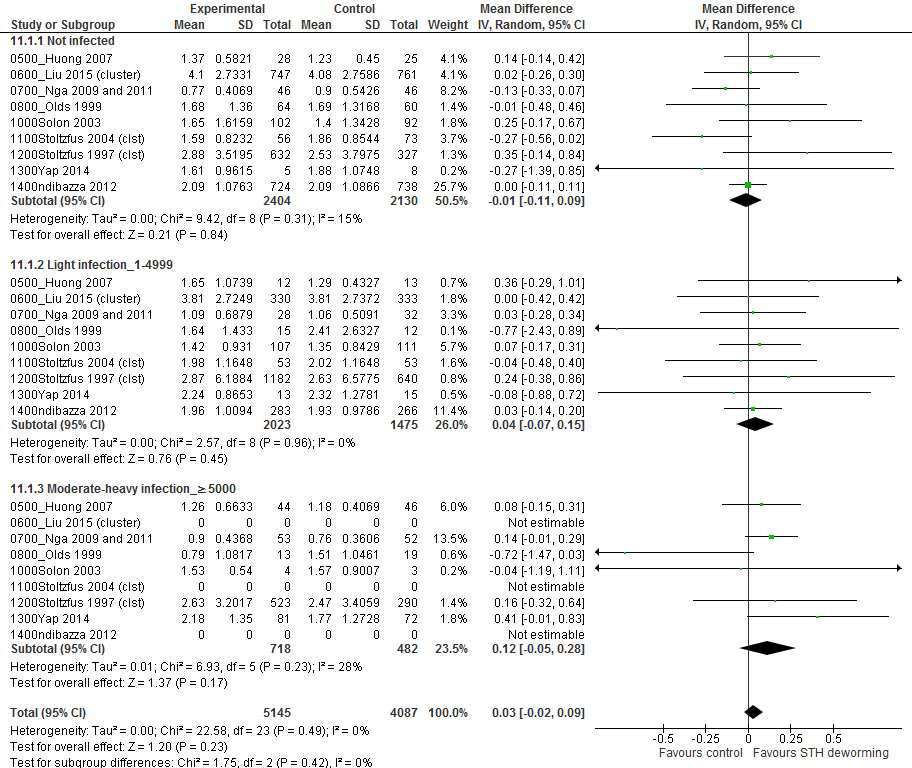


Additional figures 26: Height gain (cm), direct evidence, with *A Lumbricoides*  infection intensity using WHO cut-offs as effect modifier

**
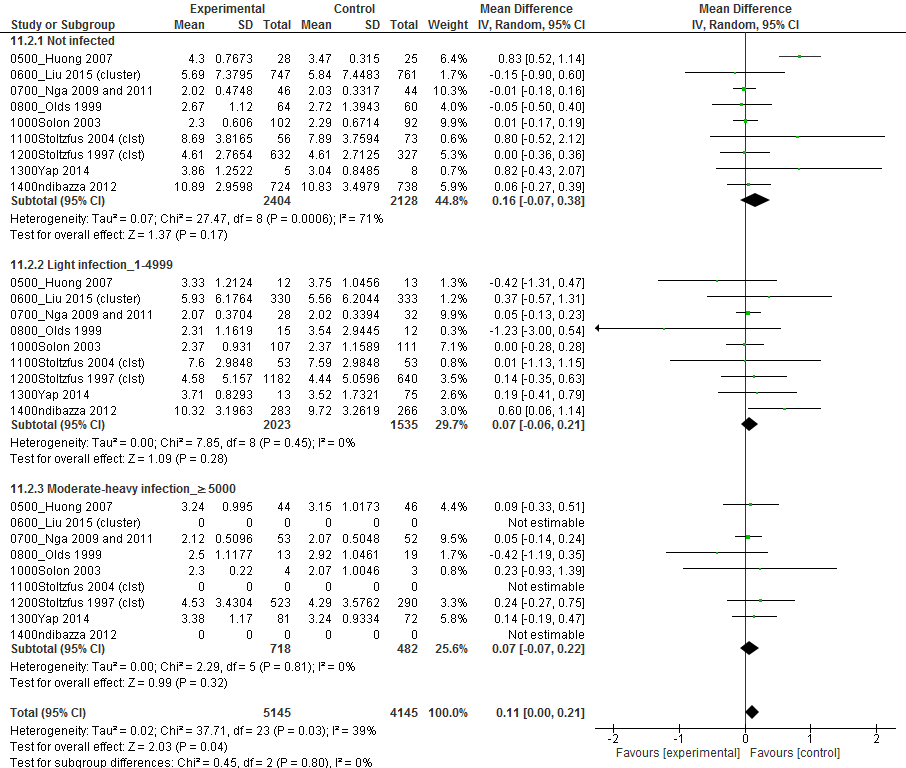
**

Additional figures 27: Change in haemoglobin (g/L), direct evidence, *A Lumbricoides*  infection intensity using WHO cutoffs as effect modifier

**
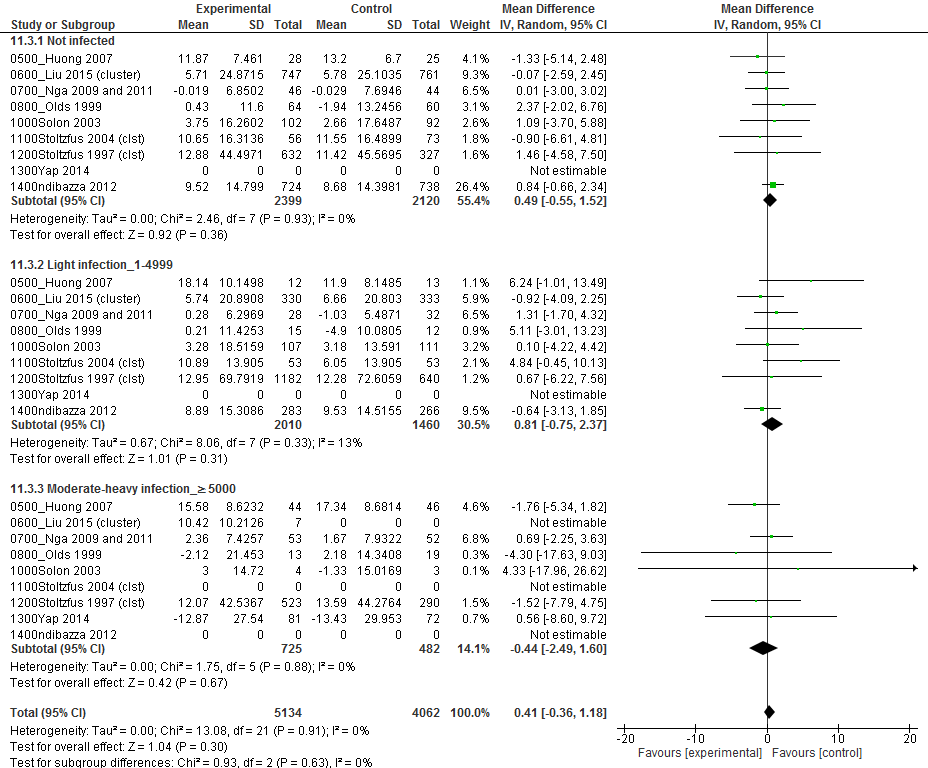
**

Additional figures 28: Weight gain (kg), direct evidence with hookworm intensityusing WHO cut-offs as effect modifier


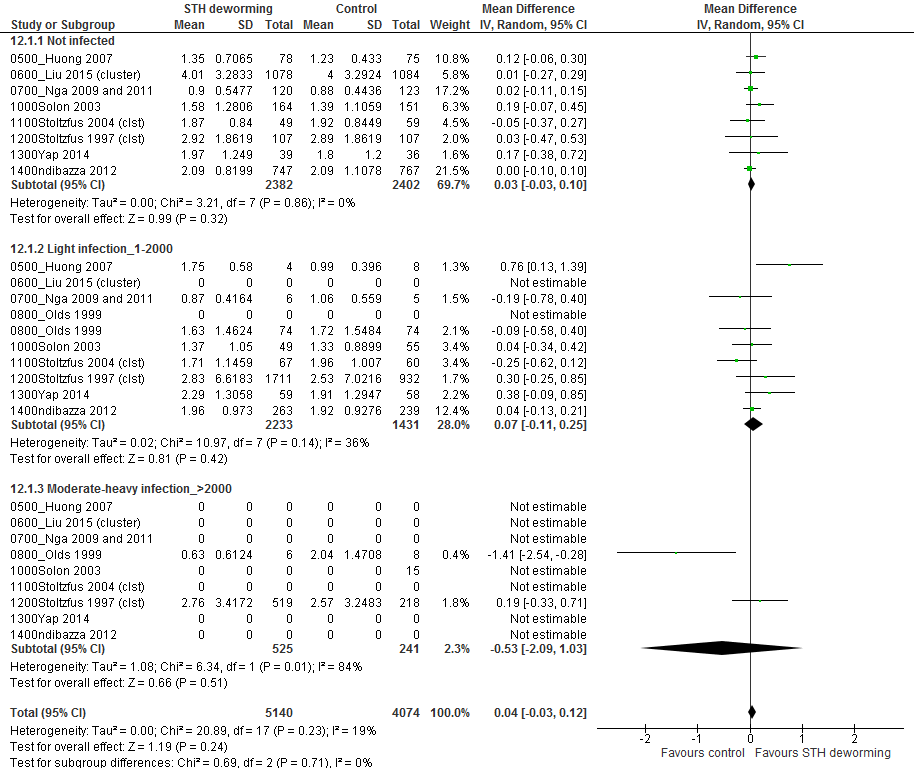


Additional figures 29: Height gain (cm), direct evidence with hookworm intensity using WHO cutoffs as effect modifier

**
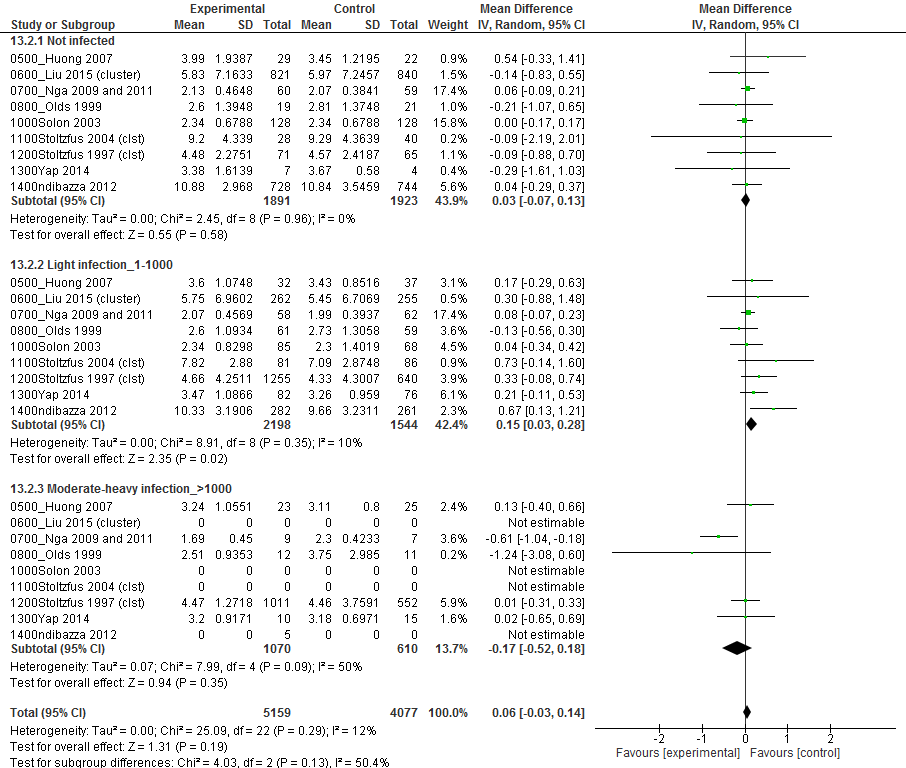
**

Additional figures 30: Change in haemoglobin (g/L), direct evidence, with hookworm intensity using WHO cutoffs as effect modifier

**
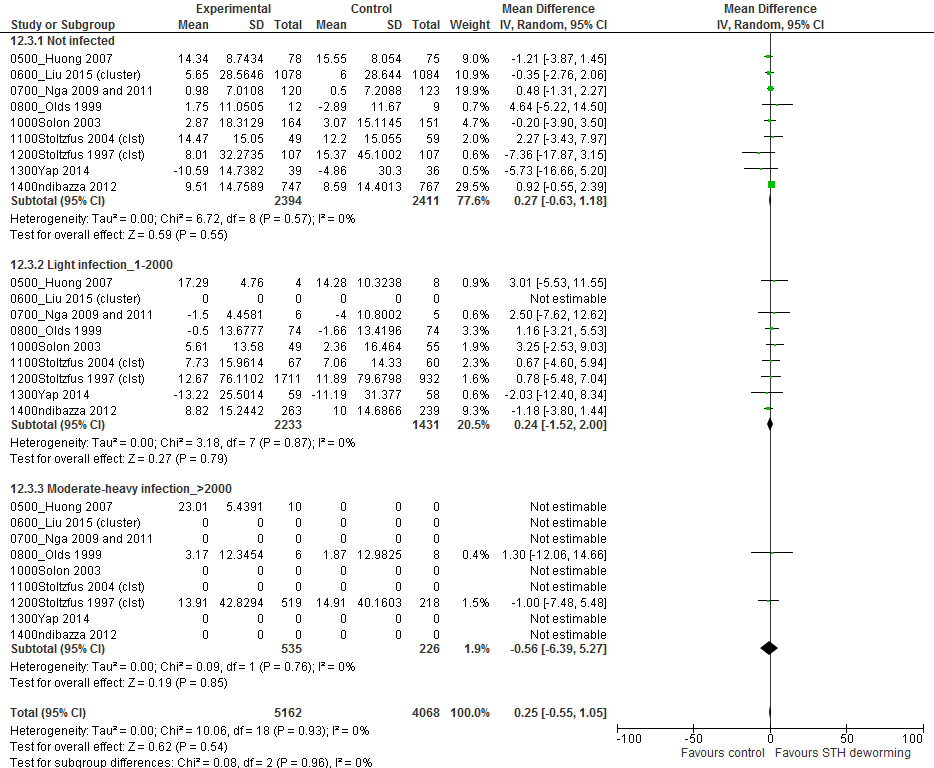
**

Additional figures 31: Weight gain (kg), direct evidence with *T. Trichiura*infection intensity using WHO cutoffs as effect modifier


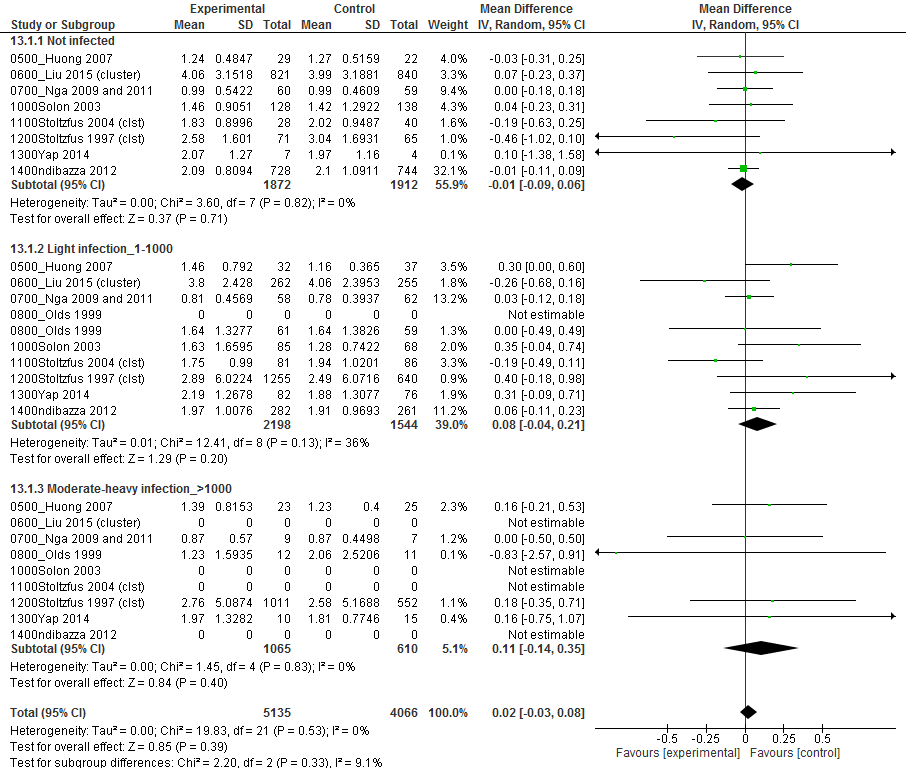


Additional figures 32: Height gain (cm), direct evidence with *T. Trichiura*infection intensity using WHO cutoffs as effect modifier

**
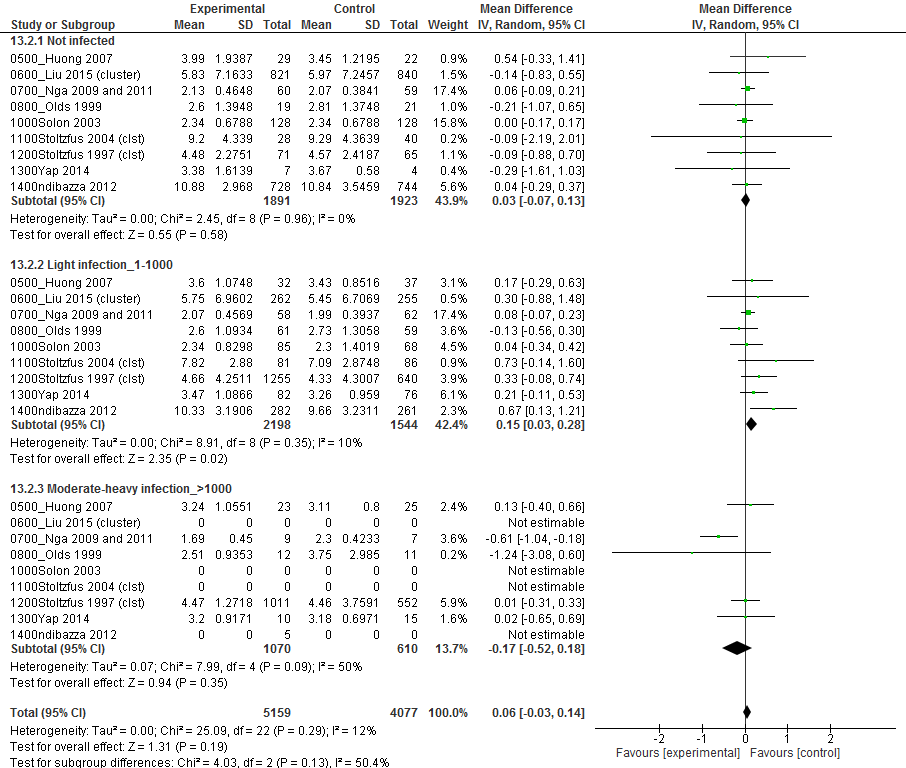
**

Additional figures 33: Change in haemoglobin (g/L),direct evidence with *T. Trichiura*infection intensity using WHO cutiffs as effect modifier

**
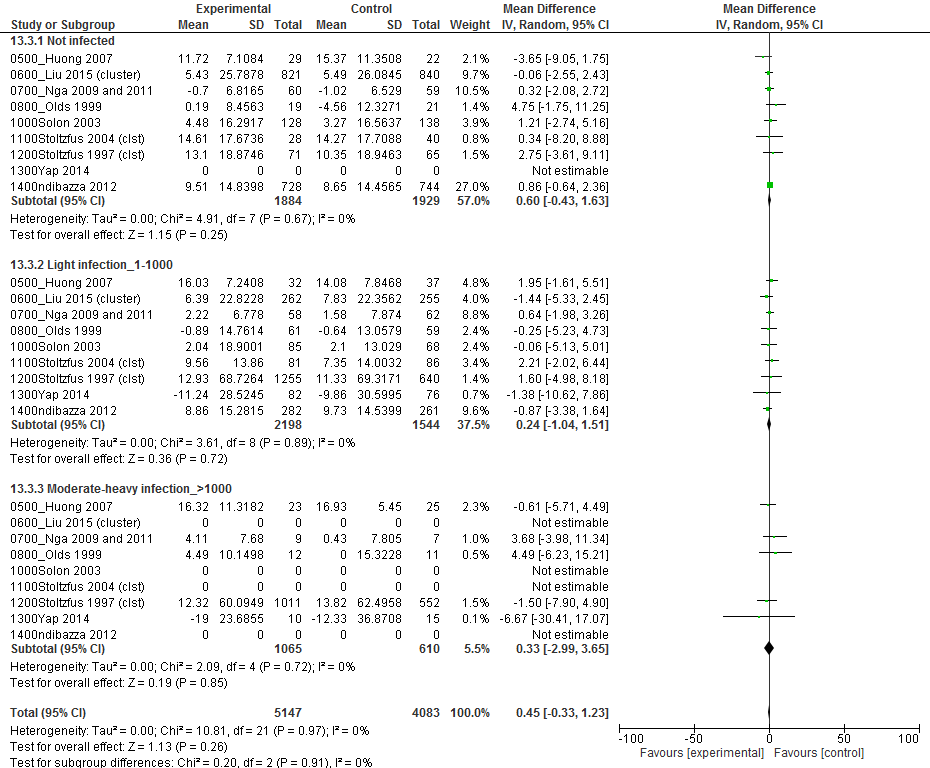
**

Additional figures 34: Weight gain (kg), with intensity of any STH as effect modifier


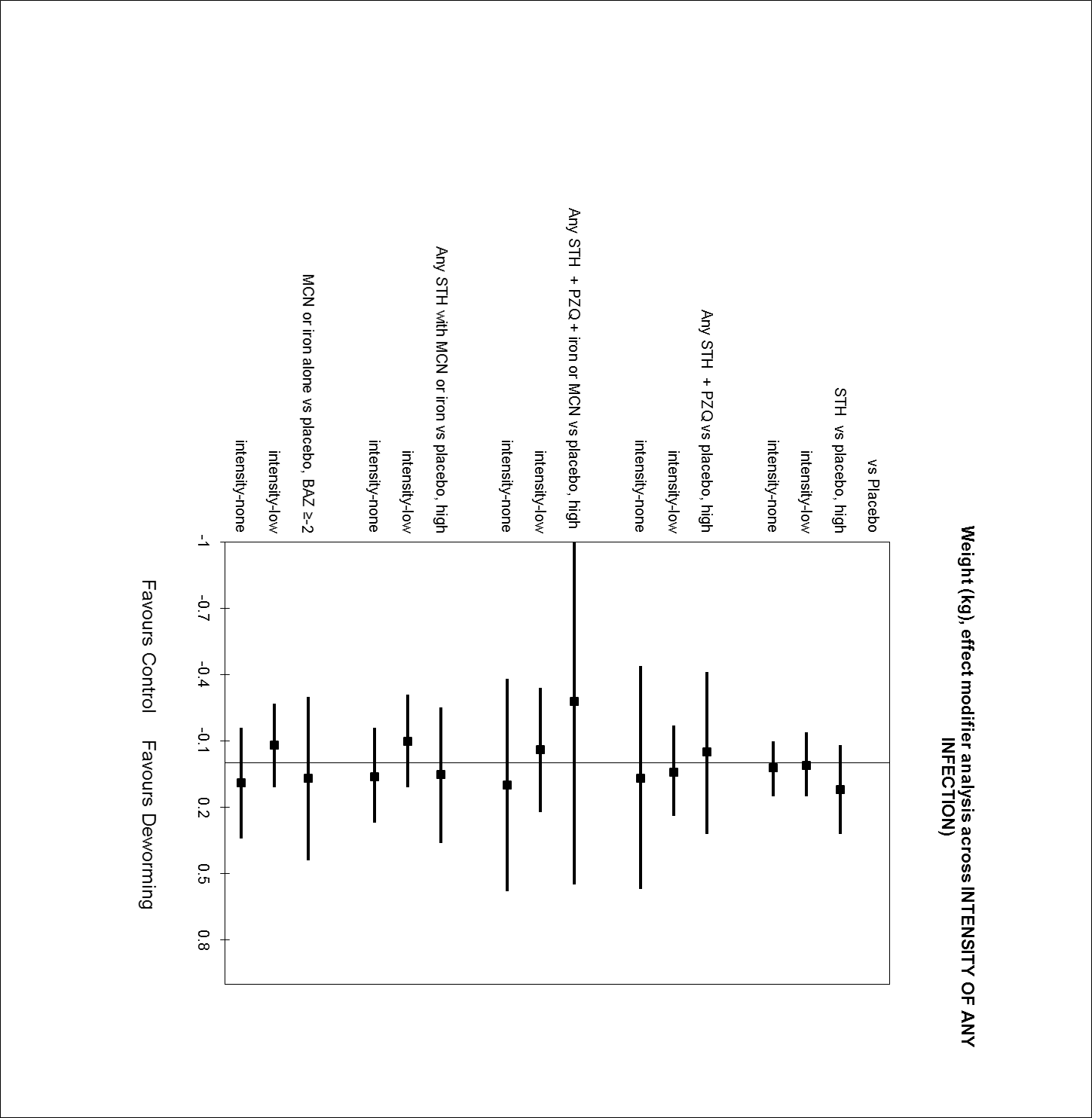


Additional figures 35: Height gain (cm) with intensity of any STH as effect modifier

Additional figures 36: Change in haemoglobin (g/L), with intensity of any STH as effect modifier


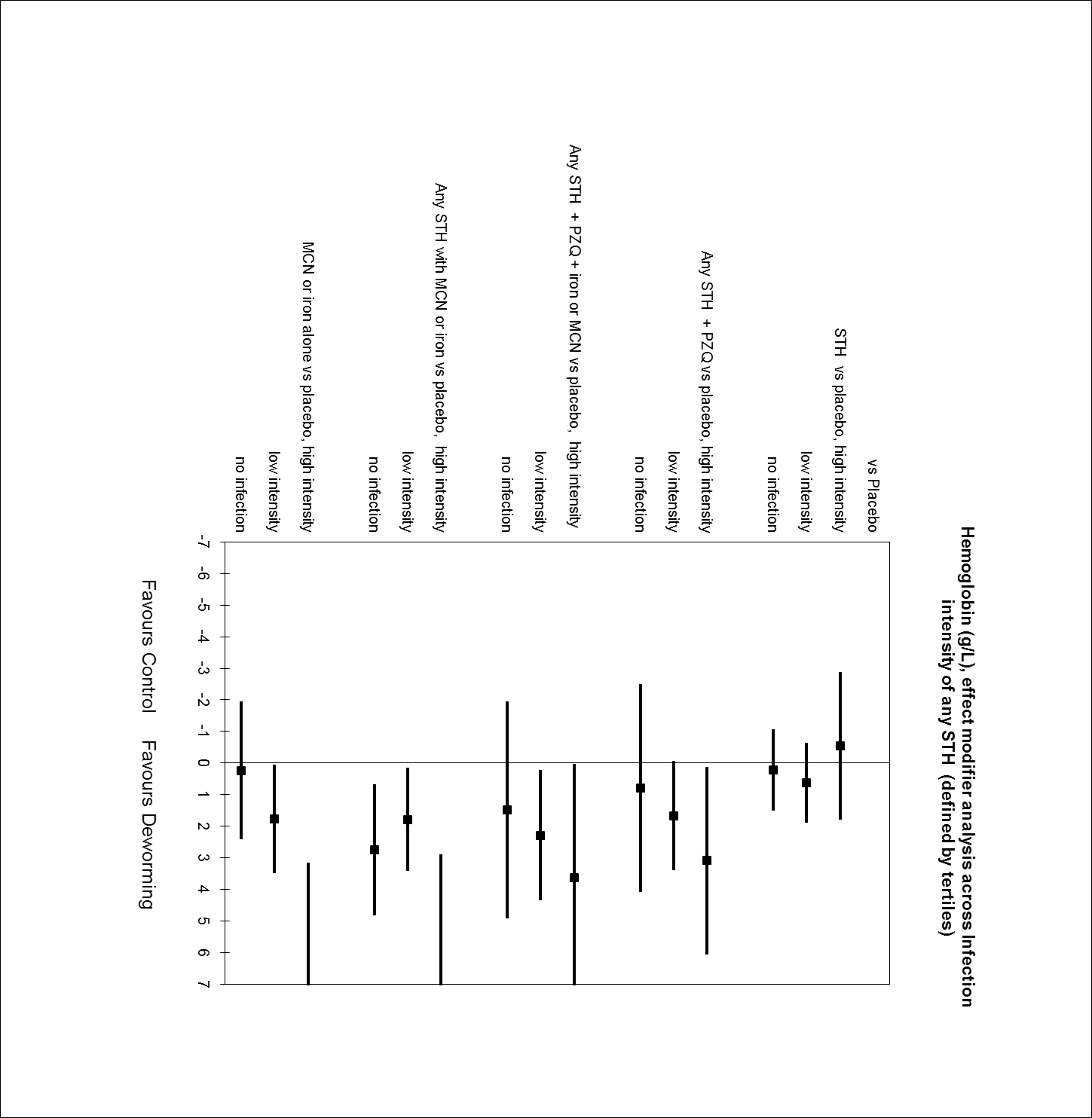


Additional figures 37: Weight gain (kg), with anaemia as effect modifier


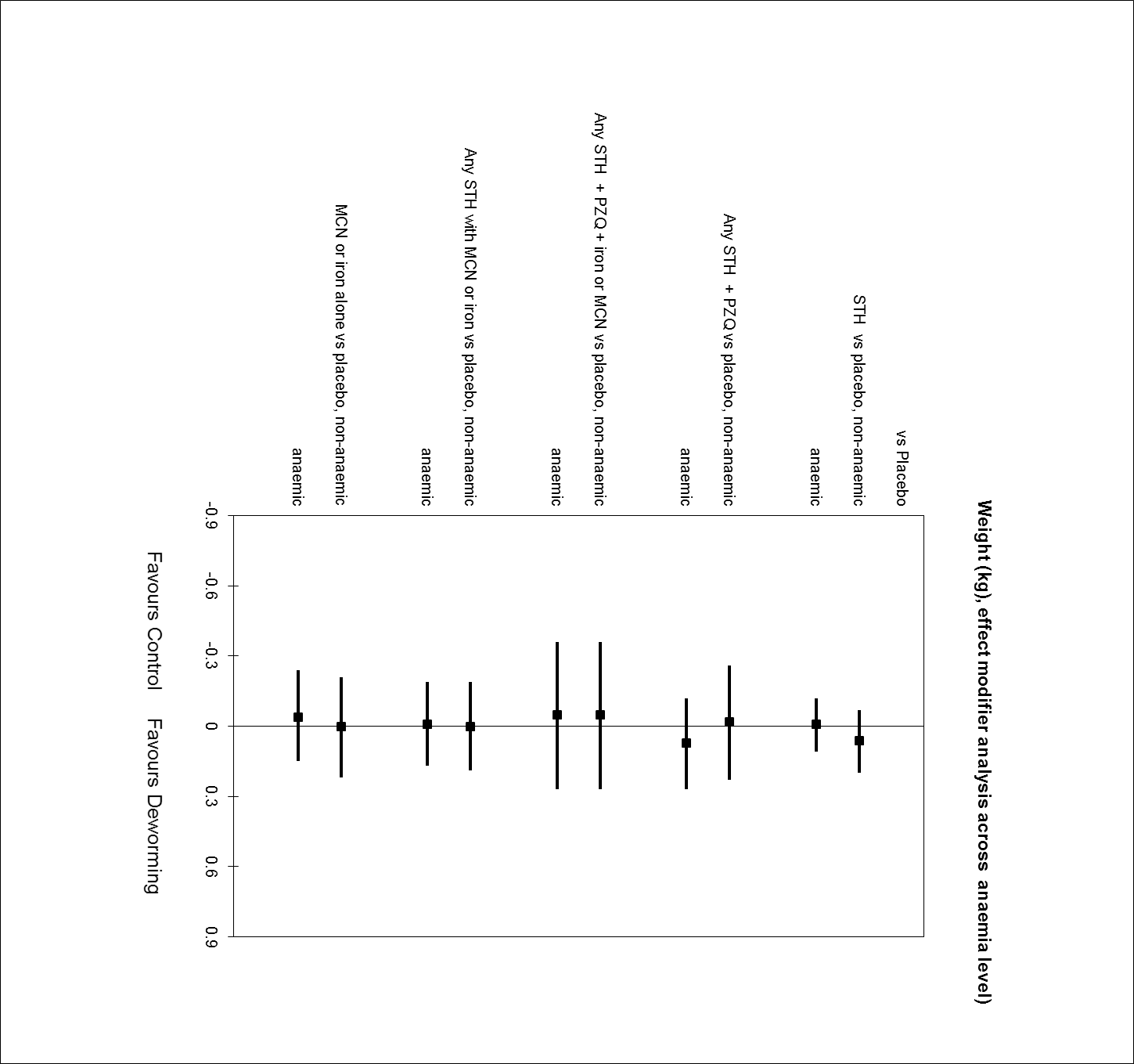


Additional figures 38: Height gain (cm), with anaemia as effect modifier

Additional figures 39: Change in haemoglobin (g/L), with anaemia as effect modifier


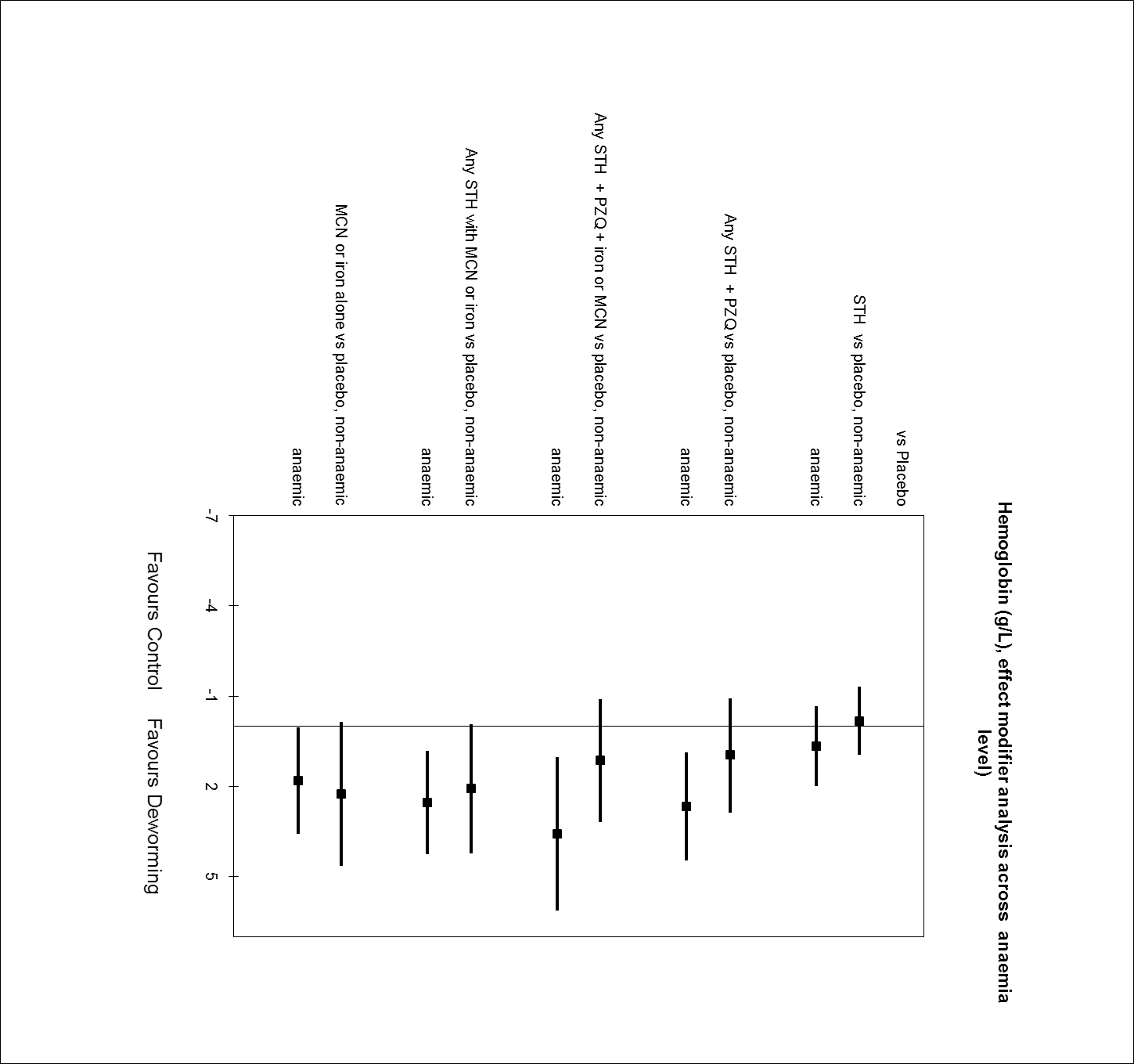


Additional tables 1: PRISMA-IPD and PRISMA NMA reporting checklists

**PRISMA-IPD Checklist of items to include when reporting a systematic review and meta-analysis of individual participant data (IPD)**

| **PRISMA-IPD**  **Section/topic** | **Item No** | **Checklist item** | **Reported on page** |
| --- | --- | --- | --- |
| **Title** | | | |
| Title | 1 | Identify the report as a systematic review and meta-analysis of individual participant data. | 1 |
| **Abstract** | | | |
| Structured summary | 2 | Provide a structured summary including as applicable: | 2 |
|  |  | **Background**: state research question and main objectives, with information on participants, interventions, comparators and outcomes. |  |
|  |  | **Methods**: report eligibility criteria; data sources including dates of last bibliographic search or elicitation, noting that IPD were sought; methods of assessing risk of bias. |  |
|  |  | **Results**: provide number and type of studies and participants identified and number (%) obtained; summary effect estimates for main outcomes (benefits and harms) with confidence intervals and measures of statistical heterogeneity. Describe the direction and size of summary effects in terms meaningful to those who would put findings into practice. |  |
|  |  | **Discussion:** state main strengths and limitations of the evidence, general interpretation of the results and any important implications. |  |
|  |  | **Other:** report primary funding source, registration number and registry name for the systematic review and IPD meta-analysis. |  |
| **Introduction** | | | |
| Rationale | 3 | Describe the rationale for the review in the context of what is already known. | 7-11 |
| Objectives | 4 | Provide an explicit statement of the questions being addressed with reference, as applicable, to participants, interventions, comparisons, outcomes and study design (PICOS). Include any hypotheses that relate to particular types of participant-level subgroups. | 11 |
| **Methods** | | | |
| Protocol and registration | 5 | Indicate if a protocol exists and where it can be accessed. If available, provide registration information including registration number and registry name. Provide publication details, if applicable. | 11 |
| Eligibility criteria | 6 | Specify inclusion and exclusion criteria including those relating to participants, interventions, comparisons, outcomes, study design and characteristics (e.g. years when conducted, required minimum follow-up). Note whether these were applied at the study or individual level i.e. whether eligible participants were included (and ineligible participants excluded) from a study that included a wider population than specified by the review inclusion criteria. The rationale for criteria should be stated. | 12-13 |
| Identifying studies - information sources | 7 | Describe all methods of identifying published and unpublished studies including, as applicable: which bibliographic databases were searched with dates of coverage; details of any hand searching including of conference proceedings; use of study registers and agency or company databases; contact with the original research team and experts in the field; open adverts and surveys. Give the date of last search or elicitation. | 13-14 |
| Identifying studies - search | 8 | Present the full electronic search strategy for at least one database, including any limits used, such that it could be repeated. | 188-220 |
| Study selection processes | 9 | State the process for determining which studies were eligible for inclusion. | 14 |
| Data collection processes | 10 | Describe how IPD were requested, collected and managed, including any processes for querying and confirming data with investigators. If IPD were not sought from any eligible study, the reason for this should be stated (for each such study). | 15 |
|  |  | If applicable, describe how any studies for which IPD were not available were dealt with. This should include whether, how and what aggregate data were sought or extracted from study reports and publications (such as extracting data independently in duplicate) and any processes for obtaining and confirming these data with investigators. |  |
| Data items | 11 | Describe how the information and variables to be collected were chosen. List and define all study level and participant level data that were sought, including baseline and follow-up information. If applicable, describe methods of standardising or translating variables within the IPD datasets to ensure common scales or measurements across studies. | 14-15 |
| IPD integrity | A1 | Describe what aspects of IPD were subject to data checking (such as sequence generation, data consistency and completeness, baseline imbalance) and how this was done. | 15, 19 |
| Risk of bias assessment in individual studies. | 12 | Describe methods used to assess risk of bias in the individual studies and whether this was applied separately for each outcome. If applicable, describe how findings of IPD checking were used to inform the assessment. Report if and how risk of bias assessment was used in any data synthesis. | 14 |
| Specification of outcomes and effect measures | 13 | State all treatment comparisons of interests. State all outcomes addressed and define them in detail. State whether they were pre-specified for the review and, if applicable, whether they were primary/main or secondary/additional outcomes. Give the principal measures of effect (such as risk ratio, hazard ratio, difference in means) used for each outcome. | 12-13 |
| Synthesis methods | 14 | Describe the meta-analysis methods used to synthesise IPD. Specify any statistical methods and models used. Issues should include (but are not restricted to):   - Use of a one-stage or two-stage approach. - How effect estimates were generated separately within each study and combined across studies (where applicable). - Specification of one-stage models (where applicable) including how clustering of patients within studies was accounted for. - Use of fixed or random effects models and any other model assumptions, such as proportional hazards. - How (summary) survival curves were generated (where applicable). - Methods for quantifying statistical heterogeneity (such as I^2^ and τ^2^). - How studies providing IPD and not providing IPD were analysed together (where applicable). - How missing data within the IPD were dealt with (where applicable). | 15-17 |
| Exploration of variation in effects | A2 | If applicable, describe any methods used to explore variation in effects by study or participant level characteristics (such as estimation of interactions between effect and covariates). State all participant-level characteristics that were analysed as potential effect modifiers, and whether these were pre-specified. | 17-18 |
| Risk of bias across studies | 15 | Specify any assessment of risk of bias relating to the accumulated body of evidence, including any pertaining to not obtaining IPD for particular studies, outcomes or other variables. | 18-19 |
| Additional analyses | 16 | Describe methods of any additional analyses, including sensitivity analyses. State which of these were pre-specified. | 18 |
| **Results** | | | |
| Study selection and IPD obtained | 17 | Give numbers of studies screened, assessed for eligibility, and included in the systematic review with reasons for exclusions at each stage. Indicate the number of studies and participants for which IPD were sought and for which IPD were obtained. For those studies where IPD were not available, give the numbers of studies and participants for which aggregate data were available. Report reasons for non-availability of IPD. Include a flow diagram. | 20-23 |
| Study characteristics | 18 | For each study, present information on key study and participant characteristics (such as description of interventions, numbers of participants, demographic data, unavailability of outcomes, funding source, and if applicable duration of follow-up). Provide (main) citations for each study. Where applicable, also report similar study characteristics for any studies not providing IPD. | 23-26, 104-123 |
| IPD integrity | A3 | Report any important issues identified in checking IPD or state that there were none. | 31-38 |
| Risk of bias within studies | 19 | Present data on risk of bias assessments. If applicable, describe whether data checking led to the up-weighting or down-weighting of these assessments. Consider how any potential bias impacts on the robustness of meta-analysis conclusions. | 27-30 |
| Results of individual studies | 20 | For each comparison and for each main outcome (benefit or harm), for each individual study report the number of eligible participants for which data were obtained and show simple summary data for each intervention group (including, where applicable, the number of events), effect estimates and confidence intervals. These may be tabulated or included on a forest plot. | 51-53  (no # of participants) |
| Results of syntheses | 21 | Present summary effects for each meta-analysis undertaken, including confidence intervals and measures of statistical heterogeneity. State whether the analysis was pre-specified, and report the numbers of studies and participants and, where applicable, the number of events on which it is based. | 43-79  (no # of participants) |
|  |  | When exploring variation in effects due to patient or study characteristics, present summary interaction estimates for each characteristic examined, including confidence intervals and measures of statistical heterogeneity. State whether the analysis was pre-specified. State whether any interaction is consistent across trials. |  |
|  |  | Provide a description of the direction and size of effect in terms meaningful to those who would put findings into practice. |  |
| Risk of bias across studies | 22 | Present results of any assessment of risk of bias relating to the accumulated body of evidence, including any pertaining to the availability and representativeness of available studies, outcomes or other variables. | 46, 49, 50, 51, 53 |
| Additional analyses | 23 | Give results of any additional analyses (e.g. sensitivity analyses). If applicable, this should also include any analyses that incorporate aggregate data for studies that do not have IPD. If applicable, summarise the main meta-analysis results following the inclusion or exclusion of studies for which IPD were not available. | 46, |
| **Discussion** | | | |
| Summary of evidence | 24 | Summarise the main findings, including the strength of evidence for each main outcome. | **91-92** |
| Strengths and limitations | 25 | Discuss any important strengths and limitations of the evidence including the benefits of access to IPD and any limitations arising from IPD that were not available. | **91-92** |
| Conclusions | 26 | Provide a general interpretation of the findings in the context of other evidence. | **91-92** |
| Implications | A4 | Consider relevance to key groups (such as policy makers, service providers and service users). Consider implications for future research. | **91-92** |
| **Funding** | | | |
| Funding | 27 | Describe sources of funding and other support (such as supply of IPD), and the role in the systematic review of those providing such support. | 84 |

**A1 – A3 denote new items that are additional to standard PRISMA items. A4 has been created as a result of re-arranging content of the standard PRISMA statement to suit the way that systematic review IPD meta-analyses are reported.**

© Reproduced with permission of the PRISMA IPD Group, which encourages sharing and reuse for non-commercial purposes

Additional tables 2: PRISMA NMA Checklist of Items to Include When Reporting A Systematic Review Involving a Network Meta-analysis

| **Section/Topic** | **Item #** | **Checklist Item** | **Reported on Page #** |
| --- | --- | --- | --- |
| **TITLE** |  |  |  |
| Title | 1 | Identify the report as a systematic review *incorporating a network meta-analysis (or related form of meta-analysis).* | 1 |
|  |  |  |  |
| **ABSTRACT** |  |  |  |
| Structured summary | 2 | Provide a structured summary including, as applicable:  **Background:** main objectives  **Methods:** data sources; study eligibility criteria, participants, and interventions; study appraisal; and *synthesis methods, such as network meta-analysis.*  **Results:** number of studies and participants identified; summary estimates with corresponding confidence/credible intervals; *treatment rankings may also be discussed. Authors may choose to summarize pairwise comparisons against a chosen treatment included in their analyses for brevity.*  **Discussion/Conclusions:** limitations; conclusions and implications of findings.  **Other:** primary source of funding; systematic review registration number with registry name. | 2 |
|  |  |  |  |
| **INTRODUCTION** |  |  |  |
| Rationale | 3 | Describe the rationale for the review in the context of what is already known*, including mention of why a network meta-analysis has been conducted.* | ***7-11*** |
| Objectives | 4 | Provide an explicit statement of questions being addressed, with reference to participants, interventions, comparisons, outcomes, and study design (PICOS). | 11 |
|  |  |  |  |
| **METHODS** |  |  |  |
| Protocol and registration | 5 | Indicate whether a review protocol exists and if and where it can be accessed (e.g., Web address); and, if available, provide registration information, including registration number. | 11 |
| Eligibility criteria | 6 | Specify study characteristics (e.g., PICOS, length of follow-up) and report characteristics (e.g., years considered, language, publication status) used as criteria for eligibility, giving rationale. *Clearly describe eligible treatments included in the treatment network, and note whether any have been clustered or merged into the same node (with justification).* | ***12-13*** |
| Information sources | 7 | Describe all information sources (e.g., databases with dates of coverage, contact with study authors to identify additional studies) in the search and date last searched. | 13-14 |
| Search | 8 | Present full electronic search strategy for at least one database, including any limits used, such that it could be repeated. | 188-220 |
| Study selection | 9 | State the process for selecting studies (i.e., screening, eligibility, included in systematic review, and, if applicable, included in the meta-analysis). | 14 |
| Data collection process | 10 | Describe method of data extraction from reports (e.g., piloted forms, independently, in duplicate) and any processes for obtaining and confirming data from investigators. | 15 |
| Data items | 11 | List and define all variables for which data were sought (e.g., PICOS, funding sources) and any assumptions and simplifications made. | 14-15 |
| **Geometry of the network** | **S1** | Describe methods used to explore the geometry of the treatment network under study and potential biases related to it. This should include how the evidence base has been graphically summarized for presentation, and what characteristics were compiled and used to describe the evidence base to readers. | ***12, 15-16*** |
| Risk of bias within individual studies | 12 | Describe methods used for assessing risk of bias of individual studies (including specification of whether this was done at the study or outcome level), and how this information is to be used in any data synthesis. | 14 |
| Summary measures | 13 | State the principal summary measures (e.g., risk ratio, difference in means). *Also describe the use of additional summary measures assessed, such as treatment rankings and surface under the cumulative ranking curve (SUCRA) values, as well as modified approaches used to present summary findings from meta-analyses.* | 12-13 |
| Planned methods of analysis | 14 | Describe the methods of handling data and combining results of studies for each network meta-analysis. This should include, but not be limited to:   - *Handling of multi-arm trials;* - *Selection of variance structure;* - *Selection of prior distributions in Bayesian analyses; and* - *Assessment of model fit.* | 15-17 |
| **Assessment of Inconsistency** | **S2** | Describe the statistical methods used to evaluate the agreement of direct and indirect evidence in the treatment network(s) studied. Describe efforts taken to address its presence when found. | 16, 17 |
| Risk of bias across studies | 15 | Specify any assessment of risk of bias that may affect the cumulative evidence (e.g., publication bias, selective reporting within studies). | **17** |
| Additional analyses | 16 | Describe methods of additional analyses if done, indicating which were pre-specified. This may include, but not be limited to, the following:   - Sensitivity or subgroup analyses; - Meta-regression analyses; - *Alternative formulations of the treatment network; and* - *Use of alternative prior distributions for Bayesian analyses (if applicable).* | ***17-18*** |
|  |  |  |  |
| **RESULTS†** |  |  |  |
| Study selection | 17 | Give numbers of studies screened, assessed for eligibility, and included in the review, with reasons for exclusions at each stage, ideally with a flow diagram. | 20-23 |
| **Presentation of network structure** | **S3** | Provide a network graph of the included studies to enable visualization of the geometry of the treatment network. |  |
| **Summary of network geometry** | **S4** | Provide a brief overview of characteristics of the treatment network. This may include commentary on the abundance of trials and randomized patients for the different interventions and pairwise comparisons in the network, gaps of evidence in the treatment network, and potential biases reflected by the network structure. | ***35-41*** |
| Study characteristics | 18 | For each study, present characteristics for which data were extracted (e.g., study size, PICOS, follow-up period) and provide the citations. | 23-26, 104-123 |
| Risk of bias within studies | 19 | Present data on risk of bias of each study and, if available, any outcome level assessment. | 27-30 |
| Results of individual studies | 20 | For all outcomes considered (benefits or harms), present, for each study: 1) simple summary data for each intervention group, and 2) effect estimates and confidence intervals. *Modified approaches may be needed to deal with information from larger networks.* | ***51-53*** |
| Synthesis of results | 21 | Present results of each meta-analysis done, including confidence/credible intervals. *In larger networks, authors may focus on comparisons versus a particular comparator (e.g. placebo or standard care), with full findings presented in an appendix. League tables and forest plots may be considered to summarize pairwise comparisons.* If additional summary measures were explored (such as treatment rankings), these should also be presented. | ***43-79*** |
| **Exploration for inconsistency** | **S5** | Describe results from investigations of inconsistency. This may include such information as measures of model fit to compare consistency and inconsistency models, *P* values from statistical tests, or summary of inconsistency estimates from different parts of the treatment network. | ***41, 43-45, 48-50*** |
| Risk of bias across studies | 22 | Present results of any assessment of risk of bias across studies for the evidence base being studied. | 46, 49, 51 |
| Results of additional analyses | 23 | Give results of additional analyses, if done (e.g., sensitivity or subgroup analyses, meta-regression analyses*, alternative network geometries studied, alternative choice of prior distributions for Bayesian analyses,* and so forth). | ***46, 49, 50, 53-80*** |
|  |  |  |  |
| **DISCUSSION** |  |  |  |
| Summary of evidence | 24 | Summarize the main findings, including the strength of evidence for each main outcome; consider their relevance to key groups (e.g., healthcare providers, users, and policy-makers). | 91-92 |
| Limitations | 25 | Discuss limitations at study and outcome level (e.g., risk of bias), and at review level (e.g., incomplete retrieval of identified research, reporting bias). *Comment on the validity of the assumptions, such as transitivity and consistency. Comment on any concerns regarding network geometry (e.g., avoidance of certain comparisons).* | 92-92 |
| Conclusions | 26 | Provide a general interpretation of the results in the context of other evidence, and implications for future research. | 91-92 |
|  |  |  |  |
| **FUNDING** |  |  | 84 |
| Funding | 27 | Describe sources of funding for the systematic review and other support (e.g., supply of data); role of funders for the systematic review. This should also include information regarding whether funding has been received from manufacturers of treatments in the network and/or whether some of the authors are content experts with professional conflicts of interest that could affect use of treatments in the network. |  |

PICOS = population, intervention, comparators, outcomes, study design.

* Text in italics indicateS wording specific to reporting of network meta-analyses that has been added to guidance from the PRISMA statement.

† Authors may wish to plan for use of appendices to present all relevant information in full detail for items in this section.

Additional tables 3: Characteristics of included studies child and setting characteristics (n=19)

| **Study** | **Source of funding** | **% female** | **Method of assessing infection intensity** | **Infection prevalence**  *A Lumbricoides*, Hookworm, *T Trichuria* | **Weight (kg), SD** | **Height (cm), SD** |
| --- | --- | --- | --- | --- | --- | --- |
| Beasley 1999 | Wellcome Trust, Smithkline Beecham and the Department for International Development (UK) | 43% | Kato-Katz technique | 51%, 92%, 71% | 24.3 (4.7) | 123.8 (9.2) |
| Beasley Tanbase 1999  (thesis) | Wellcome Trust, Smithkline Beecham and the Department for International Development (UK) | 42% | Kato-Katz technique | 13%, 84%, 46% | 25.9 (4.1) | 129.6 (7.1) |
| Ebenezer 2013 | The Partnership for Child Development (PCD) with funds received through the Development Grant Facility programme of the World Bank.  State Pharmaceutical Manufacturing Corporation of Sri Lanka and MSJ Industries Ceylon for providing us with the placebo for mebendazole and ferrous sulphate tablets, respectively, free of charge | 48% | Modified Kato-Katz technique | 21%, 6%, 6% | 22.2 (3.3) | 126.0 (6.0) |
| Friis 2003 | The Danish International Development Assistance | 51% | Modified Kato-Katz technique | 14%, 55%, 45% | 37.4 (7.5) | 148.4 (9.5) |
| Huong 2007 | Neys-van Hoogstraten Foundation, Ellison Medical Foundation and the Ministry of Education and Training, Vietnam. Akzo Nobel Chemicals, Arnhem, The Netherlands, is acknowledged for supplying NaFeEDTA.  Hospital De Gelderse Vallei, Ede, The Netherlands is thanked for preparation of MEB and identical placebo. | 51% | Kato-Katz technique | 64%, 9%, 73% | 17.8 (2.4) | 113.8 (5.9) |
| Liu 2017 | The National Natural Science Foundation of China (grant numbers 71473240 and 71333012), as well as the International Initiative for Impact Evaluation (3IE, grant number PW2.04.02.02). | 46% | Kato-Katz technique | 17%, 0%, 17% | 28.4 (5.9) | 132.5 (8.1) |
| Nga 2009 | the Neys-van Hoogstraten Foundation, The Netherlands, and the Ellison Medical  Foundation. | 52% | Kato-Katz technique | 64%, 5%, 54% | 19.3 (2.7) | 116.2 (6.2) |
| Olds 1999 | Product Development Unit of WHO/TDR. Praziquantel from Bayer (Biltricide) and an identical placebo tablet (both, Merck, Darmstadt, Germany) and albendazole (Zentel) and an identical placebo tablet (both, SmithKline Beecham, Brentfore, UK) were donated to WHO/TDR. | 41% | Kato-Katz technique | 91%, 100%, 95% | 30.5 (10.0) | 136.3 (16.1) |
| Rohner 2010 | Not reported | 42% | Kato-Katz technique | 2%, 53%, 3% | 27.0 (8.1) | 130.2 (13.4) |
| Solon 2003 | Nutrition  Center of the Philippines and the Procter & Gamble Co. | 48% | Kato-Katz technique | 43%, 11%, 22% | 24.4 (6.5) | 126.5 (12.0) |
| Stoltzfus 2004 | Thrasher Research Fund, Cooperative Agreement #HRN-A-00-97-00015-00 between the Johns Hopkins University and the United States Agency for International Development, and Alpharma USPD, Baltimore, MD | 47% | Kato-Katz technique | 43%, 54%, 72% | 11.8 (2.7) | 86.9 (11.0) |
| Stoltzfus 1997 | The Johns Hopkins University and the Office of Health and Nutrition, United States Agency for International Development | 48% | Kato-Katz technique | 72%, 94% ,96% | 24.7 (5.6) | 128.8 (9.8) |
| Yap 2014 | the Swiss Tropical and Public Health Institute in Basel, Switzerland and the National Institute of Parasitic Diseases, Chinese Center of Diseases Control and Prevention in Shanghai, P.R. China. Health Institute in Basel, Switzerland and the National Institute of Parasitic Diseases, Chinese Center of Diseases Control and Prevention in Shanghai, P.R. China. The research of SJK and JU is financially supported by the Swiss National Science Foundation (project no. 320030_141246). | 52% | Kato-Katz technique | 93%, 61%, 94% | 26.0 (4.9) | 126.9 (8.1) |
| Ndibazza 2013 | Wellcome Trust grant numbers 064693 and 079110. Albendazole and matching placebo were provided by GlaxoSmithKline; mycobacterial antigens were provided through the National Institutes of Health contract NOI-AI-25147 | 48% | Kato-Katz technique | 1%, 0.2%, 1% | 9.1 (1.3) | 73.3 (3.1) |
| Hall 2006 | the Partnership for Child Development | 49% | Not described | 71%, 7%, 84% | 20.6 (2.2) | 121.1 (4.7) |
| Kirwan 2009 | Health Research Board for funding the project, and GlaxoSmithKline for providing the albendazole. | 48% | Not described | 47%, 4%, 4% | 12.0 (3.5) | 87.8 (11.3) |
| Miguel 2004 | the World Bank and the Partnership for Child Development | 48% | Kato-Katz technique | 42%, 77%, 55% | 37.0 (9.7) | Not reported |
| Rousham 1994 | the Overseas Development Administration and the University of Cambridge Maintenance Fund | 50% | Modified  ether sedimentation technique | 75%, 9%, 44% | 10.8 (1.6) | 86.1 (7.5) |
| Wiria 2013 | The Royal Netherlands Academy of Arts and Science (KNAW), Ref.KNAW-05-PP-35, European Commission contracts INCO-CT2006-031714 and INCO-CT-2006-032436, Glofal FP6-2003-FOOD-2-B, and the Prof. Dr. P.C. Flu Foundation | 50% | Multiplex, real-time PCR for A Lumbricoides and hookworm, formol concentration method for T Trichuria | 35%, 78%, 28% | 28.0 (11.8) | 131.1 (16.9) |

Additional tables 4: Excluded studies

| Studies | Participants | Reason For Exclusion |
| --- | --- | --- |
| STH deworming |  |  |
| Alderman 2006 | 14,940/13,055 | No intensity data collected |
| Awasthi 2000 | 1061 | No infection intensity collected |
| Awasthi 2001 | 2010 | No infection intensity collected |
| Awasthi 2008 | 3935 | No infection intensity collected |
| Awasthi 2013 (DEVTA articles) | >1,000,000 children | No infection intensity collected |
| Bhoite 2012 a& B | 496 | No infection intensity collected |
| Bobonis 2006 | 1462 | No infection intensity collected and did not follow the same children |
| Gateff 1972 | 584 | No infection intensity collected |
| Goto 2009 | 394 | Does not have baseline infection intensity |
| Gupta 1977 | 154 | Not an RCT |
| Gupta 1982 | 159 | Infection intensity not reported |
| Joseph 2015 | 1760 | Infection intensity only collected at endline |
| Kaba 1978 | 176 | Not an RCT |
| Kruger 1996 | 178 | No intensity of infection |
| Linnemayr 2011 | 4296 | no intensity of infection |
| Moser 2016 | 350 | No weight or height outcomes, cure rates only |
| Nokes 1992 | 104 | Exclude too short: 9 weeks long |
| Palupi 1997 | 289 | Exclude too short, 9 weeks long |
| Pust 1985 | 896 | Exclude CBA |
| Reddy 1986 | 517 | no intensity of infection |
| Shah 1975 | 325 | Exclude too short 3months |
| Simeon 1995 | 289 | Exclude too short 12 weeks |
| Sternberg 1997 | 196 | Exclude too short 10 weeks |
| Sur 2005 | 702 | no intensity of infection |
| Tahapary 2015 | protocol | Adults (aged 16 years and above) |
| Tanumihardjo 2004 | 131 | Ineligible -they gave all children deworming, but at 3 different times, and the study did not measure effects on growth at endline (which was only 4 weeks) |
| Wang 2017 | 254 | No baseline infection intensity |
| Schistosomiasis Studies | Participants | Reason For Excluding |
| Bejon 2008 | 405 | Not randomized by deworming treatment |
| Borrmann 2001 | 300 | too short, 56 days |
| Druilhe 1981 | 325 | not an RCT |
| Inyang-Etoh 2004 | 145 | too short, 4 weeks |
| Karanja 2017 |  | outcome is infection intensity only, no weight or height measured |
| Kosinski 2012 | 247 | not an RCT |
| Koukonari 2006 | 2788 | not an RCT |
| Kvalsvig 1991 | 1991 | not an RCT |
| Monse 2013 | 412 | not an RCT |
| Obonyo 2010 | 212 | too short, 28 days |
| Sissoko 2009 | 800 | too short (28 days) |
|  |  |  |

Additional tables 5: Ongoing studies

| STH Studies | Participants | Details |
| --- | --- | --- |
| Leung 2016 | 700 | Ongoing RCT of albendazole on diarrheal episodes, with WAZ as an outcome |

Additional tables 6: STH studies contacted for data, showing whether data was received

| Study | category | Participants | Received | Eligible | Pending | Refused | Data lost | No response |
| --- | --- | --- | --- | --- | --- | --- | --- | --- |
| 1.Ebenezer 2013 | sth post 2000 | 1621 | X | Yes |  |  |  |  |
| 2.Friis 2003 | sth post 2000 | 746 | X | Yes |  |  |  |  |
| 3.Liu 2015 | sth post 2000 | 2179 | X | Yes |  |  |  |  |
| 4.Kirwan 2009 | sth post 2000 | 1228 | X | Yes |  |  |  |  |
| 5.Le Thi Huong 2007 | sth post 2000 | 425 | X | Yes |  |  |  |  |
| 6.Nga 2009 | sth post 2000 | 510 | X | Yes |  |  |  |  |
| 7.Rohner 2010 | sth post 2000 | 591 | X | Yes |  |  |  |  |
| 8.Solon 2003 | sth post 2000 | 800 | X | Yes |  |  |  |  |
| 9.Yap 2014 | sth post 2000 | 211 | X | Yes |  |  |  |  |
| 10.Fox 2005 | sth post 2000 | 1292 |  | Yes | X |  |  |  |
| 11.Garg 2002 | sth post 2000 | 370 |  | Yes | X |  |  |  |
| 12.Stoltzfus 1997 and 2001 | sth post 2000 | 3605 | x | Yes |  |  |  |  |
| 13.Stoltzfus 2001&2004 | sth post 2000 | 621 | x | Yes |  |  |  |  |
| 14.Haque 2010 | sth post 2000 | 248 |  | Yes |  |  | x |  |
| 15.Jinabhai 2001(2) | sth post 2000 | 268 |  | Yes |  |  | x |  |
| 16. JinabhaiB 2001 | sth post-2000 | 579 |  |  |  |  |  |  |
| 17.Taylor 2001 | sth post 2000 | 428 |  | Yes |  |  | x |  |
| 18.Dossa 2001 | sth post 2000 | 140 |  | Yes |  |  |  | X |
| 19. Miguel 2006 | sth post 2000 | 7434 | x | yes |  |  |  |  |
| 20. Hall 2006 | sth post 2000 | 2659 | x | yes |  |  |  |  |
| 21. Ndibazza 2012 | sth post 2000 | 2016 | x | yes |  |  |  |  |
| 22. Wiria 2013 | sth post 2000 | 4004 | x | yes |  |  |  |  |
| 1. Donnen 1998 | STH pre-2000 | 358 | X | Yes |  |  |  |  |
| 2. Olds 1999 | STH pre-2000 | 370 | X | Yes |  |  |  |  |
| 3. Rousham 1994 | STH pre-2000 | 1402 | X | Yes |  |  |  |  |
| 4.Beach 1999 | STH pre-2000 | 965 |  | Yes | X |  |  |  |
| 5.Stephenson 1989 | STH pre-2000 | 150 |  | Yes | X |  |  |  |
| 6.Watkins 1996 | STH pre-2000 | 250 |  | Yes |  |  | x |  |
| 7.Greenberg 1981 | STH pre-2000 | 185 |  | Yes |  |  | X |  |
| 8.Koroma 1996 | STH pre-2000 | 247 |  | Yes |  |  | x |  |
| 9.Michaelson 1985 | STH pre-2000 | 228 |  | Yes |  |  | x |  |
| 10.Pust 1985 | STH pre-2000 | 696 |  | Yes |  |  | X |  |
| 11.Willett 1979 | STH pre-2000 | 341 |  | Yes |  |  | x |  |
| 12.Hadidjaja 1998 | STH pre-2000 | 1000 |  | Yes |  |  |  | X |
| 13.Hadju 1997 | STH pre-2000 | 330 |  | Yes |  |  |  | X |
| 14.Henry 1988 | STH pre-2000 | 229 |  | Yes |  |  | X |  |
| 15.Kloetzel 1982 | STH pre-2000 | 337 |  | Yes |  |  |  | X |
| 16.Lai 1995 | STH pre-2000 | 353 |  | Yes |  |  |  | X |
| 17.Ostwald 1984 | STH pre-2000 | 118 |  | Yes |  |  |  | X |
| 18. Beasley 1999 (Tanga) | STH pre-2000 | 357 | X | Yes |  |  |  |  |
| 19. Beasley (Muheza) | STH pre-2000 | 1166 | X | Yes |  |  |  |  |

Additional tables 7: Schistosomiasis studies providing data

| Study | Participants | Collaborative | Received | Eligible | Pending | Refused | Data lost | No response |
| --- | --- | --- | --- | --- | --- | --- | --- | --- |
| 1.Bhargava 2003 | 1650 | Matthew Jukes | X | Yes |  |  |  |  |
| 3.Ferrari 2003 | 106 |  |  | Yes |  |  |  | X |
| 1.Olds 1999 | 370 | Charles King | X | Yes |  |  |  |  |
| 2.Befidi-Mengue 1992 | 653 | Raoult Ratard |  | Yes | X |  |  |  |
| 3.Kardaman 1985 | 237 | Alan Fenwick |  | Yes |  |  | X |  |
| 4.Lambertucci 1982 | 136 |  |  | Yes |  | X |  |  |
| 5.Branchini 1982 | 101 |  |  | Yes |  |  | X |  |
| 6.Assis 1998 | 489 |  |  | Yes |  |  |  | X |
| 7.Clark 1973 | 442 |  |  | Yes |  |  |  | X |
| 8.da Silva 1986 | 120 |  |  | Yes |  |  |  | X |
| 9.Ibrahim 1980 | 129 |  |  | Yes |  |  |  | X |
| 10.Omer 1981 | 153 |  |  | Yes |  |  |  | X |
| 11.Rezende 1985 | 539 |  |  | Yes |  |  |  | X |
| 12.Sukwa 1993 | 377 |  |  | Yes |  |  |  | X |

Additional tables 8: Characteristics of included studies that provided data, context and setting (n=19)

| Authors | Study design | Country | Proportion female; Socioeconomic status | Baseline weight (kg); weight-for-age (WAZ); proportion under-weight | Baseline height (cm); height-for-age (HAZ); proportion stunted | Nutritional status | Outcomes measured | Worm prevalence | Worm intensity | Environmental risk for worm infection |
| --- | --- | --- | --- | --- | --- | --- | --- | --- | --- | --- |
| Beasley 1999 | RCT | Tanzania  Community based | Proportion female: 48.4%  socioeconomic status scores (treatment score : 2.67, placebo  score : 2.72 P : 0.94). | NR | NR | Baseline Prevalence of Anemia (%): Treatment: 46 Placebo: 51 p-value: 0.38 r Mean haemoglobin (g/dl) 6 SE: Treament: 11.0 +/- 0.09 r Placebo 11.0 +/- 0.09 p-value: 0.80 | Hookworm, A. lumbricoides, S.  Haematobium, Weight, height,  concentration of haemoglobin and intensity  of infection with Plasmodium spp. | Hookworm  Baseline Treatment: 94 Placebo 92 P-value 0.31  T. trichiura  Baseline Treatment 69 Placebo 67 P-value 0.76 A. lumbricoides  Baseline Treatment 47 Placebo 51 P-value 0.54 | Hookworm  Baseline Treatment 2247 +/- 31.2 0 Placebo 1837 +/- 226 P-value 0.59 T. trichiura  Baseline 0 Treatment 449 +/- 89 00 Placebo 393 +/- 65 P-value 0.91 A. lumbricoides  Baseline Treatment 9124 +/- 1695 Placebo 12376 +/- 2319 P-value 0.30 | The overall decline may have been caused by both  malaria and low dietary iron intake, as the study occurred  during the peak period of malaria transmission from October  until February, encompassing unusually heavy short rains in  November and December. |
| Beasley_tanbase (unpublished data) | RCT | Tanzania  School based | Proportion females: 0.415 | Weight: 24.9 | Height: 129.5 |  | Hookworm, A. lumbricoides, S.  Haematobium, Weight, height,  concentration of haemoglobin and intensity  of infection with Plasmodium spp. |  |  |  |
| Ebenezer 2013 | cRCT  Intervention  49 schools  Control: 49 schools | Sri Lanka | Proportion females: 0.468 | WAZ: not reported. proportion with low BMI : 0.438 (control) | HAZ: not reported. proportion stunted: 0.294 (control) | Proportion anemic: 16.5 % (control) mean haemoglobin levels  (sd): 12.4(1.3) (control ) | Code transmission test to measure children's attention, haemoglobin levels were estimated, egg count using the  modified kato-katz techinque | Any helminth infection: 25.2%  Hookworm: 5%  Roundworm: 21.2 %  Whipworm: 4.7 % | Nr | Nr |
| Friis 2003 | RCT | Kenya | Proportion female: 51.9% | Weight: NR;  WAZ :-1.11 (95% confidence  interval (95% CI) -1.16, -1.06) | Height: NR; HAZ -0.96 (95% CI  -1.03, -0.89), | Hb at baseline was  123.7 g/l (95% Cl 122.8, 124.6)  Anaemia: 41.4% | Height, Weight, Hb, height-forage  (HAZ) and weight-for-age (WAZ) Z-scores, intestinal helminths (S. mansoni, T. trichiura  and A. lumbricoides) and schistosome  eggs | 14% for A. lumbricoides,  45% for T. trichiura, 55% for hookworm and 71% for S.  mansoni. | NR | residents of the study area were members of the Luo community  and their principal occupations were subsistence  crop farming, raising Zebu cattle, commercial fishing and  petty trade. Malaria was known to be holoendemic in the study area, and intestinal helminths (hookworm (Necator  americanus), Trichuris trichiura and Ascaris lumbricoides) and  Schistosoma mansoni were prevalent, whereas S. haematobium  was not endemic. |
| Hall 2006 | cRCT | Vietnam | Proportion female: NR  Place of origin: Dong Thap province, Vietnam;  SES: nr | Baseline weight: 17.8 kg +/- 2.54 (comparison group)  WAZ: -1.541 +/- 0.907 (comparison group)  Proportion underweight: NR | Height (cm): 121.0 (4.81)  HAZ: NR  Proportion stunted: 29% | NR | Weight, height, height for age, weight for age, weight for height, body mass index, appetite | *A Lumbricoides* : 70.7%  Hookworm: 7.2%  Trichuris: 83.6% | *A Lumbricoides* : 7533 epg  Hookworm: 7 epg  Trichuris: 518 epg | NR |
| Le Thi Huong 2007 | RCT | North Vietnam | Proportion female: 51.2 % (Arm 1) | WAZ: -1.9(0.6) (placebo) proportion underweight (baseline) : 45.1 % (Arm 1); (defined as z-scores <-2 SD (WHO, 1995)) | HAZ: -1.7(0.8) (placebo)  proportion underweight: 31.7 % (Arm 1) (defined as z-  scores <-2 SD (WHO, 1995)) | Proportion anemic: 91.5 % (Arm 1) (defined as: Hb concentration of <115 g/l); Haemoglobin: 107.8  (6.2) (Arm 1) (Mean Hb in g/l -(SD)) | Haemoglobin, prevalence of underweight, stunting and wasting, prevalence of anemia, body iron (plasma ferritin, TfR, CRP, haemoglobinopathies), parasite infection status,  inflammations, and IgE | *A Lumbricoides* : 69.5 %  Hookworm: 11 %  Trichuris: 73.2% | Data on intensity not reported in paper, however, "most infections were 'light' or 'average'  and only 27 % and 2 % among infected children showed severe infection with Ascaris or Trichuris". | Lack of adequate sanitation and safe water supplies, poor sanitation facilities. |
| Liu 2017 (Rozelle 2015) | cRCT  Intervention: 56 townships  Control: 56 townships | China | 43 % female (control group)  Migrant workers: 31%  Mother attended secondary school: 7% | Weight: 28.63 kg  WAZ: nr  Underweight: 24% | Height: 132.95 cm  HAZ: nr  Proportion stunted: 23.48% | Proportion anemic: 16.62%  Haemoglobin: 125.17 g/dL  Vitamin A deficient | STH infection prevalence, stunting and underweight prevalence, working memory index, processing speed index, school attendance, mathematics test scores, infection intensity (fecal egg counts), anemia prevalence (Hb levels), height, weight, HAZ, WAZ, BMI, | *A Lumbricoides* : 30.5%  Trichuris: 23.3%  Hookworm: 1.0%  Any STH: 41.1 % | *A Lumbricoides* : 728.3 epg  Trichuris: 55.9 epg  Hookworm: 17.3 epg | Nr |
| Kirwan 2009 | RCT | Nigeria | proportion female: 195 (50.3%) Socio-economic status  Mean ± SE 6.28 ± 0.06 | Calculated: WgtB: 12.75 | Calculated: HgtB: 89.36 | NR | egg counts, adverse effects | A. lumbricoides : 45.4%  T. trichiura : 3.1 %  Hookworm  :3.9%  S. haematobium: 1.8% | Mean epg ± SE: A. lumbricoides : 1111 ± 149.53  T.trichiura : 1.70 ± 1.05 Hookworm: 3.41 ± 1.75S. haematobium: 0.06 ± 0.02 | no organised  sewage disposal system and refuse and human faeces  are dumped in the bush or burned. Shared source of  water ( community taps and/or wells ) Health care centers inadequately equipped  and lack essential supplies and qualified staff |
| Nga 2009 | RCT | Vietnam | Proportion female: 0.508 | WAZ: -1.56 (0.69) , proportion of underweight: 0.285 | HAZ: -1.41 +/- 0.87. proportion of stunting: 0.251 | Proportion anemic: 23.7 % (placebo) haemoglobin concentration levels: 12.03(0.7)  Vitamin A deficiency: 11.2 % of  the sample | WAZ, HAZ, WHZ, mean MUAC, cognitive function, haemoglobin, worm prevalence, changes in zinc, iodine and ferritin concentrations | Overall helminth prevalence: 92 %  *A Lumbricoides* : 66.7 %  Trichuris: 56.1 %  Hookworm: 4.1% | *A Lumbricoides*  mod-heavy: 4.1%  Hookworm mod-heavy: 12%  *T. Trichiura*mod-  heavy: 8% | Nr |
| Ndibazza 2012 | RCT | Uganda | Proportion female: 48%  Place of origin: rural settings,  education of mothers: none or primary: 54% | 2yrs : WAZ: 20.57 (1.07) (placebo) proportion underweight: not reported. | 2yrs: placebo=20.98 (1.37)  proportion stunted: not reported | haemoglobin levels: 2yrs placebo=11.06 (1.27) | WAZ, HAZ, WHZ, Haemoglobin, cognitive tests, adverse events, death, prevalence of helminth infection, post immunisation recall responses to BCG and tetanus antigens, incidence of malaria, diarrhoea, pneumonia, and eczema, fine motor function and gross motor function | Trichuris: 2.3%, 4.4%, 5.2 % and 5.3 % in 2, 3, 4 and 5 yr olds  *A Lumbricoides* : 1%, 0.9%,0.9 % and 0.5 % in 2, 3, 4, 5 yr olds.  Hookworm: 0.3%, 0.1%, 0.7 % and 0.5 % in 2, 3, 4, and 5 yr olds | Intensity of helminth infection was generally low | Nr |
| Miguel 2004 | cRCT | Kenya | Proportion female: 47%;  Place of origin: farming region, SES: similar socioeconomic characteristics; | WAZ: -1.44 | HAZ:  -1.44 +/- 0.86; Proportion stunted : 25.5 % | Proportion anemic: 4%, haemoglobin: 12.4g/dL | WAZ, HAZ, haemoglobin, malaria, exam score; performance, cognitive tests, school participation, worm prevalence and intensity, self-reported sickness, worm; prevention behaviours: proportion “clean” as per health worker observation, proportion; wearing shoes as per health worker observation, self-reported contact with fresh-water; in past week, access to home latrine, malaria/fever | Overall: 92%  *A Lumbricoides* : 42%  Hookworm: 77%  Trichuris: 55%  Schistosomiasis in schools <5 km from Lake Victoria: 80%  Any STH: 92% | Any worm mod to heavy:37%  *A Lumbricoides* : 16%  Hookworm mod-heavy:15%  *T. Trichiura*mod to heavy:10%, Schistosomiasis mod to heavy:39% | NR |
| Olds 1999 | RCT | China, Philippines and Kenya | proportion female: 0.525 PROGRESS+: NR | 29.6 kg (10.7) | 33.54 cm (20.1) | Initial haemoglobin level: 11.8 (1.8) (placebo) (all values are in g/dL). | Haemoglobin levels, egg counts, anthropometric measurements, side effects | *A Lumbricoides* : 60.2 % Hookworm: 52.1% Trichuris: 81%, Schistosomiasis Japonicum (46%), Mansoni (79%), Haematobium (87%) | Study conducted in an area with generally light infections | There was increase in infection among the control group due to environmental exposures of the toddlers |
| Rohner 2010 | RCT | Côte d'Ivoire | proportion female:42.8%  Socio-economic status: NR | Calculated: WgtB: 27.40 | Calculated: HgtB: 131.04 |  | Haemoglobin levels, plasma ferritin, anemia, iron deficiency, malaria, egg counts, zinc protoporphyrin (ZPP), soluble transferrin receptor (TfR),  C-reactive protein (CRP), and a-1-acid-glycoprotein (AGP). | Any worm: 54.8%  A. lumbricoides : 1.4%  T. trichiura : 2.9 %  Hookworm  : 52.6% | hookworm : 107.8 EPG (95% CI: 92.4, 125.7). Overall,  infection intensity was low; |  |
| Rousham 1994 B | RCT | Bangladesh | NR Occupation: diverse: farmers, business men and professionally employed. | Children of Farmers:  - 2.61, Children of Business Men: - 2.48, Children of other Professionals: - 2.18; underweight: 73% | Male:  -2.76 Female:  -2.99 Children of Farmers:  -2.98, Children of business men:  -2.74, children of other professionals: -2.31 | NR | Height weight, HAZ, WAZ, WHZ, MUAC, worm prevalence and intensity (egg counts) | *A Lumbricoides* : 71%  Hookworm: 10%  T. Trichiura: 44% | Mean intensity was low. No mod-heavy infections. | Clean water was provided by deep tubewells. Very few households had sanitary latrines |
| Solon 2003 | RCT | Philippines | Proportion female: 0.481 | WAZ: -1.70(0.71) | HAZ: -1.83(0.88) | Proportion anemic: 0.52  haemoglobin concentration 11.92(1.23) | Weight, height, haemoglobin, UIE, stool egg count, physical fitness,  heart rate, cognitive ability | Overall: 54 %  *A Lumbricoides* : 43 %  Hookworm: 11%  Trichuris: 22 % | Most of the infections were of  light intensity. | Nr |
| Stoltzfus 1997 | RCT | Zanzibar, Tanzania | Proportion female: 0.512 | Nr | < 10yrs HAZ: 1.44 (1.30) (control) ≥10 HAZ :  2.33 (1.25) (control)  <10yrs, proportion stunted: 0.307 (control) ≥10 proportion stunted was 0.640 (control) | Proportion anemic: 0.623, 22 % <5 % BMI for <10 years, 48 % <5 %  percentile for >10 years  Haemoglobin: 68g/l | Weight, Height, prevalence and intensity of helminth infections, haemoglobin, plasma  ferritin, anemia, severe anemia | *A Lumbricoides* : 73%  Hookworm: 91.2 %  Trichuris: 94.7% | *A Lumbricoides* : 239 epg  Hookworm: 332 epg  Trichuris: 531 epg | 62 % and 52 % had access to safe water supply in the  treatment and control groups respectively. 82 % and 76 % indiscriminately disposed of their children feces in the treatment and control groups  respectively. low percentages hand washed with soap before food, and other activities |
| Stoltzfus 2001 | RCT | Zanzibar | Proportion female: 0.465 | Proportion underweight: 0.345 | Proportion stunted: 0.385 | Proportion anemic: 0.96  Haemoglobin: 86+/-15 gl/L | Anemia , haemoglobin, erythrocyte protoporphyrin, plasma ferritin, prevalence of helminth infection,  motor and language development | *A Lumbricoides* : 42 %  Hookworm: 46 %  Trichuris: 68% | *A Lumbricoides* : 26 epg Whipworm: 57 epg  Hookworm:14 epg | Nr |
| Wiria 2013 | cRCT, households | Indonesia | Proportion female: 53.9 % (placebo). SES: most in the middle percentile; | Nr | Nr | NR | Weight, height (provided by author), adverse effects, allergen response (skin prick test, IgE to aeroallergens), malarial parasitemia | Overall 87.2%  *A Lumbricoides* : 34.9%  Hookworm: 74.5% | *A Lumbricoides* : 23 % high or moderate  Hookworm: 67 % high or moderate  Trichuris: nr |  |
| Yap 2014 | RCT | China | proportion female:51.5% | IPD: Baseline weight (kg): 26.05  Proportion underweight: 3.6% | IPD: Baseline height (cm): 126.85  Proportion stunted: 76.8% |  | Body weight, height, sum of skin folds, Hb levels, physical strength (grip strength, standing broad jump tests), physical fitness (run test, VO2 max), egg count | A. lumbricoides : 93.3%  T. trichiura : 94.2 %  Hookworm  :61.3% | IPD : A. lumbricoides :  T. trichiura : Hookworm |  |

Additional tables 8: Studies which were eligible but did not provide data

| Authors | Study design | Country | Proportion female; Socioeconomic status | Baseline weight (kg); weight-for-age (WAZ); proportion under-weight | Baseline height (cm); height-for-age (HAZ); proportion stunted | Nutritional status | Outcomes measured | Worm prevalence | Worm intensity | Environmental risk for worm infection |
| --- | --- | --- | --- | --- | --- | --- | --- | --- | --- | --- |
| Beach 1999 | RCT | Haiti | Proportion female: 47.7%  no others reported | Baseline weight: 20.8  WAZ: -0.859  Proportion underweight: nr | Baseline height: 117.6  HAZ: -0.770  Proportion stunted: nr | Baseline anemia: mild = 5%; moderate = 0.3%, severe = 0% | Nutritional benefits, prevalence and intensity of intestinal helminth infection post treatment, anthropometric measurements (height, weight, HAZ, WAZ, WHZ), reinfection | *A Lumbricoides* : 29.2 %  Hookworm: 6.9%  Trichuris: 42.2% | 5 % of infections heavy | Nr |
| Donnen 1998 | RCT | Zaire | Proportion female: 0.436 | Weight: NR  WAZ: NR  Proportion underweight: 50.4 % | HAZ: NR  Proportion stunted: 65% | Proportion anemic: nr  Haemoglobin: nr  Retinol deficient: 19.2 to 25.6% | Weight gain, height gain, WAZ, HAZ, WHZ, mid-upper-arm-circumference, fecal egg counts (prevalence and intensity); hematology: levels of retinol, albumin, retinol binding protein (RBP), c-reactive protein (CRP) | *A Lumbricoides* : 10.5 % | Nr | The population settled in the region, most of whom are farmers, live in a poor rural  environment, characterized by subsistence economy, rapid demographic  expansion and rudimentary sanitation. Food supply is constantly poor in energy and periodically poor in protein, depending on the season. Diet very poor in lipids. |
| Dossa 2001 | RCT | Benin | nr | Weight (kg) 12.7 (1.5) (Arm 1-control) | HAZ -2.48 +/- 0.88 (control). proportion stunted:  0.66 (control) | Proportion anemic: 78 % Haemoglobin:  10.2+/-0.9 (control) | Change in weight, height, MUAC, triceps skinfold thickness; haemoglobin level  and eggs per gram of feces | *A Lumbricoides* : 38%  Hookworm: 13%  Trichuris: 47% | *A Lumbricoides* : 19,874 epg  Hookworm 781 epg  *T. Trichiura*1918 epg | Results suggest that reinfection was a continual  process as sanitation and hygiene conditions did not improve throughout study. The living conditions are  characterized by poor sanitation and limited access to tap  water. |
| Fox 2005 | RCT | Haiti | Proportion female: 54.3% | nr | Nr | Nr | Weight, height, WAZ, HAZ, adverse effects (eg headache, fever, stomach pain, etc), egg count in the feces sample, and nutritional benefits if any. | *A Lumbricoides* : 31.7 %  Hookworm: 10.1%  Trichuris: 51.0% | Nr | Nr |
| Garg 2002 | RCT | Kenya | Proportion female: 44 %  Education: 90-93 % of parents had at least primary education,  SES (reported similar socio-economic status, but  no data shown), | Weight: 12.92+/-0.19 WAZ: -1.24 (0.08) Proportion underweight: 28% | HAZ: -1.48 (0.08) | Proportion anemic: 61 % haemoglobin:11.24 +/- 0.11;  Hb concentration <11 g/dL | Mean differences for weight, height, weight for age, height for age and weight for height in SD units; change in haemoglobin concentration; egg counts (intensity). | *A Lumbricoides* : six %  Hookworm: six %  Trichuris:1% | Moderate to heavy infection: 1.6% | Low: most/all of the children had access to a pit latrine (not associated with helminth infections) |
| Greenberg 1981 | RCT | Bangladesh | Proportion female: nr  Gender/sex: comparable  Religion: comparable  Education: comparable  SES: comparable | Weight/WAZ: measured but not reported proportion underweight: 71.4 % (<-2) | baseline stunted: 88.9 % (Arm 1) | Nr | Weight, height, weight-for-age, height-for-age, weight-for-height, triceps skinfold, midarm circumference, triceps-skinfold-for-  age, pot belly, abdominal girth to chest circumference ratio, cure rates, reinfection rates, severity of infection | *A Lumbricoides* : 81%  Hookworm: 5%  Trichuris: 65 % | Moderate to high: 36% | Inadequate sanitation |
| Hadidjaja 1998 | CRT | Indonesia | Proportion female: NR  education of the mother,  60.0% belonged to the lowest level (primary school graduate  or lower), 37.4% belonged to the middle class (secondary  school), and only 1.7% had attained the highest level (university  education). | NR | NR | Mebendazole:Good  94 (84.7)  Mild malnutrition  5 (4.5)  Moderate malnutrition  12 (10.8)  Health education  Good  97 (85.9)  Mild malnutrition  1 (0.9)  Moderate malnutrition  15 (13.3)  Mebendazole plus  health education  Good  64 (86.5)  Mild malnutrition  3 (4.0)  Moderate malnutrition  7 (9.4)  Placebo  Good  155 (83.8)  Mild malnutrition  8 (4.3)  Moderate malnutrition  22 (11.9) | education of the  mother, nutritional status of the  children, intensity of infection with *A.*  lumbricoides and T. trichiura (epg), intelligence  , and the cognitive  functions | Mebendazole 138 (62.6%)  Health education 113 (49.1%) Mebendazole plus  health education 125 (71.4%) Placebo 224 (56.1%) | *A Lumbricoides*  (epg) Mebendazole 2435  Health education 1264 Mebendazole plus  health education 1679 Placebo 1898  *T. Trichiuratrichiura* (epg)  Mebendazole 143  Health education 88 Mebendazole plus  health education 104 Placebo 113 | These are slum areas in which environmental sanitation, as well as personal  hygiene, was poor. Most of the irrigation ditches seemed to be the main  places for defecation. The ditches were full of garbage and  human feces polluted the river water. |
| Hadju 1997 | RCT | Indonesia | Proportion female: 1.05,  Place of origin:  urban slums;  SES: socioeconomic data was not collected | Weight: 18.9 (2.9) kg (Arm 1) | HAZ: -2.08 (0.8) (Arm 1) | Nr | Prevalence and intensity of infection, anthropometric measurements  (weight, height, mid arm circumference, WAZ HAZ, midarm circumference Z score). | *A Lumbricoides* : 93 %  Trichuris: 97% | *A Lumbricoides* : 4,518 epg  *T. Trichiura*2,427 epg | Nr |
| Haque 2010 | RCT | Bangladesh | Proportion Female: Albendazole+β-  Carotene: 48%  Albendazole+  Placebo: 38%  β-carotene+  placebo: 43%  Placebo+  Placebo: 52% | Weight (kg) 1 Albendazole+carotene:12.2+/-0.3 Albendazole+  Placebo: 12.3+/-0.3 β-carotene+  placebo: 12.4+/-0.3 Placebo+  Placebo: 12.4+/-0.3  Weight-for-age (%)Albendazole+carotene: 76.3+/-1.4 Albendazole+  Placebo: 78.4+/-1.5 β-carotene+  placebo: 78.1+/-1.6 Placebo+  Placebo: 79.2+/-1.5 | Height (cm) Albendazole+carotene:90.9+/-1.1 Albendazole+  Placebo: 90.9+/-1.2 β-carotene+  placebo: 91.4+/-1.0 Placebo+Placebo: 91.6+/-1.3  Height-for-age (%)†Albendazole+β-Carotene 90.4+/-0.9 Albendazole+placebo91.7+/-0.9 β-carotene+  Placebo 90.6+/-0.9 Placebo+placebo91.9+/-1.0 | 127 (57.5%) children were vitamin Adeficient  (serum retinol of ≤0.7 μmol/L), of which  15 (6.8%) were severely deficient (<0.35 μmol/L).  Over 95% of the children (211 of 221) had serum  β-carotene of <0.35 μmol/L. The vitamin A-deficient  children were equally distributed into four  groups (data not shown). | A. lumbricoides,  *Trichuris trichiura*, and hookworm prevalence, intensity, height, weight, nutritional status | 67% of the children had  light infection (<4,999 epg), 33% had moderate  infection (5,000-49,999 epg) Ninety-six percent of the  children were co-infected with *T. trichiura;* 56% of  these had light co-infections with *T. trichiura* (1-999  epg)  hookworm: <1%) | *A Lumbricoides*  *Lumbricoides*(epg) Albendazole+  β-carotene3854+/-340  Albendazole+  placebo 4923+/-551 β-carotene+placebo 4853+/-474 Placebo+  placebo 4689+/-426  Trichuris  *trichiura* (epg) Albendazole+  βcarotene1082+/-159 Albendazole+  placebo 1311+/-188 β-carotene+placebo 1310+/-136  Placebo+  Placebo 1571+/-194 | a population of about one million in an area  of 59 sq km. The population of Mirpur is stable  with low socioeconomic conditions which is similar  to other parts of Dhaka city. The average income  is Tk 4,200 (about US$ 68) per month per family.  Twenty-five percent of fathers and 15% of mothers  have more than five years of formal education |
| Henry 1988 | CRT | St. Lucia | Proportion female: 51.6 % | NR | NR | NR | Weight, height, *A Lumbricoides* , Trichuis | *A Lumbricoides* : M(26.1%), F(21.9%) Trichuis: M(16.7%), F(18.3%) |  | Crowding  (persons/room)Income/person(SECC)’  Water/person/day  (litres)  Family size  Weight gain, kg (during  interval 3)  Height gain, cm(during interval 3) |
| Jinabhai 2001 A | RCT | South Africa | Place of origin: rural;  SES: low SES; | Weight: Arm 1 = 26.8kg (SD 3.5) WAZ: 0.7 % or one child (<-2 SD); virtually none underweight (7%) | HAZ: 9.5 % or 13 children (<-2 SD); stunted: 6.8% | vitamin A deficiency found in 34.7 % of the children (retinol <0.70) | Weight, height, % stunted, % underweight, worm burden, prevalence and intensity of helminth infection | *A Lumbricoides* :28.8%  Hookworm: 3.1%  Trichuris: 53.7%  Schistosomias: 24.5% | Mod to heavy infection: 40.3% | Nr |
| Jinabhai 2001 B | RCT | South Africa | Nr | Mean Weight (kg): 26.89 +/- 3.75; underweight: 0.8 % | Height (cm): 127.73 +/- 5.87;  stunted: 7.3% | Proportion anemic: 15.5 % (Hb<120g/L); Haemoglobin:  128.09+/1.10 (control)  vitamin A deficiency: 34.7 % of the children (retinol <0.70); | Serum albumin, serum retinol, haemoglobin, haematocrit, plasma ferritin,serum iron and percentage  transferrin saturation).  Helminthic infections (prevalence and intensity).  Nutritional status. Weight, height and knee–heel length.  Scholastic and cognitive tests. | *A Lumbricoides* : 28.8 %  Hookworm: 3.1 %  Trichuris: 53.7 %  Schistosomiasis: 24.5% | Mod to heavy infection: 40.3 % | Only 50 % of children had access to portable  water at home |
| Kloetzel 1982 | RCT | Brazil | Proportion female: nr  Place of origin: rural communities  SES: children were from poor  socioeconomic | nr | nr | after 10 months of treatment nutritional status improved more than it deteriorated for both the experimental and  control groups | Weight (for undernourishment-number improved/ deteriorated), length, head, chest and mid-arm circumference, and triceps skin fold. | *A Lumbricoides* : 53%  Trichuris: 20%  Hookworm: 8% | Mod to heavy infection: 18% | Nr |
| Koroma 1996 | RCT | Sierra Leone | nr | proportion underweight: rural -1.17+/0.21  urban: -1.07+/0.77 | mean stunted: rural: -1.68+/0.30  urban: -1.14+/0.09 | Nr | WAZ, HAZ, WHZ, egg counts (prevalence and intensity) | *A Lumbricoides*  urban: 32%; Rural: 46%  Hookworm: urban: 10%; Rural: 25%  Trichuris: urban:  65%; Rural: 1%  Schistosomias: urban: 6%; rurual: 14% | *A Lumbricoides* : 2278epg Hookworm: 588 epg  Trichuris: 262epg | Nr |
| Lai 1995 | RCT | Malaysia | Proportion female: 60.5%;  Place of origin: urban/periurban,  Ethnicity: same ethnic groups  (eating similar food),  Education: similar mother's education,  SES: similar SES | Weight: 19.12kg (Arm 1)  WAZ-underweight: 29.35 | HAZ: 15.6 % (male and female both)  Stunted: 14.35 (Arm 1) | Nr | Weight and height (weight for age, height for age, weight for height), worm prevalence, eggs/g feces | *A Lumbricoides* : 66 %  Hookworm: 5 %  Trichuris: 69% | Mod to heavy *A Lumbricoides* : 26%  Mod to heavy trichuris: 34 % | Nr |
| Michaelsen 1985 | CBA | Botswana | Nr | Not mentioned | nr | anaemia: the lowest value: 9.6 g/100 ml., haemoglobin baseline: 12.91 g/ml (control) | Haemoglobin, weight, height, weight-for-height, egg counts. | Hookworm: 86% | Mod to heavy infections: 1% | Nr |
| Ostwald 1984 | RCT | Papua New Guinea | Proportion female: nr  Place of origin: central highlands of Papua New Guinea  SES: nr | Baseline weight: 27.8 kg (control)  WAZ and  proportion underweight: nr | Baseline height: 126.6 cm (control)  HAZ and  proportion stunted: nr | Proportion anemic: nr  Haemoglobin baseline: 14.2 g/dl | Weight, height, weight for height, haemoglobin, ferritin, transferrin, serum folate, serum ascorbate, parasite prevalence. | *A Lumbricoides*  67 %  Hookworm 92 %  *T. Trichiura*64% | Majority light infections | Few public taps served as water supply, non-hygienic as there were few public toilets hence defecation on the streets. |
| Pust 1985 | Cluster CBA | Papua New Guinea | Nr | Nr | nr | Proportion anemic: 5 % | Anthropometry (weight, mid-upper arm circumference, triceps skin fold and length), haematocrit, vitamin A and serum albumin analysis, morbidity and mortality | *A Lumbricoides*  63 % Hookworm 60% *T. Trichiura*37% | Heavy infections 30% |  |
| Stephenson 1989 | RCT | Kenya | Proportion female: 50-51 % per group  Place of origin: Kwale district, Coast  Province, Kenya | Baseline weight (kg): 21.8+/-0.50 (placebo)  %WAZ: 74.2+/- 1.19 (placebo  Proportion underweight: nr  ) | Baseline height (cm): 122.3+/-0.95 (placebo)  %HAZ: 91.5 +/-  0.54 (placebo)  Proportion stunted: nr | Nr | Weight, % weight for age, height, % height for age, % weight for height, MUAC, %MUAC for age, triceps skinfold thickness, % triceps for age, subscapular skinfold thickness, % subscap for age, Harvard step test,  prevalence and mean egg counts | *A Lumbricoides* : 49%  Hookworm: 87%  Trichuris: 97 % | *A Lumbricoides* : 32044epg Hookworm: 2795epg  Trichuris: 10234epg | Nr |
| Taylor 2001 | RCT | South Africa | Nr | WAZ: -0.62(0.86) proportion underweight was not recorded | HAZ: -0.60(1.16). Proportion stunted was not recorded | Haemoglobin: 12.53g/dL, (group 1) | Height, weight, blood count, anemia (haemoglobin concentration), urine analysis, helminth infection prevalence | *A Lumbricoides* : 55.9%  Hookworm: 59.4 %  Trichuris: 83.6%  Schistosomiasis: 44 % with blood in the urine | Most pupils had light infections. | Second half of study carried out during rainy season  when people collect rain from roofs; and water taps were not working during study |
| Watkins 1996A&B | RCT | Guatemala | Proportion female: not reported  Education: parental education: 0.42 (se 0.03)- scale not given | Weight: 23.37 (0.36 se); WAZ: -1.66 (0.06 se);  Proportion underweight: not reported | Height: 119.58 (se 0.72); HAZ: -2.71 (se 0.09);  Proportion stunted: nr | Nr | Worm burden, weight, height, WAZ, HAZ, WHZ, mid-upper arm circumference, cognitive tests (vocabulary (InterAmerican vocabulary test), reading (InterAmerican reading test), Peabody picture vocabulary test), Attendance by teacher's attendance books, information  processing (different paper) | *A Lumbricoides* : 91 %  Trichuris: 82 %  Hookworm: 0% | *A Lumbricoides*  heavy: 45%.  *T. Trichiura*heavy: 36 % | Nr |
| Willett 1979 | RCT | Tanzania | nr | Baseline weight: 11.45 (SD 0.25) | Baseline height: 87.56 (SD 0.99) | Nutritional status, expressed as the ratio of observed over expected weight for age, baseline  mean = 0.79 | weight, length, *A Lumbricoides*  prevalence | *A Lumbricoides* : 53%  Hookworm: 10.9% | Nr | Nr |

Additional tables 9: Studies included in 2016 Campbell review of aggregate data but excluded from this IPD systematic review

| Authors | Reason for exclusion | Study design | Age | Country | Proportion female; Socioeconomic status | Baseline weight (kg); weight-for-age (WAZ); proportion underweight | Baseline height (cm); height-for-age (HAZ); proportion stunted | Nutritional status | Outcomes measured | Worm prevalence | Worm intensity | Environmental risk for worm infection |
| --- | --- | --- | --- | --- | --- | --- | --- | --- | --- | --- | --- | --- |
| Alderman 2006 | No intensity data collected | cRCT  intervention: 25 parishes; 14 940 children  control: 25 parishes;  13 055  children | 1 and 7 years | Uganda | Proportion female: 50%  SES: | Baseline weight: NR  Mean WAZ (control group): -1.17 (SD 1.45)  Proportion underweight: 26% | NR | Proportion anemic: nr; Haemoglobin: nr  vitamin A/retinol: nr | Weight gain (Alderman)  Long-term math, English, height (Croke 2014) | Overall: 55.9%  *A Lumbricoides* : 17.5%  Hookworm: 44.5%  Trichuris: 7.3% | Not reported | "Controlled for using multivariate regression models" |
| Awasthi 2000 | No infection intensity collected | RCT  32 slums 1061 children (enrolled) | 1.5 to 3.5 years | India | Proportion Female: 48%;  Place of origin:  Education: illiterate mother: 80.3%  Religion: 67 % Hindus; 33 Percentage Muslims  SES < 1000 rupees/month: 94.4% | Baseline weight: 10.2 (SD 2.1);  WAZ: NR  Proportion underweight: 66.3% | Baseline height: 81.7 (SD 8.7);  HAZ: NR  Proportion stunted: 54.77% | Proportion Anemic: 91.1 % (less than 11g/dl);  Haemoglobin baseline: 9.5 (SD 0.9)  Vitamin A: NR | Number underweight, number stunted, weight (kg), height (cm), haemoglobin (g/dl), development (R-PDQ-Denver Questionnaire), cost per child prevented from becoming underweight.  Not used: illness episodes | *A Lumbricoides* : 11-13% | Not reported | Field defecation: 52.5% |
| Awasthi 2001 | No infection intensity collected | cRCT  124 slums;  2,010 children | 0.5-1 year | India | Proportion female: 49%  SES: Family income below poverty line,  Setting: suboptimal living conditions | Baseline weight: 7.03 (0.15);  WAZ: -1.99 (SE 0.13),  Proportion underweight: 47.1% | Baseline height: 64.9 (SE 3.25)  HAZ: -2.44 (SE 0.2) Proportion stunted: 59.7% | NR | Weight, height, WAZ, HAZ, WHZ, number underweight, number stunted, number wasted | *A Lumbricoides* : 9% | NR | Suboptimal living conditions |
| Awasthi 2008 | No infection intensity collected | cRCT  50 slums; 3,935 children | 1 to 5 years | India | Proportion female: 51%,  Place of origin: Lived in slums | Baseline weight: 11.6 | Baseline height: 85.2 | NR | Height and weight gain | NR | NR | Government defined slum areas |
| Awasthi 2013 (DEVTA articles) | No infection intensity collected | cRCT  72 mainly rural administrative blocks; >1,000,000 children | 1·0–6·0 years | India | Proportion female: 50% | Baseline weight: 11.05  Proportion underweight: NR | Baseline height: 81.6 | Haemoglobin level: 99.4 | Helminth egg count, weight gain, deaths, height gain, BMI, haemoglobin, illness in past four weeks | *A Lumbricoides*  midstudy: 27%;  Hookworm: midstudy:8.0%; | *A Lumbricoides*  96 epg  Hookworm 83 epg | NR |
| Bhoite 2012 a& B | No infection intensity collected | cRCT  3 schools; 496 children | NR | India | NR | Weight (kg): 23.7 (4.8)  Proportion underweight: 50.9 % | Baseline height: 131.4 cm (SD 9.1)  Proportion stunted: 26.7% | Proportion anemic: 90.6  Haemoglobin:10.5 +/- 1.4 g/dL | Weight, height, weight for age, height for age, BMI, haemoglobin levels and physical work capacity (number of steps) | NR | NR | NR |
| Bobonis 2006 | No infection intensity collected and did not follow the same children | cRCT  Intervention: 59 clusters; 930 children  Control: 96 clusters; 1462 children | 2-6 years | India | Proportion female: 55 %  Occupation: Mother Housework: 78%. Father Labourer: 35 %  Religion: Hindu: 75%, Muslim 25%  Education: Mother: education level (years): 3.3; father 5.8; | WAZ: -1.02  Proportion underweight. 30 % | HAZ:  -0.45  Proportion stunted: 24 % | Proportion anemic: 7 % (severely) and 41 % (moderately)  69 % of children in group 1 had anemia.  Mean haemoglobin: 9.95g/dL in group 1 | WHZ, WAZ, HAZ, weight, height, BMI, haemoglobin, school participation | Overall: 30%  *A Lumbricoides* : 21 % | NR | Reinfection deemed likely |
| Gateff 1972 | No infection intensity collected | RCT  584 students | 6-15 years | Cameroon | 212 female, 180 male  -very poor hygiene, poor sanitation (open excretion), shoes rare, poor hygiene | Not reported | NR | NR | Weight (difference), School notes (difference), attendance (difference) | Any parasite: 80%  (mostly *A Lumbricoides* , ankylostoma duodenale (hookworm), or strongyloides) | NR, hyperendemic area | High: almost no access to toilets, poor hygiene, poor sanitation (open excretion) |
| Goto 2009 | Does not have baseline infection intensity | RCT  394 infants | 3-11 months | Bangladesh | Proportion female: 20 % (FSA)  Occupation-FSA grp: (fathers) None or daily income: 15 % (mother) household: 32 % Education-FSA grp: (father's) None: 17%; (mother's) None: 14%; SES: Housing condition:  Poor= 5 % | WAZ: -1.95  Proportion underweight: 48 % of infants | HAZ:  -1.22  Proportion stunted: 23 % of infants | Proportion anemic: 96 % at baseline, 82 % at end of study  Haemoglobin: 91.9+/-12.2 | Haemoglobin, HAZ, WAZ, WHZ, plasma albumin, IgG, Alpha-1-acid glycoprotein, Giardia-specific IgM titre, lactulose/mannitol ratio, prevalence of Giardia-specific IgM titre, prevalence of Giardia cysts, prevalence of Ascaris/Trichuris, prevalence of Intestinal mucosal damage, prevalence of Anaemia | *A Lumbricoides* : 1%  Trichuris: 1% | NR | NR |
| Gupta 1977 | Not an RCT | CBA  154 children | 4-48 months | India | Proportion female: NR;  SES: comparable | baseline weight ( % WAZ): 71.15, (group 1) | baseline height ( % HAZ): 85.36 (group 1) | Haemoglobin: NR  Nutritional status improved: 0.372 (16//43) (placebo) | Weight, change in nutritional status, presence of *A Lumbricoides*  in stools | *A Lumbricoides* : 60% | NR | NR |
| Gupta 1982 | Infection intensity not reported | RCT  159 children | 24-61 months | Guatemala | Proportion female: nr  Place of origin: Santa Maria Cauque village in Guatemala; SES:comparable | Baseline weight (%WAZ): 71.15+/-7.57 (group 1)  Proportion underweight: NR | Baseline height (%HAZ): 85.36 (group 1)  Proportion stunted: NR | NR | Height, weight, %height for age, % weight for age, % weight for height, slope of height on age, slope of weight on age, ascariasis prevalence, giardiasis prevalence | *A Lumbricoides* : 60% | NR | NR |
| Joseph 2015 | Infection intensity only collected at endline | RCT  1760 children | 12-24 months | Peru | Proportion female:45.5%; n Maternal secondary education: 32%  Employment outside the home: 8%  Peri-urban or rural residence: 91%; Earth or wood house: 76% | Weight: 8.7 kg (0.9) | Height: 72.2 cm (2.5) | NR | Weight, height, WAZ, HAZ, adverse events, mortality, Bayley Scales of Infant and Toddler Development, Third Edition  (Bayley-III) | *A Lumbricoides* : 20.2%  Hookworm: 1.5%  Trichuris: 10.8%  Schistosomiasis: NR | No heavy infections | NR |
| Kaba 1978 | Not an RCT | CBA  176 children | 6-11 years | Zaire | Proportion female: 61.7 %  PROGRESS+: NR | Baseline weight: 20.01 kg (range 10-30)  Weight: 20 kg, sd 5 kg | not done | Proportion anemic: 63 % (defined as <12 g percent haemoglobin)Baseline Haemoglobin: 11.73g p.cent | Weight, haemoglobin, parasite load | Any infection: 85 % *A Lumbricoides* : 40%;  Hookworm: 43%  Trichuris: 68%  Schistosomiasis: 1.1% | NR | NR |
| Kruger 1996 | No intensity of infection | RCT for deworming vs. placebo  cRCT for iron vs. no iron, with and without deworming;  178 children | 6-8 years | South Africa | Proportion female: nr;  Ethnicity: mixed ethnic origin (European, African, Malay); | Weight: 19.1 (2.6) (low Iron) 19.6 (2.3) (adequate iron) p= .2264 | Height 113.8 (5.2) (low Iron) 115.5 (4.6) (adequate iron) p= 0.432 | Proportion anemic: 0.235 or 0.425 according to WHO  Haemoglobin: Mean SD p(g/dl) 11.7g/dl (0.9)  Arm 1 | Weight, height, WAZ, HAZ, WHZ, haemoglobin, iron status (MCV, serum ferritin, MCH, TIBC, TS, WCC), attendance (school records), egg counts | Overall: 58.7%  *A Lumbricoides* : 20.0 %  Trichuris: 38.1 %  Any STH infection: 58.7% | NR | NR |
| Linnemayr 2011 | no intensity of infection | cRCT  212 villages; 4296 children | < 3 years | Senegal | Gender, parent’s occupation and level of education are presented as regression coefficients and not proportions. | WAZ: -1.317 (1.417) proportion underweight not reported | HAZ and proportion stunted not reported | NR | Weight for age Z scores | NR | NR | Poor sanitation with limited piped water/latrines |
| Reddy 1986 | no intensity of infection | RCT  517 children | 1-5 years | India | Place of origin: rural residence, Ethnicity: majority muslims,  Occupation: dependent on rickshaw pulling or casual labor, some have clerical jobs  SES: low socio-economic strata. | NR | NR | Vitamin A Deficiency: 13 % and 45 % had low levels of vitamin A | Height and weight, serum vitamin A, *A Lumbricoides*  prevalence | *A Lumbricoides* : 35% | NR | High, 5 % drink boiled water only, 65 % use feces as fertilizer, 84 % washes hands before eating, 87 % washes hands after toilet, |
| Sur 2005 | no intensity of infection | RCT  702 children | 2-5 years | India | NR | Baseline weight ~11 kg (only graphically presented) | NR | NR | Weight gain, *A Lumbricoides*  prevalence, diarrhoeal episodes | *A Lumbricoides* : 51.8% | NR | NR |

Additional tables 10: List of all analysis

| Model |  | weight | height | haemoglobin |
| --- | --- | --- | --- | --- |
| COLLAPSED base case model, adjusted (6 nodes, 14 studies) |  | . | . | . |
| base case, imputed, no covariates, k=14, 6 nodes, unadjusted |  | . | . | . |
| Full model, imputed, with 18 nodes, 14 studies, adjusted |  | . | . | . |
| complete case, k=14, 6 nodes, unadjusted |  | . | . | . |
| include missing case/datasets (k=19), no covariates and no missing data (6 nodes) |  | . | . | . |
| low risk of bias on allocation concealment (Ebenezer, Liu, Nga, Rohner, Yap, Ndibazza), 6 nodes) |  | . | . | . |
|  |  |  |  |  |
| **Effect modifiers - base case (k=14)** | **levels** | **weight** | **height** | **haemoglobin** |
| intensity (anyworm)-categories 0,1,2 | 3 | . | . | . |
| age (<5 yrs, => 5 years) | 2 | . | . | . |
| HAZ (<-2, =>-2) | 2 | . | . | . |
| BMI for age, <-2, =>-2) | 2 | . | . | . |
| Anemia (y/n) | 2 | . | . | . |
| hookworm, 0,1, 2 | 3 | . | . | . |
| ascaris, 0,1,2 | 3 | . | . | . |
| trichuris, 0,1,2 | 3 | . | . | . |
| sex , m/f | 2 | . | . | . |
|  |  |  |  |  |
|  |  | Weight | height | haemoglobin |
| **Direct evidence of aggregate level data (6 nodes,s)** |  | **adjusted** |  |  |
| STH vs placebo (Stoltzfus 2004, Stoltzfus 1997, Liu, Yap, Nga, Solon, Olds, Huong, Ndibazza) | pooled estimate | . | . | . |
| Huong_2vs1 |  | . | . | . |
| Liu_2vs1 |  | . | . | . |
| Nga_2vs1 |  | . | . | . |
| Olds_2vs1 |  | . | . | . |
| Stoltzfus 2004_2vs1 |  | . | . | . |
| Stoltzfus 1997_2vs1 |  | . | . | . |
| Yap_2vs1 |  | . | . | . |
| Ndibazza_2vs1 |  | . | . | . |
| STH+MCN/iron vs placebo (Nga, Solon, Ebenezer, Stoltzfus 2004, Huong) | pooled estimate | . | . | . |
| Nga_5vs1 |  | . | . | . |
| Solon_5vs1 |  | . | . | . |
| Ebenezer_5vs1 |  | . | . | . |
| Stoltzfus 2004_5vs1 |  | . | . | . |
| Huong_5vs1 |  | . | . | . |
| PZQ alone or with STH vs placebo (Olds, Beasley, Friis, Rohner, Tanbase (Beasley)) | pooled estimate | . | . | . |
| Olds_3vs1 |  | . | . | . |
| Beasley_3vs1 |  | . | . | . |
| Friis_3vs1 |  | . | . | . |
| Rohner_3vs1 |  | . | . | . |
| Tanbase (Beasley)_3vs1 |  | . | . | . |
| PZQ with iron/MCN (Friis, Rohner, Tanbase (Beasley)) | pooled estimate | . | . | . |
| Friis_4vs1 |  | . | . | . |
| Rohner_4vs1 |  | . | . | . |
| Tanbase (Beasley)_4vs1 |  | . | . | . |
| MCN/iron vs placebo (Huong, Rohner, Stoltzfus 2004, Friis, Nga, Solon ) | pooled estimate | . | . | . |
| Huong_6vs1 |  | . | . | . |
| Rohner_6vs1 |  | . | . | . |
| Stoltzfus 2004_6vs1 |  | . | . | . |
| Friis_6vs1 |  | . | . | . |
| Nga_6vs1 |  | . | . | . |
| Solon_6vs1 |  | . | . | . |
| STH vs iron/MCN (Nga, Solon, Huong, Stoltzfus 2004) | pooled estimate | . | . | . |
| Nga_2vs6 |  | . | . | . |
| Solon_2vs6 |  | . | . | . |
| Huong_2vs6 |  | . | . | . |
| Stoltzfus 2004_2vs6 |  | . | . | . |
| STH+MCN/iron vs iron/MCN (Nga, Solon, Huong, Stoltzfus 2004) | pooled estimate | . | . | . |
| Nga_5vs6 |  | . | . | . |
| Solon_5vs6 |  | . | . | . |
| Huong_5vs6 |  | . | . | . |
| Stoltzfus 2004_5vs6 |  | . | . | . |

*Note: All cells with a “.” Indicate an analysis that was performed. Comparisons are denoted with codes: 1-placebo, 2: STH, 3: PZQ alone or with STH, 4: PZQ with iron/MCN, 5: STH+MCN/iron, 6: MCN/iron

Additional tables 11: Results from IPD NMA model, 14 studies, 6 nodes, adjusted for covariates, base case analyses for weight, height and haemoglobin

| Main analyses with covariates (adjusted) | | weight | height | haemoglobin |
| --- | --- | --- | --- | --- |
| treatment | comparison | MD (95% CI) | MD (95% CI) | MD (95% CI) |
| STH deworming with any drug | Placebo or control | 0.01(-0.08,0.11) | 0.09(-0.08,0.27) | 0.32(-0.63,1.26) |
| Praziquantel alone or in combination with STH deworming | Placebo or control | 0.04(-0.11,0.19) | -0.06(-0.31,0.18) | **1.85(0.53,3.18)** |
| Praziquantel alone or in combination with STH deworming with iron or micronutrients | Placebo or control | -0.03(-0.27,0.21) | -0.06(-0.38,0.26) | **2.72(1.05,4.40)** |
| Any STH deworming with micronutrients or iron | Placebo or control | -0.02(-0.15,0.12) | -0.03(-0.27,0.22) | **1.98(0.74,3.21)** |
| Micronutrients or iron alone | Placebo or control | -0.02(-0.16,0.12) | -0.07(-0.30,0.15) | **1.28(0.07,2.49)** |
| Praziquantel alone or in combination with STH deworming | STH deworming with any drug | 0.03(-0.14,0.20) | -0.16(-0.43,0.11) | 1.54(-0.03,3.11) |
| Praziquantel alone or in combination with STH deworming with iron or micronutrients | STH deworming with any drug | -0.04(-0.30,0.22) | -0.16(-0.50,0.18) | **2.41(0.51,4.30)** |
| Any STH deworming with micronutrients or iron | STH deworming with any drug | -0.03(-0.17,0.11) | -0.12(-0.37,0.13) | **1.66(0.33,2.99)** |
| Micronutrients or iron alone | STH deworming with any drug | -0.03(-0.18,0.12) | -0.17(-0.40,0.07) | 0.96(-0.39,2.32) |
| Praziquantel alone or in combination with STH deworming with iron or micronutrients | Praziquantel alone or in combination with STH deworming | -0.07(-0.31,0.17) | 0.00(-0.33,0.33) | 0.87(-0.99,2.72) |
| Any STH deworming with micronutrients or iron | Praziquantel alone or in combination with STH deworming | -0.06(-0.25,0.13) | 0.04(-0.28,0.36) | 0.12(-1.63,1.87) |
| Micronutrients or iron alone | Praziquantel alone or in combination with STH deworming | -0.06(-0.24,0.12) | -0.01(-0.29,0.27) | -0.58(-2.08,0.92) |
| Any STH deworming with micronutrients or iron | Praziquantel alone or in combination with STH deworming with iron or micronutrients | 0.01(-0.26,0.28) | 0.04(-0.34,0.41) | -0.74(-2.76,1.27) |
| Micronutrients or iron alone | Praziquantel alone or in combination with STH deworming with iron or micronutrients | 0.01(-0.22,0.24) | -0.01(-0.35,0.32) | -1.44(-3.31,0.43) |
| Micronutrients or iron alone | Any STH deworming with micronutrients or iron | 0.00(-0.16,0.16) | -0.05(-0.31,0.21) | -0.70(-2.11,0.72) |

*bolded numbers indiciate statistically significant effects

**This table provided all 15 comparisons in the network

Additional tables 12: Sensitivity analyses for IPD-NMA model

**Unadjusted main analyses – 14 studies, 6 nodes**

| Unadjusted analyses | | MD (95% CI) | | |
| --- | --- | --- | --- | --- |
| treatment | comparison | weight | height | haemoglobin |
| STH deworming with any drug | Placebo or control | 0.01(-0.08,0.11) | 0.09(-0.08,0.27) | 0.32(-0.63,1.26) |
| Praziquantel alone or in combination with STH deworming | Placebo or control | 0.04(-0.11,0.19) | -0.06(-0.31,0.18) | 1.85(0.53,3.18) |
| Praziquantel alone or in combination with STH deworming with iron or micronutrients | Placebo or control | -0.03(-0.27,0.21) | -0.06(-0.38,0.26) | 2.72(1.05,4.40) |
| Any STH deworming with micronutrients or iron | Placebo or control | -0.02(-0.15,0.12) | -0.03(-0.27,0.22) | 1.98(0.74,3.21) |
| Micronutrients or iron alone | Placebo or control | -0.02(-0.16,0.12) | -0.07(-0.30,0.15) | 1.28(0.07,2.49) |
| Praziquantel alone or in combination with STH deworming | STH deworming with any drug | 0.03(-0.14,0.20) | -0.16(-0.43,0.11) | 1.54(-0.03,3.11) |
| Praziquantel alone or in combination with STH deworming with iron or micronutrients | STH deworming with any drug | -0.04(-0.30,0.22) | -0.16(-0.50,0.18) | 2.41(0.51,4.30) |
| Any STH deworming with micronutrients or iron | STH deworming with any drug | -0.03(-0.17,0.11) | -0.12(-0.37,0.13) | 1.66(0.33,2.99) |
| Micronutrients or iron alone | STH deworming with any drug | -0.03(-0.18,0.12) | -0.17(-0.40,0.07) | 0.96(-0.39,2.32) |
| Praziquantel alone or in combination with STH deworming with iron or micronutrients | Praziquantel alone or in combination with STH deworming | -0.07(-0.31,0.17) | 0.00(-0.33,0.33) | 0.87(-0.99,2.72) |
| Any STH deworming with micronutrients or iron | Praziquantel alone or in combination with STH deworming | -0.06(-0.25,0.13) | 0.04(-0.28,0.36) | 0.12(-1.63,1.87) |
| Micronutrients or iron alone | Praziquantel alone or in combination with STH deworming | -0.06(-0.24,0.12) | -0.01(-0.29,0.27) | -0.58(-2.08,0.92) |
| Any STH deworming with micronutrients or iron | Praziquantel alone or in combination with STH deworming with iron or micronutrients | 0.01(-0.26,0.28) | 0.04(-0.34,0.41) | -0.74(-2.76,1.27) |
| Micronutrients or iron alone | Praziquantel alone or in combination with STH deworming with iron or micronutrients | 0.01(-0.22,0.24) | -0.01(-0.35,0.32) | -1.44(-3.31,0.43) |
| Micronutrients or iron alone | Any STH deworming with micronutrients or iron | 0.00(-0.16,0.16) | -0.05(-0.31,0.21) | -0.70(-2.11,0.72) |

**Complete case analyses – 14 studies, 6 nodes**

| Unadjusted analyses | | MD (95% CI) | | |
| --- | --- | --- | --- | --- |
| treatment | comparison | weight | height | haemoglobin |
| STH deworming with any drug | Placebo or control | 0.03(-0.07,0.13) | 0.06(-0.24,0.35) | 0.30(-0.69,1.29) |
| Praziquantel alone or in combination with STH deworming | Placebo or control | 0.13(-0.04,0.29) | -0.49(-1.17,0.19) | 2.06(0.57,3.56) |
| Praziquantel alone or in combination with STH deworming with iron or micronutrients | Placebo or control | 0.10(-0.09,0.28) | -0.55(-1.27,0.17) | 1.76(0.08,3.45) |
| Any STH deworming with micronutrients or iron | Placebo or control | 0.00(-0.21,0.21) | 0.19(-0.68,1.06) | 3.18(1.28,5.09) |
| Micronutrients or iron alone | Placebo or control | -0.04(-0.26,0.19) | 0.13(-0.77,1.04) | 2.88(0.81,4.95) |
| Praziquantel alone or in combination with STH deworming | STH deworming with any drug | -0.13(-0.35,0.09) | 0.68(-0.23,1.59) | 1.12(-0.88,3.11) |
| Praziquantel alone or in combination with STH deworming with iron or micronutrients | STH deworming with any drug | -0.02(-0.16,0.13) | -0.04(-0.53,0.46) | 1.83(0.44,3.22) |
| Any STH deworming with micronutrients or iron | STH deworming with any drug | -0.05(-0.20,0.11) | -0.09(-0.63,0.45) | 1.53(0.04,3.01) |
| Micronutrients or iron alone | STH deworming with any drug | -0.14(-0.35,0.07) | 0.45(-0.36,1.27) | -0.24(-2.18,1.71) |
| Praziquantel alone or in combination with STH deworming with iron or micronutrients | Praziquantel alone or in combination with STH deworming | -0.01(-0.26,0.23) | -0.22(-1.20,0.75) | -1.35(-3.61,0.91) |
| Any STH deworming with micronutrients or iron | Praziquantel alone or in combination with STH deworming | 0.00(-0.14,0.14) | -0.11(-0.68,0.46) | 1.08(-0.23,2.38) |
| Micronutrients or iron alone | Praziquantel alone or in combination with STH deworming | -0.03(-0.19,0.12) | -0.16(-0.76,0.44) | 0.78(-0.66,2.21) |
| Any STH deworming with micronutrients or iron | Praziquantel alone or in combination with STH deworming with iron or micronutrients | -0.13(-0.32,0.06) | 0.39(-0.40,1.17) | -0.99(-2.71,0.73) |
| Micronutrients or iron alone | Praziquantel alone or in combination with STH deworming with iron or micronutrients | 0.00(-0.22,0.22) | -0.29(-1.20,0.62) | -2.11(-4.11,-0.11) |
| Micronutrients or iron alone | Any STH deworming with micronutrients or iron | 0.01(-0.16,0.18) | -0.07(-0.73,0.59) | -0.75(-2.34,0.83) |

**Complete case analysis – 19 studies, 6 nodes**

| Unadjusted analysis | | MD (95% CI) | | |
| --- | --- | --- | --- | --- |
| treatment | comparison | weight | height | haemoglobin |
| STH deworming with any drug | Placebo or control | 0.01(-0.11,0.12) | 0.06(-0.19,0.31) | 0.37(-0.77,1.51) |
| Praziquantel alone or in combination with STH deworming | Placebo or control | 0.17(-0.04,0.37) | -0.49(-1.10,0.12) | 2.14(0.38,3.90) |
| Praziquantel alone or in combination with STH deworming with iron or micronutrients | Placebo or control | 0.01(-0.28,0.29) | 0.19(-0.59,0.97) | 3.25(1.01,5.49) |
| Any STH deworming with micronutrients or iron | Placebo or control | -0.01(-0.18,0.16) | -0.03(-0.43,0.37) | 1.83(0.05,3.61) |
| Micronutrients or iron alone | Placebo or control | 0.00(-0.17,0.18) | -0.10(-0.53,0.33) | 1.13(-0.44,2.70) |
| Praziquantel alone or in combination with STH deworming | STH deworming with any drug | 0.16(-0.06,0.38) | -0.56(-1.20,0.09) | 1.77(-0.20,3.74) |
| Praziquantel alone or in combination with STH deworming with iron or micronutrients | STH deworming with any drug | 0.00(-0.30,0.30) | 0.13(-0.68,0.93) | 2.88(0.45,5.31) |
| Any STH deworming with micronutrients or iron | STH deworming with any drug | -0.01(-0.20,0.17) | -0.09(-0.53,0.35) | 1.46(-0.39,3.30) |
| Micronutrients or iron alone | STH deworming with any drug | 0.00(-0.19,0.18) | -0.16(-0.62,0.30) | 0.76(-0.94,2.47) |
| Praziquantel alone or in combination with STH deworming with iron or micronutrients | Praziquantel alone or in combination with STH deworming | -0.16(-0.46,0.14) | 0.68(-0.14,1.51) | 1.11(-1.23,3.45) |
| Any STH deworming with micronutrients or iron | Praziquantel alone or in combination with STH deworming | -0.18(-0.43,0.07) | 0.46(-0.23,1.15) | -0.31(-2.69,2.06) |
| Micronutrients or iron alone | Praziquantel alone or in combination with STH deworming | -0.16(-0.40,0.07) | 0.40(-0.28,1.07) | -1.01(-3.05,1.04) |
| Any STH deworming with micronutrients or iron | Praziquantel alone or in combination with STH deworming with iron or micronutrients | -0.02(-0.33,0.30) | -0.22(-1.05,0.61) | -1.42(-4.15,1.30) |
|  |  |  |  |  |
| Micronutrients or iron alone | Praziquantel alone or in combination with STH deworming with iron or micronutrients | -0.01(-0.30,0.29) | -0.29(-1.09,0.52) | -2.12(-4.47,0.24) |
| Micronutrients or iron alone | Any STH deworming with micronutrients or iron | 0.01(-0.16,0.19) | -0.07(-0.43,0.30) | -0.69(-2.64,1.25) |

*bolded numbers indiciate statistically significant effects

**This table provided all 15 comparisons in the network

**Analyses with studies at low risk of bias – 6 studies, 6 nodes (adjusted, with multiple imputation)**

| Adjusted analyses | | MD (95% CI) | | |
| --- | --- | --- | --- | --- |
| treatment | comparison | weight | height | haemoglobin |
| STH deworming with any drug | Placebo or control | 0.01(-0.10,0.12) | 0.11(-0.11,0.33) | 0.07(-1.05,1.19) |
| Any STH deworming combination with praziquantel | Placebo or control | 0.17(-0.28,0.62) | -1.17(-1.99,-0.36) | 2.12(-1.68,5.91) |
| Any STH deworming combination with praziquantel with iron or micronutrients | Placebo or control | 0.34(-0.10,0.78) | -0.09(-0.92,0.75) | 2.52(-1.18,6.21) |
| Any STH deworming with micronutrients or iron | Placebo or control | 0.00(-0.21,0.21) | 0.03(-0.37,0.44) | 2.48(0.57,4.39) |
| Micronutrients or iron alone | Placebo or control | 0.00(-0.26,0.26) | -0.06(-0.55,0.43) | 1.18(-1.07,3.43) |
| Any STH deworming combination with praziquantel | STH deworming with any drug | 0.16(-0.30,0.62) | -1.28(-2.10,-0.46) | 2.05(-1.98,6.08) |
| Any STH deworming combination with praziquantel with iron or micronutrients | STH deworming with any drug | 0.33(-0.12,0.79) | -0.19(-1.05,0.66) | 2.45(-1.45,6.35) |
| Any STH deworming with micronutrients or iron | STH deworming with any drug | -0.01(-0.23,0.22) | -0.08(-0.52,0.36) | 2.41(0.32,4.50) |
| Micronutrients or iron alone | STH deworming with any drug | -0.01(-0.28,0.26) | -0.16(-0.67,0.34) | 1.11(-1.32,3.55) |
| Any STH deworming combination with praziquantel with iron or micronutrients | Any STH deworming combination with praziquantel | 0.17(-0.32,0.66) | 1.09(0.16,2.02) | 0.40(-3.71,4.51) |
| Any STH deworming with micronutrients or iron | Any STH deworming combination with praziquantel | -0.17(-0.66,0.32) | 1.21(0.32,2.09) | 0.36(-3.81,4.54) |
| Micronutrients or iron alone | Any STH deworming combination with praziquantel | -0.17(-0.62,0.27) | 1.12(0.30,1.93) | -0.94(-4.66,2.79) |
| Any STH deworming with micronutrients or iron | Any STH deworming combination with praziquantel with iron or micronutrients | -0.34(-0.82,0.13) | 0.12(-0.79,1.02) | -0.03(-4.15,4.09) |
| Micronutrients or iron alone | Any STH deworming combination with praziquantel with iron or micronutrients | -0.34(-0.78,0.09) | 0.03(-0.78,0.84) | -1.33(-5.03,2.36) |
| Micronutrients or iron alone | Any STH deworming with micronutrients or iron | 0.00(-0.30,0.29) | -0.09(-0.66,0.48) | -1.30(-3.94,1.34) |

*bolded numbers indiciate statistically significant effects

**This table provided all 15 comparisons in the network

Additional tables 13: Full network – 14 studies, 18 nodes

| Adjusted analyses | | MD (95% CI) | | |
| --- | --- | --- | --- | --- |
| Treatment | Comparison | weight | height | haemoglobin |
| Albendazole | Placebo | 0.01(-0.09,0.11) | 0.09(-0.09,0.28) | 0.16(-0.86,1.17) |
| Praziquantel |  | 0.18(-0.19,0.56) | 0.12(-0.47,0.71) | 2.44(-0.92,5.80) |
|  |  |  |  |  |
| Albendazole + praziquantel |  | 0.02(-0.16,0.19) | 0.08(-0.21,0.36) | 2.03(0.56,3.50) |
| Albendazole (high) + praziquantel + iron fortified (food/beverage) |  | 0.36(-0.10,0.82) | -0.13(-0.90,0.64) | 2.54(-1.26,6.33) |
| Albendazole (high) + Praziquantel |  | 0.19(-0.28,0.65) | -1.22(-1.97,-0.47) | 2.24(-1.65,6.12) |
| Iron fortified (food/beverage) |  | -0.04(-0.35,0.27) | -0.19(-0.71,0.32) | 0.99(-1.67,3.65) |
| Iron supplement (tablet/liquid) |  | -0.32(-0.69,0.06) | -0.16(-0.84,0.53) | 1.35(-2.06,4.76) |
| Mebendazole (high) |  | -0.02(-0.29,0.25) | 0.16(-0.34,0.65) | 0.52(-1.93,2.96) |
| Micronutrient tablet |  | 0.03(-0.26,0.32) | -0.08(-0.55,0.39) | 2.74(0.54,4.94) |
| Alben + pzq + micronutrient tablet |  | -0.21(-0.53,0.10) | -0.12(-0.55,0.32) | 3.23(0.92,5.54) |
| Alben + micronutrient fortified (food/beverage) |  | -0.01(-0.22,0.19) | 0.01(-0.32,0.35) | 0.85(-0.95,2.64) |
| Iron fortified (food/beverage) + Mebendazole (high) |  | -0.01(-0.41,0.39) | 0.11(-0.54,0.75) | 1.44(-1.93,4.82) |
| Mebendazole + iron tablet |  | -0.02(-0.27,0.22) | -0.05(-0.62,0.53) | 2.88(0.29,5.48) |
| Micronutrient fortified (food/beverage) |  | 0.04(-0.17,0.24) | -0.06(-0.39,0.28) | 0.33(-1.48,2.13) |
| Mebendazole (regular) |  | 0.25(-0.37,0.86) | 0.13(-1.47,1.74) | -0.10(-6.61,6.41) |
| Mebendazole (high) + Iron supplement (tablet/liquid) |  | -0.07(-0.37,0.22) | -0.17(-0.68,0.34) | 2.96(0.40,5.52) |
| Albendazole (reg) + praziquantel + iron |  | 0.23(-0.29,0.75) | -0.03(-0.79,0.73) | 3.24(-0.62,7.11) |
| Praziquantel | Albendazole | 0.17(-0.21,0.55) | 0.03(-0.57,0.62) | 2.28(-1.20,5.77) |
| Albendazole + praziquantel |  | 0.00(-0.20,0.20) | -0.02(-0.34,0.31) | 1.88(0.16,3.59) |
| Albendazole (high) + praziquantel + iron fortified (food/beverage) |  | 0.35(-0.12,0.82) | -0.22(-1.01,0.56) | 2.38(-1.61,6.37) |
| Albendazole (high) + Praziquantel |  | 0.17(-0.31,0.65) | -1.31(-2.06,-0.56) | 2.08(-2.02,6.19) |
| Iron fortified (food/beverage) |  | -0.06(-0.38,0.27) | -0.28(-0.82,0.25) | 0.83(-2.03,3.69) |
| Iron supplement (tablet/liquid) |  | -0.33(-0.72,0.07) | -0.25(-0.94,0.44) | 1.19(-2.31,4.69) |
| Mebendazole (high) |  | -0.03(-0.32,0.26) | 0.07(-0.44,0.57) | 0.36(-2.19,2.91) |
| Micronutrient tablet |  | 0.02(-0.30,0.33) | -0.17(-0.66,0.31) | 2.58(0.15,5.02) |
| Alben + pzq + micronutrient tablet |  | -0.22(-0.57,0.12) | -0.21(-0.66,0.24) | 3.08(0.52,5.64) |
| Alben + micronutrient fortified (food/beverage) |  | -0.03(-0.23,0.18) | -0.08(-0.41,0.26) | 0.69(-1.09,2.48) |
| Iron fortified (food/beverage) + Mebendazole (high) |  | -0.03(-0.44,0.38) | 0.02(-0.65,0.68) | 1.29(-2.28,4.85) |
| Mebendazole + iron tablet |  | -0.04(-0.30,0.23) | -0.14(-0.74,0.46) | 2.73(-0.06,5.51) |
| Micronutrient fortified (food/beverage) |  | 0.02(-0.18,0.23) | -0.15(-0.48,0.19) | 0.17(-1.62,1.96) |
| Mebendazole (regular) |  | 0.23(-0.39,0.86) | 0.04(-1.57,1.66) | -0.25(-6.80,6.29) |
| Mebendazole (high) + Iron supplement (tablet/liquid) |  | -0.09(-0.40,0.22) | -0.26(-0.79,0.26) | 2.80(0.10,5.51) |
| Albendazole (reg) + praziquantel + iron |  | 0.22(-0.32,0.76) | -0.12(-0.89,0.65) | 3.09(-0.86,7.04) |
| Albendazole + praziquantel | Praziquantel | -0.17(-0.55,0.22) | -0.05(-0.66,0.57) | -0.41(-3.76,2.95) |
| Albendazole (high) + praziquantel + iron fortified (food/beverage) |  | 0.18(-0.42,0.77) | -0.25(-1.21,0.71) | 0.10(-4.82,5.01) |
| Albendazole (high) + Praziquantel |  | 0.00(-0.61,0.61) | -1.34(-2.27,-0.40) | -0.20(-5.17,4.76) |
| Iron fortified (food/beverage) |  | -0.23(-0.71,0.26) | -0.31(-1.09,0.46) | -1.45(-5.57,2.67) |
| Iron supplement (tablet/liquid) |  | -0.50(-1.03,0.03) | -0.28(-1.17,0.62) | -1.09(-5.70,3.52) |
| Mebendazole (high) |  | -0.20(-0.67,0.26) | 0.04(-0.72,0.80) | -1.92(-6.16,2.31) |
| Micronutrient tablet |  | -0.15(-0.60,0.30) | -0.20(-0.93,0.52) | 0.30(-3.54,4.14) |
| Alben + pzq + micronutrient tablet |  | -0.39(-0.86,0.07) | -0.24(-0.95,0.47) | 0.79(-3.41,4.99) |
| Alben + micronutrient fortified (food/beverage) |  | -0.20(-0.62,0.23) | -0.11(-0.77,0.56) | -1.59(-5.45,2.27) |
| Iron fortified (food/beverage) + Mebendazole (high) |  | -0.20(-0.75,0.35) | -0.01(-0.88,0.86) | -0.99(-5.78,3.79) |
| Mebendazole + iron tablet |  | -0.21(-0.66,0.24) | -0.17(-0.99,0.66) | 0.45(-3.80,4.69) |
| Micronutrient fortified (food/beverage) |  | -0.15(-0.57,0.28) | -0.18(-0.84,0.49) | -2.11(-5.87,1.64) |
| Mebendazole (regular) |  | 0.06(-0.65,0.78) | 0.01(-1.70,1.73) | -2.54(-9.91,4.84) |
| Mebendazole (high) + Iron supplement (tablet/liquid) |  | -0.26(-0.73,0.22) | -0.29(-1.06,0.48) | 0.52(-3.65,4.69) |
| Albendazole (reg) + praziquantel + iron |  | 0.05(-0.58,0.68) | -0.15(-1.10,0.80) | 0.81(-4.29,5.91) |
| Albendazole (high) + praziquantel + iron fortified (food/beverage) | Albendazole + praziquantel | 0.34(-0.14,0.83) | -0.20(-1.02,0.61) | 0.50(-3.54,4.55) |
| Albendazole (high) + Praziquantel |  | 0.17(-0.33,0.67) | -1.29(-2.08,-0.50) | 0.21(-3.92,4.33) |
| Iron fortified (food/beverage) |  | -0.06(-0.41,0.29) | -0.27(-0.86,0.32) | -1.04(-4.04,1.95) |
| Iron supplement (tablet/liquid) |  | -0.33(-0.74,0.08) | -0.23(-0.97,0.50) | -0.68(-4.34,2.97) |
| Mebendazole (high) |  | -0.04(-0.36,0.29) | 0.08(-0.47,0.64) | -1.52(-4.37,1.34) |
| Micronutrient tablet |  | 0.01(-0.26,0.29) | -0.16(-0.59,0.27) | 0.71(-1.47,2.89) |
| Alben + pzq + micronutrient tablet |  | -0.23(-0.52,0.07) | -0.19(-0.61,0.22) | 1.20(-1.21,3.61) |
| Alben + micronutrient fortified (food/beverage) |  | -0.03(-0.30,0.24) | -0.06(-0.49,0.37) | -1.18(-3.50,1.13) |
| Iron fortified (food/beverage) + Mebendazole (high) |  | -0.03(-0.47,0.41) | 0.03(-0.66,0.73) | -0.59(-4.29,3.11) |
| Mebendazole + iron tablet |  | -0.04(-0.34,0.26) | -0.12(-0.76,0.51) | 0.85(-2.13,3.83) |
| Micronutrient fortified (food/beverage) |  | 0.02(-0.25,0.29) | -0.13(-0.56,0.30) | -1.71(-3.99,0.58) |
| Mebendazole (regular) |  | 0.23(-0.41,0.87) | 0.06(-1.57,1.69) | -2.13(-8.80,4.55) |
| Mebendazole (high) + Iron supplement (tablet/liquid) |  | -0.09(-0.44,0.26) | -0.24(-0.81,0.32) | 0.93(-2.00,3.86) |
| Albendazole (reg) + praziquantel + iron |  | 0.21(-0.29,0.72) | -0.11(-0.85,0.64) | 1.21(-2.67,5.09) |
| Albendazole (high) + Praziquantel | Albendazole (high) + praziquantel + iron fortified (food/beverage) | -0.18(-0.68,0.32) | -1.09(-1.92,-0.26) | -0.30(-4.45,3.85) |
| Iron fortified (food/beverage) |  | -0.40(-0.86,0.05) | -0.06(-0.79,0.66) | -1.55(-5.32,2.23) |
| Iron supplement (tablet/liquid) |  | -0.68(-1.25,-0.10) | -0.03(-1.09,1.03) | -1.19(-6.18,3.81) |
| Mebendazole (high) |  | -0.38(-0.89,0.13) | 0.29(-0.59,1.16) | -2.02(-6.42,2.38) |
| Micronutrient tablet |  | -0.33(-0.86,0.20) | 0.05(-0.85,0.94) | 0.20(-4.16,4.57) |
| Alben + pzq + micronutrient tablet |  | -0.57(-1.11,-0.04) | 0.01(-0.87,0.90) | 0.70(-3.79,5.18) |
| Alben + micronutrient fortified (food/beverage) |  | -0.37(-0.88,0.13) | 0.14(-0.69,0.98) | -1.69(-5.96,2.58) |
| Iron fortified (food/beverage) + Mebendazole (high) |  | -0.38(-0.95,0.20) | 0.24(-0.71,1.19) | -1.09(-5.90,3.72) |
| Mebendazole + iron tablet |  | -0.39(-0.90,0.13) | 0.08(-0.88,1.05) | 0.35(-4.31,5.01) |
| Micronutrient fortified (food/beverage) |  | -0.32(-0.83,0.18) | 0.07(-0.76,0.91) | -2.21(-6.46,2.04) |
| Mebendazole (regular) |  | -0.12(-0.87,0.64) | 0.26(-1.51,2.03) | -2.63(-10.16,4.90) |
| Mebendazole (high) + Iron supplement (tablet/liquid) |  | -0.43(-0.96,0.09) | -0.04(-0.92,0.84) | 0.42(-3.96,4.81) |
| Albendazole (reg) + praziquantel + iron |  | -0.13(-0.79,0.52) | 0.10(-1.02,1.22) | 0.71(-4.76,6.18) |
| Iron fortified (food/beverage) | Albendazole (high) + Praziquantel | -0.23(-0.69,0.23) | 1.02(0.29,1.76) | -1.25(-5.06,2.56) |
| Iron supplement (tablet/liquid) |  | -0.50(-1.09,0.09) | 1.06(0.09,2.03) | -0.89(-5.85,4.07) |
| Mebendazole (high) |  | -0.21(-0.73,0.31) | 1.38(0.55,2.20) | -1.72(-6.24,2.79) |
| Micronutrient tablet |  | -0.16(-0.71,0.40) | 1.14(0.29,1.98) | 0.50(-3.94,4.94) |
| Alben + pzq + micronutrient tablet |  | -0.40(-0.97,0.18) | 1.10(0.25,1.94) | 1.00(-3.52,5.51) |
| Alben + micronutrient fortified (food/beverage) |  | -0.20(-0.71,0.31) | 1.23(0.42,2.04) | -1.39(-5.73,2.95) |
| Iron fortified (food/beverage) + Mebendazole (high) |  | -0.20(-0.78,0.38) | 1.33(0.40,2.25) | -0.79(-5.66,4.08) |
| Mebendazole + iron tablet |  | -0.21(-0.74,0.32) | 1.17(0.23,2.11) | 0.65(-4.04,5.33) |
| Micronutrient fortified (food/beverage) |  | -0.15(-0.66,0.36) | 1.16(0.35,1.97) | -1.91(-6.21,2.39) |
| Mebendazole (regular) |  | 0.06(-0.71,0.83) | 1.35(-0.40,3.11) | -2.33(-9.92,5.25) |
| Mebendazole (high) + Iron supplement (tablet/liquid) |  | -0.26(-0.79,0.27) | 1.05(0.21,1.89) | 0.72(-3.77,5.21) |
| Albendazole (reg) + praziquantel + iron |  | 0.04(-0.65,0.74) | 1.19(0.13,2.24) | 1.01(-4.56,6.58) |
| Iron supplement (tablet/liquid) | Iron fortified (food/beverage) | -0.27(-0.73,0.18) | 0.04(-0.78,0.85) | 0.36(-3.55,4.27) |
| Mebendazole (high) |  | 0.02(-0.33,0.38) | 0.35(-0.25,0.96) | -0.47(-3.64,2.70) |
| Micronutrient tablet |  | 0.07(-0.35,0.49) | 0.11(-0.58,0.81) | 1.75(-1.68,5.18) |
| Alben + pzq + micronutrient tablet |  | -0.17(-0.60,0.27) | 0.07(-0.60,0.74) | 2.24(-1.33,5.82) |
| Alben + micronutrient fortified (food/beverage) |  | 0.03(-0.34,0.40) | 0.21(-0.40,0.82) | -0.14(-3.37,3.09) |
| Iron fortified (food/beverage) + Mebendazole (high) |  | 0.03(-0.40,0.46) | 0.30(-0.39,1.00) | 0.46(-3.20,4.11) |
| Mebendazole + iron tablet |  | 0.02(-0.38,0.41) | 0.15(-0.63,0.92) | 1.90(-1.82,5.61) |
| Micronutrient fortified (food/beverage) |  | 0.08(-0.29,0.45) | 0.14(-0.47,0.75) | -0.66(-3.85,2.52) |
| Mebendazole (regular) |  | 0.29(-0.38,0.96) | 0.33(-1.34,1.99) | -1.09(-8.02,5.85) |
| Mebendazole (high) + Iron supplement (tablet/liquid) |  | -0.03(-0.40,0.34) | 0.02(-0.59,0.63) | 1.97(-1.18,5.13) |
| Albendazole (reg) + praziquantel + iron |  | 0.27(-0.32,0.87) | 0.16(-0.78,1.10) | 2.26(-2.48,6.99) |
| Mebendazole (high) | Iron supplement (tablet/liquid) | 0.30(-0.09,0.68) | 0.32(-0.37,1.00) | -0.83(-4.37,2.70) |
| Micronutrient tablet |  | 0.35(-0.11,0.80) | 0.08(-0.74,0.89) | 1.39(-2.65,5.44) |
| Alben + pzq + micronutrient tablet |  | 0.10(-0.36,0.57) | 0.04(-0.75,0.83) | 1.88(-2.31,6.07) |
| Alben + micronutrient fortified (food/beverage) |  | 0.30(-0.13,0.73) | 0.17(-0.58,0.92) | -0.50(-4.32,3.31) |
| Iron fortified (food/beverage) + Mebendazole (high) |  | 0.30(-0.21,0.81) | 0.27(-0.60,1.13) | 0.09(-4.39,4.58) |
| Mebendazole + iron tablet |  | 0.29(-0.16,0.74) | 0.11(-0.78,1.00) | 1.54(-2.68,5.75) |
| Micronutrient fortified (food/beverage) |  | 0.35(-0.08,0.78) | 0.10(-0.65,0.85) | -1.02(-4.76,2.71) |
| Mebendazole (regular) |  | 0.56(-0.13,1.25) | 0.29(-1.42,2.01) | -1.45(-8.61,5.71) |
| Mebendazole (high) + Iron supplement (tablet/liquid) |  | 0.24(-0.15,0.64) | -0.01(-0.69,0.67) | 1.61(-1.85,5.07) |
| Albendazole (reg) + praziquantel + iron |  | 0.55(-0.08,1.17) | 0.13(-0.86,1.11) | 1.90(-3.29,7.08) |
| Micronutrient tablet | Mebendazole (high) | 0.05(-0.34,0.44) | -0.24(-0.89,0.41) | 2.22(-1.06,5.51) |
| Alben + pzq + micronutrient tablet |  | -0.19(-0.60,0.22) | -0.28(-0.90,0.35) | 2.72(-0.67,6.11) |
| Alben + micronutrient fortified (food/beverage) |  | 0.01(-0.33,0.35) | -0.14(-0.73,0.44) | 0.33(-2.63,3.29) |
| Iron fortified (food/beverage) + Mebendazole (high) |  | 0.01(-0.41,0.42) | -0.05(-0.73,0.63) | 0.93(-2.62,4.47) |
| Mebendazole + iron tablet |  | 0.00(-0.37,0.36) | -0.20(-0.95,0.54) | 2.37(-1.12,5.86) |
| Micronutrient fortified (food/beverage) |  | 0.06(-0.29,0.40) | -0.21(-0.80,0.37) | -0.19(-3.17,2.79) |
| Mebendazole (regular) |  | 0.27(-0.35,0.88) | -0.02(-1.63,1.59) | -0.61(-7.10,5.88) |
| Mebendazole (high) + Iron supplement (tablet/liquid) |  | -0.05(-0.36,0.26) | -0.33(-0.85,0.19) | 2.44(-0.23,5.12) |
| Albendazole (reg) + praziquantel + iron |  | 0.25(-0.34,0.84) | -0.19(-1.07,0.69) | 2.73(-1.77,7.23) |
| Alben + pzq + micronutrient tablet | Micronutrient tablet | -0.24(-0.52,0.04) | -0.04(-0.51,0.43) | 0.49(-2.09,3.07) |
| Alben + micronutrient fortified (food/beverage) |  | -0.04(-0.40,0.31) | 0.10(-0.47,0.66) | -1.89(-4.73,0.95) |
| Iron fortified (food/beverage) + Mebendazole (high) |  | -0.04(-0.54,0.46) | 0.19(-0.58,0.97) | -1.30(-5.31,2.72) |
| Mebendazole + iron tablet |  | -0.05(-0.43,0.32) | 0.04(-0.69,0.76) | 0.14(-3.19,3.47) |
| Micronutrient fortified (food/beverage) |  | 0.01(-0.35,0.37) | 0.03(-0.54,0.59) | -2.41(-5.24,0.41) |
| Mebendazole (regular) |  | 0.22(-0.45,0.89) | 0.22(-1.45,1.88) | -2.84(-9.71,4.04) |
| Mebendazole (high) + Iron supplement (tablet/liquid) |  | -0.10(-0.52,0.32) | -0.09(-0.74,0.56) | 0.22(-3.12,3.56) |
| Albendazole (reg) + praziquantel + iron |  | 0.20(-0.34,0.74) | 0.05(-0.78,0.88) | 0.50(-3.80,4.80) |
| Alben + micronutrient fortified (food/beverage) | Alben + pzq + micronutrient tablet | 0.20(-0.18,0.57) | 0.13(-0.41,0.67) | -2.38(-5.29,0.52) |
| Iron fortified (food/beverage) + Mebendazole (high) |  | 0.20(-0.32,0.71) | 0.23(-0.54,1.00) | -1.79(-5.84,2.26) |
| Mebendazole + iron tablet |  | 0.19(-0.21,0.58) | 0.07(-0.63,0.78) | -0.35(-3.86,3.16) |
| Micronutrient fortified (food/beverage) |  | 0.25(-0.14,0.63) | 0.06(-0.47,0.60) | -2.91(-5.87,0.06) |
| Mebendazole (regular) |  | 0.46(-0.22,1.13) | 0.25(-1.41,1.91) | -3.33(-10.23,3.58) |
| Mebendazole (high) + Iron supplement (tablet/liquid) |  | 0.14(-0.31,0.58) | -0.05(-0.69,0.59) | -0.27(-3.81,3.26) |
| Albendazole (reg) + praziquantel + iron |  | 0.44(-0.09,0.97) | 0.09(-0.73,0.91) | 0.01(-4.40,4.43) |
| Iron fortified (food/beverage) + Mebendazole (high) | Alben + micronutrient fortified (food/beverage) | 0.00(-0.45,0.45) | 0.10(-0.63,0.82) | 0.60(-3.24,4.43) |
| Mebendazole + iron tablet |  | -0.01(-0.33,0.31) | -0.06(-0.72,0.60) | 2.04(-1.04,5.11) |
| Micronutrient fortified (food/beverage) |  | 0.05(-0.18,0.28) | -0.07(-0.44,0.30) | -0.52(-2.49,1.45) |
| Mebendazole (regular) |  | 0.26(-0.39,0.91) | 0.12(-1.52,1.76) | -0.95(-7.66,5.77) |
| Mebendazole (high) + Iron supplement (tablet/liquid) |  | -0.06(-0.42,0.30) | -0.18(-0.78,0.42) | 2.11(-0.99,5.21) |
| Albendazole (reg) + praziquantel + iron |  | 0.24(-0.32,0.81) | -0.04(-0.87,0.78) | 2.40(-1.82,6.61) |
| Mebendazole + iron tablet | Iron fortified (food/beverage) + Mebendazole (high) | -0.01(-0.48,0.46) | -0.16(-1.02,0.70) | 1.44(-2.79,5.67) |
| Micronutrient fortified (food/beverage) |  | 0.05(-0.40,0.50) | -0.16(-0.89,0.56) | -1.12(-4.97,2.73) |
| Mebendazole (regular) |  | 0.26(-0.45,0.97) | 0.02(-1.68,1.73) | -1.54(-8.71,5.63) |
| Mebendazole (high) + Iron supplement (tablet/liquid) |  | -0.06(-0.48,0.36) | -0.28(-0.95,0.39) | 1.52(-2.05,5.09) |
| Albendazole (reg) + praziquantel + iron |  | 0.24(-0.42,0.91) | -0.14(-1.13,0.85) | 1.80(-3.33,6.93) |
| Micronutrient fortified (food/beverage) | Mebendazole + iron tablet | 0.06(-0.26,0.38) | -0.01(-0.67,0.65) | -2.56(-5.62,0.51) |
| Mebendazole (regular) |  | 0.27(-0.39,0.93) | 0.18(-1.52,1.88) | -2.98(-9.95,3.99) |
| Mebendazole (high) + Iron supplement (tablet/liquid) |  | -0.05(-0.44,0.34) | -0.12(-0.88,0.63) | 0.08(-3.51,3.66) |
| Albendazole (reg) + praziquantel + iron |  | 0.25(-0.32,0.83) | 0.02(-0.92,0.95) | 0.36(-4.21,4.93) |
| Mebendazole (regular) | Micronutrient fortified (food/beverage) | 0.21(-0.44,0.86) | 0.19(-1.45,1.83) | -0.42(-7.15,6.30) |
| Mebendazole (high) + Iron supplement (tablet/liquid) |  | -0.11(-0.47,0.25) | -0.11(-0.71,0.49) | 2.63(-0.46,5.73) |
| Albendazole (reg) + praziquantel + iron |  | 0.19(-0.37,0.76) | 0.02(-0.80,0.85) | 2.92(-1.31,7.15) |
| Mebendazole (high) + Iron supplement (tablet/liquid) | Mebendazole (regular) | -0.32(-0.98,0.34) | -0.30(-1.96,1.35) | 3.06(-3.74,9.85) |
| Albendazole (reg) + praziquantel + iron |  | -0.02(-0.80,0.77) | -0.16(-1.93,1.61) | 3.34(-4.19,10.87) |
| Albendazole (reg) + praziquantel + iron | Mebendazole (high) + Iron supplement (tablet/liquid) | 0.30(-0.31,0.92) | 0.14(-0.75,1.03) | 0.28(-4.30,4.87) |

**Adjusted main analyses – 13 studies (sensitivity excluding Nga 2009), 6 nodes**

| Adjusted analyses | | weight | height | haemoglobin |
| --- | --- | --- | --- | --- |
| treatment | comparison | MD (95% CI) | MD (95% CI) | MD (95% CI) |
| STH deworming with any drug | Placebo or control | 0.01(-0.08,0.11) | 0.11(-0.08,0.30) | 0.33(-0.68,1.33) |
| Any STH deworming combination with praziquantel | Placebo or control | 0.04(-0.11,0.19) | -0.07(-0.32,0.19) | 1.84(0.48,3.20) |
| Any STH deworming combination with praziquantel with iron or micronutrients | Placebo or control | -0.03(-0.28,0.22) | -0.07(-0.40,0.26) | 2.70(1.00,4.41) |
| Any STH deworming with micronutrients or iron | Placebo or control | -0.03(-0.18,0.12) | -0.06(-0.33,0.21) | 1.95(0.60,3.31) |
| Micronutrients or iron alone | Placebo or control | -0.03(-0.18,0.13) | -0.10(-0.35,0.15) | 1.23(-0.12,2.58) |
| Any STH deworming combination with praziquantel | STH deworming with any drug | 0.03(-0.15,0.20) | -0.18(-0.46,0.10) | 1.52(-0.11,3.14) |
| Any STH deworming combination with praziquantel with iron or micronutrients | STH deworming with any drug | -0.05(-0.31,0.22) | -0.18(-0.53,0.17) | 2.38(0.43,4.33) |
| Any STH deworming with micronutrients or iron | STH deworming with any drug | -0.04(-0.20,0.11) | -0.17(-0.45,0.11) | 1.63(0.15,3.10) |
| Micronutrients or iron alone | STH deworming with any drug | -0.04(-0.21,0.13) | -0.21(-0.47,0.06) | 0.90(-0.61,2.42) |
| Any STH deworming combination with praziquantel with iron or micronutrients | Any STH deworming combination with praziquantel | -0.07(-0.32,0.17) | 0.00(-0.34,0.33) | 0.86(-1.02,2.74) |
| Any STH deworming with micronutrients or iron | Any STH deworming combination with praziquantel | -0.07(-0.27,0.13) | 0.01(-0.33,0.35) | 0.11(-1.75,1.97) |
| Micronutrients or iron alone | Any STH deworming combination with praziquantel | -0.07(-0.25,0.12) | -0.03(-0.32,0.27) | -0.61(-2.18,0.95) |
| Any STH deworming with micronutrients or iron | Any STH deworming combination with praziquantel with iron or micronutrients | 0.00(-0.28,0.28) | 0.01(-0.39,0.41) | -0.75(-2.85,1.35) |
| Micronutrients or iron alone | Any STH deworming combination with praziquantel with iron or micronutrients | 0.01(-0.22,0.24) | -0.02(-0.37,0.32) | -1.47(-3.41,0.46) |
| Micronutrients or iron alone | Any STH deworming with micronutrients or iron | 0.00(-0.18,0.19) | -0.03(-0.33,0.26) | -0.72(-2.31,0.87) |

**Sensitivity analysis for 6 studies with >20% relative reduction in *A Lumbricoides*  prevalence**

| treatment | comparison | weight | height | haemoglobin |
| --- | --- | --- | --- | --- |
| STH deworming with any drug | Placebo or control | 0.08(-0.10,0.26) | 0.06(-0.29,0.40) | 0.72(-1.06,2.50) |
| Any STH deworming combination with praziquantel | Placebo or control | -0.05(-0.26,0.16) | 0.00(-0.29,0.28) | 1.72(-0.01,3.44) |
| Any STH deworming combination with praziquantel with iron or micronutrients | Placebo or control | -0.26(-0.56,0.04) | -0.14(-0.52,0.23) | 2.84(0.58,5.09) |
| Any STH deworming with micronutrients or iron | Placebo or control | 0.04(-0.17,0.25) | -0.01(-0.34,0.32) | 2.74(0.95,4.52) |
| Micronutrients or iron alone | Placebo or control | -0.04(-0.23,0.14) | -0.11(-0.40,0.17) | 1.98(0.48,3.48) |
| Any STH deworming combination with praziquantel | STH deworming with any drug | -0.13(-0.39,0.14) | -0.06(-0.46,0.34) | 1.00(-1.35,3.34) |
| Any STH deworming combination with praziquantel with iron or micronutrients | STH deworming with any drug | -0.34(-0.67,-0.01) | -0.20(-0.65,0.25) | 2.12(-0.62,4.85) |
| Any STH deworming with micronutrients or iron | STH deworming with any drug | -0.04(-0.26,0.18) | -0.06(-0.40,0.28) | 2.02(0.13,3.91) |
| Micronutrients or iron alone | STH deworming with any drug | -0.12(-0.33,0.09) | -0.17(-0.50,0.16) | 1.26(-0.61,3.14) |
| Any STH deworming combination with praziquantel with iron or micronutrients | Any STH deworming combination with praziquantel | -0.22(-0.51,0.08) | -0.14(-0.51,0.23) | 1.12(-1.30,3.54) |
| Any STH deworming with micronutrients or iron | Any STH deworming combination with praziquantel | 0.09(-0.20,0.37) | 0.00(-0.40,0.39) | 1.02(-1.33,3.38) |
| Micronutrients or iron alone | Any STH deworming combination with praziquantel | 0.01(-0.23,0.24) | -0.11(-0.43,0.21) | 0.26(-1.63,2.16) |
| Any STH deworming with micronutrients or iron | Any STH deworming combination with praziquantel with iron or micronutrients | 0.30(-0.04,0.65) | 0.14(-0.32,0.59) | -0.10(-2.88,2.68) |
| Micronutrients or iron alone | Any STH deworming combination with praziquantel with iron or micronutrients | 0.22(-0.05,0.50) | 0.03(-0.34,0.40) | -0.86(-3.23,1.52) |
| Micronutrients or iron alone | Any STH deworming with micronutrients or iron | -0.08(-0.30,0.14) | -0.11(-0.42,0.20) | -0.76(-2.58,1.07) |

*bolded numbers indiciate statistically significant effects

**This table provided all 15 comparisons in the network

Additional tables 14: Distribution of effect modifiers for each comparison of collapsed network

Table a. Placebo vs. STH

| Variable | | Placebo | | | STH | | |
| --- | --- | --- | --- | --- | --- | --- | --- |
|  |  | (n) | (%) | C.I (95%) | (n) | (%) | C.I (95%) |
| BMI for Age |  |  |  |  |  |  |  |
|  | < = -2 | 520 | 13 | (12 – 14) | 797 | 15 | (14 – 16) |
|  | > - 2 | 3568 | 87 | (86 – 88) | 4358 | 85 | (84 – 86) |
| Height for Age |  |  |  |  |  |  |  |
|  | < = -2 | 1350 | 33 | (32 – 34) | 1892 | 37 | (35 – 38) |
|  | > - 2 | 2738 | 67 | (66 – 68) | 3263 | 63 | (62 – 65) |
| Hookworm |  |  |  |  |  |  |  |
|  | 0 – No | 2411 | 59 | (57 – 60) | 2394 | 46 | (45 – 48) |
|  | 1 – 384 | 876 | 21 | (20 – 23) | 1102 | 21 | (20 – 22) |
|  | > 384 | 800 | 20 | (18 – 21) | 1659 | 32 | (31 – 33) |
| Trichuris |  |  |  |  |  |  |  |
|  | 0 – No | 1933 | 47 | (46 – 39) | 1891 | 37 | (35 – 38) |
|  | 1 – 288 | 1025 | 25 | (24 – 26) | 1321 | 26 | (24 – 27) |
|  | > 288 | 1124 | 28 | (26 – 29) | 1939 | 38 | (36 – 39) |
| *A Lumbricoides* |  |  |  |  |  |  |  |
|  | 0 - No | 2128 | 52 | (51 – 54) | 2404 | 47 | (45 – 48) |
|  | 1 – 1776 | 1109 | 27 | (26 – 28) | 1441 | 28 | (27 – 29) |
|  | > 1776 | 851 | 21 | (20 – 22) | 1309 | 25 | (24 – 27) |
| Anyworm |  |  |  |  |  |  |  |
|  | 0 | 1521 | 37 | (36 - 39) | 1487 | 29 | (28 – 30) |
|  | 1 | 1697 | 42 | (40 - 43) | 2192 | 43 | (41 – 44) |
|  | 2 | 870 | 21 | (20 – 23) | 1476 | 29 | (27 - 30) |
| Anemia |  |  |  |  |  |  |  |
|  | No | 2222 | 54 | (53 – 56) | 3048 | 59 | (58 – 60) |
|  | Yes | 1866 | 46 | (44 – 47) | 2107 | 41 | (40 – 42) |
| Age |  |  |  |  |  |  |  |
|  | < = 5 | 1123 | 27 | (26 – 29) | 1113 | 22 | (20 – 23) |
|  | > 5 | 2965 | 73 | (71 – 74) | 4042 | 78 | (77 – 80) |
| Sex |  |  |  |  |  |  |  |
|  | Male | 2171 | 53 | (52 – 55) | 2643 | 51 | (50 – 53) |
|  | Female | 1917 | 47 | (45 – 48) | 2512 | 49 | (47 – 50) |
| Total (n ) |  | 4168 |  |  | 5155 |  |  |
| Number of studies | 9 | | | | | | |

Table b. Placebo vs PZQ ± STH

| Variable | | Placebo | | | PZQ ± STH | | |
| --- | --- | --- | --- | --- | --- | --- | --- |
|  |  | (n) | (%) | C.I (95%) | (n) | (%) | C.I (95%) |
| BMI for Age |  |  |  |  |  |  |  |
|  | < = -2 | 41 | 6 | (4 - 8) | 65 | 9 | (7 - 11) |
|  | > - 2 | 602 | 94 | (92 - 96) | 686 | 91 | (89 - 93) |
| Height for Age |  |  |  |  |  |  |  |
|  | < = -2 | 169 | 26 | (23 - 30) | 215 | 29 | (25 - 32) |
|  | > - 2 | 474 | 74 | (70 - 77) | 536 | 71 | (68 - 75) |
| Hookworm |  |  |  |  |  |  |  |
|  | 0 | 177 | 28 | (24 - 31) | 192 | 26 | (22 - 29) |
|  | 1 – 384 | 253 | 39 | (36 - 43) | 298 | 40 | (36 - 43) |
|  | > 384 | 213 | 33 | (29 - 37) | 261 | 35 | (31 - 38) |
| Trichuris |  |  |  |  |  |  |  |
|  | 0 | 302 | 47 | (43 - 51) | 325 | 43 | (40 - 47) |
|  | 1 – 288 | 228 | 35 | (32 - 39) | 280 | 37 | (34 - 41) |
|  | > 288 | 113 | 18 | (15 - 21) | 146 | 19 | (17 - 22) |
| *A Lumbricoides* |  |  |  |  |  |  |  |
|  | 0 | 474 | 74 | (70 - 77) | 547 | 73 | (70 - 76) |
|  | 1 – 1776 | 33 | 5 | (3 - 7) | 47 | 6 | (5 - 8) |
|  | > 1776 | 136 | 21 | (18 - 24) | 157 | 21 | (18 - 24) |
| Anyworm |  |  |  |  |  |  |  |
|  | 0 | 93 | 14 | (12 - 17) | 97 | 13 | (11 - 15) |
|  | 1 | 384 | 60 | (56 - 64) | 477 | 65 | (60 - 67) |
|  | 2 | 166 | 26 | (22 - 29) | 177 | 24 | (21 - 27) |
| Anemia |  |  |  |  |  |  |  |
|  | No | 346 | 54 | (50 - 58) | 434 | 58 | (54 - 61) |
|  | Yes | 297 | 46 | (42 - 50) | 317 | 42 | (39 - 46) |
| Age |  |  |  |  |  |  |  |
|  | < = 5 | 1 | 0.2 | (0) | 1 | 0.1 | (0) |
|  | > 5 | 642 | 99.8 | (100) | 750 | 99.9 | (100) |
| Sex |  |  |  |  |  |  |  |
|  | Male | 347 | 54 | (50 - 58) | 416 | 55 | (52 - 59) |
|  | Female | 296 | 45 | (42 - 50) | 335 | 45 | (41 - 48) |
| Total (n) |  | 643 |  |  | 751 |  |  |
| Number of studies | 5 | | | | | | |

Table c. Placebo vs PZQ ± STH + MCN/iron

| Variable | | Placebo | | | PZQ + STH + MCN/Iron | | |
| --- | --- | --- | --- | --- | --- | --- | --- |
|  |  | (n) | (%) | C.I (95%) | (n) | (%) | C.I (95%) |
| BMI for Age |  |  |  |  |  |  |  |
|  | < = -2 | 28 | 8 | (5 - 10) | 22 | 6 | (3 - 8) |
|  | > - 2 | 345 | 92 | (90 - 95) | 352 | 94 | (92 - 97) |
| Height for Age |  |  |  |  |  |  |  |
|  | < = -2 | 73 | 20 | (16 - 24) | 71 | 19 | (15 - 23) |
|  | > - 2 | 300 | 80 | (76 - 84) | 303 | 81 | (77 - 85) |
| Hookworm |  |  |  |  |  |  |  |
|  | 0 - No | 151 | 41 | (35 - 45) | 155 | 41 | (36 - 46) |
|  | 1 – 384 | 161 | 43 | (38 - 48) | 168 | 45 | (40 - 50) |
|  | > 384 | 61 | 16 | (13 - 20) | 51 | 14 | (10 - 17) |
| Trichuris |  |  |  |  |  |  |  |
|  | 0 – No | 229 | 61 | (56 - 66) | 242 | 65 | (60 - 70) |
|  | 1 – 288 | 121 | 32 | (28 - 37) | 111 | 30 | (25 - 34) |
|  | > 288 | 23 | 6 | (4 - 9) | 21 | 6 | (3 - 8) |
| *A Lumbricoides* |  |  |  |  |  |  |  |
|  | 0 - No | 326 | 87 | (84 - 91) | 325 | 87 | (83 - 90) |
|  | 1 – 1776 | 19 | 5 | (3 - 7) | 23 | 6 | (4 - 9) |
|  | > 1776 | 28 | 8 | (5- 10) | 26 | 7 | (4 - 10) |
| Anyworm |  |  |  |  |  |  |  |
|  | 0 | 87 | 23 | (19 - 28) | 95 | 25 | (21 - 30) |
|  | 1 | 248 | 67 | (62 - 71) | 248 | 66 | (61 - 71) |
|  | 2 | 38 | 10 | (7 - 13) | 31 | 8 | (5 - 11) |
| Anemia |  |  |  |  |  |  |  |
|  | No | 158 | 42 | (37 - 47) | 178 | 48 | (43 - 53) |
|  | Yes | 215 | 58 | (53 - 63) | 196 | 52 | (47 - 57) |
| Age |  |  |  |  |  |  |  |
|  | < = 5 | 0 | 0 | (0) | 0 | 0 | (0) |
|  | > 5 | 373 | 100 | (100) | 374 | 100 | (100) |
| Sex |  |  |  |  |  |  |  |
|  | Male | 192 | 51 | (46 - 57) | 199 | 53 | (48 - 58) |
|  | Female | 181 | 49 | (43 - 54) | 175 | 47 | (42 - 52) |
| Total (n) |  | 373 |  |  | 374 |  |  |
| Number of studies | 3 | | | | | | |

Table d. Placebo vs STH + MCN/Iron

| Variable | | Placebo | | | STH + MCN/Iron | | | |
| --- | --- | --- | --- | --- | --- | --- | --- | --- |
|  |  | (n) | (%) | C.I (95%) | | (n) | (%) | C.I (95%) |
| BMI for Age |  |  |  |  | |  |  |  |
|  | < = -2 | 383 | 29 | (27 - 32) | | 405 | 28 | (26 - 31) |
|  | > - 2 | 918 | 71 | (68 - 73) | | 1019 | 72 | (69 - 74) |
| Height for Age |  |  |  |  | |  |  |  |
|  | < = -2 | 424 | 33 | (30 - 35) | | 454 | 32 | (29 - 34) |
|  | > - 2 | 877 | 67 | (65 - 70) | | 970 | 68 | (66 - 71) |
| Hookworm |  |  |  |  | |  |  |  |
|  | 0 – No | 1074 | 83 | (80 - 85) | | 1186 | 83 | (81 - 85) |
|  | 1 – 384 | 176 | 14 | (12 - 15) | | 178 | 13 | (11 - 14) |
|  | > 384 | 51 | 4 | (3 - 5) | | 60 | 4 | (3 - 5) |
| Trichuris |  |  |  |  | |  |  |  |
|  | 0 – No | 929 | 71 | (69 - 74) | | 954 | 67 | (65 - 69) |
|  | 1 – 288 | 273 | 21 | (19 - 23) | | 324 | 23 | (21 - 25) |
|  | > 288 | 99 | 8 | (6 - 9) | | 146 | 10 | (9 - 12) |
| *A Lumbricoides* |  |  |  |  | |  |  |  |
|  | 0 - No | 791 | 61 | (58 - 63) | | 850 | 60 | (57 - 62) |
|  | 1 – 1776 | 225 | 17 | (15 - 19) | | 237 | 17 | (15 - 19) |
|  | > 1776 | 285 | 22 | (20 - 24) | | 337 | 24 | (21 - 26) |
| Anyworm |  |  |  |  | |  |  |  |
|  | 0 | 657 | 50 | (48 - 53) | | 688 | 48 | (46 - 51) |
|  | 1 | 436 | 34 | (31 - 36) | | 468 | 33 | (30 - 35) |
|  | 2 | 208 | 16 | (14 - 18) | | 268 | 19 | (17 - 21) |
| Anemia |  |  |  |  | |  |  |  |
|  | No | 493 | 38 | (35- 41) | | 511 | 36 | (33 - 38) |
|  | Yes | 808 | 62 | (59 - 65) | | 913 | 64 | (62 - 67) |
| Age |  |  |  |  | |  |  |  |
|  | < = 5 | 117 | 9 | (7 - 11) | | 107 | 8 | (6 - 9) |
|  | > 5 | 1184 | 91 | (89 - 93) | | 1317 | 92 | (91 - 94) |
| Sex |  |  |  |  | |  |  |  |
|  | Male | 666 | 51 | (48 - 54) | | 729 | 51 | (49 - 54) |
|  | Female | 635 | 49 | (46 - 52) | | 695 | 49 | (46 - 51) |
| Total (n) |  | 1301 |  |  | | 1424 |  |  |
| Number of studies | 5 | | | | | | | |

Table e. Placebo vs MCN/iron

| Variable | | Placebo | | | MCN/Iron | | |
| --- | --- | --- | --- | --- | --- | --- | --- |
|  |  | (n) | (%) | C.I (95%) | (n) | (%) | C.I (95%) |
| BMI for Age |  |  |  |  |  |  |  |
|  | < = -2 | 98 | 12 | (9 - 14) | 104 | 12 | (10 - 14) |
|  | > - 2 | 744 | 88 | (86 - 91) | 750 | 88 | (86 - 90) |
| Height for Age |  |  |  |  |  |  |  |
|  | < = -2 | 249 | 30 | (26 - 33) | 252 | 30 | (26 - 33) |
|  | > - 2 | 593 | 70 | (67 - 74) | 602 | 70 | (67 - 74) |
| Hookworm |  |  |  |  |  |  |  |
|  | 0 – No | 546 | 65 | (62 - 68) | 533 | 62 | (59 - 66) |
|  | 1 – 384 | 264 | 31 | (28 - 34) | 284 | 33 | (30 - 36) |
|  | > 384 | 32 | 4 | (3 - 5) | 37 | 4 | (3 - 6) |
| Trichuris |  |  |  |  |  |  |  |
|  | 0 – No | 449 | 53 | (50 - 57) | 437 | 51 | (48 - 55) |
|  | 1 – 288 | 295 | 35 | (32 - 38) | 327 | 38 | (35 - 42) |
|  | > 288 | 98 | 12 | (9 - 14) | 90 | 11 | (8 - 13) |
| *A Lumbricoides* |  |  |  |  |  |  |  |
|  | 0 - No | 493 | 59 | (55 - 62) | 512 | 60 | (57 - 63) |
|  | 1 – 1776 | 170 | 20 | (17 - 23) | 172 | 20 | (17 - 23) |
|  | > 1776 | 179 | 21 | (18 - 24) | 170 | 20 | (17 - 23) |
| Anyworm |  |  |  |  |  |  |  |
|  | 0 | 218 | 26 | (23 - 29) | 218 | 26 | (23 - 28) |
|  | 1 | 497 | 59 | (56 - 62) | 501 | 59 | (55 - 62) |
|  | 2 | 127 | 15 | (13 - 18) | 135 | 16 | (13 - 18) |
| Anemia |  |  |  |  |  |  |  |
|  | No | 424 | 50 | (47 - 54) | 436 | 51 | (48 - 54) |
|  | Yes | 418 | 50 | (46 - 53) | 418 | 49 | (46 - 52) |
| Age |  |  |  |  |  |  |  |
|  | < = 5 | 117 | 14 | (12 - 16) | 109 | 13 | (11 - 15) |
|  | > 5 | 725 | 86 | (84 - 88) | 745 | 87 | (85 - 89) |
| Sex |  |  |  |  |  |  |  |
|  | Male | 416 | 49 | (46 - 53) | 444 | 52 | (49 - 55) |
|  | Female | 426 | 51 | (47 - 54) | 410 | 48 | (45 - 51) |
| Total (n) |  | 842 |  |  | 854 |  |  |
| Number of studies | 6 | | | | | | |

Table f. Head to Head (STH vs PZQ ± STH)

| Variable | | STH | | | PZQ ± STH | | |
| --- | --- | --- | --- | --- | --- | --- | --- |
|  |  | (n) | (%) | C.I (95%) | (n) | (%) | C.I (95%) |
| BMI for Age |  |  |  |  |  |  |  |
|  | < = -2 | 10 | 11 | (4 - 17) | 24 | 13 | (8 - 18) |
|  | > - 2 | 82 | 89 | (83 - 96) | 164 | 87 | (82 - 92) |
| Height for Age |  |  |  |  |  |  |  |
|  | < = -2 | 27 | 29 | (20 - 39) | 56 | 30 | (23 - 36) |
|  | > - 2 | 65 | 71 | (61 - 80) | 132 | 70 | (64 - 77) |
| Hookworm |  |  |  |  |  |  |  |
|  | 0 – No | 12 | 13 | (6 - 20) | 28 | 15 | (10 - 20) |
|  | 1 – 384 | 46 | 50 | (40 - 60) | 85 | 45 | (38 - 52) |
|  | > 384 | 34 | 37 | (27 - 47) | 75 | 40 | (33 - 47) |
| Trichuris |  |  |  |  |  |  |  |
|  | 0 – No | 19 | 21 | (12 - 29) | 39 | 21 | (15 - 27) |
|  | 1 – 288 | 42 | 46 | (35 - 56) | 91 | 48 | (41 - 56) |
|  | > 288 | 31 | 34 | (24 - 44) | 58 | 31 | (24 - 38) |
| *A Lumbricoides* |  |  |  |  |  |  |  |
|  | 0 - No | 64 | 70 | (60 - 79) | 120 | 64 | (57 - 71) |
|  | 1 – 1776 | 13 | 14 | (7 - 21) | 23 | 12 | (8 - 17) |
|  | > 1776 | 15 | 16 | (9 - 24) | 45 | 24 | (18 - 30) |
| Anyworm |  |  |  |  |  |  |  |
|  | 0 | 5 | 5 | (1 - 10) | 12 | 6 | (3 - 10) |
|  | 1 | 64 | 70 | (60 - 79) | 114 | 61 | (54 - 68) |
|  | 2 | 23 | 25 | (16 - 34) | 62 | 33 | (26 - 40) |
| Anemia |  |  |  |  |  |  |  |
|  | No | 63 | 68 | (59 - 78) | 137 | 73 | (66 - 79) |
|  | Yes | 29 | 31.5 | (22 - 41) | 51 | 27 | (21 - 34) |
| Age |  |  |  |  |  |  |  |
|  | < = 5 | 0 | 0 | (0) | 0 | 0 | (0) |
|  | > 5 | 92 | 100 | (100) | 188 | 100 | (100) |
| Sex |  |  |  |  |  |  |  |
|  | Male | 55 | 60 | (50 - 70) | 117 | 62 | (55 - 69) |
|  | Female | 37 | 40 | (30 - 50) | 71 | 38 | (31 - 45) |
| Total (n) |  | 92 |  |  | 188 |  |  |
| Number of studies | 1 | | | | | | |

Table g. Head to Head (STH vs STH + MCN/iron)

| Variable | | STH | | | STH + MCN/Iron | | |
| --- | --- | --- | --- | --- | --- | --- | --- |
|  |  | (n) | (%) | C.I (95%) | (n) | (%) | C.I (95%) |
| BMI for Age |  |  |  |  |  |  |  |
|  | < = -2 | 86 | 16 | (13 - 19) | 86 | 14 | (11 - 17) |
|  | > - 2 | 447 | 84 | (81 - 87) | 524 | 86 | (83 - 89) |
| Height for Age |  |  |  |  |  |  |  |
|  | < = -2 | 203 | 38 | (38 - 42) | 214 | 35 | (31 - 39) |
|  | > - 2 | 330 | 62 | (58 - 66) | 396 | 65 | (61 - 69) |
| Hookworm |  |  |  |  |  |  |  |
|  | 0 – No | 411 | 77 | (74 - 81) | 473 | 78 | (74 - 81) |
|  | 1 – 384 | 113 | 21 | (18 - 25) | 122 | 20 | (17 - 23) |
|  | > 384 | 9 | 2 | (1 - 3) | 15 | 2 | (1 - 4) |
| Trichuris |  |  |  |  |  |  |  |
|  | 0 – No | 245 | 46 | (42 - 50) | 256 | 42 | (38 - 46) |
|  | 1 – 288 | 207 | 39 | (35 - 43) | 230 | 38 | (34 - 42) |
|  | > 288 | 81 | 15 | (12 - 18) | 124 | 20 | (17 - 24) |
| *A Lumbricoides* |  |  |  |  |  |  |  |
|  | 0 - No | 232 | 44 | (39 - 48) | 250 | 41 | (37 - 45) |
|  | 1 – 1776 | 145 | 27 | (23 -31) | 164 | 27 | (23 - 30) |
|  | > 1776 | 156 | 29 | (25 - 33) | 196 | 32 | (28 - 36) |
| Anyworm |  |  |  |  |  |  |  |
|  | 0 | 138 | 26 | (22 - 30) | 137 | 22 | (19 - 26) |
|  | 1 | 285 | 53 | (49 - 58) | 310 | 51 | (47 - 55) |
|  | 2 | 110 | 21 | (17 - 24) | 163 | 27 | (23 - 30) |
| Anemia |  |  |  |  |  |  |  |
|  | No | 300 | 56 | (52 - 61) | 353 | 58 | (54 - 62) |
|  | Yes | 233 | 44 | (39 - 48) | 257 | 42 | (38 - 46) |
| Age |  |  |  |  |  |  |  |
|  | < = 5 | 101 | 19 | (16 - 22) | 107 | 18 | (15 - 21) |
|  | > 5 | 432 | 81 | (78 - 84) | 503 | 82 | (79 - 85) |
| Sex |  |  |  |  |  |  |  |
|  | Male | 271 | 51 | (47 - 55) | 314 | 51 | (47 - 55) |
|  | Female | 262 | 49 | (45 - 53) | 296 | 49 | (45 - 53) |
| Total (n) |  | 533 |  |  | 610 |  |  |
| Number of studies | 4 | | | | | | |

Table h. Head to Head (STH vs MCN/Iron)

| Variable | | STH | | | MCN/Iron | | |
| --- | --- | --- | --- | --- | --- | --- | --- |
|  |  | (n) | (%) | C.I (95%) | (n) | (%) | C.I (95%) |
| BMI for Age |  |  |  |  |  |  |  |
|  | < = -2 | 86 | 16 | (13 - 19) | 74 | 14 | (11 - 17) |
|  | > - 2 | 447 | 84 | (81 - 87) | 469 | 86 | (83 - 89) |
| Height for Age |  |  |  |  |  |  |  |
|  | < = -2 | 203 | 38 | (38 - 42) | 203 | 37 | (33 - 41) |
|  | > - 2 | 330 | 62 | (58 - 66) | 340 | 63 | (59 - 67) |
| Hookworm |  |  |  |  |  |  |  |
|  | 0 – No | 411 | 77 | (74 - 81) | 409 | 75 | (72 - 79) |
|  | 1 – 384 | 113 | 21 | (18 - 25) | 126 | 23 | (20 - 27) |
|  | > 384 | 9 | 2 | (1 - 3) | 8 | 2 | (0 - 2) |
| Trichuris |  |  |  |  |  |  |  |
|  | 0 – No | 245 | 46 | (42 - 50) | 243 | 45 | (41 - 49) |
|  | 1 – 288 | 207 | 39 | (35 - 43) | 220 | 41 | (36 - 45) |
|  | > 288 | 81 | 15 | (12 - 18) | 80 | 15 | (12 - 18) |
| *A Lumbricoides* |  |  |  |  |  |  |  |
|  | 0 - No | 232 | 44 | (39 - 48) | 243 | 45 | (41 - 49) |
|  | 1 – 1776 | 145 | 27 | (23 -31) | 150 | 28 | (24 - 31) |
|  | > 1776 | 156 | 29 | (25 - 33) | 150 | 28 | (24 - 31) |
| Anyworm |  |  |  |  |  |  |  |
|  | 0 | 138 | 26 | (22 - 30) | 139 | 26 | (22 - 29) |
|  | 1 | 285 | 53 | (49 - 58) | 286 | 53 | (48 - 57) |
|  | 2 | 110 | 21 | (17 - 24) | 118 | 22 | (18 - 25) |
| Anemia |  |  |  |  |  |  |  |
|  | No | 300 | 56 | (52 - 61) | 301 | 55 | (51 - 60) |
|  | Yes | 233 | 44 | (39 - 48) | 242 | 45 | (40 - 49) |
| Age |  |  |  |  |  |  |  |
|  | < = 5 | 101 | 19 | (16 - 22) | 108 | 20 | (17 - 23) |
|  | > 5 | 432 | 81 | (78 - 84) | 435 | 80 | (77 - 83) |
| Sex |  |  |  |  |  |  |  |
|  | Male | 271 | 51 | (47 - 55) | 280 | 52 | (47 - 56) |
|  | Female | 262 | 49 | (45 - 53) | 263 | 48 | (44 - 53) |
| Total (n) |  | 533 |  |  | 543 |  |  |
| Number of studies | 4 | | | | | | |

Table i. Head to Head (PZQ ± STH vs PZQ ± STH + MCN/Iron)

| Variable | | PZQ ± STH | | | PZQ + STH + MCN/Iron | | |
| --- | --- | --- | --- | --- | --- | --- | --- |
|  |  | (n) | (%) | C.I (95%) | (n) | (%) | C.I (95%) |
| BMI for Age |  |  |  |  |  |  |  |
|  | < = -2 | 31 | 8 | (5 - 11) | 22 | 6 | (3 - 8) |
|  | > - 2 | 354 | 92 | (89 - 95) | 352 | 94 | (92 - 97) |
| Height for Age |  |  |  |  |  |  |  |
|  | < = -2 | 74 | 19 | (15 - 23) | 71 | 19 | (15 - 23) |
|  | > - 2 | 311 | 81 | (77 - 85) | 303 | 81 | (77 - 85) |
| Hookworm |  |  |  |  |  |  |  |
|  | 0 – No | 151 | 39 | (34 - 44) | 155 | 41 | (36 - 46) |
|  | 1 – 384 | 175 | 45 | (40 - 50) | 168 | 45 | (40 - 50) |
|  | > 384 | 59 | 15 | (12 - 19) | 51 | 14 | (10 - 17) |
| Trichuris |  |  |  |  |  |  |  |
|  | 0 – No | 235 | 61 | (56 - 66) | 242 | 65 | (60 - 70) |
|  | 1 – 288 | 126 | 33 | (28 - 37) | 111 | 30 | (25 - 34) |
|  | > 288 | 24 | 6 | (4 - 9) | 21 | 6 | (3 - 8) |
| *A Lumbricoides* |  |  |  |  |  |  |  |
|  | 0 - No | 340 | 88 | (85 - 92) | 325 | 87 | (83 - 90) |
|  | 1 – 1776 | 18 | 5 | (3 - 7) | 23 | 6 | (4 - 9) |
|  | > 1776 | 27 | 7 | (4 - 10) | 26 | 7 | (4 - 10) |
| Anyworm |  |  |  |  |  |  |  |
|  | 0 | 85 | 22 | (18 - 26) | 95 | 25 | (21 - 30) |
|  | 1 | 271 | 70 | (66 - 75) | 248 | 66 | (61 - 71) |
|  | 2 | 29 | 8 | (5 - 10) | 31 | 8 | (5 - 11) |
| Anemia |  |  |  |  |  |  |  |
|  | No | 174 | 45 | (40 - 50) | 178 | 48 | (43 - 53) |
|  | Yes | 211 | 55 | (50 - 60) | 196 | 52 | (47 - 57) |
| Age |  |  |  |  |  |  |  |
|  | < = 5 | 0 | 0 | (0) | 0 | 0 | (0) |
|  | > 5 | 385 | 100 | (100) | 374 | 100 | (100) |
| Sex |  |  |  |  |  |  |  |
|  | Male | 203 | 53 | (48 - 58) | 199 | 53 | (48 - 58) |
|  | Female | 182 | 47 | (42 - 52) | 175 | 47 | (42 - 52) |
| Total (n) |  | 385 |  |  | 374 |  |  |
| Number of studies | 3 | | | | | | |

Table j. Head to Head (PZQ ± STH vs MCN/Iron)

| Variable | | PZQ ± STH | | | MCN/Iron | | |
| --- | --- | --- | --- | --- | --- | --- | --- |
|  |  | (n) | (%) | C.I (95%) | (n) | (%) | C.I (95%) |
| BMI for Age |  |  |  |  |  |  |  |
|  | < = -2 | 23 | 7 | (5 - 10) | 30 | 10 | (6 - 13) |
|  | > - 2 | 284 | 93 | (90 - 95) | 281 | 90 | (87 - 94) |
| Height for Age |  |  |  |  |  |  |  |
|  | < = -2 | 52 | 17 | (13 -21) | 49 | 16 | (12 - 20) |
|  | > - 2 | 255 | 83 | (79 - 87) | 262 | 84 | (80 - 88) |
| Hookworm |  |  |  |  |  |  |  |
|  | 0 – No | 139 | 45 | (40 - 51) | 124 | 40 | (34 - 45) |
|  | 1 – 384 | 147 | 48 | (42 - 54) | 158 | 51 | (45 - 56) |
|  | > 384 | 21 | 7 | (4 - 10) | 29 | 9 | (6 - 13) |
| Trichuris |  |  |  |  |  |  |  |
|  | 0 – No | 187 | 61 | (55 - 66) | 194 | 62 | (57 - 68) |
|  | 1 – 288 | 111 | 36 | (31 - 42) | 107 | 34 | (29 - 40) |
|  | > 288 | 9 | 3 | (1 - 5) | 10 | 3 | (1 - 5) |
| *A Lumbricoides* |  |  |  |  |  |  |  |
|  | 0 – No | 270 | 88 | (84 - 92) | 269 | 87 | (83 - 90) |
|  | 1 – 1776 | 16 | 5 | (3 - 8) | 22 | 7 | (4 - 10) |
|  | > 1776 | 21 | 7 | (4 - 10) | 20 | 6 | (4 - 9) |
| Anyworm |  |  |  |  |  |  |  |
|  | 0 | 85 | 28 | (23 - 33) | 79 | 25 | (21 - 30) |
|  | 1 | 207 | 67 | (62 - 73) | 215 | 69 | (64 - 74) |
|  | 2 | 15 | 5 | (2 - 7) | 17 | 6 | (3 - 8) |
| Anemia |  |  |  |  |  |  |  |
|  | No | 125 | 41 | (35 - 46) | 135 | 43 | (38 - 49) |
|  | Yes | 182 | 59 | (54 - 65) | 176 | 57 | (51 - 62) |
| Age |  |  |  |  |  |  |  |
|  | < = 5 | 0 | 0 | (0) | 1 | 0.3 | (0) |
|  | > 5 | 307 | 100 | (100) | 310 | 99.7 | (100) |
| Sex |  |  |  |  |  |  |  |
|  | Male | 163 | 53 | (47 - 59) | 164 | 53 | (47 - 58) |
|  | Female | 144 | 47 | (41 - 53) | 147 | 47 | (42 - 53) |
| Total (n) |  | 307 |  |  | 311 |  |  |
| Number of studies | 2 | | | | | | |

Table k. Head to Head (PZQ ± STH + MCN/Iron vs MCN/Iron)

| Variable | | PZQ + STH + MCN/Iron | | | MCN/Iron | | |
| --- | --- | --- | --- | --- | --- | --- | --- |
|  |  | (n) | (%) | C.I (95%) | (n) | (%) | C.I (95%) |
| BMI for Age |  |  |  |  |  |  |  |
|  | < = -2 | 19 | 6 | (3 - 9) | 30 | 10 | (6 - 13) |
|  | > - 2 | 291 | 94 | (91 - 97) | 281 | 90 | (87 - 94) |
| Height for Age |  |  |  |  |  |  |  |
|  | < = -2 | 52 | 17 | (13 - 21) | 49 | 16 | (12 - 20) |
|  | > - 2 | 258 | 83 | (79 - 87) | 262 | 84 | (80 - 88) |
| Hookworm |  |  |  |  |  |  |  |
|  | 0 – No | 145 | 47 | (41 - 52) | 124 | 40 | (34 - 45) |
|  | 1 – 384 | 148 | 48 | (42 - 53) | 158 | 51 | (45 - 56) |
|  | > 384 | 17 | 6 | (3 - 8) | 29 | 9 | (6 - 13) |
| Trichuris |  |  |  |  |  |  |  |
|  | 0 – No | 211 | 68 | (63 - 73) | 194 | 62 | (57 - 68) |
|  | 1 – 288 | 89 | 29 | (24 - 34) | 107 | 34 | (29 - 40) |
|  | > 288 | 10 | 3 | (1 - 5) | 10 | 3 | (1 - 5) |
| *A Lumbricoides* |  |  |  |  |  |  |  |
|  | 0 - No | 274 | 88 | (85 - 92) | 269 | 87 | (83 - 90) |
|  | 1 – 1776 | 21 | 7 | (4 - 10) | 22 | 7 | (4 - 10) |
|  | > 1776 | 15 | 5 | (2 - 7) | 20 | 6 | (4 - 9) |
| Anyworm |  |  |  |  |  |  |  |
|  | 0 | 95 | 31 | (25 - 38) | 79 | 25 | (21 - 30) |
|  | 1 | 202 | 65 | (60 - 70) | 215 | 69 | (64 - 74) |
|  | 2 | 13 | 4 | (2 - 6) | 17 | 6 | (3 - 8) |
| Anemia |  |  |  |  |  |  |  |
|  | No | 133 | 43 | (37 - 48) | 135 | 43 | (38 - 49) |
|  | Yes | 177 | 57 | (52 - 63) | 176 | 57 | (51 - 62) |
| Age |  |  |  |  |  |  |  |
|  | < = 5 | 0 | 0 | (0) | 1 | 0.3 | (0) |
|  | > 5 | 310 | 100 | (100) | 310 | 99.7 | (100) |
| Sex |  |  |  |  |  |  |  |
|  | Male | 157 | 51 | (45 - 56) | 164 | 53 | (47 - 58) |
|  | Female | 153 | 49 | (44 - 55) | 147 | 47 | (42 - 53) |
| Total (n) |  | 310 |  |  | 311 |  |  |
| Number of studies | 2 | | | | | | |

Table l. Head to Head (STH + MCN/Iron vs MCN/Iron)

| Variable | | STH + MCN/Iron | | | MCN/Iron | | |
| --- | --- | --- | --- | --- | --- | --- | --- |
|  |  | (n) | (%) | C.I (95%) | (n) | (%) | C.I (95%) |
| BMI for Age |  |  |  |  |  |  |  |
|  | < = -2 | 86 | 14 | (11 - 17) | 74 | 14 | (11 - 17) |
|  | > - 2 | 524 | 86 | (83 - 89) | 469 | 86 | (83 - 89) |
| Height for Age |  |  |  |  |  |  |  |
|  | < = -2 | 214 | 35 | (31 - 39) | 203 | 37 | (33 - 41) |
|  | > - 2 | 396 | 65 | (61 - 69) | 340 | 63 | (59 - 67) |
| Hookworm |  |  |  |  |  |  |  |
|  | 0 – No | 473 | 78 | (74 - 81) | 409 | 75 | (72 - 79) |
|  | 1 – 384 | 122 | 20 | (17 - 23) | 126 | 23 | (20 - 27) |
|  | > 384 | 15 | 2 | (1 - 4) | 8 | 2 | (0 - 2) |
| Trichuris |  |  |  |  |  |  |  |
|  | 0 – No | 256 | 42 | (38 - 46) | 243 | 45 | (41 - 49) |
|  | 1 – 288 | 230 | 38 | (34 - 42) | 220 | 41 | (36 - 45) |
|  | > 288 | 124 | 20 | (17 - 24) | 80 | 15 | (12 - 18) |
| *A Lumbricoides* |  |  |  |  |  |  |  |
|  | 0 - No | 250 | 41 | (37 - 45) | 243 | 45 | (41 - 49) |
|  | 1 – 1776 | 164 | 27 | (23 - 30) | 150 | 28 | (24 - 31) |
|  | > 1776 | 196 | 32 | (28 - 36) | 150 | 28 | (24 - 31) |
| Anyworm |  |  |  |  |  |  |  |
|  | 0 | 137 | 22 | (19 - 26) | 139 | 26 | (22 - 29) |
|  | 1 | 310 | 51 | (47 - 55) | 286 | 53 | (48 - 57) |
|  | 2 | 163 | 27 | (23 - 30) | 118 | 22 | (18 - 25) |
| Anemia |  |  |  |  |  |  |  |
|  | No | 353 | 58 | (54 - 62) | 301 | 55 | (51 - 60) |
|  | Yes | 257 | 42 | (38 - 46) | 242 | 45 | (40 - 49) |
| Age |  |  |  |  |  |  |  |
|  | < = 5 | 107 | 18 | (15 - 21) | 108 | 20 | (17 - 23) |
|  | > 5 | 503 | 82 | (79 - 85) | 435 | 80 | (77 - 83) |
| Sex |  |  |  |  |  |  |  |
|  | Male | 314 | 51 | (47 - 55) | 280 | 52 | (47 - 56) |
|  | Female | 296 | 49 | (45 - 53) | 263 | 48 | (44 - 53) |
| Total (n) |  | 610 |  |  | 543 |  |  |
| Number of studies | 4 | | | | | | |

Note: There are no direct head to head comparisons for: 2 vs 4, 3 vs 5, 4 vs 5

Additional tables 15: Effect modifier analyses for weight, height and haemoglobin

**Effect modifiers**

**BAZ (≥2)**

| treatment | comparison | weight | height | haemoglobin |
| --- | --- | --- | --- | --- |
| STH deworming with any drug | Placebo or control | 0.01(-0.08,0.11) | 0.09(-0.11,0.28) | 0.40(-0.56,1.37) |
| Any STH deworming combination with praziquantel | Placebo or control | 0.04(-0.12,0.20) | -0.05(-0.32,0.22) | 1.82(0.44,3.19) |
| Any STH deworming combination with praziquantel with iron or micronutrients | Placebo or control | -0.01(-0.25,0.23) | -0.02(-0.37,0.33) | 2.76(0.98,4.54) |
| Any STH deworming with micronutrients or iron | Placebo or control | -0.02(-0.16,0.13) | 0.02(-0.24,0.29) | 2.23(0.88,3.57) |
| Micronutrients or iron alone | Placebo or control | 0.01(-0.14,0.16) | -0.03(-0.28,0.22) | 1.12(-0.16,2.40) |
| Any STH deworming combination with praziquantel | STH deworming with any drug | 0.03(-0.15,0.20) | -0.14(-0.43,0.15) | 1.41(-0.18,3.01) |
| Any STH deworming combination with praziquantel with iron or micronutrients | STH deworming with any drug | -0.02(-0.28,0.24) | -0.11(-0.46,0.25) | 2.36(0.34,4.37) |
| Any STH deworming with micronutrients or iron | STH deworming with any drug | -0.03(-0.18,0.13) | -0.06(-0.34,0.21) | 1.82(0.35,3.29) |
| Micronutrients or iron alone | STH deworming with any drug | 0.00(-0.16,0.15) | -0.12(-0.37,0.14) | 0.72(-0.72,2.15) |
| Any STH deworming combination with praziquantel with iron or micronutrients | Any STH deworming combination with praziquantel | -0.05(-0.29,0.19) | 0.03(-0.31,0.38) | 0.94(-1.02,2.90) |
| Any STH deworming with micronutrients or iron | Any STH deworming combination with praziquantel | -0.06(-0.26,0.15) | 0.08(-0.27,0.42) | 0.41(-1.45,2.26) |
| Micronutrients or iron alone | Any STH deworming combination with praziquantel | -0.03(-0.22,0.15) | 0.02(-0.28,0.32) | -0.70(-2.27,0.88) |
| Any STH deworming with micronutrients or iron | Any STH deworming combination with praziquantel with iron or micronutrients | -0.01(-0.28,0.27) | 0.04(-0.36,0.45) | -0.54(-2.70,1.63) |
| Micronutrients or iron alone | Any STH deworming combination with praziquantel with iron or micronutrients | 0.02(-0.22,0.26) | -0.01(-0.37,0.34) | -1.64(-3.61,0.33) |
| Micronutrients or iron alone | Any STH deworming with micronutrients or iron | 0.03(-0.15,0.20) | -0.05(-0.34,0.23) | -1.10(-2.63,0.42) |

**BAZ (≤2)**

| treatment | comparison | weight | height | haemoglobin |
| --- | --- | --- | --- | --- |
| STH deworming with any drug | Placebo or control | -0.02  (-0.24,0.21) | -0.13  (-0.70,0.43) | -0.63  (-2.90,1.64) |
| Any STH deworming combination with praziquantel | Placebo or control | -0.10(-0.62,0.43) | -0.39(-1.17,0.39) | 1.82(-2.70,6.35) |
| Any STH deworming combination with praziquantel with iron or micronutrients | Placebo or control | -0.30(-1.16,0.56) | -0.05(-1.20,1.11) | 2.12(-4.35,8.59) |
| Any STH deworming with micronutrients or iron | Placebo or control | 0.01(-0.22,0.23) | -0.15(-0.67,0.36) | 1.26(-0.91,3.44) |
| Micronutrients or iron alone | Placebo or control | -0.23(-0.61,0.14) | -0.33(-0.94,0.28) | 2.58(-0.61,5.76) |
| Any STH deworming combination with praziquantel | STH deworming with any drug | -0.08(-0.62,0.46) | -0.26(-1.20,0.69) | 2.45(-2.39,7.30) |
| Any STH deworming combination with praziquantel with iron or micronutrients | STH deworming with any drug | -0.28(-1.15,0.59) | 0.09(-1.10,1.27) | 2.75(-3.82,9.33) |
| Any STH deworming with micronutrients or iron | STH deworming with any drug | 0.02(-0.27,0.32) | -0.02(-0.62,0.58) | 1.90(-0.87,4.66) |
| Micronutrients or iron alone | STH deworming with any drug | -0.22(-0.61,0.18) | -0.20(-0.80,0.41) | 3.21(-0.14,6.56) |
| Any STH deworming combination with praziquantel with iron or micronutrients | Any STH deworming combination with praziquantel | -0.20(-1.10,0.70) | 0.34(-0.87,1.56) | 0.30(-6.72,7.32) |
| Any STH deworming with micronutrients or iron | Any STH deworming combination with praziquantel | 0.10(-0.45,0.66) | 0.24(-0.69,1.16) | -0.56(-5.54,4.43) |
| Micronutrients or iron alone | Any STH deworming combination with praziquantel | -0.14(-0.72,0.44) | 0.06(-0.87,0.99) | 0.76(-4.18,5.69) |
| Any STH deworming with micronutrients or iron | Any STH deworming combination with praziquantel with iron or micronutrients | 0.31(-0.54,1.16) | -0.10(-1.32,1.11) | -0.86(-7.51,5.79) |
| Micronutrients or iron alone | Any STH deworming combination with praziquantel with iron or micronutrients | 0.07(-0.77,0.90) | -0.28(-1.47,0.90) | 0.46(-6.10,7.01) |
| Micronutrients or iron alone | Any STH deworming with micronutrients or iron | -0.24(-0.64,0.16) | -0.18(-0.83,0.47) | 1.31(-2.08,4.71) |

HAZ (>-2)

| treatment | comparison | weight | height | haemoglobin |
| --- | --- | --- | --- | --- |
| STH deworming with any drug | Placebo or control | 0.05(-0.06,0.15) | 0.03(-0.17,0.23) | 0.09(-1.08,1.26) |
| Any STH deworming combination with praziquantel | Placebo or control | -0.01(-0.20,0.18) | -0.12(-0.40,0.16) | 2.08(0.51,3.65) |
| Any STH deworming combination with praziquantel with iron or micronutrients | Placebo or control | -0.06(-0.33,0.20) | -0.11(-0.48,0.25) | 2.78(0.89,4.66) |
| Any STH deworming with micronutrients or iron | Placebo or control | -0.02(-0.19,0.14) | 0.00(-0.28,0.27) | 1.71(0.32,3.11) |
| Micronutrients or iron alone | Placebo or control | -0.02(-0.19,0.16) | -0.14(-0.41,0.13) | 1.37(0.06,2.68) |
| Any STH deworming combination with praziquantel | STH deworming with any drug | -0.06(-0.26,0.15) | -0.15(-0.46,0.16) | 1.99(0.11,3.87) |
| Any STH deworming combination with praziquantel with iron or micronutrients | STH deworming with any drug | -0.11(-0.40,0.18) | -0.14(-0.51,0.23) | 2.69(0.56,4.81) |
| Any STH deworming with micronutrients or iron | STH deworming with any drug | -0.07(-0.25,0.10) | -0.03(-0.33,0.26) | 1.63(0.05,3.20) |
| Micronutrients or iron alone | STH deworming with any drug | -0.06(-0.26,0.13) | -0.17(-0.45,0.11) | 1.28(-0.28,2.85) |
| Any STH deworming combination with praziquantel with iron or micronutrients | Any STH deworming combination with praziquantel | -0.06(-0.33,0.22) | 0.01(-0.35,0.37) | 0.70(-1.32,2.71) |
| Any STH deworming with micronutrients or iron | Any STH deworming combination with praziquantel | -0.02(-0.25,0.22) | 0.12(-0.25,0.49) | -0.37(-2.41,1.67) |
| Micronutrients or iron alone | Any STH deworming combination with praziquantel | -0.01(-0.23,0.21) | -0.02(-0.34,0.31) | -0.71(-2.43,1.02) |
| Any STH deworming with micronutrients or iron | Any STH deworming combination with praziquantel with iron or micronutrients | 0.04(-0.26,0.34) | 0.11(-0.31,0.53) | -1.06(-3.34,1.22) |
| Micronutrients or iron alone | Any STH deworming combination with praziquantel with iron or micronutrients | 0.05(-0.20,0.30) | -0.02(-0.39,0.34) | -1.41(-3.40,0.59) |
| Micronutrients or iron alone | Any STH deworming with micronutrients or iron | 0.01(-0.20,0.21) | -0.13(-0.44,0.18) | -0.34(-1.97,1.28) |

Effect modifier

HAZ (<-2)

| treatment | comparison | weight | height | haemoglobin |
| --- | --- | --- | --- | --- |
| STH deworming with any drug | Placebo or control | -0.04(-0.20,0.11) | 0.30(-0.01,0.61) | 0.94(-0.54,2.41) |
| Any STH deworming combination with praziquantel | Placebo or control | 0.18(-0.10,0.47) | 0.02(-0.47,0.52) | 1.44(-1.35,4.24) |
| Any STH deworming combination with praziquantel with iron or micronutrients | Placebo or control | 0.07(-0.47,0.61) | 0.11(-0.61,0.83) | 2.45(-1.44,6.35) |
| Any STH deworming with micronutrients or iron | Placebo or control | -0.04(-0.24,0.16) | -0.01(-0.43,0.41) | 2.74(0.68,4.81) |
| Micronutrients or iron alone | Placebo or control | -0.05(-0.28,0.18) | 0.08(-0.34,0.50) | 1.30(-1.13,3.74) |
| Any STH deworming combination with praziquantel | STH deworming with any drug | 0.23(-0.09,0.55) | -0.28(-0.82,0.27) | 0.51(-2.52,3.54) |
| Any STH deworming combination with praziquantel with iron or micronutrients | STH deworming with any drug | 0.12(-0.41,0.64) | -0.19(-0.95,0.58) | 1.52(-2.60,5.63) |
| Any STH deworming with micronutrients or iron | STH deworming with any drug | 0.01(-0.21,0.23) | -0.31(-0.75,0.14) | 1.80(-0.32,3.93) |
| Micronutrients or iron alone | STH deworming with any drug | -0.01(-0.25,0.24) | -0.22(-0.66,0.22) | 0.36(-2.10,2.83) |
| Any STH deworming combination with praziquantel with iron or micronutrients | Any STH deworming combination with praziquantel | -0.11(-0.68,0.45) | 0.09(-0.66,0.83) | 1.01(-3.06,5.08) |
| Any STH deworming with micronutrients or iron | Any STH deworming combination with praziquantel | -0.22(-0.57,0.12) | -0.03(-0.66,0.60) | 1.30(-2.24,4.83) |
| Micronutrients or iron alone | Any STH deworming combination with praziquantel | -0.24(-0.59,0.11) | 0.06(-0.56,0.67) | -0.14(-3.84,3.55) |
| Any STH deworming with micronutrients or iron | Any STH deworming combination with praziquantel with iron or micronutrients | -0.11(-0.66,0.45) | -0.12(-0.93,0.70) | 0.29(-4.14,4.71) |
| Micronutrients or iron alone | Any STH deworming combination with praziquantel with iron or micronutrients | -0.12(-0.68,0.43) | -0.03(-0.82,0.75) | -1.15(-5.64,3.33) |
| Micronutrients or iron alone | Any STH deworming with micronutrients or iron | -0.02(-0.27,0.24) | 0.09(-0.39,0.56) | -1.44(-3.98,1.10) |

**Effect modifier**

**Sex (Female)**

| treatment | comparison | weight | height | haemoglobin |
| --- | --- | --- | --- | --- |
| STH deworming with any drug | Placebo or control | 0.04(-0.08,0.17) | 0.04(-0.18,0.27) | 0.32(-0.86,1.50) |
| Any STH deworming combination with praziquantel | Placebo or control | 0.19(-0.04,0.41) | 0.00(-0.35,0.35) | 1.64(-0.30,3.58) |
| Any STH deworming combination with praziquantel with iron or micronutrients | Placebo or control | -0.21(-0.62,0.19) | -0.06(-0.49,0.38) | 3.11(0.69,5.53) |
| Any STH deworming with micronutrients or iron | Placebo or control | 0.02(-0.16,0.20) | -0.09(-0.39,0.21) | 2.04(0.39,3.69) |
| Micronutrients or iron alone | Placebo or control | -0.04(-0.24,0.16) | -0.13(-0.42,0.16) | 1.50(-0.10,3.10) |
| Any STH deworming combination with praziquantel | STH deworming with any drug | 0.14(-0.11,0.39) | -0.04(-0.42,0.33) | 1.32(-0.85,3.49) |
| Any STH deworming combination with praziquantel with iron or micronutrients | STH deworming with any drug | -0.25(-0.70,0.19) | -0.10(-0.56,0.36) | 2.79(0.11,5.48) |
| Any STH deworming with micronutrients or iron | STH deworming with any drug | -0.02(-0.23,0.18) | -0.13(-0.46,0.19) | 1.72(-0.14,3.57) |
| Micronutrients or iron alone | STH deworming with any drug | -0.09(-0.30,0.13) | -0.18(-0.50,0.15) | 1.18(-0.60,2.96) |
| Any STH deworming combination with praziquantel with iron or micronutrients | Any STH deworming combination with praziquantel | -0.40(-0.81,0.02) | -0.06(-0.51,0.39) | 1.47(-1.20,4.14) |
| Any STH deworming with micronutrients or iron | Any STH deworming combination with praziquantel | -0.17(-0.45,0.11) | -0.09(-0.52,0.34) | 0.40(-1.99,2.78) |
| Micronutrients or iron alone | Any STH deworming combination with praziquantel | -0.23(-0.50,0.04) | -0.13(-0.53,0.26) | -0.14(-2.32,2.03) |
| Any STH deworming with micronutrients or iron | Any STH deworming combination with praziquantel with iron or micronutrients | 0.23(-0.21,0.67) | -0.03(-0.53,0.47) | -1.07(-3.87,1.72) |
| Micronutrients or iron alone | Any STH deworming combination with praziquantel with iron or micronutrients | 0.17(-0.26,0.60) | -0.08(-0.53,0.38) | -1.61(-4.16,0.93) |
| Micronutrients or iron alone | Any STH deworming with micronutrients or iron | -0.06(-0.29,0.17) | -0.04(-0.39,0.30) | -0.54(-2.44,1.36) |

**Sex (Male)**

| treatment | comparison | weight | height | haemoglobin |
| --- | --- | --- | --- | --- |
| STH deworming with any drug | Placebo or control | -0.01(-0.13,0.12) | 0.13(-0.09,0.36) | 0.28(-0.93,1.50) |
| Any STH deworming combination with praziquantel | Placebo or control | -0.08(-0.29,0.13) | -0.15(-0.50,0.21) | 1.96(0.19,3.73) |
| Any STH deworming combination with praziquantel with iron or micronutrients | Placebo or control | 0.13(-0.20,0.46) | -0.05(-0.50,0.40) | 2.46(0.11,4.82) |
| Any STH deworming with micronutrients or iron | Placebo or control | -0.05(-0.22,0.13) | 0.04(-0.30,0.37) | 2.26(0.68,3.84) |
| Micronutrients or iron alone | Placebo or control | 0.00(-0.19,0.19) | -0.01(-0.33,0.31) | 1.20(-0.51,2.91) |
| Any STH deworming combination with praziquantel | STH deworming with any drug | -0.07(-0.29,0.15) | -0.28(-0.66,0.10) | 1.68(-0.47,3.82) |
| Any STH deworming combination with praziquantel with iron or micronutrients | STH deworming with any drug | 0.14(-0.21,0.48) | -0.19(-0.67,0.30) | 2.18(-0.42,4.79) |
| Any STH deworming with micronutrients or iron | STH deworming with any drug | -0.04(-0.23,0.15) | -0.10(-0.45,0.26) | 1.98(0.25,3.71) |
| Micronutrients or iron alone | STH deworming with any drug | 0.01(-0.19,0.21) | -0.14(-0.48,0.19) | 0.92(-1.05,2.89) |
| Any STH deworming combination with praziquantel with iron or micronutrients | Any STH deworming combination with praziquantel | 0.21(-0.10,0.52) | 0.10(-0.38,0.57) | 0.50(-2.00,3.01) |
| Any STH deworming with micronutrients or iron | Any STH deworming combination with praziquantel | 0.03(-0.23,0.29) | 0.18(-0.27,0.63) | 0.30(-2.03,2.63) |
| Micronutrients or iron alone | Any STH deworming combination with praziquantel | 0.08(-0.17,0.33) | 0.14(-0.27,0.54) | -0.76(-2.88,1.36) |
| Any STH deworming with micronutrients or iron | Any STH deworming combination with praziquantel with iron or micronutrients | -0.18(-0.54,0.18) | 0.09(-0.44,0.62) | -0.20(-2.97,2.57) |
| Micronutrients or iron alone | Any STH deworming combination with praziquantel with iron or micronutrients | -0.13(-0.48,0.23) | 0.04(-0.43,0.52) | -1.26(-3.86,1.33) |
| Micronutrients or iron alone | Any STH deworming with micronutrients or iron | 0.05(-0.17,0.27) | -0.05(-0.42,0.33) | -1.06(-3.05,0.93) |

**Effect modifier**

**Age (>5years**)

| treatment | comparison | weight | height | haemoglobin |
| --- | --- | --- | --- | --- |
| STH deworming with any drug | Placebo or control | 0.06(-0.07,0.20) | 0.03(-0.19,0.24) | 0.33(-0.79,1.44) |
| Any STH deworming combination with praziquantel | Placebo or control | 0.11(-0.06,0.28) | -0.08(-0.31,0.15) | 1.93(0.61,3.26) |
| Any STH deworming combination with praziquantel with iron or micronutrients | Placebo or control | 0.05(-0.21,0.30) | -0.05(-0.36,0.25) | 2.83(1.17,4.49) |
| Any STH deworming with micronutrients or iron | Placebo or control | -0.02(-0.17,0.14) | -0.06(-0.30,0.19) | 2.10(0.78,3.41) |
| Micronutrients or iron alone | Placebo or control | 0.04(-0.12,0.20) | -0.08(-0.31,0.15) | 1.33(0.09,2.57) |
| Any STH deworming combination with praziquantel | STH deworming with any drug | 0.05(-0.15,0.25) | -0.10(-0.39,0.18) | 1.61(0.01,3.20) |
| Any STH deworming combination with praziquantel with iron or micronutrients | STH deworming with any drug | -0.02(-0.28,0.25) | -0.08(-0.43,0.27) | 2.50(0.61,4.40) |
| Any STH deworming with micronutrients or iron | STH deworming with any drug | -0.08(-0.26,0.10) | -0.08(-0.34,0.18) | 1.77(0.33,3.20) |
| Micronutrients or iron alone | STH deworming with any drug | -0.02(-0.20,0.16) | -0.11(-0.36,0.15) | 1.00(-0.41,2.41) |
| Any STH deworming combination with praziquantel with iron or micronutrients | Any STH deworming combination with praziquantel | -0.06(-0.32,0.19) | 0.02(-0.29,0.33) | 0.90(-0.93,2.73) |
| Any STH deworming with micronutrients or iron | Any STH deworming combination with praziquantel | -0.13(-0.34,0.09) | 0.02(-0.29,0.33) | 0.16(-1.64,1.96) |
| Micronutrients or iron alone | Any STH deworming combination with praziquantel | -0.07(-0.27,0.13) | 0.00(-0.27,0.27) | -0.61(-2.11,0.90) |
| Any STH deworming with micronutrients or iron | Any STH deworming combination with praziquantel with iron or micronutrients | -0.06(-0.34,0.22) | 0.00(-0.37,0.36) | -0.74(-2.75,1.27) |
| Micronutrients or iron alone | Any STH deworming combination with praziquantel with iron or micronutrients | -0.01(-0.25,0.24) | -0.03(-0.34,0.29) | -1.51(-3.34,0.32) |
| Micronutrients or iron alone | Any STH deworming with micronutrients or iron | 0.06(-0.13,0.24) | -0.03(-0.29,0.24) | -0.77(-2.24,0.71) |

**Age (<5years)**

| treatment | comparison | weight | height | haemoglobin |
| --- | --- | --- | --- | --- |
| STH deworming with any drug | Placebo or control | -0.02(-0.12,0.08) | 0.18(-0.14,0.49) | 0.37(-1.13,1.86) |
| Any STH deworming combination with praziquantel | Placebo or control | 0.08(-4.51,4.67) | -2.04(-9.22,5.13) | 14.19(-46.06,74.45) |
| Any STH deworming combination with praziquantel with iron or micronutrients | Placebo or control | -0.02(-0.26,0.22) | -0.02(-0.78,0.73) | 1.73(-1.99,5.45) |
| Any STH deworming with micronutrients or iron | Placebo or control | -0.18(-0.42,0.05) | -0.07(-0.84,0.70) | 1.79(-2.08,5.65) |
| Micronutrients or iron alone | Placebo or control | 0.10(-4.51,4.71) | -2.22(-9.39,4.95) | 13.83(-45.84,73.49) |
| Any STH deworming combination with praziquantel | STH deworming with any drug | 0.00(-0.23,0.22) | -0.20(-0.94,0.54) | 1.36(-2.38,5.11) |
| Any STH deworming combination with praziquantel with iron or micronutrients | STH deworming with any drug | -0.17(-0.41,0.08) | -0.25(-1.01,0.51) | 1.42(-2.42,5.27) |
| Any STH deworming with micronutrients or iron | STH deworming with any drug | -0.10(-4.73,4.53) | 2.02(-5.17,9.21) | -12.46(-72.59,47.66) |
| Micronutrients or iron alone | STH deworming with any drug | -0.27(-4.83,4.30) | 1.97(-5.28,9.22) | -12.41(-73.03,48.22) |
| Any STH deworming combination with praziquantel with iron or micronutrients | Any STH deworming combination with praziquantel | -0.16(-0.46,0.13) | -0.05(-0.92,0.82) | 0.06(-4.09,4.21) |
| Any STH deworming with micronutrients or iron | Any STH deworming combination with praziquantel | 0.00(0.00,0.00) | 0.00(0.00,0.00) | 0.00(0.00,0.00) |
| Micronutrients or iron alone | Any STH deworming combination with praziquantel | 0.00(0.00,0.00) | 0.00(0.00,0.00) | 0.00(0.00,0.00) |
| Any STH deworming with micronutrients or iron | Any STH deworming combination with praziquantel with iron or micronutrients | 0.00(0.00,0.00) | 0.00(0.00,0.00) | 0.00(0.00,0.00) |
| Micronutrients or iron alone | Any STH deworming combination with praziquantel with iron or micronutrients | 0.00(0.00,0.00) | 0.00(0.00,0.00) | 0.00(0.00,0.00) |
| Micronutrients or iron alone | Any STH deworming with micronutrients or iron | 0.00(0.00,0.00) | 0.000.00,0.00) | 0.00(0.00,0.00) |

**Effect modifier**

***A Lumbricoides*  – no infection**

| treatment | comparison | weight | height | haemoglobin |
| --- | --- | --- | --- | --- |
| STH deworming with any drug | Placebo or control | 0.00(-0.11,0.12) | 0.07(-0.14,0.27) | 0.48(-0.69,1.66) |
| Any STH deworming combination with praziquantel | Placebo or control | 0.10(-0.09,0.29) | -0.10(-0.39,0.18) | 1.86(0.35,3.38) |
| Any STH deworming combination with praziquantel with iron or micronutrients | Placebo or control | 0.05(-0.21,0.31) | -0.03(-0.39,0.33) | 2.50(0.75,4.24) |
| Any STH deworming with micronutrients or iron | Placebo or control | 0.02(-0.16,0.20) | -0.07(-0.40,0.26) | 2.43(0.72,4.15) |
| Micronutrients or iron alone | Placebo or control | -0.04(-0.21,0.14) | -0.12(-0.41,0.17) | 1.19(-0.24,2.61) |
| Any STH deworming combination with praziquantel | STH deworming with any drug | 0.09(-0.12,0.31) | -0.17(-0.50,0.16) | 1.38(-0.50,3.26) |
| Any STH deworming combination with praziquantel with iron or micronutrients | STH deworming with any drug | 0.05(-0.23,0.32) | -0.09(-0.48,0.29) | 2.01(0.05,3.98) |
| Any STH deworming with micronutrients or iron | STH deworming with any drug | 0.02(-0.18,0.22) | -0.14(-0.49,0.21) | 1.95(0.00,3.90) |
| Micronutrients or iron alone | STH deworming with any drug | -0.04(-0.24,0.16) | -0.19(-0.50,0.13) | 0.70(-1.06,2.47) |
| Any STH deworming combination with praziquantel with iron or micronutrients | Any STH deworming combination with praziquantel | -0.05(-0.30,0.20) | 0.08(-0.29,0.44) | 0.63(-1.32,2.58) |
| Any STH deworming with micronutrients or iron | Any STH deworming combination with praziquantel | -0.08(-0.32,0.17) | 0.03(-0.38,0.45) | 0.57(-1.55,2.70) |
| Micronutrients or iron alone | Any STH deworming combination with praziquantel | -0.13(-0.35,0.08) | -0.02(-0.35,0.32) | -0.68(-2.36,1.00) |
| Any STH deworming with micronutrients or iron | Any STH deworming combination with praziquantel with iron or micronutrients | -0.03(-0.33,0.27) | -0.04(-0.51,0.42) | -0.06(-2.45,2.33) |
| Micronutrients or iron alone | Any STH deworming combination with praziquantel with iron or micronutrients | -0.08(-0.34,0.17) | -0.09(-0.47,0.29) | -1.31(-3.24,0.62) |
| Micronutrients or iron alone | Any STH deworming with micronutrients or iron | -0.06(-0.28,0.16) | -0.05(-0.42,0.33) | -1.25(-3.15,0.65) |

***A Lumbricoides*  – light infection**

| treatment | comparison | weight | height | haemoglobin |
| --- | --- | --- | --- | --- |
| STH deworming with any drug | Placebo or control | 0.07(-0.13,0.27) | 0.25(-0.14,0.63) | -0.10(-1.75,1.55) |
| Any STH deworming combination with praziquantel | Placebo or control | -0.10(-0.82,0.61) | -0.19(-1.36,0.97) | 1.89(-3.64,7.43) |
| Any STH deworming combination with praziquantel with iron or micronutrients | Placebo or control | -0.69(-1.58,0.19) | -0.82(-2.32,0.68) | 1.69(-6.67,10.06) |
| Any STH deworming with micronutrients or iron | Placebo or control | -0.19(-0.48,0.10) | -0.02(-0.55,0.50) | 1.70(-0.86,4.26) |
| Micronutrients or iron alone | Placebo or control | 0.00(-0.31,0.31) | 0.01(-0.55,0.57) | 2.50(-0.48,5.48) |
| Any STH deworming combination with praziquantel | STH deworming with any drug | -0.17(-0.89,0.55) | -0.44(-1.57,0.69) | 1.99(-3.48,7.46) |
| Any STH deworming combination with praziquantel with iron or micronutrients | STH deworming with any drug | -0.76(-1.68,0.16) | -1.07(-2.61,0.48) | 1.80(-6.59,10.18) |
| Any STH deworming with micronutrients or iron | STH deworming with any drug | -0.26(-0.56,0.04) | -0.27(-0.83,0.28) | 1.80(-0.76,4.36) |
| Micronutrients or iron alone | STH deworming with any drug | -0.07(-0.40,0.27) | -0.23(-0.82,0.35) | 2.60(-0.28,5.48) |
| Any STH deworming combination with praziquantel with iron or micronutrients | Any STH deworming combination with praziquantel | -0.59(-1.53,0.35) | -0.63(-2.36,1.11) | -0.20(-8.50,8.11) |
| Any STH deworming with micronutrients or iron | Any STH deworming combination with praziquantel | -0.08(-0.85,0.69) | 0.17(-1.05,1.39) | -0.19(-6.05,5.66) |
| Micronutrients or iron alone | Any STH deworming combination with praziquantel | 0.11(-0.63,0.84) | 0.21(-1.00,1.42) | 0.61(-5.20,6.42) |
| Any STH deworming with micronutrients or iron | Any STH deworming combination with praziquantel with iron or micronutrients | 0.51(-0.44,1.45) | 0.80(-0.76,2.35) | 0.01(-8.74,8.75) |
| Micronutrients or iron alone | Any STH deworming combination with praziquantel with iron or micronutrients | 0.70(-0.18,1.57) | 0.83(-0.68,2.35) | 0.80(-7.67,9.28) |
| Micronutrients or iron alone | Any STH deworming with micronutrients or iron | 0.19(-0.16,0.54) | 0.04(-0.57,0.65) | 0.80(-2.60,4.20) |

***A Lumbricoides*  – high infection**

| treatment | comparison | weight | height | haemoglobin |
| --- | --- | --- | --- | --- |
| STH deworming with any drug | Placebo or control | 0.08(-0.13,0.29) | 0.04(-0.22,0.30) | 0.03(-2.08,2.14) |
| Any STH deworming combination with praziquantel | Placebo or control | -0.17(-0.53,0.19) | 0.00(-0.45,0.45) | 1.73(-1.41,4.87) |
| Any STH deworming combination with praziquantel with iron or micronutrients | Placebo or control | -0.52(-1.34,0.30) | -0.40(-1.43,0.63) | 7.37(-0.24,14.98) |
| Any STH deworming with micronutrients or iron | Placebo or control | 0.06(-0.18,0.30) | -0.06(-0.36,0.24) | 2.85(0.49,5.21) |
| Micronutrients or iron alone | Placebo or control | 0.04(-0.26,0.34) | -0.17(-0.55,0.20) | 0.84(-1.74,3.43) |
| Any STH deworming combination with praziquantel | STH deworming with any drug | -0.25(-0.67,0.16) | -0.04(-0.54,0.46) | 1.70(-2.09,5.49) |
| Any STH deworming combination with praziquantel with iron or micronutrients | STH deworming with any drug | -0.60(-1.39,0.20) | -0.44(-1.53,0.65) | 7.34(-0.41,15.09) |
| Any STH deworming with micronutrients or iron | STH deworming with any drug | -0.02(-0.27,0.23) | -0.10(-0.45,0.24) | 2.82(0.44,5.20) |
| Micronutrients or iron alone | STH deworming with any drug | -0.04(-0.33,0.25) | -0.22(-0.62,0.19) | 0.81(-2.02,3.65) |
| Any STH deworming combination with praziquantel with iron or micronutrients | Any STH deworming combination with praziquantel | -0.34(-1.15,0.46) | -0.40(-1.44,0.65) | 5.64(-2.64,13.93) |
| Any STH deworming with micronutrients or iron | Any STH deworming combination with praziquantel | 0.23(-0.17,0.63) | -0.06(-0.59,0.47) | 1.12(-2.82,5.07) |
| Micronutrients or iron alone | Any STH deworming combination with praziquantel | 0.21(-0.22,0.64) | -0.17(-0.73,0.38) | -0.88(-4.80,3.03) |
| Any STH deworming with micronutrients or iron | Any STH deworming combination with praziquantel with iron or micronutrients | 0.58(-0.23,1.39) | 0.34(-0.72,1.39) | -4.52(-12.36,3.32) |
| Micronutrients or iron alone | Any STH deworming combination with praziquantel with iron or micronutrients | 0.56(-0.26,1.37) | 0.23(-0.82,1.27) | -6.53(-14.40,1.35) |
| Micronutrients or iron alone | Any STH deworming with micronutrients or iron | -0.02(-0.30,0.26) | -0.11(-0.51,0.29) | -2.01(-4.86,0.84) |

**Effect modifier**

**Hookworm – no infection**

| treatment | comparison | weight | height | haemoglobin |
| --- | --- | --- | --- | --- |
| STH deworming with any drug | Placebo or control | 0.02(-0.09,0.13) | 0.06(-0.13,0.26) | 0.07(-0.92,1.06) |
| Any STH deworming combination with praziquantel | Placebo or control | 0.00(-0.34,0.33) | -0.13(-0.61,0.35) | 0.59(-1.72,2.89) |
| Any STH deworming combination with praziquantel with iron or micronutrients | Placebo or control | -0.04(-0.41,0.33) | -0.14(-0.67,0.39) | 1.98(-0.66,4.63) |
| Any STH deworming with micronutrients or iron | Placebo or control | -0.04(-0.19,0.11) | -0.09(-0.37,0.18) | 1.94(0.59,3.29) |
| Micronutrients or iron alone | Placebo or control | 0.00(-0.16,0.17) | -0.10(-0.38,0.18) | 0.80(-0.56,2.16) |
| Any STH deworming combination with praziquantel | STH deworming with any drug | -0.02(-0.38,0.33) | -0.20(-0.70,0.31) | 0.52(-1.94,2.98) |
| Any STH deworming combination with praziquantel with iron or micronutrients | STH deworming with any drug | -0.06(-0.45,0.33) | -0.21(-0.75,0.34) | 1.92(-0.74,4.58) |
| Any STH deworming with micronutrients or iron | STH deworming with any drug | -0.06(-0.22,0.10) | -0.16(-0.45,0.13) | 1.88(0.45,3.30) |
| Micronutrients or iron alone | STH deworming with any drug | -0.02(-0.19,0.15) | -0.17(-0.47,0.14) | 0.74(-0.72,2.19) |
| Any STH deworming combination with praziquantel with iron or micronutrients | Any STH deworming combination with praziquantel | -0.03(-0.39,0.33) | -0.01(-0.58,0.56) | 1.40(-1.44,4.24) |
| Any STH deworming with micronutrients or iron | Any STH deworming combination with praziquantel | -0.03(-0.40,0.33) | 0.04(-0.49,0.57) | 1.36(-1.21,3.92) |
| Micronutrients or iron alone | Any STH deworming combination with praziquantel | 0.01(-0.35,0.37) | 0.03(-0.47,0.53) | 0.22(-2.23,2.67) |
| Any STH deworming with micronutrients or iron | Any STH deworming combination with praziquantel with iron or micronutrients | 0.00(-0.40,0.40) | 0.05(-0.52,0.61) | -0.04(-2.93,2.84) |
| Micronutrients or iron alone | Any STH deworming combination with praziquantel with iron or micronutrients | 0.04(-0.35,0.43) | 0.04(-0.50,0.58) | -1.18(-3.93,1.56) |
| Micronutrients or iron alone | Any STH deworming with micronutrients or iron | 0.04(-0.14,0.22) | -0.01(-0.33,0.31) | -1.14(-2.74,0.46) |

**Hookworm – mild infection**

| treatment | comparison | weight | height | haemoglobin |
| --- | --- | --- | --- | --- |
| STH deworming with any drug | Placebo or control | 0.05(-0.12,0.23) | 0.20(-0.16,0.55) | -0.04(-1.84,1.77) |
| Any STH deworming combination with praziquantel | Placebo or control | 0.03(-0.22,0.27) | -0.12(-0.49,0.24) | 2.06(-0.34,4.46) |
| Any STH deworming combination with praziquantel with iron or micronutrients | Placebo or control | -0.12(-0.44,0.20) | 0.02(-0.44,0.47) | 2.75(-0.01,5.51) |
| Any STH deworming with micronutrients or iron | Placebo or control | 0.10(-0.19,0.40) | 0.15(-0.38,0.68) | 1.43(-1.42,4.28) |
| Micronutrients or iron alone | Placebo or control | -0.05(-0.30,0.20) | -0.03(-0.42,0.35) | 1.76(-0.56,4.08) |
| Any STH deworming combination with praziquantel | STH deworming with any drug | -0.03(-0.30,0.25) | -0.32(-0.74,0.10) | 2.10(-0.57,4.76) |
| Any STH deworming combination with praziquantel with iron or micronutrients | STH deworming with any drug | -0.17(-0.53,0.19) | -0.18(-0.71,0.35) | 2.78(-0.36,5.93) |
| Any STH deworming with micronutrients or iron | STH deworming with any drug | 0.05(-0.26,0.36) | -0.05(-0.58,0.48) | 1.46(-1.55,4.48) |
| Micronutrients or iron alone | STH deworming with any drug | -0.10(-0.39,0.19) | -0.23(-0.63,0.17) | 1.80(-0.69,4.29) |
| Any STH deworming combination with praziquantel with iron or micronutrients | Any STH deworming combination with praziquantel | -0.15(-0.47,0.18) | 0.14(-0.32,0.60) | 0.69(-2.08,3.46) |
| Any STH deworming with micronutrients or iron | Any STH deworming combination with praziquantel | 0.07(-0.28,0.43) | 0.27(-0.32,0.87) | -0.63(-4.00,2.74) |
| Micronutrients or iron alone | Any STH deworming combination with praziquantel | -0.08(-0.37,0.22) | 0.09(-0.32,0.49) | -0.30(-2.77,2.17) |
| Any STH deworming with micronutrients or iron | Any STH deworming combination with praziquantel with iron or micronutrients | 0.22(-0.19,0.63) | 0.13(-0.55,0.82) | -1.32(-4.83,2.19) |
| Micronutrients or iron alone | Any STH deworming combination with praziquantel with iron or micronutrients | 0.07(-0.23,0.37) | -0.05(-0.52,0.42) | -0.99(-3.77,1.80) |
| Micronutrients or iron alone | Any STH deworming with micronutrients or iron | -0.15(-0.49,0.19) | -0.18(-0.73,0.36) | 0.33(-2.64,3.31) |

**Hookworm – high infection**

| Treatment | comparison | weight | height | haemoglobin |
| --- | --- | --- | --- | --- |
| STH deworming with any drug | Placebo or control | 0.16(-0.13,0.46) | 0.20(-0.13,0.52) | 3.58(0.13,7.02) |
| Any STH deworming combination with praziquantel | Placebo or control | 0.05(-0.27,0.37) | 0.00(-0.43,0.43) | 2.81(0.32,5.30) |
| Any STH deworming combination with praziquantel with iron or micronutrients | Placebo or control | 0.23(-0.51,0.98) | -0.16(-1.01,0.68) | 4.63(-0.30,9.56) |
| Any STH deworming with micronutrients or iron | Placebo or control | 0.09(-0.55,0.73) | -0.12(-1.02,0.77) | 5.46(-0.21,11.14) |
| Micronutrients or iron alone | Placebo or control | -0.04(-0.80,0.71) | -0.36(-1.35,0.63) | 2.05(-3.86,7.96) |
| Any STH deworming combination with praziquantel | STH deworming with any drug | -0.11(-0.51,0.29) | -0.20(-0.71,0.32) | -0.77(-4.59,3.05) |
| Any STH deworming combination with praziquantel with iron or micronutrients | STH deworming with any drug | 0.07(-0.69,0.84) | -0.36(-1.27,0.55) | 1.06(-4.76,6.87) |
| Any STH deworming with micronutrients or iron | STH deworming with any drug | -0.08(-0.77,0.62) | -0.32(-1.25,0.61) | 1.89(-4.37,8.14) |
| Micronutrients or iron alone | STH deworming with any drug | -0.20(-1.00,0.59) | -0.55(-1.56,0.45) | -1.52(-8.00,4.95) |
| Any STH deworming combination with praziquantel with iron or micronutrients | Any STH deworming combination with praziquantel | 0.19(-0.53,0.90) | -0.16(-1.03,0.70) | 1.82(-3.18,6.83) |
| Any STH deworming with micronutrients or iron | Any STH deworming combination with praziquantel | 0.04(-0.67,0.75) | -0.12(-1.08,0.84) | 2.66(-3.42,8.74) |
| Micronutrients or iron alone | Any STH deworming combination with praziquantel | -0.09(-0.84,0.66) | -0.36(-1.39,0.67) | -0.76(-6.70,5.19) |
| Any STH deworming with micronutrients or iron | Any STH deworming combination with praziquantel with iron or micronutrients | -0.15(-1.22,0.93) | 0.04(-1.20,1.28) | 0.83(-6.88,8.54) |
| Micronutrients or iron alone | Any STH deworming combination with praziquantel with iron or micronutrients | -0.28(-1.28,0.73) | -0.19(-1.44,1.05) | -2.58(-9.34,4.18) |
| Micronutrients or iron alone | Any STH deworming with micronutrients or iron | -0.13(-1.01,0.75) | -0.23(-1.51,1.05) | -3.41(-11.23,4.41) |

**Effect Modifier**

***T. Trichiura*– no infection**

| treatment | comparison | weight | height | haemoglobin |
| --- | --- | --- | --- | --- |
| STH deworming with any drug | Placebo or control | -0.01(-0.12,0.10) | 0.02(-0.18,0.22) | 0.17(-1.01,1.35) |
| Any STH deworming combination with praziquantel | Placebo or control | 0.03(-0.20,0.25) | -0.26(-0.62,0.11) | 1.15(-0.68,2.98) |
| Any STH deworming combination with praziquantel with iron or micronutrients | Placebo or control | 0.09(-0.19,0.36) | -0.08(-0.48,0.33) | 1.27(-0.82,3.36) |
| Any STH deworming with micronutrients or iron | Placebo or control | -0.04(-0.22,0.13) | -0.12(-0.44,0.20) | 1.85(0.23,3.46) |
| Micronutrients or iron alone | Placebo or control | -0.09(-0.26,0.09) | -0.17(-0.47,0.13) | 0.37(-1.11,1.85) |
| Any STH deworming combination with praziquantel | STH deworming with any drug | 0.04(-0.22,0.29) | -0.28(-0.68,0.12) | 0.98(-1.08,3.03) |
| Any STH deworming combination with praziquantel with iron or micronutrients | STH deworming with any drug | 0.10(-0.19,0.38) | -0.10(-0.54,0.34) | 1.10(-1.18,3.37) |
| Any STH deworming with micronutrients or iron | STH deworming with any drug | -0.03(-0.22,0.16) | -0.14(-0.48,0.20) | 1.67(-0.11,3.46) |
| Micronutrients or iron alone | STH deworming with any drug | -0.08(-0.27,0.12) | -0.19(-0.52,0.13) | 0.19(-1.57,1.96) |
| Any STH deworming combination with praziquantel with iron or micronutrients | Any STH deworming combination with praziquantel | 0.06(-0.21,0.33) | 0.18(-0.25,0.61) | 0.12(-2.13,2.37) |
| Any STH deworming with micronutrients or iron | Any STH deworming combination with praziquantel | -0.07(-0.35,0.21) | 0.14(-0.33,0.60) | 0.70(-1.71,3.10) |
| Micronutrients or iron alone | Any STH deworming combination with praziquantel | -0.11(-0.35,0.13) | 0.09(-0.32,0.49) | -0.78(-2.78,1.22) |
| Any STH deworming with micronutrients or iron | Any STH deworming combination with praziquantel with iron or micronutrients | -0.13(-0.44,0.18) | -0.05(-0.55,0.46) | 0.58(-1.98,3.14) |
| Micronutrients or iron alone | Any STH deworming combination with praziquantel with iron or micronutrients | -0.17(-0.45,0.10) | -0.10(-0.52,0.33) | -0.90(-3.12,1.32) |
| Micronutrients or iron alone | Any STH deworming with micronutrients or iron | -0.04(-0.26,0.18) | -0.05(-0.43,0.32) | -1.48(-3.42,0.46) |

***T. Trichiura*– mild infection**

| treatment | comparison | weight | height | haemoglobin |
| --- | --- | --- | --- | --- |
| STH deworming with any drug | Placebo or control | 0.04(-0.11,0.20) | 0.30(-0.04,0.64) | 0.24(-1.23,1.71) |
| Any STH deworming combination with praziquantel | Placebo or control | 0.00(-0.26,0.26) | 0.18(-0.26,0.62) | 1.97(-0.12,4.06) |
| Any STH deworming combination with praziquantel with iron or micronutrients | Placebo or control | -0.33(-0.74,0.08) | -0.21(-0.83,0.41) | 4.23(1.20,7.25) |
| Any STH deworming with micronutrients or iron | Placebo or control | 0.06(-0.17,0.30) | 0.09(-0.34,0.53) | 2.05(0.00,4.10) |
| Micronutrients or iron alone | Placebo or control | 0.11(-0.12,0.35) | 0.08(-0.33,0.49) | 2.10(-0.10,4.31) |
| Any STH deworming combination with praziquantel | STH deworming with any drug | -0.05(-0.33,0.23) | -0.12(-0.58,0.34) | 1.73(-0.57,4.04) |
| Any STH deworming combination with praziquantel with iron or micronutrients | STH deworming with any drug | -0.38(-0.83,0.08) | -0.51(-1.17,0.14) | 3.99(0.46,7.52) |
| Any STH deworming with micronutrients or iron | STH deworming with any drug | 0.02(-0.22,0.26) | -0.21(-0.65,0.23) | 1.81(-0.23,3.85) |
| Micronutrients or iron alone | STH deworming with any drug | 0.07(-0.18,0.31) | -0.22(-0.62,0.19) | 1.87(-0.15,3.88) |
| Any STH deworming combination with praziquantel with iron or micronutrients | Any STH deworming combination with praziquantel | -0.33(-0.79,0.13) | -0.39(-1.03,0.24) | 2.26(-1.00,5.51) |
| Any STH deworming with micronutrients or iron | Any STH deworming combination with praziquantel | 0.07(-0.27,0.41) | -0.09(-0.64,0.47) | 0.08(-2.59,2.74) |
| Micronutrients or iron alone | Any STH deworming combination with praziquantel | 0.11(-0.20,0.43) | -0.10(-0.58,0.39) | 0.13(-2.33,2.60) |
| Any STH deworming with micronutrients or iron | Any STH deworming combination with praziquantel with iron or micronutrients | 0.40(-0.08,0.87) | 0.31(-0.42,1.04) | -2.18(-5.87,1.51) |
| Micronutrients or iron alone | Any STH deworming combination with praziquantel with iron or micronutrients | 0.44(0.03,0.86) | 0.30(-0.37,0.96) | -2.12(-5.86,1.61) |
| Micronutrients or iron alone | Any STH deworming with micronutrients or iron | 0.05(-0.22,0.31) | -0.01(-0.46,0.44) | 0.05(-2.15,2.26) |

***T. Trichiura*– high infection**

| treatment | comparison | weight | height | haemoglobin |
| --- | --- | --- | --- | --- |
| STH deworming with any drug | Placebo or control | 0.17(-0.06,0.41) | 0.07(-0.20,0.34) | 1.33(-1.14,3.81) |
| Any STH deworming combination with praziquantel | Placebo or control | 0.15(-0.24,0.54) | -0.11(-0.64,0.43) | 3.05(-0.48,6.59) |
| Any STH deworming combination with praziquantel with iron or micronutrients | Placebo or control | 0.23(-0.66,1.13) | 0.27(-0.95,1.50) | 6.76(-1.27,14.80) |
| Any STH deworming with micronutrients or iron | Placebo or control | 0.09(-0.30,0.48) | -0.06(-0.57,0.46) | 3.57(0.12,7.02) |
| Micronutrients or iron alone | Placebo or control | -0.04(-0.46,0.39) | -0.21(-0.79,0.38) | 3.12(-0.67,6.91) |
| Any STH deworming combination with praziquantel | STH deworming with any drug | -0.02(-0.45,0.41) | -0.18(-0.75,0.40) | 1.72(-2.42,5.86) |
| Any STH deworming combination with praziquantel with iron or micronutrients | STH deworming with any drug | 0.06(-0.85,0.98) | 0.21(-1.04,1.45) | 5.43(-2.89,13.75) |
| Any STH deworming with micronutrients or iron | STH deworming with any drug | -0.08(-0.48,0.31) | -0.12(-0.67,0.42) | 2.24(-1.27,5.75) |
| Micronutrients or iron alone | STH deworming with any drug | -0.21(-0.64,0.22) | -0.27(-0.87,0.33) | 1.79(-2.10,5.67) |
| Any STH deworming combination with praziquantel with iron or micronutrients | Any STH deworming combination with praziquantel | 0.08(-0.85,1.02) | 0.38(-0.84,1.60) | 3.71(-4.26,11.68) |
| Any STH deworming with micronutrients or iron | Any STH deworming combination with praziquantel | -0.06(-0.59,0.47) | 0.05(-0.67,0.77) | 0.52(-4.45,5.49) |
| Micronutrients or iron alone | Any STH deworming combination with praziquantel | -0.19(-0.75,0.37) | -0.10(-0.86,0.66) | 0.07(-5.01,5.14) |
| Any STH deworming with micronutrients or iron | Any STH deworming combination with praziquantel with iron or micronutrients | -0.14(-1.11,0.83) | -0.33(-1.63,0.97) | -3.19(-11.92,5.54) |
| Micronutrients or iron alone | Any STH deworming combination with praziquantel with iron or micronutrients | -0.27(-1.25,0.71) | -0.48(-1.78,0.82) | -3.64(-12.45,5.16) |
| Micronutrients or iron alone | Any STH deworming with micronutrients or iron | -0.13(-0.58,0.32) | -0.15(-0.78,0.48) | -0.45(-4.40,3.49) |

**Effect modifier**

**Anyworm – no infection**

| treatment | comparison | weight | height | haemoglobin |
| --- | --- | --- | --- | --- |
| STH deworming with any drug | Placebo or control | 0.02(-0.10,0.15) | 0.06(-0.16,0.29) | 0.21(-1.08,1.51) |
| Any STH deworming combination with praziquantel | Placebo or control | 0.07(-0.44,0.57) | -0.27(-0.94,0.40) | 0.78(-2.50,4.07) |
| Any STH deworming combination with praziquantel with iron or micronutrients | Placebo or control | 0.10(-0.38,0.58) | 0.01(-0.68,0.71) | 1.48(-1.96,4.91) |
| Any STH deworming with micronutrients or iron | Placebo or control | 0.06(-0.16,0.27) | -0.13(-0.53,0.26) | 2.75(0.68,4.82) |
| Micronutrients or iron alone | Placebo or control | 0.09(-0.16,0.34) | -0.14(-0.58,0.30) | 0.24(-1.94,2.42) |
| Any STH deworming combination with praziquantel | STH deworming with any drug | 0.04(-0.49,0.58) | -0.34(-1.03,0.36) | 0.57(-2.78,3.92) |
| Any STH deworming combination with praziquantel with iron or micronutrients | STH deworming with any drug | 0.08(-0.43,0.58) | -0.05(-0.76,0.66) | 1.26(-2.21,4.74) |
| Any STH deworming with micronutrients or iron | STH deworming with any drug | 0.04(-0.20,0.27) | -0.19(-0.62,0.23) | 2.54(0.23,4.84) |
| Micronutrients or iron alone | STH deworming with any drug | 0.07(-0.20,0.33) | -0.20(-0.66,0.26) | 0.02(-2.34,2.39) |
| Any STH deworming combination with praziquantel with iron or micronutrients | Any STH deworming combination with praziquantel | 0.03(-0.40,0.47) | 0.29(-0.49,1.06) | 0.69(-2.98,4.36) |
| Any STH deworming with micronutrients or iron | Any STH deworming combination with praziquantel | -0.01(-0.53,0.51) | 0.14(-0.61,0.89) | 1.97(-1.78,5.72) |
| Micronutrients or iron alone | Any STH deworming combination with praziquantel | 0.02(-0.47,0.51) | 0.13(-0.55,0.82) | -0.55(-3.94,2.84) |
| Any STH deworming with micronutrients or iron | Any STH deworming combination with praziquantel with iron or micronutrients | -0.04(-0.55,0.47) | -0.15(-0.92,0.63) | 1.27(-2.76,5.31) |
| Micronutrients or iron alone | Any STH deworming combination with praziquantel with iron or micronutrients | -0.01(-0.48,0.46) | -0.15(-0.86,0.56) | -1.24(-4.66,2.18) |
| Micronutrients or iron alone | Any STH deworming with micronutrients or iron | 0.03(-0.26,0.32) | -0.01(-0.52,0.50) | -2.51(-5.25,0.23) |

**Anyworm – lighter intensity infection**

| treatment | comparison | weight | height | haemoglobin |
| --- | --- | --- | --- | --- |
| STH deworming with any drug | Placebo or control | 0.01(-0.14,0.15) | 0.16(-0.13,0.44) | 0.63(-0.63,1.89) |
| Any STH deworming combination with praziquantel | Placebo or control | 0.04(-0.17,0.24) | -0.04(-0.36,0.29) | 1.66(-0.06,3.39) |
| Any STH deworming combination with praziquantel with iron or micronutrients | Placebo or control | -0.06(-0.34,0.22) | -0.10(-0.51,0.30) | 2.28(0.22,4.34) |
| Any STH deworming with micronutrients or iron | Placebo or control | -0.10(-0.31,0.11) | -0.01(-0.36,0.34) | 1.78(0.14,3.42) |
| Micronutrients or iron alone | Placebo or control | -0.08(-0.27,0.11) | -0.07(-0.38,0.25) | 1.77(0.05,3.49) |
| Any STH deworming combination with praziquantel | STH deworming with any drug | 0.03(-0.20,0.26) | -0.19(-0.56,0.18) | 1.04(-0.85,2.92) |
| Any STH deworming combination with praziquantel with iron or micronutrients | STH deworming with any drug | -0.07(-0.38,0.24) | -0.26(-0.73,0.21) | 1.65(-0.67,3.97) |
| Any STH deworming with micronutrients or iron | STH deworming with any drug | -0.11(-0.32,0.11) | -0.17(-0.55,0.21) | 1.15(-0.63,2.93) |
| Micronutrients or iron alone | STH deworming with any drug | -0.09(-0.30,0.12) | -0.23(-0.56,0.11) | 1.14(-0.60,2.89) |
| Any STH deworming combination with praziquantel with iron or micronutrients | Any STH deworming combination with praziquantel | -0.10(-0.37,0.17) | -0.06(-0.48,0.35) | 0.62(-1.60,2.84) |
| Any STH deworming with micronutrients or iron | Any STH deworming combination with praziquantel | -0.14(-0.41,0.14) | 0.03(-0.42,0.47) | 0.12(-2.16,2.39) |
| Micronutrients or iron alone | Any STH deworming combination with praziquantel | -0.12(-0.36,0.12) | -0.03(-0.40,0.34) | 0.11(-1.75,1.97) |
| Any STH deworming with micronutrients or iron | Any STH deworming combination with praziquantel with iron or micronutrients | -0.04(-0.38,0.30) | 0.09(-0.43,0.61) | -0.50(-2.95,1.95) |
| Micronutrients or iron alone | Any STH deworming combination with praziquantel with iron or micronutrients | -0.02(-0.30,0.26) | 0.03(-0.41,0.48) | -0.51(-2.77,1.75) |
| Micronutrients or iron alone | Any STH deworming with micronutrients or iron | 0.02(-0.22,0.26) | -0.06(-0.43,0.32) | -0.01(-2.00,1.98) |

**Anyworm – high infection**

| treatment | comparison | weight | height | haemoglobin |
| --- | --- | --- | --- | --- |
| STH deworming with any drug | Placebo or control | 0.12(-0.08,0.32) | 0.02(-0.21,0.26) | -0.55(-2.89,1.80) |
| Any STH deworming combination with praziquantel | Placebo or control | -0.05(-0.41,0.32) | -0.06(-0.50,0.38) | 3.08(0.12,6.05) |
| Any STH deworming combination with praziquantel with iron or micronutrients | Placebo or control | -0.28(-1.12,0.55) | -0.12(-1.11,0.88) | 3.63(0.02,7.25) |
| Any STH deworming with micronutrients or iron | Placebo or control | 0.05(-0.25,0.36) | -0.05(-0.41,0.31) | 9.21(2.89,15.53) |
| Micronutrients or iron alone | Placebo or control | 0.07(-0.30,0.44) | -0.14(-0.59,0.31) | 9.76(3.15,16.37) |
| Any STH deworming combination with praziquantel | STH deworming with any drug | -0.16(-0.58,0.25) | -0.08(-0.56,0.40) | 6.13(-0.51,12.76) |
| Any STH deworming combination with praziquantel with iron or micronutrients | STH deworming with any drug | -0.40(-1.24,0.44) | -0.14(-1.14,0.86) | 2.43(-0.24,5.10) |
| Any STH deworming with micronutrients or iron | STH deworming with any drug | -0.06(-0.39,0.26) | -0.07(-0.46,0.32) | 2.98(0.09,5.87) |
| Micronutrients or iron alone | STH deworming with any drug | -0.05(-0.42,0.33) | -0.16(-0.63,0.31) | -0.65(-4.62,3.32) |
| Any STH deworming combination with praziquantel with iron or micronutrients | Any STH deworming combination with praziquantel | -0.24(-1.12,0.65) | -0.06(-1.07,0.95) | -6.78(-13.60,0.04) |
| Any STH deworming with micronutrients or iron | Any STH deworming combination with praziquantel | 0.10(-0.37,0.57) | 0.01(-0.55,0.57) | 0.83(-2.26,3.92) |
| Micronutrients or iron alone | Any STH deworming combination with praziquantel | 0.12(-0.41,0.64) | -0.08(-0.69,0.53) | 1.38(-1.82,4.58) |
| Any STH deworming with micronutrients or iron | Any STH deworming combination with praziquantel with iron or micronutrients | 0.34(-0.60,1.28) | 0.07(-0.99,1.13) | -2.25(-6.42,1.92) |
| Micronutrients or iron alone | Any STH deworming combination with praziquantel with iron or micronutrients | 0.35(-0.59,1.29) | -0.02(-1.09,1.04) | -8.38(-15.13,-1.63) |
| Micronutrients or iron alone | Any STH deworming with micronutrients or iron | 0.02(-0.33,0.36) | -0.09(-0.56,0.38) | -1.60(-4.74,1.54) |

**Effect modifier**

**Anaemic**

| treatment | comparison | weight | height | haemoglobin |
| --- | --- | --- | --- | --- |
| STH deworming with any drug | Placebo or control | -0.01(-0.12,0.11) | 0.08(-0.16,0.32) | 0.66(-0.67,1.98) |
| Any STH deworming combination with praziquantel | Placebo or control | 0.07(-0.12,0.27) | -0.15(-0.48,0.19) | 2.66(0.88,4.45) |
| Any STH deworming combination with praziquantel with iron or micronutrients | Placebo or control | -0.05(-0.36,0.27) | -0.05(-0.51,0.41) | 3.57(1.03,6.12) |
| Any STH deworming with micronutrients or iron | Placebo or control | -0.01(-0.19,0.17) | -0.07(-0.39,0.25) | 2.54(0.82,4.25) |
| Micronutrients or iron alone | Placebo or control | -0.04(-0.24,0.15) | -0.15(-0.46,0.16) | 1.80(0.03,3.57) |
| Any STH deworming combination with praziquantel | STH deworming with any drug | 0.08(-0.13,0.30) | -0.23(-0.58,0.13) | 2.01(-0.14,4.15) |
| Any STH deworming combination with praziquantel with iron or micronutrients | STH deworming with any drug | -0.04(-0.36,0.29) | -0.13(-0.61,0.34) | 2.92(0.21,5.63) |
| Any STH deworming with micronutrients or iron | STH deworming with any drug | 0.00(-0.19,0.19) | -0.16(-0.50,0.18) | 1.88(0.07,3.69) |
| Micronutrients or iron alone | STH deworming with any drug | -0.03(-0.23,0.16) | -0.23(-0.55,0.09) | 1.15(-0.76,3.06) |
| Any STH deworming combination with praziquantel with iron or micronutrients | Any STH deworming combination with praziquantel | -0.12(-0.44,0.20) | 0.09(-0.36,0.55) | 0.91(-1.82,3.64) |
| Any STH deworming with micronutrients or iron | Any STH deworming combination with praziquantel | -0.08(-0.33,0.17) | 0.07(-0.35,0.50) | -0.13(-2.56,2.31) |
| Micronutrients or iron alone | Any STH deworming combination with praziquantel | -0.12(-0.37,0.13) | 0.00(-0.40,0.39) | -0.86(-3.15,1.43) |
| Any STH deworming with micronutrients or iron | Any STH deworming combination with praziquantel with iron or micronutrients | 0.04(-0.30,0.38) | -0.02(-0.54,0.49) | -1.04(-3.94,1.86) |
| Micronutrients or iron alone | Any STH deworming combination with praziquantel with iron or micronutrients | 0.00(-0.30,0.31) | -0.10(-0.57,0.37) | -1.77(-4.67,1.13) |
| Micronutrients or iron alone | Any STH deworming with micronutrients or iron | -0.03(-0.24,0.17) | -0.08(-0.43,0.28) | -0.73(-2.79,1.33) |

**Nonanaemic**

| treatment | comparison | weight | height | haemoglobin |
| --- | --- | --- | --- | --- |
| STH deworming with any drug | Placebo or control | 0.06(-0.07,0.20) | 0.09(-0.15,0.34) | -0.18(-1.31,0.95) |
| Any STH deworming combination with praziquantel | Placebo or control | -0.02(-0.26,0.23) | 0.02(-0.36,0.40) | 0.96(-0.93,2.86) |
| Any STH deworming combination with praziquantel with iron or micronutrients | Placebo or control | -0.05(-0.36,0.27) | -0.07(-0.53,0.39) | 1.14(-0.89,3.18) |
| Any STH deworming with micronutrients or iron | Placebo or control | 0.00(-0.19,0.19) | 0.00(-0.32,0.33) | 2.07(-0.08,4.23) |
| Micronutrients or iron alone | Placebo or control | 0.00(-0.21,0.22) | -0.03(-0.34,0.29) | 2.25(-0.14,4.64) |
| Any STH deworming combination with praziquantel | STH deworming with any drug | -0.08(-0.35,0.19) | -0.07(-0.50,0.35) | 1.11(-1.16,3.37) |
| Any STH deworming combination with praziquantel with iron or micronutrients | STH deworming with any drug | -0.11(-0.44,0.23) | -0.16(-0.66,0.34) | 1.67(0.11,3.23) |
| Any STH deworming with micronutrients or iron | STH deworming with any drug | -0.06(-0.27,0.15) | -0.09(-0.46,0.28) | 1.85(0.15,3.54) |
| Micronutrients or iron alone | STH deworming with any drug | -0.06(-0.29,0.17) | -0.12(-0.48,0.25) | 0.71(-1.62,3.03) |
| Any STH deworming combination with praziquantel with iron or micronutrients | Any STH deworming combination with praziquantel | -0.03(-0.35,0.29) | -0.09(-0.58,0.40) | -0.40(-3.01,2.20) |
| Any STH deworming with micronutrients or iron | Any STH deworming combination with praziquantel | 0.02(-0.28,0.32) | -0.01(-0.50,0.47) | 0.92(-0.65,2.49) |
| Micronutrients or iron alone | Any STH deworming combination with praziquantel | 0.02(-0.27,0.30) | -0.04(-0.47,0.38) | 1.10(-0.63,2.83) |
| Any STH deworming with micronutrients or iron | Any STH deworming combination with praziquantel with iron or micronutrients | 0.05(-0.31,0.40) | 0.07(-0.47,0.62) | -0.05(-2.10,2.01) |
| Micronutrients or iron alone | Any STH deworming combination with praziquantel with iron or micronutrients | 0.05(-0.26,0.36) | 0.04(-0.45,0.53) | -1.15(-3.45,1.14) |
| Micronutrients or iron alone | Any STH deworming with micronutrients or iron | 0.00(-0.24,0.24) | -0.03(-0.42,0.36) | -0.75(-2.56,1.06) |

Additional tables 16: Effect modifier analyses for cognition

BMI for age

| **Study** | **Treatment** | **Comparator** | **Cognition outcome** | **Sub-group** | **EE** | **95% LCI** | **95% UCI** |
| --- | --- | --- | --- | --- | --- | --- | --- |
| **Ebenezer** | Mebendazole + iron | Placebo | Single digit attention score | BMI ≤ -2 | -0.14 | -1.16 | 0.88 |
| **Ebenezer** | Mebendazole + iron | Placebo | Single digit attention score | BMI > -2 | 0.05 | -1.28 | 1.38 |
| **Ebenezer** | Mebendazole + iron | Placebo | Double digit attention score | BMI ≤ -2 | -0.06 | -1.05 | 0.93 |
| **Ebenezer** | Mebendazole + iron | Placebo | Double digit attention score | BMI > -2 | -0.22 | -1.51 | 1.07 |
| **Ebenezer** | Mebendazole + iron | Placebo | Math score | BMI ≤ -2 | 2.40 | -0.23 | 5.02 |
| **Ebenezer** | Mebendazole + iron | Placebo | Math score | BMI > -2 | 1.20 | -2.54 | 4.95 |
| **Ebenezer** | Mebendazole + iron | Placebo | Tamil language score | BMI ≤ -2 | -0.83 | -5.45 | 3.80 |
| **Ebenezer** | Mebendazole + iron | Placebo | Tamil language score | BMI > -2 | -1.48 | -4.08 | 1.12 |
| **Liu** | Albendazole | Placebo | Processing speed index | BMI ≤ -2 | 0.91 | -0.36 | 2.18 |
| **Liu** | Albendazole | Placebo | Processing speed index | BMI > -2 | 0.80 | -3.12 | 4.71 |
| **Liu** | Albendazole | Placebo | Working memory index | BMI ≤ -2 | 0.63 | -0.34 | 1.60 |
| **Liu** | Albendazole | Placebo | Working memory index | BMI > -2 | -0.93 | -4.52 | 2.67 |
| **Liu** | Albendazole | Placebo | TIMSS z-score | BMI ≤ -2 | -0.04 | -0.12 | 0.04 |
| **Liu** | Albendazole | Placebo | TIMSS z-score | BMI > -2 | 0.07 | -0.23 | 0.37 |
| **Nga** | Albendazole | Placebo | Raven score | BMI ≤ -2 | 0.21 | -0.95 | 1.37 |
| **Nga** | Albendazole | Placebo | Raven score | BMI > -2 | -1.96 | -5.53 | 1.62 |
| **Nga** | Albendazole | Placebo | Digit forward | BMI ≤ -2 | 0.15 | -0.19 | 0.48 |
| **Nga** | Albendazole | Placebo | Digit forward | BMI > -2 | 0.74 | -0.19 | 1.67 |
| **Nga** | Albendazole | Placebo | Digit back | BMI ≤ -2 | 0.12 | -0.18 | 0.42 |
| **Nga** | Albendazole | Placebo | Digit back | BMI > -2 | 0.24 | -0.57 | 1.04 |
| **Nga** | Albendazole | Placebo | Block score | BMI ≤ -2 | 0.94 | -0.91 | 2.78 |
| **Nga** | Albendazole | Placebo | Block score | BMI > -2 | 0.82 | -4.89 | 6.54 |
| **Nga** | Albendazole | Placebo | Code score | BMI ≤ -2 | -0.04 | -2.38 | 2.31 |
| **Nga** | Albendazole | Placebo | Code score | BMI > -2 | 0.93 | -5.50 | 7.37 |
| **Nga** | Albendazole + micronutrients | Placebo | Raven score | BMI ≤ -2 | 0.42 | -0.75 | 1.60 |
| **Nga** | Albendazole + micronutrients | Placebo | Raven score | BMI > -2 | -2.52 | -6.07 | 1.03 |
| **Nga** | Albendazole + micronutrients | Placebo | Digit forward | BMI ≤ -2 | **0.44** | **0.10** | **0.79** |
| **Nga** | Albendazole + micronutrients | Placebo | Digit forward | BMI > -2 | -0.13 | -1.07 | 0.81 |
| **Nga** | Albendazole + micronutrients | Placebo | Digit back | BMI ≤ -2 | -0.14 | -0.45 | 0.17 |
| **Nga** | Albendazole + micronutrients | Placebo | Digit back | BMI > -2 | 0.66 | -0.15 | 1.47 |
| **Nga** | Albendazole + micronutrients | Placebo | Block score | BMI ≤ -2 | -0.55 | -2.51 | 1.42 |
| **Nga** | Albendazole + micronutrients | Placebo | Block score | BMI > -2 | -2.74 | -8.32 | 2.85 |
| **Nga** | Albendazole + micronutrients | Placebo | Code score | BMI ≤ -2 | 1.26 | -1.07 | 3.59 |
| **Nga** | Albendazole + micronutrients | Placebo | Code score | BMI > -2 | 0.85 | -5.56 | 7.26 |
| **Nga** | Micronutrients | Placebo | Raven score | BMI ≤ -2 | 0.92 | -0.19 | 2.03 |
| **Nga** | Micronutrients | Placebo | Raven score | BMI > -2 | -0.01 | -3.47 | 3.45 |
| **Nga** | Micronutrients | Placebo | Digit forward | BMI ≤ -2 | **0.64** | **0.31** | **0.97** |
| **Nga** | Micronutrients | Placebo | Digit forward | BMI > -2 | 0.05 | -0.87 | 0.97 |
| **Nga** | Micronutrients | Placebo | Digit back | BMI ≤ -2 | 0.02 | -0.28 | 0.32 |
| **Nga** | Micronutrients | Placebo | Digit back | BMI > -2 | -0.07 | -0.86 | 0.72 |
| **Nga** | Micronutrients | Placebo | Block score | BMI ≤ -2 | -0.49 | -2.36 | 1.38 |
| **Nga** | Micronutrients | Placebo | Block score | BMI > -2 | -1.59 | -7.16 | 3.99 |
| **Nga** | Micronutrients | Placebo | Code score | BMI ≤ -2 | 0.86 | -1.55 | 3.27 |
| **Nga** | Micronutrients | Placebo | Code score | BMI > -2 | 5.70 | -0.61 | 12.01 |
| Rohner | 5 = Albendazole + Praziquantel + Iron | 1 = Placebo | Coding total | BMI ≤ -2 | -2.27 | -5.07 | 0.54 |
| Rohner | 5 = Albendazole + Praziquantel + Iron | 1 = Placebo | Coding total | BMI > -2 | NA | NA | NA |
| Rohner | 6 = Albendazole + Praziquantel | 1 = Placebo | Coding total | BMI ≤ -2 | 0.44 | -2.39 | 3.28 |
| Rohner | 6 = Albendazole + Praziquantel | 1 = Placebo | Coding total | BMI > -2 | NA | NA | NA |
| Rohner | 7 = Iron fortified food/bev | 1 = Placebo | Coding total | BMI ≤ -2 | -0.14 | -3.07 | 2.80 |
| Rohner | 7 = Iron fortified food/bev | 1 = Placebo | Coding total | BMI > -2 | NA | NA | NA |
| Rohner | 5 = Albendazole + Praziquantel + Iron | 1 = Placebo | Raven score | BMI ≤ -2 | -1.14 | -3.10 | 0.83 |
| Rohner | 5 = Albendazole + Praziquantel + Iron | 1 = Placebo | Raven score | BMI > -2 | NA | NA | NA |
| Rohner | 6 = Albendazole + Praziquantel | 1 = Placebo | Raven score | BMI ≤ -2 | 1.11 | -0.89 | 3.12 |
| Rohner | 6 = Albendazole + Praziquantel | 1 = Placebo | Raven score | BMI > -2 | NA | NA | NA |
| Rohner | 7 = Iron fortified food/bev | 1 = Placebo | Raven score | BMI ≤ -2 | -1.38 | -3.43 | 0.68 |
| Rohner | 7 = Iron fortified food/bev | 1 = Placebo | Raven score | BMI > -2 | NA | NA | NA |
| Rohner | 5 = Albendazole + Praziquantel + Iron | 1 = Placebo | Symbols total | BMI ≤ -2 | 0.08 | -1.81 | 1.96 |
| Rohner | 5 = Albendazole + Praziquantel + Iron | 1 = Placebo | Symbols total | BMI > -2 | NA | NA | NA |
| Rohner | 6 = Albendazole + Praziquantel | 1 = Placebo | Symbols total | BMI ≤ -2 | 0.77 | -1.15 | 2.69 |
| Rohner | 6 = Albendazole + Praziquantel | 1 = Placebo | Symbols total | BMI > -2 | NA | NA | NA |
| Rohner | 7 = Iron fortified food/bev | 1 = Placebo | Symbols total | BMI ≤ -2 | 0.97 | -1.03 | 2.98 |
| Rohner | 7 = Iron fortified food/bev | 1 = Placebo | Symbols total | BMI > -2 | NA | NA | NA |
| **Stoltzfus04** | Iron | Placebo | Language skills | BMI ≤ -2 | 0.36 | -0.83 | 1.55 |
| **Stoltzfus04** | Iron | Placebo | Language skills | BMI > -2 | NA | NA | NA |
| **Stoltzfus04** | Iron | Placebo | Motor skills | BMI ≤ -2 | 0.00 | -0.08 | 0.08 |
| **Stoltzfus04** | Iron | Placebo | Motor skills | BMI > -2 | NA | NA | NA |
| **Stoltzfus04** | Mebendazole (high) | Placebo | Language skills | BMI ≤ -2 | 0.32 | -0.86 | 1.51 |
| **Stoltzfus04** | Mebendazole (high) | Placebo | Language skills | BMI > -2 | NA | NA | NA |
| **Stoltzfus04** | Mebendazole (high) | Placebo | Motor skills | BMI ≤ -2 | 0.01 | -0.08 | 0.10 |
| **Stoltzfus04** | Mebendazole (high) | Placebo | Motor skills | BMI > -2 | NA | NA | NA |
| **Stoltzfus04** | Mebendazole (high) + iron | Placebo | Language skills | BMI ≤ -2 | 0.67 | -0.45 | 1.79 |
| **Stoltzfus04** | Mebendazole (high) + iron | Placebo | Language skills | BMI > -2 | NA | NA | NA |
| **Stoltzfus04** | Mebendazole (high) + iron | Placebo | Motor skills | BMI ≤ -2 | 0.01 | -0.07 | 0.10 |
| **Stoltzfus04** | Mebendazole (high) + iron | Placebo | Motor skills | BMI > -2 | NA | NA | NA |
| **Solon** | Albendazole | Placebo | Verbal ability | BMI ≤ -2 | 0.02 | -0.63 | 0.68 |
| **Solon** | Albendazole | Placebo | Verbal ability | BMI > -2 | -0.37 | -1.92 | 1.18 |
| **Solon** | Albendazole | Placebo | Quantitative ability | BMI ≤ -2 | 0.00 | -0.47 | 0.48 |
| **Solon** | Albendazole | Placebo | Quantitative ability | BMI > -2 | -0.06 | -1.24 | 1.12 |
| **Solon** | Albendazole | Placebo | Non-verbal ability | BMI ≤ -2 | 0.08 | -0.34 | 0.50 |
| **Solon** | Albendazole | Placebo | Non-verbal ability | BMI > -2 | -0.02 | -1.05 | 1.01 |
| **Solon** | Albendazole | Placebo | Total cognition score | BMI ≤ -2 | 0.11 | -0.92 | 1.14 |
| **Solon** | Albendazole | Placebo | Total cognition score | BMI > -2 | -0.44 | -2.80 | 1.92 |
| **Solon** | Albendazole + micronutrients | Placebo | Verbal ability | BMI ≤ -2 | 0.30 | -0.29 | 0.90 |
| **Solon** | Albendazole + micronutrients | Placebo | Verbal ability | BMI > -2 | 1.17 | -0.51 | 2.85 |
| **Solon** | Albendazole + micronutrients | Placebo | Quantitative ability | BMI ≤ -2 | 0.02 | -0.43 | 0.48 |
| **Solon** | Albendazole + micronutrients | Placebo | Quantitative ability | BMI > -2 | 0.10 | -1.20 | 1.41 |
| **Solon** | Albendazole + micronutrients | Placebo | Non-verbal ability | BMI ≤ -2 | -0.10 | -0.50 | 0.30 |
| **Solon** | Albendazole + micronutrients | Placebo | Non-verbal ability | BMI > -2 | -0.21 | -1.35 | 0.93 |
| **Solon** | Albendazole + micronutrients | Placebo | Total cognition score | BMI ≤ -2 | 0.23 | -0.67 | 1.13 |
| **Solon** | Albendazole + micronutrients | Placebo | Total cognition score | BMI > -2 | 1.07 | -1.52 | 3.65 |
| **Solon** | Micronutrients | Placebo | Verbal ability | BMI ≤ -2 | 0.09 | -0.50 | 0.68 |
| **Solon** | Micronutrients | Placebo | Verbal ability | BMI > -2 | 0.58 | -0.89 | 2.05 |
| **Solon** | Micronutrients | Placebo | Quantitative ability | BMI ≤ -2 | 0.16 | -0.30 | 0.62 |
| **Solon** | Micronutrients | Placebo | Quantitative ability | BMI > -2 | 0.18 | -1.08 | 1.43 |
| **Solon** | Micronutrients | Placebo | Non-verbal ability | BMI ≤ -2 | 0.02 | -0.40 | 0.45 |
| **Solon** | Micronutrients | Placebo | Non-verbal ability | BMI > -2 | -0.89 | -1.92 | 0.14 |
| **Solon** | Micronutrients | Placebo | Total cognition score | BMI ≤ -2 | 0.27 | -0.66 | 1.20 |
| **Solon** | Micronutrients | Placebo | Total cognition score | BMI > -2 | -0.13 | -2.37 | 2.11 |

**HAZ**

| **Study** | **Treatment** | **Comparator** | **Cognition outcome** | **Sub-group** | **EE** | **95% LCI** | **95% UCI** |
| --- | --- | --- | --- | --- | --- | --- | --- |
| **Ebenezer** | Mebendazole + iron | Placebo | Single digit attention score | HAZ ≤ -2 | -0.18 | -1.24 | 0.89 |
| **Ebenezer** | Mebendazole + iron | Placebo | Single digit attention score | HAZ > -2 | 0.14 | -1.26 | 1.53 |
| **Ebenezer** | Mebendazole + iron | Placebo | Double digit attention score | HAZ ≤ -2 | 0.18 | -0.82 | 1.18 |
| **Ebenezer** | Mebendazole + iron | Placebo | Double digit attention score | HAZ > -2 | -0.65 | -1.78 | 0.47 |
| **Ebenezer** | Mebendazole + iron | Placebo | Math score | HAZ ≤ -2 | 2.24 | -0.54 | 5.02 |
| **Ebenezer** | Mebendazole + iron | Placebo | Math score | HAZ > -2 | 1.82 | -2.48 | 6.13 |
| **Ebenezer** | Mebendazole + iron | Placebo | Tamil language score | HAZ ≤ -2 | -1.50 | -3.96 | 0.97 |
| **Ebenezer** | Mebendazole + iron | Placebo | Tamil language score | HAZ > -2 | -0.97 | -5.31 | 3.37 |
| **Liu** | Albendazole | Placebo | Processing speed index | HAZ ≤ -2 | 0.96 | -0.50 | 2.42 |
| **Liu** | Albendazole | Placebo | Processing speed index | HAZ > -2 | 0.97 | -0.87 | 2.81 |
| **Liu** | Albendazole | Placebo | Working memory index | HAZ ≤ -2 | 0.98 | -0.05 | 2.00 |
| **Liu** | Albendazole | Placebo | Working memory index | HAZ > -2 | -0.83 | -2.49 | 0.83 |
| **Liu** | Albendazole | Placebo | TIMSS z-score | HAZ ≤ -2 | -0.02 | -0.10 | 0.07 |
| **Liu** | Albendazole | Placebo | TIMSS z-score | HAZ > -2 | -0.07 | -0.20 | 0.06 |
| **Nga** | Albendazole | Placebo | Raven score | HAZ ≤ -2 | 0.02 | -1.20 | 1.24 |
| **Nga** | Albendazole | Placebo | Raven score | HAZ > -2 | 0.74 | -1.57 | 3.04 |
| **Nga** | Albendazole | Placebo | Digit forward | HAZ ≤ -2 | 0.25 | -0.12 | 0.61 |
| **Nga** | Albendazole | Placebo | Digit forward | HAZ > -2 | 0.33 | -0.33 | 0.99 |
| **Nga** | Albendazole | Placebo | Digit back | HAZ ≤ -2 | 0.12 | -0.21 | 0.46 |
| **Nga** | Albendazole | Placebo | Digit back | HAZ > -2 | 0.10 | -0.45 | 0.64 |
| **Nga** | Albendazole | Placebo | Block score | HAZ ≤ -2 | 0.82 | -1.20 | 2.84 |
| **Nga** | Albendazole | Placebo | Block score | HAZ > -2 | 1.23 | -2.33 | 4.79 |
| **Nga** | Albendazole | Placebo | Code score | HAZ ≤ -2 | 0.71 | -1.72 | 3.15 |
| **Nga** | Albendazole | Placebo | Code score | HAZ > -2 | -1.51 | -6.20 | 3.18 |
| **Nga** | Albendazole + micronutrients | Placebo | Raven score | HAZ ≤ -2 | 0.41 | -0.80 | 1.62 |
| **Nga** | Albendazole + micronutrients | Placebo | Raven score | HAZ > -2 | -0.12 | -2.39 | 2.16 |
| **Nga** | Albendazole + micronutrients | Placebo | Digit forward | HAZ ≤ -2 | 0.20 | -0.17 | 0.57 |
| **Nga** | Albendazole + micronutrients | Placebo | Digit forward | HAZ > -2 | **0.85** | **0.18** | **1.52** |
| **Nga** | Albendazole + micronutrients | Placebo | Digit back | HAZ ≤ -2 | -0.13 | -0.48 | 0.22 |
| **Nga** | Albendazole + micronutrients | Placebo | Digit back | HAZ > -2 | 0.09 | -0.44 | 0.63 |
| **Nga** | Albendazole + micronutrients | Placebo | Block score | HAZ ≤ -2 | -1.27 | -3.36 | 0.82 |
| **Nga** | Albendazole + micronutrients | Placebo | Block score | HAZ > -2 | 0.10 | -3.53 | 3.73 |
| **Nga** | Albendazole + micronutrients | Placebo | Code score | HAZ ≤ -2 | 1.62 | -0.80 | 4.04 |
| **Nga** | Albendazole + micronutrients | Placebo | Code score | HAZ > -2 | -0.34 | -4.94 | 4.26 |
| **Nga** | Micronutrients | Placebo | Raven score | HAZ ≤ -2 | 1.02 | -0.25 | 2.29 |
| **Nga** | Micronutrients | Placebo | Raven score | HAZ > -2 | 0.81 | -1.36 | 2.98 |
| **Nga** | Micronutrients | Placebo | Digit forward | HAZ ≤ -2 | **0.54** | **0.18** | **0.90** |
| **Nga** | Micronutrients | Placebo | Digit forward | HAZ > -2 | **0.78** | **0.14** | **1.42** |
| **Nga** | Micronutrients | Placebo | Digit back | HAZ ≤ -2 | 0.00 | -0.35 | 0.34 |
| **Nga** | Micronutrients | Placebo | Digit back | HAZ > -2 | -0.04 | -0.60 | 0.52 |
| **Nga** | Micronutrients | Placebo | Block score | HAZ ≤ -2 | -1.01 | -3.09 | 1.06 |
| **Nga** | Micronutrients | Placebo | Block score | HAZ > -2 | 0.67 | -2.78 | 4.13 |
| **Nga** | Micronutrients | Placebo | Code score | HAZ ≤ -2 | 2.03 | -0.49 | 4.55 |
| **Nga** | Micronutrients | Placebo | Code score | HAZ > -2 | -0.14 | -4.64 | 4.37 |
| Rohner | 5 = Albendazole + Praziquantel + Iron | 1 = Placebo | Coding total | HAZ = 0 | -2.97 | -6.08 | 0.13 |
| Rohner | 5 = Albendazole + Praziquantel + Iron | 1 = Placebo | Coding total | HAZ = 1 | 0.21 | -6.70 | 7.13 |
| Rohner | 6 = Albendazole + Praziquantel | 1 = Placebo | Coding total | HAZ = 0 | 1.98 | -1.38 | 5.34 |
| Rohner | 6 = Albendazole + Praziquantel | 1 = Placebo | Coding total | HAZ = 1 | -1.67 | -7.52 | 4.19 |
| Rohner | 7 = Iron fortified food/bev | 1 = Placebo | Coding total | HAZ = 0 | 1.00 | -2.17 | 4.18 |
| Rohner | 7 = Iron fortified food/bev | 1 = Placebo | Coding total | HAZ = 1 | -4.55 | -12.39 | 3.29 |
| Rohner | 5 = Albendazole + Praziquantel + Iron | 1 = Placebo | Raven score | HAZ = 0 | -1.66 | -3.89 | 0.58 |
| Rohner | 5 = Albendazole + Praziquantel + Iron | 1 = Placebo | Raven score | HAZ = 1 | -0.63 | -5.70 | 4.43 |
| Rohner | 6 = Albendazole + Praziquantel | 1 = Placebo | Raven score | HAZ = 0 | 1.26 | -1.16 | 3.67 |
| Rohner | 6 = Albendazole + Praziquantel | 1 = Placebo | Raven score | HAZ = 1 | 0.92 | -3.37 | 5.20 |
| Rohner | 7 = Iron fortified food/bev | 1 = Placebo | Raven score | HAZ = 0 | -0.80 | -3.08 | 1.48 |
| Rohner | 7 = Iron fortified food/bev | 1 = Placebo | Raven score | HAZ = 1 | -1.53 | -7.26 | 4.21 |
| Rohner | 5 = Albendazole + Praziquantel + Iron | 1 = Placebo | Symbols total | HAZ = 0 | -0.22 | -2.40 | 1.96 |
| Rohner | 5 = Albendazole + Praziquantel + Iron | 1 = Placebo | Symbols total | HAZ = 1 | 1.56 | -2.95 | 6.07 |
| Rohner | 6 = Albendazole + Praziquantel | 1 = Placebo | Symbols total | HAZ = 0 | 1.55 | -0.80 | 3.89 |
| Rohner | 6 = Albendazole + Praziquantel | 1 = Placebo | Symbols total | HAZ = 1 | 0.26 | -3.55 | 4.07 |
| Rohner | 7 = Iron fortified food/bev | 1 = Placebo | Symbols total | HAZ = 0 | -0.16 | -2.41 | 2.09 |
| Rohner | 7 = Iron fortified food/bev | 1 = Placebo | Symbols total | HAZ = 1 | 3.59 | -1.51 | 8.70 |
| Rohner | 5 = Albendazole + Praziquantel + Iron | 1 = Placebo | Target marking errors | HAZ = 0 | -0.13 | -0.30 | 0.04 |
| Rohner | 5 = Albendazole + Praziquantel + Iron | 1 = Placebo | Target marking errors | HAZ = 1 | 0.85 | -0.57 | 2.28 |
| Rohner | 6 = Albendazole + Praziquantel | 1 = Placebo | Target marking errors | HAZ = 0 | -0.12 | -0.31 | 0.06 |
| Rohner | 6 = Albendazole + Praziquantel | 1 = Placebo | Target marking errors | HAZ = 1 | 0.29 | -0.91 | 1.50 |
| Rohner | 7 = Iron fortified food/bev | 1 = Placebo | Target marking errors | HAZ = 0 | -0.06 | -0.23 | 0.12 |
| Rohner | 7 = Iron fortified food/bev | 1 = Placebo | Target marking errors | HAZ = 1 | 0.24 | -1.37 | 1.86 |
| Rohner | 5 = Albendazole + Praziquantel + Iron | 1 = Placebo | Target marking time | HAZ = 0 | -2.39 | -11.84 | 7.06 |
| Rohner | 5 = Albendazole + Praziquantel + Iron | 1 = Placebo | Target marking time | HAZ = 1 | 3.66 | -10.48 | 17.81 |
| Rohner | 6 = Albendazole + Praziquantel | 1 = Placebo | Target marking time | HAZ = 0 | -2.98 | -13.17 | 7.22 |
| Rohner | 6 = Albendazole + Praziquantel | 1 = Placebo | Target marking time | HAZ = 1 | -0.07 | -12.06 | 11.91 |
| Rohner | 7 = Iron fortified food/bev | 1 = Placebo | Target marking time | HAZ = 0 | -0.57 | -10.21 | 9.07 |
| Rohner | 7 = Iron fortified food/bev | 1 = Placebo | Target marking time | HAZ = 1 | 16.94 | 0.66 | 33.22 |
| Rohner | 5 = Albendazole + Praziquantel + Iron | 1 = Placebo | Coding total | HAZ = 0 | -2.97 | -6.08 | 0.13 |
| **Stoltzfus04** | Iron | Placebo | Language skills | HAZ ≤ -2 | 1.25 | -0.14 | 2.64 |
| **Stoltzfus04** | Iron | Placebo | Language skills | HAZ > -2 | -0.88 | -2.69 | 0.93 |
| **Stoltzfus04** | Iron | Placebo | Motor skills | HAZ ≤ -2 | -0.04 | -0.15 | 0.07 |
| **Stoltzfus04** | Iron | Placebo | Motor skills | HAZ > -2 | 0.04 | -0.08 | 0.17 |
| **Stoltzfus04** | Mebendazole (high) | Placebo | Language skills | HAZ ≤ -2 | 0.50 | -0.90 | 1.91 |
| **Stoltzfus04** | Mebendazole (high) | Placebo | Language skills | HAZ > -2 | -0.49 | -2.31 | 1.32 |
| **Stoltzfus04** | Mebendazole (high) | Placebo | Motor skills | HAZ ≤ -2 | -0.02 | -0.15 | 0.10 |
| **Stoltzfus04** | Mebendazole (high) | Placebo | Motor skills | HAZ > -2 | 0.03 | -0.10 | 0.16 |
| **Stoltzfus04** | Mebendazole (high) + iron | Placebo | Language skills | HAZ ≤ -2 | 0.72 | -0.77 | 2.21 |
| **Stoltzfus04** | Mebendazole (high) + iron | Placebo | Language skills | HAZ > -2 | 0.19 | -1.55 | 1.94 |
| **Stoltzfus04** | Mebendazole (high) + iron | Placebo | Motor skills | HAZ ≤ -2 | -0.04 | -0.17 | 0.08 |
| **Stoltzfus04** | Mebendazole (high) + iron | Placebo | Motor skills | HAZ > -2 | 0.06 | -0.07 | 0.18 |
| **Solon** | Albendazole | Placebo | Verbal ability | HAZ ≤ -2 | -0.13 | -0.86 | 0.61 |
| **Solon** | Albendazole | Placebo | Verbal ability | HAZ > -2 | 0.09 | -0.85 | 1.03 |
| **Solon** | Albendazole | Placebo | Quantitative ability | HAZ ≤ -2 | 0.06 | -0.52 | 0.64 |
| **Solon** | Albendazole | Placebo | Quantitative ability | HAZ > -2 | -0.02 | -0.72 | 0.67 |
| **Solon** | Albendazole | Placebo | Non-verbal ability | HAZ ≤ -2 | 0.09 | -0.41 | 0.58 |
| **Solon** | Albendazole | Placebo | Non-verbal ability | HAZ > -2 | 0.14 | -0.52 | 0.80 |
| **Solon** | Albendazole | Placebo | Total cognition score | HAZ ≤ -2 | 0.02 | -1.07 | 1.12 |
| **Solon** | Albendazole | Placebo | Total cognition score | HAZ > -2 | 0.20 | -1.41 | 1.82 |
| **Solon** | Albendazole + micronutrients | Placebo | Verbal ability | HAZ ≤ -2 | 0.54 | -0.15 | 1.24 |
| **Solon** | Albendazole + micronutrients | Placebo | Verbal ability | HAZ > -2 | 0.13 | -0.79 | 1.06 |
| **Solon** | Albendazole + micronutrients | Placebo | Quantitative ability | HAZ ≤ -2 | -0.04 | -0.65 | 0.57 |
| **Solon** | Albendazole + micronutrients | Placebo | Quantitative ability | HAZ > -2 | 0.21 | -0.46 | 0.88 |
| **Solon** | Albendazole + micronutrients | Placebo | Non-verbal ability | HAZ ≤ -2 | -0.37 | -0.89 | 0.14 |
| **Solon** | Albendazole + micronutrients | Placebo | Non-verbal ability | HAZ > -2 | 0.32 | -0.31 | 0.94 |
| **Solon** | Albendazole + micronutrients | Placebo | Total cognition score | HAZ ≤ -2 | 0.13 | -0.98 | 1.24 |
| **Solon** | Albendazole + micronutrients | Placebo | Total cognition score | HAZ > -2 | 0.66 | -0.75 | 2.08 |
| **Solon** | Micronutrients | Placebo | Verbal ability | HAZ ≤ -2 | 0.18 | -0.51 | 0.88 |
| **Solon** | Micronutrients | Placebo | Verbal ability | HAZ > -2 | 0.04 | -0.87 | 0.96 |
| **Solon** | Micronutrients | Placebo | Quantitative ability | HAZ ≤ -2 | 0.19 | -0.43 | 0.81 |
| **Solon** | Micronutrients | Placebo | Quantitative ability | HAZ > -2 | 0.04 | -0.61 | 0.69 |
| **Solon** | Micronutrients | Placebo | Non-verbal ability | HAZ ≤ -2 | -0.12 | -0.63 | 0.40 |
| **Solon** | Micronutrients | Placebo | Non-verbal ability | HAZ > -2 | -0.01 | -0.61 | 0.59 |
| **Solon** | Micronutrients | Placebo | Total cognition score | HAZ ≤ -2 | 0.26 | -0.91 | 1.43 |
| **Solon** | Micronutrients | Placebo | Total cognition score | HAZ > -2 | 0.07 | -1.28 | 1.42 |

**Sex**

| **Study** | **Treatment** | **Comparator** | **Cognition outcome** | **Sub-group** | **EE** | **95% LCI** | **95% UCI** |
| --- | --- | --- | --- | --- | --- | --- | --- |
| **Ebenezer** | Mebendazole + iron | Placebo | Single digit attention score | female | 0.31 | -0.86 | 1.48 |
| **Ebenezer** | Mebendazole + iron | Placebo | Single digit attention score | male | -0.28 | -1.51 | 0.95 |
| **Ebenezer** | Mebendazole + iron | Placebo | Double digit attention score | female | 0.21 | -0.75 | 1.17 |
| **Ebenezer** | Mebendazole + iron | Placebo | Double digit attention score | male | -0.34 | -1.51 | 0.84 |
| **Ebenezer** | Mebendazole + iron | Placebo | Math score | female | 2.65 | -0.57 | 5.87 |
| **Ebenezer** | Mebendazole + iron | Placebo | Math score | male | 1.68 | -1.33 | 4.70 |
| **Ebenezer** | Mebendazole + iron | Placebo | Tamil language score | female | 0.16 | -2.91 | 3.23 |
| **Ebenezer** | Mebendazole + iron | Placebo | Tamil language score | male | -2.47 | -5.33 | 0.39 |
| **Liu** | Albendazole | Placebo | Processing speed index | female | 1.35 | -0.29 | 3.00 |
| **Liu** | Albendazole | Placebo | Processing speed index | male | 0.47 | -1.13 | 2.07 |
| **Liu** | Albendazole | Placebo | Working memory index | female | 0.19 | -1.13 | 1.50 |
| **Liu** | Albendazole | Placebo | Working memory index | male | 0.79 | -0.36 | 1.94 |
| **Liu** | Albendazole | Placebo | TIMSS z-score | female | -0.01 | -0.11 | 0.09 |
| **Liu** | Albendazole | Placebo | TIMSS z-score | male | -0.05 | -0.15 | 0.06 |
| **Nga** | Albendazole | Placebo | Raven score | female | -0.65 | -2.15 | 0.85 |
| **Nga** | Albendazole | Placebo | Raven score | male | 0.81 | -0.71 | 2.33 |
| **Nga** | Albendazole | Placebo | Digit forward | female | 0.23 | -0.19 | 0.66 |
| **Nga** | Albendazole | Placebo | Digit forward | male | 0.28 | -0.22 | 0.78 |
| **Nga** | Albendazole | Placebo | Digit back | female | 0.12 | -0.26 | 0.51 |
| **Nga** | Albendazole | Placebo | Digit back | male | 0.20 | -0.21 | 0.61 |
| **Nga** | Albendazole | Placebo | Block score | female | 1.30 | -1.09 | 3.70 |
| **Nga** | Albendazole | Placebo | Block score | male | 0.87 | -1.93 | 3.68 |
| **Nga** | Albendazole | Placebo | Code score | female | -0.48 | -3.46 | 2.50 |
| **Nga** | Albendazole | Placebo | Code score | male | 0.85 | -2.38 | 4.07 |
| **Nga** | Albendazole + Micronutrients | Placebo | Raven score | female | -0.14 | -1.69 | 1.41 |
| **Nga** | Albendazole + Micronutrients | Placebo | Raven score | male | 0.23 | -1.29 | 1.75 |
| **Nga** | Albendazole + Micronutrients | Placebo | Digit forward | female | 0.39 | -0.05 | 0.84 |
| **Nga** | Albendazole + Micronutrients | Placebo | Digit forward | male | 0.36 | -0.13 | 0.85 |
| **Nga** | Albendazole + Micronutrients | Placebo | Digit back | female | -0.11 | -0.50 | 0.29 |
| **Nga** | Albendazole + Micronutrients | Placebo | Digit back | male | 0.04 | -0.36 | 0.45 |
| **Nga** | Albendazole + Micronutrients | Placebo | Block score | female | -1.44 | -4.04 | 1.15 |
| **Nga** | Albendazole + Micronutrients | Placebo | Block score | male | 0.18 | -2.46 | 2.81 |
| **Nga** | Albendazole + Micronutrients | Placebo | Code score | female | -0.25 | -3.21 | 2.72 |
| **Nga** | Albendazole + Micronutrients | Placebo | Code score | male | 2.96 | -0.27 | 6.19 |
| **Nga** | Micronutrients | Placebo | Raven score | female | 0.24 | -1.29 | 1.76 |
| **Nga** | Micronutrients | Placebo | Raven score | male | **1.55** | **0.04** | **3.05** |
| **Nga** | Micronutrients | Placebo | Digit forward | female | **0.59** | **0.13** | **1.04** |
| **Nga** | Micronutrients | Placebo | Digit forward | male | **0.50** | **0.01** | **1.00** |
| **Nga** | Micronutrients | Placebo | Digit back | female | -0.05 | -0.45 | 0.34 |
| **Nga** | Micronutrients | Placebo | Digit back | male | 0.09 | -0.32 | 0.50 |
| **Nga** | Micronutrients | Placebo | Block score | female | -0.24 | -2.66 | 2.17 |
| **Nga** | Micronutrients | Placebo | Block score | male | -1.16 | -3.83 | 1.50 |
| **Nga** | Micronutrients | Placebo | Code score | female | 1.31 | -1.73 | 4.35 |
| **Nga** | Micronutrients | Placebo | Code score | male | 1.50 | -1.73 | 4.74 |
| **Stoltzfus04** | Iron | Placebo | Language skills | female | -0.64 | -2.21 | 0.93 |
| **Stoltzfus04** | Iron | Placebo | Language skills | male | 1.44 | -0.22 | 3.09 |
| **Stoltzfus04** | Iron | Placebo | Motor skills | female | 0.01 | -0.09 | 0.12 |
| **Stoltzfus04** | Iron | Placebo | Motor skills | male | -0.03 | -0.16 | 0.10 |
| **Stoltzfus04** | Mebendazole (high) | Placebo | Language skills | female | -0.56 | -2.18 | 1.06 |
| **Stoltzfus04** | Mebendazole (high) | Placebo | Language skills | male | 1.17 | -0.48 | 2.82 |
| **Stoltzfus04** | Mebendazole (high) | Placebo | Motor skills | female | -0.01 | -0.15 | 0.13 |
| **Stoltzfus04** | Mebendazole (high) | Placebo | Motor skills | male | 0.01 | -0.12 | 0.15 |
| **Stoltzfus04** | Mebendazole (high) + iron | Placebo | Language skills | female | -0.27 | -1.82 | 1.29 |
| **Stoltzfus04** | Mebendazole (high) + iron | Placebo | Language skills | male | 1.53 | -0.09 | 3.14 |
| **Stoltzfus04** | Mebendazole (high) + iron | Placebo | Motor skills | female | -0.05 | -0.15 | 0.05 |
| **Stoltzfus04** | Mebendazole (high) + iron | Placebo | Motor skills | male | 0.05 | -0.09 | 0.20 |
| **Solon** | Albendazole | Placebo | Verbal ability | female | -0.03 | -0.86 | 0.80 |
| **Solon** | Albendazole | Placebo | Verbal ability | male | -0.08 | -0.89 | 0.74 |
| **Solon** | Albendazole | Placebo | Quantitative ability | female | -0.09 | -0.63 | 0.45 |
| **Solon** | Albendazole | Placebo | Quantitative ability | male | 0.11 | -0.57 | 0.80 |
| **Solon** | Albendazole | Placebo | Non-verbal ability | female | 0.03 | -0.50 | 0.56 |
| **Solon** | Albendazole | Placebo | Non-verbal ability | male | 0.16 | -0.41 | 0.74 |
| **Solon** | Albendazole | Placebo | Total cognition score | female | -0.09 | -1.30 | 1.11 |
| **Solon** | Albendazole | Placebo | Total cognition score | male | 0.20 | -1.14 | 1.54 |
| **Solon** | Albendazole + Micronutrients | Placebo | Verbal ability | female | -0.02 | -0.83 | 0.79 |
| **Solon** | Albendazole + Micronutrients | Placebo | Verbal ability | male | 0.68 | -0.16 | 1.52 |
| **Solon** | Albendazole + Micronutrients | Placebo | Quantitative ability | female | -0.08 | -0.68 | 0.53 |
| **Solon** | Albendazole + Micronutrients | Placebo | Quantitative ability | male | 0.20 | -0.48 | 0.89 |
| **Solon** | Albendazole + Micronutrients | Placebo | Non-verbal ability | female | -0.30 | -0.85 | 0.25 |
| **Solon** | Albendazole + Micronutrients | Placebo | Non-verbal ability | male | 0.09 | -0.46 | 0.65 |
| **Solon** | Albendazole + Micronutrients | Placebo | Total cognition score | female | -0.41 | -1.56 | 0.75 |
| **Solon** | Albendazole + Micronutrients | Placebo | Total cognition score | male | 0.97 | -0.37 | 2.32 |
| **Solon** | Micronutrients | Placebo | Verbal ability | female | -0.21 | -1.00 | 0.57 |
| **Solon** | Micronutrients | Placebo | Verbal ability | male | 0.35 | -0.43 | 1.12 |
| **Solon** | Micronutrients | Placebo | Quantitative ability | female | 0.26 | -0.26 | 0.79 |
| **Solon** | Micronutrients | Placebo | Quantitative ability | male | 0.09 | -0.60 | 0.79 |
| **Solon** | Micronutrients | Placebo | Non-verbal ability | female | 0.05 | -0.51 | 0.61 |
| **Solon** | Micronutrients | Placebo | Non-verbal ability | male | -0.20 | -0.80 | 0.39 |
| **Solon** | Micronutrients | Placebo | Total cognition score | female | 0.10 | -1.06 | 1.27 |
| **Solon** | Micronutrients | Placebo | Total cognition score | male | 0.24 | -1.05 | 1.52 |

***A Lumbricoides***

| **Study** | **Treatment** | **Comparator** | **Cognition outcome** | **Sub-group** | **EE** | **95% LCI** | **95% UCI** |
| --- | --- | --- | --- | --- | --- | --- | --- |
| **Ebenezer** | Mebendazole + iron | Placebo | Single digit attention score | No infection | -0.24 | -1.33 | 0.84 |
| **Ebenezer** | Mebendazole + iron | Placebo | Single digit attention score | Low intensity | 1.13 | -1.14 | 3.41 |
| **Ebenezer** | Mebendazole + iron | Placebo | Single digit attention score | High intensity | -0.03 | -2.04 | 1.97 |
| **Ebenezer** | Mebendazole + iron | Placebo | Double digit attention score | No infection | 0.04 | -0.99 | 1.06 |
| **Ebenezer** | Mebendazole + iron | Placebo | Double digit attention score | Low intensity | 0.15 | -1.99 | 2.30 |
| **Ebenezer** | Mebendazole + iron | Placebo | Double digit attention score | High intensity | -0.24 | -1.74 | 1.27 |
| **Ebenezer** | Mebendazole + iron | Placebo | Math score | No infection | 1.99 | -0.77 | 4.74 |
| **Ebenezer** | Mebendazole + iron | Placebo | Math score | Low intensity | 4.62 | -2.09 | 11.32 |
| **Ebenezer** | Mebendazole + iron | Placebo | Math score | High intensity | 2.76 | -2.29 | 7.80 |
| **Ebenezer** | Mebendazole + iron | Placebo | Tamil language score | No infection | -1.67 | -4.06 | 0.72 |
| **Ebenezer** | Mebendazole + iron | Placebo | Tamil language score | Low intensity | -7.77 | -15.92 | 0.39 |
| **Ebenezer** | Mebendazole + iron | Placebo | Tamil language score | High intensity | 3.24 | -2.79 | 9.27 |
| **Liu** | Albendazole | Placebo | Processing speed index | No infection | 1.28 | -0.21 | 2.77 |
| **Liu** | Albendazole | Placebo | Processing speed index | Low intensity | 0.73 | -1.34 | 2.80 |
| **Liu** | Albendazole | Placebo | Processing speed index | High intensity | 0.95 | -5.24 | 7.13 |
| **Liu** | Albendazole | Placebo | Working memory index | No infection | 0.73 | -0.49 | 1.94 |
| **Liu** | Albendazole | Placebo | Working memory index | Low intensity | 0.11 | -1.44 | 1.66 |
| **Liu** | Albendazole | Placebo | Working memory index | High intensity | 0.33 | -6.59 | 7.25 |
| **Liu** | Albendazole | Placebo | TIMSS z-score | No infection | 0.00 | -0.10 | 0.09 |
| **Liu** | Albendazole | Placebo | TIMSS z-score | Low intensity | -0.06 | -0.17 | 0.05 |
| **Liu** | Albendazole | Placebo | TIMSS z-score | High intensity | -0.19 | -0.52 | 0.15 |
| **Nga** | Albendazole | Placebo | Raven score | No infection | **-2.04** | **-3.67** | **-0.40** |
| **Nga** | Albendazole | Placebo | Raven score | Low intensity | -1.52 | -7.93 | 4.89 |
| **Nga** | Albendazole | Placebo | Raven score | High intensity | 1.35 | -0.05 | 2.75 |
| **Nga** | Albendazole | Placebo | Digit forward | No infection | 0.56 | -0.02 | 1.14 |
| **Nga** | Albendazole | Placebo | Digit forward | Low intensity | 0.19 | -2.16 | 2.54 |
| **Nga** | Albendazole | Placebo | Digit forward | High intensity | 0.06 | -0.32 | 0.44 |
| **Nga** | Albendazole | Placebo | Digit back | No infection | 0.35 | -0.20 | 0.90 |
| **Nga** | Albendazole | Placebo | Digit back | Low intensity | 0.67 | -0.23 | 1.56 |
| **Nga** | Albendazole | Placebo | Digit back | High intensity | 0.03 | -0.29 | 0.35 |
| **Nga** | Albendazole | Placebo | Block score | No infection | 1.54 | -1.41 | 4.49 |
| **Nga** | Albendazole | Placebo | Block score | Low intensity | -3.01 | -12.90 | 6.87 |
| **Nga** | Albendazole | Placebo | Block score | High intensity | 0.36 | -1.88 | 2.60 |
| **Nga** | Albendazole | Placebo | Code score | No infection | 1.79 | -1.97 | 5.56 |
| **Nga** | Albendazole | Placebo | Code score | Low intensity | 6.93 | -3.75 | 17.61 |
| **Nga** | Albendazole | Placebo | Code score | High intensity | -0.54 | -3.37 | 2.29 |
| **Nga** | Albendazole + Micronutrients | Placebo | Raven score | No infection | 0.04 | -1.61 | 1.69 |
| **Nga** | Albendazole + Micronutrients | Placebo | Raven score | Low intensity | -1.70 | -7.53 | 4.12 |
| **Nga** | Albendazole + Micronutrients | Placebo | Raven score | High intensity | 0.15 | -1.29 | 1.60 |
| **Nga** | Albendazole + Micronutrients | Placebo | Digit forward | No infection | 0.52 | -0.08 | 1.12 |
| **Nga** | Albendazole + Micronutrients | Placebo | Digit forward | Low intensity | 0.29 | -1.88 | 2.45 |
| **Nga** | Albendazole + Micronutrients | Placebo | Digit forward | High intensity | 0.25 | -0.14 | 0.65 |
| **Nga** | Albendazole + Micronutrients | Placebo | Digit back | No infection | 0.25 | -0.33 | 0.84 |
| **Nga** | Albendazole + Micronutrients | Placebo | Digit back | Low intensity | **1.52** | **0.65** | **2.38** |
| **Nga** | Albendazole + Micronutrients | Placebo | Digit back | High intensity | -0.30 | -0.63 | 0.03 |
| **Nga** | Albendazole + Micronutrients | Placebo | Block score | No infection | -2.64 | -5.89 | 0.61 |
| **Nga** | Albendazole + Micronutrients | Placebo | Block score | Low intensity | 3.67 | -6.25 | 13.58 |
| **Nga** | Albendazole + Micronutrients | Placebo | Block score | High intensity | 0.06 | -2.23 | 2.36 |
| **Nga** | Albendazole + Micronutrients | Placebo | Code score | No infection | 3.35 | -0.52 | 7.21 |
| **Nga** | Albendazole + Micronutrients | Placebo | Code score | Low intensity | 10.21 | -0.52 | 20.95 |
| **Nga** | Albendazole + Micronutrients | Placebo | Code score | High intensity | -0.32 | -3.08 | 2.44 |
| **Nga** | Micronutrients | Placebo | Raven score | No infection | 0.23 | -1.31 | 1.77 |
| **Nga** | Micronutrients | Placebo | Raven score | Low intensity | **-5.64** | **-10.98** | **-0.31** |
| **Nga** | Micronutrients | Placebo | Raven score | High intensity | 1.57 | 0.14 | 3.00 |
| **Nga** | Micronutrients | Placebo | Digit forward | No infection | **0.64** | **0.05** | **1.23** |
| **Nga** | Micronutrients | Placebo | Digit forward | Low intensity | 1.12 | -0.84 | 3.07 |
| **Nga** | Micronutrients | Placebo | Digit forward | High intensity | **0.58** | **0.19** | **0.97** |
| **Nga** | Micronutrients | Placebo | Digit back | No infection | 0.33 | -0.25 | 0.91 |
| **Nga** | Micronutrients | Placebo | Digit back | Low intensity | -0.26 | -1.01 | 0.48 |
| **Nga** | Micronutrients | Placebo | Digit back | High intensity | -0.21 | -0.54 | 0.12 |
| **Nga** | Micronutrients | Placebo | Block score | No infection | -0.44 | -3.54 | 2.66 |
| **Nga** | Micronutrients | Placebo | Block score | Low intensity | -1.62 | -9.85 | 6.60 |
| **Nga** | Micronutrients | Placebo | Block score | High intensity | -0.73 | -3.03 | 1.58 |
| **Nga** | Micronutrients | Placebo | Code score | No infection | 2.88 | -1.02 | 6.79 |
| **Nga** | Micronutrients | Placebo | Code score | Low intensity | 1.71 | -7.18 | 10.60 |
| **Nga** | Micronutrients | Placebo | Code score | High intensity | 0.42 | -2.32 | 3.17 |
| **Stoltzfus04** | Iron | Placebo | Language skills | No infection | 0.29 | -1.26 | 1.84 |
| **Stoltzfus04** | Iron | Placebo | Language skills | Low intensity | 0.85 | -0.93 | 2.63 |
| **Stoltzfus04** | Iron | Placebo | Language skills | High intensity | NA | NA | NA |
| **Stoltzfus04** | Iron | Placebo | Motor skills | No infection | 0.01 | -0.10 | 0.12 |
| **Stoltzfus04** | Iron | Placebo | Motor skills | Low intensity | -0.06 | -0.20 | 0.07 |
| **Stoltzfus04** | Iron | Placebo | Motor skills | High intensity | NA | NA | NA |
| **Stoltzfus04** | Mebendazole (high) | Placebo | Language skills | No infection | -0.33 | -1.88 | 1.22 |
| **Stoltzfus04** | Mebendazole (high) | Placebo | Language skills | Low intensity | 1.26 | -0.55 | 3.06 |
| **Stoltzfus04** | Mebendazole (high) | Placebo | Language skills | High intensity | NA | NA | NA |
| **Stoltzfus04** | Mebendazole (high) | Placebo | Motor skills | No infection | 0.00 | -0.14 | 0.14 |
| **Stoltzfus04** | Mebendazole (high) | Placebo | Motor skills | Low intensity | -0.02 | -0.14 | 0.11 |
| **Stoltzfus04** | Mebendazole (high) | Placebo | Motor skills | High intensity | NA | NA | NA |
| **Stoltzfus04** | Mebendazole (high) + iron | Placebo | Language skills | No infection | 0.24 | -1.26 | 1.74 |
| **Stoltzfus04** | Mebendazole (high) + iron | Placebo | Language skills | Low intensity | 1.29 | -0.44 | 3.02 |
| **Stoltzfus04** | Mebendazole (high) + iron | Placebo | Language skills | High intensity | NA | NA | NA |
| **Stoltzfus04** | Mebendazole (high) + iron | Placebo | Motor skills | No infection | 0.02 | -0.10 | 0.14 |
| **Stoltzfus04** | Mebendazole (high) + iron | Placebo | Motor skills | Low intensity | -0.02 | -0.17 | 0.13 |
| **Stoltzfus04** | Mebendazole (high) + iron | Placebo | Motor skills | High intensity | NA | NA | NA |
| **Solon** | Albendazole | Placebo | Verbal ability | No infection | -0.45 | -1.31 | 0.42 |
| **Solon** | Albendazole | Placebo | Verbal ability | Low intensity | -0.25 | -1.73 | 1.24 |
| **Solon** | Albendazole | Placebo | Verbal ability | High intensity | 0.57 | -0.36 | 1.49 |
| **Solon** | Albendazole | Placebo | Quantitative ability | No infection | 0.42 | -0.17 | 1.02 |
| **Solon** | Albendazole | Placebo | Quantitative ability | Low intensity | -0.34 | -1.48 | 0.80 |
| **Solon** | Albendazole | Placebo | Quantitative ability | High intensity | -0.27 | -1.08 | 0.53 |
| **Solon** | Albendazole | Placebo | Non-verbal ability | No infection | 0.23 | -0.38 | 0.83 |
| **Solon** | Albendazole | Placebo | Non-verbal ability | Low intensity | 0.98 | -0.19 | 2.15 |
| **Solon** | Albendazole | Placebo | Non-verbal ability | High intensity | -0.30 | -0.95 | 0.35 |
| **Solon** | Albendazole | Placebo | Total cognition score | No infection | 0.20 | -1.06 | 1.47 |
| **Solon** | Albendazole | Placebo | Total cognition score | Low intensity | 0.40 | -1.95 | 2.74 |
| **Solon** | Albendazole | Placebo | Total cognition score | High intensity | -0.01 | -1.53 | 1.51 |
| **Solon** | Albendazole + micronutrients | Placebo | Verbal ability | No infection | 0.43 | -0.44 | 1.31 |
| **Solon** | Albendazole + micronutrients | Placebo | Verbal ability | Low intensity | 0.66 | -0.91 | 2.23 |
| **Solon** | Albendazole + micronutrients | Placebo | Verbal ability | High intensity | 0.34 | -0.60 | 1.29 |
| **Solon** | Albendazole + micronutrients | Placebo | Quantitative ability | No infection | 0.49 | -0.22 | 1.20 |
| **Solon** | Albendazole + micronutrients | Placebo | Quantitative ability | Low intensity | -1.13 | -2.51 | 0.25 |
| **Solon** | Albendazole + micronutrients | Placebo | Quantitative ability | High intensity | -0.11 | -0.83 | 0.62 |
| **Solon** | Albendazole + micronutrients | Placebo | Non-verbal ability | No infection | 0.18 | -0.42 | 0.78 |
| **Solon** | Albendazole + micronutrients | Placebo | Non-verbal ability | Low intensity | -0.04 | -1.51 | 1.42 |
| **Solon** | Albendazole + micronutrients | Placebo | Non-verbal ability | High intensity | -0.36 | -0.95 | 0.23 |
| **Solon** | Albendazole + micronutrients | Placebo | Total cognition score | No infection | 1.10 | -0.14 | 2.34 |
| **Solon** | Albendazole + micronutrients | Placebo | Total cognition score | Low intensity | -0.51 | -3.26 | 2.25 |
| **Solon** | Albendazole + micronutrients | Placebo | Total cognition score | High intensity | -0.12 | -1.55 | 1.31 |
| **Solon** | Micronutrients | Placebo | Verbal ability | No infection | -0.05 | -0.86 | 0.76 |
| **Solon** | Micronutrients | Placebo | Verbal ability | Low intensity | 0.07 | -1.41 | 1.55 |
| **Solon** | Micronutrients | Placebo | Verbal ability | High intensity | 0.42 | -0.55 | 1.39 |
| **Solon** | Micronutrients | Placebo | Quantitative ability | No infection | 0.48 | -0.14 | 1.10 |
| **Solon** | Micronutrients | Placebo | Quantitative ability | Low intensity | -0.15 | -1.27 | 0.96 |
| **Solon** | Micronutrients | Placebo | Quantitative ability | High intensity | -0.09 | -0.83 | 0.65 |
| **Solon** | Micronutrients | Placebo | Non-verbal ability | No infection | 0.09 | -0.49 | 0.66 |
| **Solon** | Micronutrients | Placebo | Non-verbal ability | Low intensity | -0.33 | -1.55 | 0.89 |
| **Solon** | Micronutrients | Placebo | Non-verbal ability | High intensity | -0.10 | -0.81 | 0.61 |
| **Solon** | Micronutrients | Placebo | Total cognition score | No infection | 0.51 | -0.66 | 1.69 |
| **Solon** | Micronutrients | Placebo | Total cognition score | Low intensity | -0.41 | -2.90 | 2.09 |
| **Solon** | Micronutrients | Placebo | Total cognition score | High intensity | 0.23 | -1.31 | 1.77 |

Hookworm

| **Study** | **Treatment** | **Comparator** | **Cognition outcome** | **Sub-group** | **EE** | **95% LCI** | **95% UCI** |
| --- | --- | --- | --- | --- | --- | --- | --- |
| **Ebenezer** | Mebendazole + iron | Placebo | Single digit attention score | No infection | -0.15 | -1.14 | 0.83 |
| **Ebenezer** | Mebendazole + iron | Placebo | Single digit attention score | Low intensity | -0.30 | -4.25 | 3.65 |
| **Ebenezer** | Mebendazole + iron | Placebo | Single digit attention score | High intensity | 2.03 | -1.27 | 5.32 |
| **Ebenezer** | Mebendazole + iron | Placebo | Double digit attention score | No infection | -0.05 | -0.97 | 0.87 |
| **Ebenezer** | Mebendazole + iron | Placebo | Double digit attention score | Low intensity | -0.21 | -3.36 | 2.94 |
| **Ebenezer** | Mebendazole + iron | Placebo | Double digit attention score | High intensity | 1.19 | -1.70 | 4.08 |
| **Ebenezer** | Mebendazole + iron | Placebo | Math score | No infection | 1.90 | -0.69 | 4.49 |
| **Ebenezer** | Mebendazole + iron | Placebo | Math score | Low intensity | 4.74 | -2.29 | 11.77 |
| **Ebenezer** | Mebendazole + iron | Placebo | Math score | High intensity | 7.96 | -2.36 | 18.28 |
| **Ebenezer** | Mebendazole + iron | Placebo | Tamil language score | No infection | -2.03 | -4.40 | 0.34 |
| **Ebenezer** | Mebendazole + iron | Placebo | Tamil language score | Low intensity | 5.69 | -3.74 | 15.12 |
| **Ebenezer** | Mebendazole + iron | Placebo | Tamil language score | High intensity | 2.07 | -11.92 | 16.05 |
| **Liu** | Albendazole | Placebo | Processing speed index | No infection | 1.18 | -0.33 | 2.70 |
| **Liu** | Albendazole | Placebo | Processing speed index | Low intensity | 0.74 | -1.28 | 2.77 |
| **Liu** | Albendazole | Placebo | Processing speed index | High intensity | NA | NA | NA |
| **Liu** | Albendazole | Placebo | Working memory index | No infection | 0.72 | -0.47 | 1.91 |
| **Liu** | Albendazole | Placebo | Working memory index | Low intensity | 0.11 | -1.47 | 1.69 |
| **Liu** | Albendazole | Placebo | Working memory index | High intensity | NA | NA | NA |
| **Liu** | Albendazole | Placebo | TIMSS z-score | No infection | -0.02 | -0.11 | 0.07 |
| **Liu** | Albendazole | Placebo | TIMSS z-score | Low intensity | -0.06 | -0.18 | 0.06 |
| **Liu** | Albendazole | Placebo | TIMSS z-score | High intensity | NA | NA | NA |
| **Nga** | Albendazole | Placebo | Raven score | No infection | 0.04 | -1.07 | 1.15 |
| **Nga** | Albendazole | Placebo | Raven score | Low intensity | NA | NA | NA |
| **Nga** | Albendazole | Placebo | Raven score | High intensity | 2.00 | NA | NA |
| **Nga** | Albendazole | Placebo | Digit forward | No infection | 0.30 | -0.02 | 0.62 |
| **Nga** | Albendazole | Placebo | Digit forward | Low intensity | NA | NA | NA |
| **Nga** | Albendazole | Placebo | Digit forward | High intensity | -0.40 | NA | NA |
| **Nga** | Albendazole | Placebo | Digit back | No infection | 0.15 | -0.12 | 0.43 |
| **Nga** | Albendazole | Placebo | Digit back | Low intensity | NA | NA | NA |
| **Nga** | Albendazole | Placebo | Digit back | High intensity | 0.17 | NA | NA |
| **Nga** | Albendazole | Placebo | Block score | No infection | 1.04 | -0.78 | 2.85 |
| **Nga** | Albendazole | Placebo | Block score | Low intensity | NA | NA | NA |
| **Nga** | Albendazole | Placebo | Block score | High intensity | -1.42 | NA | NA |
| **Nga** | Albendazole | Placebo | Code score | No infection | 0.32 | -1.91 | 2.54 |
| **Nga** | Albendazole | Placebo | Code score | Low intensity | NA | NA | NA |
| **Nga** | Albendazole | Placebo | Code score | High intensity | 3.31 | NA | NA |
| **Nga** | Albendazole + Micronutrients | Placebo | Raven score | No infection | 0.13 | -1.02 | 1.28 |
| **Nga** | Albendazole + Micronutrients | Placebo | Raven score | Low intensity | NA | NA | NA |
| **Nga** | Albendazole + Micronutrients | Placebo | Raven score | High intensity | -2.25 | NA | NA |
| **Nga** | Albendazole + Micronutrients | Placebo | Digit forward | No infection | **0.39** | **0.06** | **0.72** |
| **Nga** | Albendazole + Micronutrients | Placebo | Digit forward | Low intensity | NA | NA | NA |
| **Nga** | Albendazole + Micronutrients | Placebo | Digit forward | High intensity | -0.31 | NA | NA |
| **Nga** | Albendazole + Micronutrients | Placebo | Digit back | No infection | -0.05 | -0.34 | 0.23 |
| **Nga** | Albendazole + Micronutrients | Placebo | Digit back | Low intensity | NA | NA | NA |
| **Nga** | Albendazole + Micronutrients | Placebo | Digit back | High intensity | -0.27 | NA | NA |
| **Nga** | Albendazole + Micronutrients | Placebo | Block score | No infection | -0.36 | -2.28 | 1.55 |
| **Nga** | Albendazole + Micronutrients | Placebo | Block score | Low intensity | NA | NA | NA |
| **Nga** | Albendazole + Micronutrients | Placebo | Block score | High intensity | -4.05 | NA | NA |
| **Nga** | Albendazole + Micronutrients | Placebo | Code score | No infection | 1.26 | -0.96 | 3.49 |
| **Nga** | Albendazole + Micronutrients | Placebo | Code score | Low intensity | NA | NA | NA |
| **Nga** | Albendazole + Micronutrients | Placebo | Code score | High intensity | 4.06 | NA | NA |
| **Nga** | Micronutrients | Placebo | Raven score | No infection | 0.93 | -0.17 | 2.03 |
| **Nga** | Micronutrients | Placebo | Raven score | Low intensity | NA | NA | NA |
| **Nga** | Micronutrients | Placebo | Raven score | High intensity | 2.60 | NA | NA |
| **Nga** | Micronutrients | Placebo | Digit forward | No infection | **0.58** | **0.26** | **0.90** |
| **Nga** | Micronutrients | Placebo | Digit forward | Low intensity | NA | NA | NA |
| **Nga** | Micronutrients | Placebo | Digit forward | High intensity | 0.02 | NA | NA |
| **Nga** | Micronutrients | Placebo | Digit back | No infection | -0.03 | -0.31 | 0.26 |
| **Nga** | Micronutrients | Placebo | Digit back | Low intensity | NA | NA | NA |
| **Nga** | Micronutrients | Placebo | Digit back | High intensity | 0.14 | NA | NA |
| **Nga** | Micronutrients | Placebo | Block score | No infection | -0.62 | -2.44 | 1.21 |
| **Nga** | Micronutrients | Placebo | Block score | Low intensity | NA | NA | NA |
| **Nga** | Micronutrients | Placebo | Block score | High intensity | -1.69 | NA | NA |
| **Nga** | Micronutrients | Placebo | Code score | No infection | 1.40 | -0.87 | 3.67 |
| **Nga** | Micronutrients | Placebo | Code score | Low intensity | NA | NA | NA |
| **Nga** | Micronutrients | Placebo | Code score | High intensity | 8.60 | NA | NA |
| **Stoltzfus04** | Iron | Placebo | Language skills | No infection | 0.64 | -1.16 | 2.43 |
| **Stoltzfus04** | Iron | Placebo | Language skills | Low intensity | -0.35 | -1.81 | 1.10 |
| **Stoltzfus04** | Iron | Placebo | Language skills | High intensity | NA | NA | NA |
| **Stoltzfus04** | Iron | Placebo | Motor skills | No infection | -0.03 | -0.14 | 0.08 |
| **Stoltzfus04** | Iron | Placebo | Motor skills | Low intensity | 0.02 | -0.10 | 0.14 |
| **Stoltzfus04** | Iron | Placebo | Motor skills | High intensity | NA | NA | NA |
| **Stoltzfus04** | Mebendazole (high) | Placebo | Language skills | No infection | -0.40 | -2.10 | 1.31 |
| **Stoltzfus04** | Mebendazole (high) | Placebo | Language skills | Low intensity | 0.65 | -0.74 | 2.04 |
| **Stoltzfus04** | Mebendazole (high) | Placebo | Language skills | High intensity | NA | NA | NA |
| **Stoltzfus04** | Mebendazole (high) | Placebo | Motor skills | No infection | 0.00 | -0.13 | 0.12 |
| **Stoltzfus04** | Mebendazole (high) | Placebo | Motor skills | Low intensity | 0.00 | -0.12 | 0.13 |
| **Stoltzfus04** | Mebendazole (high) | Placebo | Motor skills | High intensity | NA | NA | NA |
| **Stoltzfus04** | Mebendazole (high) + iron | Placebo | Language skills | No infection | 0.19 | -1.70 | 2.08 |
| **Stoltzfus04** | Mebendazole (high) + iron | Placebo | Language skills | Low intensity | 0.88 | -0.51 | 2.27 |
| **Stoltzfus04** | Mebendazole (high) + iron | Placebo | Language skills | High intensity | NA | NA | NA |
| **Stoltzfus04** | Mebendazole (high) + iron | Placebo | Motor skills | No infection | 0.01 | -0.11 | 0.14 |
| **Stoltzfus04** | Mebendazole (high) + iron | Placebo | Motor skills | Low intensity | 0.02 | -0.11 | 0.14 |
| **Stoltzfus04** | Mebendazole (high) + iron | Placebo | Motor skills | High intensity | NA | NA | NA |
| **Solon** | Albendazole | Placebo | Verbal ability | No infection | -0.32 | -1.00 | 0.36 |
| **Solon** | Albendazole | Placebo | Verbal ability | Low intensity | 0.89 | -0.29 | 2.07 |
| **Solon** | Albendazole | Placebo | Verbal ability | High intensity | NA | NA | NA |
| **Solon** | Albendazole | Placebo | Quantitative ability | No infection | 0.01 | -0.48 | 0.50 |
| **Solon** | Albendazole | Placebo | Quantitative ability | Low intensity | 0.11 | -0.82 | 1.03 |
| **Solon** | Albendazole | Placebo | Quantitative ability | High intensity | NA | NA | NA |
| **Solon** | Albendazole | Placebo | Non-verbal ability | No infection | 0.20 | -0.26 | 0.67 |
| **Solon** | Albendazole | Placebo | Non-verbal ability | Low intensity | -0.30 | -1.07 | 0.46 |
| **Solon** | Albendazole | Placebo | Non-verbal ability | High intensity | NA | NA | NA |
| **Solon** | Albendazole | Placebo | Total cognition score | No infection | -0.11 | -1.15 | 0.93 |
| **Solon** | Albendazole | Placebo | Total cognition score | Low intensity | 0.69 | -1.19 | 2.57 |
| **Solon** | Albendazole | Placebo | Total cognition score | High intensity | NA | NA | NA |
| **Solon** | Albendazole + micronutrients | Placebo | Verbal ability | No infection | 0.46 | -0.19 | 1.10 |
| **Solon** | Albendazole + micronutrients | Placebo | Verbal ability | Low intensity | 0.28 | -0.97 | 1.52 |
| **Solon** | Albendazole + micronutrients | Placebo | Verbal ability | High intensity | NA | NA | NA |
| **Solon** | Albendazole + micronutrients | Placebo | Quantitative ability | No infection | 0.14 | -0.37 | 0.66 |
| **Solon** | Albendazole + micronutrients | Placebo | Quantitative ability | Low intensity | -0.17 | -1.14 | 0.79 |
| **Solon** | Albendazole + micronutrients | Placebo | Quantitative ability | High intensity | NA | NA | NA |
| **Solon** | Albendazole + micronutrients | Placebo | Non-verbal ability | No infection | 0.08 | -0.38 | 0.53 |
| **Solon** | Albendazole + micronutrients | Placebo | Non-verbal ability | Low intensity | -0.63 | -1.38 | 0.13 |
| **Solon** | Albendazole + micronutrients | Placebo | Non-verbal ability | High intensity | NA | NA | NA |
| **Solon** | Albendazole + micronutrients | Placebo | Total cognition score | No infection | 0.68 | -0.31 | 1.66 |
| **Solon** | Albendazole + micronutrients | Placebo | Total cognition score | Low intensity | -0.52 | -2.28 | 1.23 |
| **Solon** | Albendazole + micronutrients | Placebo | Total cognition score | High intensity | NA | NA | NA |
| **Solon** | Micronutrients | Placebo | Verbal ability | No infection | -0.08 | -0.73 | 0.57 |
| **Solon** | Micronutrients | Placebo | Verbal ability | Low intensity | 0.83 | -0.25 | 1.91 |
| **Solon** | Micronutrients | Placebo | Verbal ability | High intensity | NA | NA | NA |
| **Solon** | Micronutrients | Placebo | Quantitative ability | No infection | 0.25 | -0.24 | 0.74 |
| **Solon** | Micronutrients | Placebo | Quantitative ability | Low intensity | 0.16 | -0.73 | 1.04 |
| **Solon** | Micronutrients | Placebo | Quantitative ability | High intensity | NA | NA | NA |
| **Solon** | Micronutrients | Placebo | Non-verbal ability | No infection | -0.10 | -0.55 | 0.36 |
| **Solon** | Micronutrients | Placebo | Non-verbal ability | Low intensity | -0.11 | -0.87 | 0.65 |
| **Solon** | Micronutrients | Placebo | Non-verbal ability | High intensity | NA | NA | NA |
| **Solon** | Micronutrients | Placebo | Total cognition score | No infection | 0.07 | -0.91 | 1.06 |
| **Solon** | Micronutrients | Placebo | Total cognition score | Low intensity | 0.87 | -0.85 | 2.60 |
| **Solon** | Micronutrients | Placebo | Total cognition score | High intensity | NA | NA | NA |

Trichuris

| **Study** | **Treatment** | **Comparator** | **Cognition outcome** | **Sub-group** | **EE** | **95% LCI** | **95% UCI** |
| --- | --- | --- | --- | --- | --- | --- | --- |
| **Ebenezer** | Mebendazole + iron | Placebo | Single digit attention score | No infection | -0.05 | -1.06 | 0.96 |
| **Ebenezer** | Mebendazole + iron | Placebo | Single digit attention score | Low intensity | -0.57 | -3.01 | 1.87 |
| **Ebenezer** | Mebendazole + iron | Placebo | Single digit attention score | High intensity | 2.35 | -2.79 | 7.50 |
| **Ebenezer** | Mebendazole + iron | Placebo | Double digit attention score | No infection | -0.05 | -1.06 | 0.96 |
| **Ebenezer** | Mebendazole + iron | Placebo | Double digit attention score | Low intensity | -0.57 | -3.01 | 1.87 |
| **Ebenezer** | Mebendazole + iron | Placebo | Double digit attention score | High intensity | 2.35 | -2.79 | 7.50 |
| **Ebenezer** | Mebendazole + iron | Placebo | Math score | No infection | 2.06 | -0.51 | 4.63 |
| **Ebenezer** | Mebendazole + iron | Placebo | Math score | Low intensity | 5.09 | -1.65 | 11.84 |
| **Ebenezer** | Mebendazole + iron | Placebo | Math score | High intensity | 6.48 | -5.12 | 18.09 |
| **Ebenezer** | Mebendazole + iron | Placebo | Tamil language score | No infection | -1.73 | -3.98 | 0.52 |
| **Ebenezer** | Mebendazole + iron | Placebo | Tamil language score | Low intensity | 1.30 | -7.63 | 10.22 |
| **Ebenezer** | Mebendazole + iron | Placebo | Tamil language score | High intensity | 1.95 | -11.65 | 15.55 |
| **Liu** | Albendazole | Placebo | Processing speed index | No infection | 1.28 | -0.21 | 2.77 |
| **Liu** | Albendazole | Placebo | Processing speed index | Low intensity | 0.89 | -1.07 | 2.86 |
| **Liu** | Albendazole | Placebo | Processing speed index | High intensity | -6.29 | -15.08 | 2.50 |
| **Liu** | Albendazole | Placebo | Working memory index | No infection | 0.70 | -0.51 | 1.91 |
| **Liu** | Albendazole | Placebo | Working memory index | Low intensity | 0.09 | -1.36 | 1.54 |
| **Liu** | Albendazole | Placebo | Working memory index | High intensity | 2.58 | -4.74 | 9.90 |
| **Liu** | Albendazole | Placebo | TIMSS z-score | No infection | -0.01 | -0.10 | 0.09 |
| **Liu** | Albendazole | Placebo | TIMSS z-score | Low intensity | -0.06 | -0.17 | 0.05 |
| **Liu** | Albendazole | Placebo | TIMSS z-score | High intensity | -0.28 | -1.27 | 0.70 |
| **Nga** | Albendazole | Placebo | Raven score | No infection | -0.21 | -1.88 | 1.45 |
| **Nga** | Albendazole | Placebo | Raven score | Low intensity | -0.53 | -2.50 | 1.45 |
| **Nga** | Albendazole | Placebo | Raven score | High intensity | 1.20 | -0.95 | 3.36 |
| **Nga** | Albendazole | Placebo | Digit forward | No infection | 0.10 | -0.37 | 0.57 |
| **Nga** | Albendazole | Placebo | Digit forward | Low intensity | 0.09 | -0.53 | 0.70 |
| **Nga** | Albendazole | Placebo | Digit forward | High intensity | 0.63 | -0.01 | 1.28 |
| **Nga** | Albendazole | Placebo | Digit back | No infection | 0.11 | -0.32 | 0.55 |
| **Nga** | Albendazole | Placebo | Digit back | Low intensity | 0.11 | -0.36 | 0.57 |
| **Nga** | Albendazole | Placebo | Digit back | High intensity | 0.22 | -0.34 | 0.78 |
| **Nga** | Albendazole | Placebo | Block score | No infection | -0.37 | -3.21 | 2.47 |
| **Nga** | Albendazole | Placebo | Block score | Low intensity | 1.65 | -1.78 | 5.07 |
| **Nga** | Albendazole | Placebo | Block score | High intensity | 2.29 | -1.01 | 5.59 |
| **Nga** | Albendazole | Placebo | Code score | No infection | 0.44 | -2.66 | 3.55 |
| **Nga** | Albendazole | Placebo | Code score | Low intensity | 1.59 | -2.73 | 5.91 |
| **Nga** | Albendazole | Placebo | Code score | High intensity | -1.70 | -6.25 | 2.85 |
| **Nga** | Albendazole + Micronutrients | Placebo | Raven score | No infection | -0.55 | -2.25 | 1.15 |
| **Nga** | Albendazole + Micronutrients | Placebo | Raven score | Low intensity | -0.86 | -2.69 | 0.97 |
| **Nga** | Albendazole + Micronutrients | Placebo | Raven score | High intensity | 2.24 | -0.18 | 4.67 |
| **Nga** | Albendazole + Micronutrients | Placebo | Digit forward | No infection | 0.19 | -0.32 | 0.70 |
| **Nga** | Albendazole + Micronutrients | Placebo | Digit forward | Low intensity | 0.43 | -0.15 | 1.02 |
| **Nga** | Albendazole + Micronutrients | Placebo | Digit forward | High intensity | **0.68** | **0.02** | **1.35** |
| **Nga** | Albendazole + Micronutrients | Placebo | Digit back | No infection | -0.13 | -0.59 | 0.32 |
| **Nga** | Albendazole + Micronutrients | Placebo | Digit back | Low intensity | 0.16 | -0.28 | 0.60 |
| **Nga** | Albendazole + Micronutrients | Placebo | Digit back | High intensity | -0.15 | -0.73 | 0.44 |
| **Nga** | Albendazole + Micronutrients | Placebo | Block score | No infection | -0.12 | -2.98 | 2.74 |
| **Nga** | Albendazole + Micronutrients | Placebo | Block score | Low intensity | -1.26 | -4.48 | 1.96 |
| **Nga** | Albendazole + Micronutrients | Placebo | Block score | High intensity | -0.63 | -4.10 | 2.84 |
| **Nga** | Albendazole + Micronutrients | Placebo | Code score | No infection | 0.84 | -2.28 | 3.96 |
| **Nga** | Albendazole + Micronutrients | Placebo | Code score | Low intensity | 2.64 | -1.44 | 6.73 |
| **Nga** | Albendazole + Micronutrients | Placebo | Code score | High intensity | 0.97 | -3.87 | 5.81 |
| **Nga** | Micronutrients | Placebo | Raven score | No infection | 0.41 | -1.13 | 1.95 |
| **Nga** | Micronutrients | Placebo | Raven score | Low intensity | 0.56 | -1.35 | 2.48 |
| **Nga** | Micronutrients | Placebo | Raven score | High intensity | 1.85 | -0.33 | 4.02 |
| **Nga** | Micronutrients | Placebo | Digit forward | No infection | **0.51** | **0.02** | **1.01** |
| **Nga** | Micronutrients | Placebo | Digit forward | Low intensity | 0.37 | -0.24 | 0.97 |
| **Nga** | Micronutrients | Placebo | Digit forward | High intensity | **1.09** | **0.46** | **1.73** |
| **Nga** | Micronutrients | Placebo | Digit back | No infection | 0.22 | -0.23 | 0.67 |
| **Nga** | Micronutrients | Placebo | Digit back | Low intensity | -0.38 | -0.84 | 0.08 |
| **Nga** | Micronutrients | Placebo | Digit back | High intensity | -0.06 | -0.63 | 0.50 |
| **Nga** | Micronutrients | Placebo | Block score | No infection | -1.28 | -3.98 | 1.42 |
| **Nga** | Micronutrients | Placebo | Block score | Low intensity | -0.68 | -4.02 | 2.67 |
| **Nga** | Micronutrients | Placebo | Block score | High intensity | 0.41 | -2.91 | 3.74 |
| **Nga** | Micronutrients | Placebo | Code score | No infection | 3.17 | -0.11 | 6.45 |
| **Nga** | Micronutrients | Placebo | Code score | Low intensity | 0.94 | -3.29 | 5.18 |
| **Nga** | Micronutrients | Placebo | Code score | High intensity | -0.92 | -5.54 | 3.71 |
| **Stoltzfus04** | Iron | Placebo | Language skills | No infection | 0.44 | -1.92 | 2.81 |
| **Stoltzfus04** | Iron | Placebo | Language skills | Low intensity | 0.52 | -0.82 | 1.86 |
| **Stoltzfus04** | Iron | Placebo | Language skills | High intensity | NA | NA | NA |
| **Stoltzfus04** | Iron | Placebo | Motor skills | No infection | -0.01 | -0.16 | 0.13 |
| **Stoltzfus04** | Iron | Placebo | Motor skills | Low intensity | -0.02 | -0.12 | 0.07 |
| **Stoltzfus04** | Iron | Placebo | Motor skills | High intensity | NA | NA | NA |
| **Stoltzfus04** | Mebendazole (high) | Placebo | Language skills | No infection | -0.71 | -3.05 | 1.62 |
| **Stoltzfus04** | Mebendazole (high) | Placebo | Language skills | Low intensity | 0.91 | -0.46 | 2.28 |
| **Stoltzfus04** | Mebendazole (high) | Placebo | Language skills | High intensity | NA | NA | NA |
| **Stoltzfus04** | Mebendazole (high) | Placebo | Motor skills | No infection | 0.04 | -0.17 | 0.25 |
| **Stoltzfus04** | Mebendazole (high) | Placebo | Motor skills | Low intensity | -0.04 | -0.14 | 0.06 |
| **Stoltzfus04** | Mebendazole (high) | Placebo | Motor skills | High intensity | NA | NA | NA |
| **Stoltzfus04** | Mebendazole (high) + iron | Placebo | Language skills | No infection | -0.36 | -2.49 | 1.77 |
| **Stoltzfus04** | Mebendazole (high) + iron | Placebo | Language skills | Low intensity | 1.06 | -0.24 | 2.37 |
| **Stoltzfus04** | Mebendazole (high) + iron | Placebo | Language skills | High intensity | NA | NA | NA |
| **Stoltzfus04** | Mebendazole (high) + iron | Placebo | Motor skills | No infection | -0.01 | -0.18 | 0.17 |
| **Stoltzfus04** | Mebendazole (high) + iron | Placebo | Motor skills | Low intensity | -0.01 | -0.11 | 0.10 |
| **Stoltzfus04** | Mebendazole (high) + iron | Placebo | Motor skills | High intensity | NA | NA | NA |
| **Solon** | Albendazole | Placebo | Verbal ability | No infection | -0.28 | -0.98 | 0.43 |
| **Solon** | Albendazole | Placebo | Verbal ability | Low intensity | -0.14 | -1.60 | 1.33 |
| **Solon** | Albendazole | Placebo | Verbal ability | High intensity | 0.98 | -0.61 | 2.58 |
| **Solon** | Albendazole | Placebo | Quantitative ability | No infection | 0.13 | -0.40 | 0.65 |
| **Solon** | Albendazole | Placebo | Quantitative ability | Low intensity | 0.16 | -1.22 | 1.55 |
| **Solon** | Albendazole | Placebo | Quantitative ability | High intensity | -0.29 | -1.71 | 1.13 |
| **Solon** | Albendazole | Placebo | Non-verbal ability | No infection | 0.05 | -0.47 | 0.58 |
| **Solon** | Albendazole | Placebo | Non-verbal ability | Low intensity | 0.36 | -1.05 | 1.77 |
| **Solon** | Albendazole | Placebo | Non-verbal ability | High intensity | -0.06 | -1.04 | 0.93 |
| **Solon** | Albendazole | Placebo | Total cognition score | No infection | -0.10 | -1.21 | 1.01 |
| **Solon** | Albendazole | Placebo | Total cognition score | Low intensity | 0.39 | -2.44 | 3.21 |
| **Solon** | Albendazole | Placebo | Total cognition score | High intensity | 0.64 | -1.79 | 3.07 |
| **Solon** | Albendazole + micronutrients | Placebo | Verbal ability | No infection | 0.36 | -0.33 | 1.06 |
| **Solon** | Albendazole + micronutrients | Placebo | Verbal ability | Low intensity | 0.64 | -0.75 | 2.04 |
| **Solon** | Albendazole + micronutrients | Placebo | Verbal ability | High intensity | 0.42 | -1.35 | 2.19 |
| **Solon** | Albendazole + micronutrients | Placebo | Quantitative ability | No infection | -0.09 | -0.67 | 0.48 |
| **Solon** | Albendazole + micronutrients | Placebo | Quantitative ability | Low intensity | 0.49 | -0.95 | 1.93 |
| **Solon** | Albendazole + micronutrients | Placebo | Quantitative ability | High intensity | 0.29 | -1.22 | 1.80 |
| **Solon** | Albendazole + micronutrients | Placebo | Non-verbal ability | No infection | -0.12 | -0.60 | 0.37 |
| **Solon** | Albendazole + micronutrients | Placebo | Non-verbal ability | Low intensity | 0.27 | -0.79 | 1.33 |
| **Solon** | Albendazole + micronutrients | Placebo | Non-verbal ability | High intensity | -0.27 | -1.29 | 0.75 |
| **Solon** | Albendazole + micronutrients | Placebo | Total cognition score | No infection | 0.15 | -0.92 | 1.22 |
| **Solon** | Albendazole + micronutrients | Placebo | Total cognition score | Low intensity | 1.40 | -0.73 | 3.52 |
| **Solon** | Albendazole + micronutrients | Placebo | Total cognition score | High intensity | 0.44 | -1.80 | 2.69 |
| **Solon** | Micronutrients | Placebo | Verbal ability | No infection | -0.17 | -0.86 | 0.53 |
| **Solon** | Micronutrients | Placebo | Verbal ability | Low intensity | 0.62 | -0.96 | 2.20 |
| **Solon** | Micronutrients | Placebo | Verbal ability | High intensity | 0.75 | -0.65 | 2.15 |
| **Solon** | Micronutrients | Placebo | Quantitative ability | No infection | 0.33 | -0.21 | 0.86 |
| **Solon** | Micronutrients | Placebo | Quantitative ability | Low intensity | 0.24 | -1.20 | 1.69 |
| **Solon** | Micronutrients | Placebo | Quantitative ability | High intensity | -0.34 | -1.73 | 1.05 |
| **Solon** | Micronutrients | Placebo | Non-verbal ability | No infection | -0.17 | -0.64 | 0.31 |
| **Solon** | Micronutrients | Placebo | Non-verbal ability | Low intensity | 0.16 | -1.20 | 1.51 |
| **Solon** | Micronutrients | Placebo | Non-verbal ability | High intensity | 0.05 | -1.01 | 1.12 |
| **Solon** | Micronutrients | Placebo | Total cognition score | No infection | -0.01 | -1.06 | 1.05 |
| **Solon** | Micronutrients | Placebo | Total cognition score | Low intensity | 1.02 | -1.48 | 3.51 |
| **Solon** | Micronutrients | Placebo | Total cognition score | High intensity | 0.46 | -1.73 | 2.66 |

Anyworm

| **Study** | **Treatment** | **Comparator** | **Cognition outcome** | **Sub-group** | **EE** | **95% LCI** | **95% UCI** |
| --- | --- | --- | --- | --- | --- | --- | --- |
| **Ebenezer** | Mebendazole + iron | Placebo | Single digit attention score | No infection | -0.27 | -1.37 | 0.83 |
| **Ebenezer** | Mebendazole + iron | Placebo | Single digit attention score | Low intensity | 0.77 | -0.80 | 2.34 |
| **Ebenezer** | Mebendazole + iron | Placebo | Single digit attention score | High intensity | 0.98 | -2.20 | 4.15 |
| **Ebenezer** | Mebendazole + iron | Placebo | Double digit attention score | No infection | -0.06 | -1.11 | 0.99 |
| **Ebenezer** | Mebendazole + iron | Placebo | Double digit attention score | Low intensity | -0.10 | -1.39 | 1.19 |
| **Ebenezer** | Mebendazole + iron | Placebo | Double digit attention score | High intensity | 1.06 | -1.47 | 3.59 |
| **Ebenezer** | Mebendazole + iron | Placebo | Math score | No infection | 1.75 | -1.06 | 4.57 |
| **Ebenezer** | Mebendazole + iron | Placebo | Math score | Low intensity | **4.27** | **0.21** | **8.33** |
| **Ebenezer** | Mebendazole + iron | Placebo | Math score | High intensity | 2.65 | -5.97 | 11.27 |
| **Ebenezer** | Mebendazole + iron | Placebo | Tamil language score | No infection | -1.91 | -4.34 | 0.53 |
| **Ebenezer** | Mebendazole + iron | Placebo | Tamil language score | Low intensity | -0.25 | -4.82 | 4.33 |
| **Ebenezer** | Mebendazole + iron | Placebo | Tamil language score | High intensity | 3.02 | -5.87 | 11.91 |
| **Liu** | Albendazole | Placebo | Processing speed index | No infection | 1.28 | -0.21 | 2.77 |
| **Liu** | Albendazole | Placebo | Processing speed index | Low intensity | 0.76 | -1.19 | 2.72 |
| **Liu** | Albendazole | Placebo | Processing speed index | High intensity | NA | NA | NA |
| **Liu** | Albendazole | Placebo | Working memory index | No infection | 0.73 | -0.49 | 1.94 |
| **Liu** | Albendazole | Placebo | Working memory index | Low intensity | 0.13 | -1.30 | 1.57 |
| **Liu** | Albendazole | Placebo | Working memory index | High intensity | NA | NA | NA |
| **Liu** | Albendazole | Placebo | TIMSS z-score | No infection | 0.00 | -0.10 | 0.09 |
| **Liu** | Albendazole | Placebo | TIMSS z-score | Low intensity | -0.07 | -0.18 | 0.04 |
| **Liu** | Albendazole | Placebo | TIMSS z-score | High intensity | NA | NA | NA |
| **Nga** | Albendazole | Placebo | Raven score | No infection | **-3.03** | **-5.34** | **-0.73** |
| **Nga** | Albendazole | Placebo | Raven score | Low intensity | 0.58 | -0.79 | 1.96 |
| **Nga** | Albendazole | Placebo | Raven score | High intensity | 1.57 | -0.81 | 3.94 |
| **Nga** | Albendazole | Placebo | Digit forward | No infection | 0.34 | -0.38 | 1.07 |
| **Nga** | Albendazole | Placebo | Digit forward | Low intensity | 0.30 | -0.10 | 0.70 |
| **Nga** | Albendazole | Placebo | Digit forward | High intensity | 0.10 | -0.71 | 0.90 |
| **Nga** | Albendazole | Placebo | Digit back | No infection | 0.10 | -0.57 | 0.77 |
| **Nga** | Albendazole | Placebo | Digit back | Low intensity | 0.08 | -0.25 | 0.41 |
| **Nga** | Albendazole | Placebo | Digit back | High intensity | 0.26 | -0.34 | 0.86 |
| **Nga** | Albendazole | Placebo | Block score | No infection | -1.94 | -6.46 | 2.57 |
| **Nga** | Albendazole | Placebo | Block score | Low intensity | 1.66 | -0.49 | 3.80 |
| **Nga** | Albendazole | Placebo | Block score | High intensity | 2.79 | -1.47 | 7.05 |
| **Nga** | Albendazole | Placebo | Code score | No infection | 3.01 | -2.09 | 8.10 |
| **Nga** | Albendazole | Placebo | Code score | Low intensity | -0.33 | -3.08 | 2.43 |
| **Nga** | Albendazole | Placebo | Code score | High intensity | -2.31 | -7.98 | 3.36 |
| **Nga** | Albendazole + Micronutrients | Placebo | Raven score | No infection | -0.95 | -3.40 | 1.50 |
| **Nga** | Albendazole + Micronutrients | Placebo | Raven score | Low intensity | 0.43 | -0.92 | 1.78 |
| **Nga** | Albendazole + Micronutrients | Placebo | Raven score | High intensity | -1.50 | -3.99 | 0.99 |
| **Nga** | Albendazole + Micronutrients | Placebo | Digit forward | No infection | 0.27 | -0.54 | 1.09 |
| **Nga** | Albendazole + Micronutrients | Placebo | Digit forward | Low intensity | **0.43** | **0.03** | **0.83** |
| **Nga** | Albendazole + Micronutrients | Placebo | Digit forward | High intensity | 0.26 | -0.59 | 1.10 |
| **Nga** | Albendazole + Micronutrients | Placebo | Digit back | No infection | -0.24 | -0.99 | 0.50 |
| **Nga** | Albendazole + Micronutrients | Placebo | Digit back | Low intensity | -0.03 | -0.36 | 0.29 |
| **Nga** | Albendazole + Micronutrients | Placebo | Digit back | High intensity | -0.07 | -0.71 | 0.57 |
| **Nga** | Albendazole + Micronutrients | Placebo | Block score | No infection | -3.61 | -8.46 | 1.25 |
| **Nga** | Albendazole + Micronutrients | Placebo | Block score | Low intensity | -0.90 | -2.99 | 1.19 |
| **Nga** | Albendazole + Micronutrients | Placebo | Block score | High intensity | 3.33 | -0.98 | 7.64 |
| **Nga** | Albendazole + Micronutrients | Placebo | Code score | No infection | 4.06 | -1.21 | 9.32 |
| **Nga** | Albendazole + Micronutrients | Placebo | Code score | Low intensity | 1.27 | -1.43 | 3.98 |
| **Nga** | Albendazole + Micronutrients | Placebo | Code score | High intensity | -3.16 | -8.47 | 2.15 |
| **Nga** | Micronutrients | Placebo | Raven score | No infection | -0.29 | -2.40 | 1.82 |
| **Nga** | Micronutrients | Placebo | Raven score | Low intensity | 0.76 | -0.62 | 2.13 |
| **Nga** | Micronutrients | Placebo | Raven score | High intensity | 1.98 | -0.50 | 4.45 |
| **Nga** | Micronutrients | Placebo | Digit forward | No infection | **0.78** | **0.01** | **1.55** |
| **Nga** | Micronutrients | Placebo | Digit forward | Low intensity | **0.61** | **0.21** | **1.02** |
| **Nga** | Micronutrients | Placebo | Digit forward | High intensity | 0.33 | -0.51 | 1.17 |
| **Nga** | Micronutrients | Placebo | Digit back | No infection | 0.23 | -0.50 | 0.97 |
| **Nga** | Micronutrients | Placebo | Digit back | Low intensity | -0.30 | -0.63 | 0.04 |
| **Nga** | Micronutrients | Placebo | Digit back | High intensity | **0.71** | **0.07** | **1.35** |
| **Nga** | Micronutrients | Placebo | Block score | No infection | -2.45 | -6.91 | 2.00 |
| **Nga** | Micronutrients | Placebo | Block score | Low intensity | -0.42 | -2.55 | 1.71 |
| **Nga** | Micronutrients | Placebo | Block score | High intensity | -0.73 | -5.41 | 3.94 |
| **Nga** | Micronutrients | Placebo | Code score | No infection | 3.47 | -1.93 | 8.87 |
| **Nga** | Micronutrients | Placebo | Code score | Low intensity | 1.04 | -1.71 | 3.78 |
| **Nga** | Micronutrients | Placebo | Code score | High intensity | 1.15 | -4.27 | 6.57 |
| **Stoltzfus04** | Iron | Placebo | Language skills | No infection | -0.06 | -2.82 | 2.69 |
| **Stoltzfus04** | Iron | Placebo | Language skills | Low intensity | 0.55 | -0.75 | 1.84 |
| **Stoltzfus04** | Iron | Placebo | Language skills | High intensity | NA | NA | NA |
| **Stoltzfus04** | Iron | Placebo | Motor skills | No infection | -0.04 | -0.21 | 0.12 |
| **Stoltzfus04** | Iron | Placebo | Motor skills | Low intensity | 0.00 | -0.10 | 0.09 |
| **Stoltzfus04** | Iron | Placebo | Motor skills | High intensity | NA | NA | NA |
| **Stoltzfus04** | Mebendazole (high) | Placebo | Language skills | No infection | -0.67 | -3.51 | 2.17 |
| **Stoltzfus04** | Mebendazole (high) | Placebo | Language skills | Low intensity | 0.66 | -0.61 | 1.94 |
| **Stoltzfus04** | Mebendazole (high) | Placebo | Language skills | High intensity | NA | NA | NA |
| **Stoltzfus04** | Mebendazole (high) | Placebo | Motor skills | No infection | 0.06 | -0.17 | 0.30 |
| **Stoltzfus04** | Mebendazole (high) | Placebo | Motor skills | Low intensity | -0.01 | -0.11 | 0.09 |
| **Stoltzfus04** | Mebendazole (high) | Placebo | Motor skills | High intensity | NA | NA | NA |
| **Stoltzfus04** | Mebendazole (high) + iron | Placebo | Language skills | No infection | -0.30 | -2.90 | 2.31 |
| **Stoltzfus04** | Mebendazole (high) + iron | Placebo | Language skills | Low intensity | 0.77 | -0.45 | 2.00 |
| **Stoltzfus04** | Mebendazole (high) + iron | Placebo | Language skills | High intensity | NA | NA | NA |
| **Stoltzfus04** | Mebendazole (high) + iron | Placebo | Motor skills | No infection | 0.01 | -0.18 | 0.21 |
| **Stoltzfus04** | Mebendazole (high) + iron | Placebo | Motor skills | Low intensity | 0.00 | -0.11 | 0.10 |
| **Stoltzfus04** | Mebendazole (high) + iron | Placebo | Motor skills | High intensity | NA | NA | NA |
| **Solon** | Albendazole | Placebo | Verbal ability | No infection | -0.53 | -1.51 | 0.45 |
| **Solon** | Albendazole | Placebo | Verbal ability | Low intensity | 0.06 | -0.75 | 0.87 |
| **Solon** | Albendazole | Placebo | Verbal ability | High intensity | 1.42 | -0.37 | 3.20 |
| **Solon** | Albendazole | Placebo | Quantitative ability | No infection | 0.39 | -0.29 | 1.07 |
| **Solon** | Albendazole | Placebo | Quantitative ability | Low intensity | -0.13 | -0.79 | 0.52 |
| **Solon** | Albendazole | Placebo | Quantitative ability | High intensity | -0.17 | -1.47 | 1.14 |
| **Solon** | Albendazole | Placebo | Non-verbal ability | No infection | 0.14 | -0.56 | 0.84 |
| **Solon** | Albendazole | Placebo | Non-verbal ability | Low intensity | 0.16 | -0.38 | 0.71 |
| **Solon** | Albendazole | Placebo | Non-verbal ability | High intensity | -0.53 | -1.71 | 0.65 |
| **Solon** | Albendazole | Placebo | Total cognition score | No infection | 0.01 | -1.47 | 1.48 |
| **Solon** | Albendazole | Placebo | Total cognition score | Low intensity | 0.09 | -1.20 | 1.38 |
| **Solon** | Albendazole | Placebo | Total cognition score | High intensity | 0.72 | -2.08 | 3.51 |
| **Solon** | Albendazole + micronutrients | Placebo | Verbal ability | No infection | 0.59 | -0.39 | 1.57 |
| **Solon** | Albendazole + micronutrients | Placebo | Verbal ability | Low intensity | 0.09 | -0.72 | 0.91 |
| **Solon** | Albendazole + micronutrients | Placebo | Verbal ability | High intensity | 1.17 | -0.46 | 2.81 |
| **Solon** | Albendazole + micronutrients | Placebo | Quantitative ability | No infection | 0.52 | -0.30 | 1.34 |
| **Solon** | Albendazole + micronutrients | Placebo | Quantitative ability | Low intensity | -0.25 | -0.86 | 0.37 |
| **Solon** | Albendazole + micronutrients | Placebo | Quantitative ability | High intensity | 0.13 | -1.13 | 1.39 |
| **Solon** | Albendazole + micronutrients | Placebo | Non-verbal ability | No infection | 0.13 | -0.54 | 0.81 |
| **Solon** | Albendazole + micronutrients | Placebo | Non-verbal ability | Low intensity | -0.10 | -0.62 | 0.43 |
| **Solon** | Albendazole + micronutrients | Placebo | Non-verbal ability | High intensity | -0.83 | -1.94 | 0.27 |
| **Solon** | Albendazole + micronutrients | Placebo | Total cognition score | No infection | 1.24 | -0.18 | 2.67 |
| **Solon** | Albendazole + micronutrients | Placebo | Total cognition score | Low intensity | -0.25 | -1.42 | 0.93 |
| **Solon** | Albendazole + micronutrients | Placebo | Total cognition score | High intensity | 0.47 | -2.14 | 3.08 |
| **Solon** | Micronutrients | Placebo | Verbal ability | No infection | -0.35 | -1.24 | 0.55 |
| **Solon** | Micronutrients | Placebo | Verbal ability | Low intensity | 0.33 | -0.42 | 1.08 |
| **Solon** | Micronutrients | Placebo | Verbal ability | High intensity | -0.13 | -2.23 | 1.97 |
| **Solon** | Micronutrients | Placebo | Quantitative ability | No infection | 0.47 | -0.24 | 1.19 |
| **Solon** | Micronutrients | Placebo | Quantitative ability | Low intensity | 0.03 | -0.56 | 0.62 |
| **Solon** | Micronutrients | Placebo | Quantitative ability | High intensity | -0.35 | -1.97 | 1.28 |
| **Solon** | Micronutrients | Placebo | Non-verbal ability | No infection | 0.14 | -0.51 | 0.80 |
| **Solon** | Micronutrients | Placebo | Non-verbal ability | Low intensity | -0.03 | -0.57 | 0.51 |
| **Solon** | Micronutrients | Placebo | Non-verbal ability | High intensity | **-1.55** | **-2.98** | **-0.13** |
| **Solon** | Micronutrients | Placebo | Total cognition score | No infection | 0.27 | -1.07 | 1.61 |
| **Solon** | Micronutrients | Placebo | Total cognition score | Low intensity | 0.32 | -0.87 | 1.51 |
| **Solon** | Micronutrients | Placebo | Total cognition score | High intensity | -2.03 | -5.36 | 1.30 |

Anaemia

| **Study** | **Treatment** | **Comparator** | **Cognition outcome** | **Sub-group** | **EE** | **95% LCI** | **95% UCI** |
| --- | --- | --- | --- | --- | --- | --- | --- |
| **Ebenezer** | Mebendazole + iron | Placebo | Single digit attention score | anaemic | -0.12 | -1.16 | 0.92 |
| **Ebenezer** | Mebendazole + iron | Placebo | Single digit attention score | non-anaemic | 0.09 | -1.83 | 2.01 |
| **Ebenezer** | Mebendazole + iron | Placebo | Double digit attention score | anaemic | -0.02 | -0.94 | 0.90 |
| **Ebenezer** | Mebendazole + iron | Placebo | Double digit attention score | non-anaemic | -0.65 | -2.35 | 1.05 |
| **Ebenezer** | Mebendazole + iron | Placebo | Math score | anaemic | 2.56 | -0.17 | 5.29 |
| **Ebenezer** | Mebendazole + iron | Placebo | Math score | non-anaemic | 1.10 | -3.18 | 5.38 |
| **Ebenezer** | Mebendazole + iron | Placebo | Tamil language score | anaemic | -1.54 | -4.21 | 1.13 |
| **Ebenezer** | Mebendazole + iron | Placebo | Tamil language score | non-anaemic | 0.05 | -5.11 | 5.21 |
| **Liu** | Albendazole | Placebo | Processing speed index | anaemic | **1.30** | **0.01** | **2.59** |
| **Liu** | Albendazole | Placebo | Processing speed index | non-anaemic | -0.93 | -3.47 | 1.60 |
| **Liu** | Albendazole | Placebo | Working memory index | anaemic | 0.64 | -0.41 | 1.70 |
| **Liu** | Albendazole | Placebo | Working memory index | non-anaemic | -0.44 | -2.37 | 1.48 |
| **Liu** | Albendazole | Placebo | TIMSS z-score | anaemic | -0.02 | -0.10 | 0.06 |
| **Liu** | Albendazole | Placebo | TIMSS z-score | non-anaemic | -0.08 | -0.24 | 0.09 |
| **Nga** | Albendazole | Placebo | Raven score | anaemic | 0.11 | -1.10 | 1.32 |
| **Nga** | Albendazole | Placebo | Raven score | non-anaemic | 0.38 | -1.94 | 2.71 |
| **Nga** | Albendazole | Placebo | Digit forward | anaemic | 0.20 | -0.15 | 0.55 |
| **Nga** | Albendazole | Placebo | Digit forward | non-anaemic | 0.57 | -0.16 | 1.30 |
| **Nga** | Albendazole | Placebo | Digit back | anaemic | 0.20 | -0.12 | 0.53 |
| **Nga** | Albendazole | Placebo | Digit back | non-anaemic | -0.35 | -0.91 | 0.21 |
| **Nga** | Albendazole | Placebo | Block score | anaemic | 0.95 | -1.18 | 3.08 |
| **Nga** | Albendazole | Placebo | Block score | non-anaemic | 0.25 | -3.16 | 3.66 |
| **Nga** | Albendazole | Placebo | Code score | anaemic | 0.86 | -1.56 | 3.27 |
| **Nga** | Albendazole | Placebo | Code score | non-anaemic | -1.23 | -6.15 | 3.69 |
| **Nga** | Albendazole + micronutrients | Placebo | Raven score | anaemic | -0.59 | -1.90 | 0.73 |
| **Nga** | Albendazole + micronutrients | Placebo | Raven score | non-anaemic | **2.31** | **0.24** | **4.37** |
| **Nga** | Albendazole + micronutrients | Placebo | Digit forward | anaemic | 0.36 | -0.01 | 0.72 |
| **Nga** | Albendazole + micronutrients | Placebo | Digit forward | non-anaemic | 0.64 | -0.06 | 1.35 |
| **Nga** | Albendazole + micronutrients | Placebo | Digit back | anaemic | -0.09 | -0.42 | 0.24 |
| **Nga** | Albendazole + micronutrients | Placebo | Digit back | non-anaemic | -0.08 | -0.62 | 0.46 |
| **Nga** | Albendazole + micronutrients | Placebo | Block score | anaemic | -0.30 | -2.46 | 1.87 |
| **Nga** | Albendazole + micronutrients | Placebo | Block score | non-anaemic | -1.44 | -4.67 | 1.80 |
| **Nga** | Albendazole + micronutrients | Placebo | Code score | anaemic | 1.81 | -0.62 | 4.24 |
| **Nga** | Albendazole + micronutrients | Placebo | Code score | non-anaemic | -0.08 | -4.87 | 4.71 |
| **Nga** | Micronutrients | Placebo | Raven score | anaemic | 0.61 | -0.63 | 1.84 |
| **Nga** | Micronutrients | Placebo | Raven score | non-anaemic | **2.29** | **0.19** | **4.40** |
| **Nga** | Micronutrients | Placebo | Digit forward | anaemic | **0.62** | **0.27** | **0.97** |
| **Nga** | Micronutrients | Placebo | Digit forward | non-anaemic | 0.68 | -0.03 | 1.39 |
| **Nga** | Micronutrients | Placebo | Digit back | anaemic | -0.03 | -0.36 | 0.30 |
| **Nga** | Micronutrients | Placebo | Digit back | non-anaemic | -0.12 | -0.68 | 0.44 |
| **Nga** | Micronutrients | Placebo | Block score | anaemic | -0.61 | -2.77 | 1.54 |
| **Nga** | Micronutrients | Placebo | Block score | non-anaemic | -1.29 | -4.67 | 2.08 |
| **Nga** | Micronutrients | Placebo | Code score | anaemic | 2.33 | -0.16 | 4.82 |
| **Nga** | Micronutrients | Placebo | Code score | non-anaemic | -1.02 | -5.81 | 3.78 |
| **Stoltzfus04** | Iron | Placebo | Language skills | anaemic | -0.69 | -6.29 | 4.90 |
| **Stoltzfus04** | Iron | Placebo | Language skills | non-anaemic | 0.52 | -0.72 | 1.75 |
| **Stoltzfus04** | Iron | Placebo | Motor skills | anaemic | -0.08 | -0.68 | 0.53 |
| **Stoltzfus04** | Iron | Placebo | Motor skills | non-anaemic | 0.00 | -0.08 | 0.08 |
| **Stoltzfus04** | Mebendazole (high) | Placebo | Language skills | anaemic | 1.45 | -4.68 | 7.58 |
| **Stoltzfus04** | Mebendazole (high) | Placebo | Language skills | non-anaemic | 0.32 | -0.89 | 1.52 |
| **Stoltzfus04** | Mebendazole (high) | Placebo | Motor skills | anaemic | 0.02 | -0.58 | 0.63 |
| **Stoltzfus04** | Mebendazole (high) | Placebo | Motor skills | non-anaemic | 0.00 | -0.09 | 0.09 |
| **Stoltzfus04** | Mebendazole (high) + iron | Placebo | Language skills | anaemic | 0.06 | -6.59 | 6.72 |
| **Stoltzfus04** | Mebendazole (high) + iron | Placebo | Language skills | non-anaemic | 0.74 | -0.42 | 1.90 |
| **Stoltzfus04** | Mebendazole (high) + iron | Placebo | Motor skills | anaemic | -0.05 | -0.74 | 0.63 |
| **Stoltzfus04** | Mebendazole (high) + iron | Placebo | Motor skills | non-anaemic | 0.01 | -0.08 | 0.10 |
| **Solon** | Albendazole | Placebo | Verbal ability | anaemic | -0.04 | -0.79 | 0.71 |
| **Solon** | Albendazole | Placebo | Verbal ability | non-anaemic | -0.02 | -1.00 | 0.97 |
| **Solon** | Albendazole | Placebo | Quantitative ability | anaemic | 0.06 | -0.50 | 0.61 |
| **Solon** | Albendazole | Placebo | Quantitative ability | non-anaemic | 0.08 | -0.61 | 0.78 |
| **Solon** | Albendazole | Placebo | Non-verbal ability | anaemic | 0.06 | -0.45 | 0.57 |
| **Solon** | Albendazole | Placebo | Non-verbal ability | non-anaemic | 0.14 | -0.51 | 0.78 |
| **Solon** | Albendazole | Placebo | Total cognition score | anaemic | 0.08 | -1.09 | 1.25 |
| **Solon** | Albendazole | Placebo | Total cognition score | non-anaemic | 0.20 | -1.29 | 1.69 |
| **Solon** | Albendazole + micronutrients | Placebo | Verbal ability | anaemic | 0.40 | -0.29 | 1.09 |
| **Solon** | Albendazole + micronutrients | Placebo | Verbal ability | non-anaemic | 0.41 | -0.55 | 1.38 |
| **Solon** | Albendazole + micronutrients | Placebo | Quantitative ability | anaemic | 0.15 | -0.42 | 0.71 |
| **Solon** | Albendazole + micronutrients | Placebo | Quantitative ability | non-anaemic | 0.03 | -0.73 | 0.79 |
| **Solon** | Albendazole + micronutrients | Placebo | Non-verbal ability | anaemic | -0.11 | -0.60 | 0.38 |
| **Solon** | Albendazole + micronutrients | Placebo | Non-verbal ability | non-anaemic | 0.00 | -0.65 | 0.65 |
| **Solon** | Albendazole + micronutrients | Placebo | Total cognition score | anaemic | 0.43 | -0.64 | 1.51 |
| **Solon** | Albendazole + micronutrients | Placebo | Total cognition score | non-anaemic | 0.44 | -0.99 | 1.88 |
| **Solon** | Micronutrients | Placebo | Verbal ability | anaemic | -0.01 | -0.72 | 0.70 |
| **Solon** | Micronutrients | Placebo | Verbal ability | non-anaemic | 0.44 | -0.54 | 1.42 |
| **Solon** | Micronutrients | Placebo | Quantitative ability | anaemic | 0.43 | -0.12 | 0.99 |
| **Solon** | Micronutrients | Placebo | Quantitative ability | non-anaemic | -0.14 | -0.87 | 0.60 |
| **Solon** | Micronutrients | Placebo | Non-verbal ability | anaemic | 0.00 | -0.49 | 0.49 |
| **Solon** | Micronutrients | Placebo | Non-verbal ability | non-anaemic | -0.24 | -0.88 | 0.41 |
| **Solon** | Micronutrients | Placebo | Total cognition score | anaemic | 0.42 | -0.68 | 1.52 |
| **Solon** | Micronutrients | Placebo | Total cognition score | non-anaemic | 0.07 | -1.36 | 1.50 |

*bolded numbers indiciate statistically significant effects

Table 21: Results – Effect modifiers

**BAZ (≥2)**

| **treatment** | **comparison** | **weight** | **height** | **haemoglobin** |
| --- | --- | --- | --- | --- |
| STH deworming with any drug | Placebo or control | 0.01(-0.08,0.11) | 0.09(-0.11,0.28) | 0.40(-0.56,1.37) |
| Any STH deworming combination with praziquantel | Placebo or control | 0.04(-0.12,0.20) | -0.05(-0.32,0.22) | 1.82(0.44,3.19) |
| Any STH deworming combination with praziquantel with iron or micronutrients | Placebo or control | -0.01(-0.25,0.23) | -0.02(-0.37,0.33) | 2.76(0.98,4.54) |
| Any STH deworming with micronutrients or iron | Placebo or control | -0.02(-0.16,0.13) | 0.02(-0.24,0.29) | 2.23(0.88,3.57) |
| Micronutrients or iron alone | Placebo or control | 0.01(-0.14,0.16) | -0.03(-0.28,0.22) | 1.12(-0.16,2.40) |
| Any STH deworming combination with praziquantel | STH deworming with any drug | 0.03(-0.15,0.20) | -0.14(-0.43,0.15) | 1.41(-0.18,3.01) |
| Any STH deworming combination with praziquantel with iron or micronutrients | STH deworming with any drug | -0.02(-0.28,0.24) | -0.11(-0.46,0.25) | 2.36(0.34,4.37) |
| Any STH deworming with micronutrients or iron | STH deworming with any drug | -0.03(-0.18,0.13) | -0.06(-0.34,0.21) | 1.82(0.35,3.29) |
| Micronutrients or iron alone | STH deworming with any drug | 0.00(-0.16,0.15) | -0.12(-0.37,0.14) | 0.72(-0.72,2.15) |
| Any STH deworming combination with praziquantel with iron or micronutrients | Any STH deworming combination with praziquantel | -0.05(-0.29,0.19) | 0.03(-0.31,0.38) | 0.94(-1.02,2.90) |
| Any STH deworming with micronutrients or iron | Any STH deworming combination with praziquantel | -0.06(-0.26,0.15) | 0.08(-0.27,0.42) | 0.41(-1.45,2.26) |
| Micronutrients or iron alone | Any STH deworming combination with praziquantel | -0.03(-0.22,0.15) | 0.02(-0.28,0.32) | -0.70(-2.27,0.88) |
| Any STH deworming with micronutrients or iron | Any STH deworming combination with praziquantel with iron or micronutrients | -0.01(-0.28,0.27) | 0.04(-0.36,0.45) | -0.54(-2.70,1.63) |
| Micronutrients or iron alone | Any STH deworming combination with praziquantel with iron or micronutrients | 0.02(-0.22,0.26) | -0.01(-0.37,0.34) | -1.64(-3.61,0.33) |
| Micronutrients or iron alone | Any STH deworming with micronutrients or iron | 0.03(-0.15,0.20) | -0.05(-0.34,0.23) | -1.10(-2.63,0.42) |

**BAZ (≤2)**

| **treatment** | **comparison** | **weight** | **height** | **haemoglobin** |
| --- | --- | --- | --- | --- |
| STH deworming with any drug | Placebo or control | -0.02  (-0.24,0.21) | -0.13  (-0.70,0.43) | -0.63  (-2.90,1.64) |
| Any STH deworming combination with praziquantel | Placebo or control | -0.10(-0.62,0.43) | -0.39(-1.17,0.39) | 1.82(-2.70,6.35) |
| Any STH deworming combination with praziquantel with iron or micronutrients | Placebo or control | -0.30(-1.16,0.56) | -0.05(-1.20,1.11) | 2.12(-4.35,8.59) |
| Any STH deworming with micronutrients or iron | Placebo or control | 0.01(-0.22,0.23) | -0.15(-0.67,0.36) | 1.26(-0.91,3.44) |
| Micronutrients or iron alone | Placebo or control | -0.23(-0.61,0.14) | -0.33(-0.94,0.28) | 2.58(-0.61,5.76) |
| Any STH deworming combination with praziquantel | STH deworming with any drug | -0.08(-0.62,0.46) | -0.26(-1.20,0.69) | 2.45(-2.39,7.30) |
| Any STH deworming combination with praziquantel with iron or micronutrients | STH deworming with any drug | -0.28(-1.15,0.59) | 0.09(-1.10,1.27) | 2.75(-3.82,9.33) |
| Any STH deworming with micronutrients or iron | STH deworming with any drug | 0.02(-0.27,0.32) | -0.02(-0.62,0.58) | 1.90(-0.87,4.66) |
| Micronutrients or iron alone | STH deworming with any drug | -0.22(-0.61,0.18) | -0.20(-0.80,0.41) | 3.21(-0.14,6.56) |
| Any STH deworming combination with praziquantel with iron or micronutrients | Any STH deworming combination with praziquantel | -0.20(-1.10,0.70) | 0.34(-0.87,1.56) | 0.30(-6.72,7.32) |
| Any STH deworming with micronutrients or iron | Any STH deworming combination with praziquantel | 0.10(-0.45,0.66) | 0.24(-0.69,1.16) | -0.56(-5.54,4.43) |
| Micronutrients or iron alone | Any STH deworming combination with praziquantel | -0.14(-0.72,0.44) | 0.06(-0.87,0.99) | 0.76(-4.18,5.69) |
| Any STH deworming with micronutrients or iron | Any STH deworming combination with praziquantel with iron or micronutrients | 0.31(-0.54,1.16) | -0.10(-1.32,1.11) | -0.86(-7.51,5.79) |
| Micronutrients or iron alone | Any STH deworming combination with praziquantel with iron or micronutrients | 0.07(-0.77,0.90) | -0.28(-1.47,0.90) | 0.46(-6.10,7.01) |
| Micronutrients or iron alone | Any STH deworming with micronutrients or iron | -0.24(-0.64,0.16) | -0.18(-0.83,0.47) | 1.31(-2.08,4.71) |

**Effect modifier**

**Sex (Female)**

| treatment | comparison | weight | height | haemoglobin |
| --- | --- | --- | --- | --- |
| STH deworming with any drug | Placebo or control | 0.04(-0.08,0.17) | 0.04(-0.18,0.27) | 0.32(-0.86,1.50) |
| Any STH deworming combination with praziquantel | Placebo or control | 0.19(-0.04,0.41) | 0.00(-0.35,0.35) | 1.64(-0.30,3.58) |
| Any STH deworming combination with praziquantel with iron or micronutrients | Placebo or control | -0.21(-0.62,0.19) | -0.06(-0.49,0.38) | 3.11(0.69,5.53) |
| Any STH deworming with micronutrients or iron | Placebo or control | 0.02(-0.16,0.20) | -0.09(-0.39,0.21) | 2.04(0.39,3.69) |
| Micronutrients or iron alone | Placebo or control | -0.04(-0.24,0.16) | -0.13(-0.42,0.16) | 1.50(-0.10,3.10) |
| Any STH deworming combination with praziquantel | STH deworming with any drug | 0.14(-0.11,0.39) | -0.04(-0.42,0.33) | 1.32(-0.85,3.49) |
| Any STH deworming combination with praziquantel with iron or micronutrients | STH deworming with any drug | -0.25(-0.70,0.19) | -0.10(-0.56,0.36) | 2.79(0.11,5.48) |
| Any STH deworming with micronutrients or iron | STH deworming with any drug | -0.02(-0.23,0.18) | -0.13(-0.46,0.19) | 1.72(-0.14,3.57) |
| Micronutrients or iron alone | STH deworming with any drug | -0.09(-0.30,0.13) | -0.18(-0.50,0.15) | 1.18(-0.60,2.96) |
| Any STH deworming combination with praziquantel with iron or micronutrients | Any STH deworming combination with praziquantel | -0.40(-0.81,0.02) | -0.06(-0.51,0.39) | 1.47(-1.20,4.14) |
| Any STH deworming with micronutrients or iron | Any STH deworming combination with praziquantel | -0.17(-0.45,0.11) | -0.09(-0.52,0.34) | 0.40(-1.99,2.78) |
| Micronutrients or iron alone | Any STH deworming combination with praziquantel | -0.23(-0.50,0.04) | -0.13(-0.53,0.26) | -0.14(-2.32,2.03) |
| Any STH deworming with micronutrients or iron | Any STH deworming combination with praziquantel with iron or micronutrients | 0.23(-0.21,0.67) | -0.03(-0.53,0.47) | -1.07(-3.87,1.72) |
| Micronutrients or iron alone | Any STH deworming combination with praziquantel with iron or micronutrients | 0.17(-0.26,0.60) | -0.08(-0.53,0.38) | -1.61(-4.16,0.93) |
| Micronutrients or iron alone | Any STH deworming with micronutrients or iron | -0.06(-0.29,0.17) | -0.04(-0.39,0.30) | -0.54(-2.44,1.36) |

**Sex (Male)**

| treatment | comparison | weight | height | haemoglobin |
| --- | --- | --- | --- | --- |
| STH deworming with any drug | Placebo or control | -0.01(-0.13,0.12) | 0.13(-0.09,0.36) | 0.28(-0.93,1.50) |
| Any STH deworming combination with praziquantel | Placebo or control | -0.08(-0.29,0.13) | -0.15(-0.50,0.21) | 1.96(0.19,3.73) |
| Any STH deworming combination with praziquantel with iron or micronutrients | Placebo or control | 0.13(-0.20,0.46) | -0.05(-0.50,0.40) | 2.46(0.11,4.82) |
| Any STH deworming with micronutrients or iron | Placebo or control | -0.05(-0.22,0.13) | 0.04(-0.30,0.37) | 2.26(0.68,3.84) |
| Micronutrients or iron alone | Placebo or control | 0.00(-0.19,0.19) | -0.01(-0.33,0.31) | 1.20(-0.51,2.91) |
| Any STH deworming combination with praziquantel | STH deworming with any drug | -0.07(-0.29,0.15) | -0.28(-0.66,0.10) | 1.68(-0.47,3.82) |
| Any STH deworming combination with praziquantel with iron or micronutrients | STH deworming with any drug | 0.14(-0.21,0.48) | -0.19(-0.67,0.30) | 2.18(-0.42,4.79) |
| Any STH deworming with micronutrients or iron | STH deworming with any drug | -0.04(-0.23,0.15) | -0.10(-0.45,0.26) | 1.98(0.25,3.71) |
| Micronutrients or iron alone | STH deworming with any drug | 0.01(-0.19,0.21) | -0.14(-0.48,0.19) | 0.92(-1.05,2.89) |
| Any STH deworming combination with praziquantel with iron or micronutrients | Any STH deworming combination with praziquantel | 0.21(-0.10,0.52) | 0.10(-0.38,0.57) | 0.50(-2.00,3.01) |
| Any STH deworming with micronutrients or iron | Any STH deworming combination with praziquantel | 0.03(-0.23,0.29) | 0.18(-0.27,0.63) | 0.30(-2.03,2.63) |
| Micronutrients or iron alone | Any STH deworming combination with praziquantel | 0.08(-0.17,0.33) | 0.14(-0.27,0.54) | -0.76(-2.88,1.36) |
| Any STH deworming with micronutrients or iron | Any STH deworming combination with praziquantel with iron or micronutrients | -0.18(-0.54,0.18) | 0.09(-0.44,0.62) | -0.20(-2.97,2.57) |
| Micronutrients or iron alone | Any STH deworming combination with praziquantel with iron or micronutrients | -0.13(-0.48,0.23) | 0.04(-0.43,0.52) | -1.26(-3.86,1.33) |
| Micronutrients or iron alone | Any STH deworming with micronutrients or iron | 0.05(-0.17,0.27) | -0.05(-0.42,0.33) | -1.06(-3.05,0.93) |

**Effect modifier**

**Age (>5years**)

| treatment | comparison | weight | height | haemoglobin |
| --- | --- | --- | --- | --- |
| STH deworming with any drug | Placebo or control | 0.06(-0.07,0.20) | 0.03(-0.19,0.24) | 0.33(-0.79,1.44) |
| Any STH deworming combination with praziquantel | Placebo or control | 0.11(-0.06,0.28) | -0.08(-0.31,0.15) | 1.93(0.61,3.26) |
| Any STH deworming combination with praziquantel with iron or micronutrients | Placebo or control | 0.05(-0.21,0.30) | -0.05(-0.36,0.25) | 2.83(1.17,4.49) |
| Any STH deworming with micronutrients or iron | Placebo or control | -0.02(-0.17,0.14) | -0.06(-0.30,0.19) | 2.10(0.78,3.41) |
| Micronutrients or iron alone | Placebo or control | 0.04(-0.12,0.20) | -0.08(-0.31,0.15) | 1.33(0.09,2.57) |
| Any STH deworming combination with praziquantel | STH deworming with any drug | 0.05(-0.15,0.25) | -0.10(-0.39,0.18) | 1.61(0.01,3.20) |
| Any STH deworming combination with praziquantel with iron or micronutrients | STH deworming with any drug | -0.02(-0.28,0.25) | -0.08(-0.43,0.27) | 2.50(0.61,4.40) |
| Any STH deworming with micronutrients or iron | STH deworming with any drug | -0.08(-0.26,0.10) | -0.08(-0.34,0.18) | 1.77(0.33,3.20) |
| Micronutrients or iron alone | STH deworming with any drug | -0.02(-0.20,0.16) | -0.11(-0.36,0.15) | 1.00(-0.41,2.41) |
| Any STH deworming combination with praziquantel with iron or micronutrients | Any STH deworming combination with praziquantel | -0.06(-0.32,0.19) | 0.02(-0.29,0.33) | 0.90(-0.93,2.73) |
| Any STH deworming with micronutrients or iron | Any STH deworming combination with praziquantel | -0.13(-0.34,0.09) | 0.02(-0.29,0.33) | 0.16(-1.64,1.96) |
| Micronutrients or iron alone | Any STH deworming combination with praziquantel | -0.07(-0.27,0.13) | 0.00(-0.27,0.27) | -0.61(-2.11,0.90) |
| Any STH deworming with micronutrients or iron | Any STH deworming combination with praziquantel with iron or micronutrients | -0.06(-0.34,0.22) | 0.00(-0.37,0.36) | -0.74(-2.75,1.27) |
| Micronutrients or iron alone | Any STH deworming combination with praziquantel with iron or micronutrients | -0.01(-0.25,0.24) | -0.03(-0.34,0.29) | -1.51(-3.34,0.32) |
| Micronutrients or iron alone | Any STH deworming with micronutrients or iron | 0.06(-0.13,0.24) | -0.03(-0.29,0.24) | -0.77(-2.24,0.71) |

**Age (<5years)**

| treatment | comparison | weight | height | haemoglobin |
| --- | --- | --- | --- | --- |
| STH deworming with any drug | Placebo or control | -0.02(-0.12,0.08) | 0.18(-0.14,0.49) | 0.37(-1.13,1.86) |
| Any STH deworming combination with praziquantel | Placebo or control | 0.08(-4.51,4.67) | -2.04(-9.22,5.13) | 14.19(-46.06,74.45) |
| Any STH deworming combination with praziquantel with iron or micronutrients | Placebo or control | -0.02(-0.26,0.22) | -0.02(-0.78,0.73) | 1.73(-1.99,5.45) |
| Any STH deworming with micronutrients or iron | Placebo or control | -0.18(-0.42,0.05) | -0.07(-0.84,0.70) | 1.79(-2.08,5.65) |
| Micronutrients or iron alone | Placebo or control | 0.10(-4.51,4.71) | -2.22(-9.39,4.95) | 13.83(-45.84,73.49) |
| Any STH deworming combination with praziquantel | STH deworming with any drug | 0.00(-0.23,0.22) | -0.20(-0.94,0.54) | 1.36(-2.38,5.11) |
| Any STH deworming combination with praziquantel with iron or micronutrients | STH deworming with any drug | -0.17(-0.41,0.08) | -0.25(-1.01,0.51) | 1.42(-2.42,5.27) |
| Any STH deworming with micronutrients or iron | STH deworming with any drug | -0.10(-4.73,4.53) | 2.02(-5.17,9.21) | -12.46(-72.59,47.66) |
| Micronutrients or iron alone | STH deworming with any drug | -0.27(-4.83,4.30) | 1.97(-5.28,9.22) | -12.41(-73.03,48.22) |
| Any STH deworming combination with praziquantel with iron or micronutrients | Any STH deworming combination with praziquantel | -0.16(-0.46,0.13) | -0.05(-0.92,0.82) | 0.06(-4.09,4.21) |
| Any STH deworming with micronutrients or iron | Any STH deworming combination with praziquantel | 0.00(0.00,0.00) | 0.00(0.00,0.00) | 0.00(0.00,0.00) |
| Micronutrients or iron alone | Any STH deworming combination with praziquantel | 0.00(0.00,0.00) | 0.00(0.00,0.00) | 0.00(0.00,0.00) |
| Any STH deworming with micronutrients or iron | Any STH deworming combination with praziquantel with iron or micronutrients | 0.00(0.00,0.00) | 0.00(0.00,0.00) | 0.00(0.00,0.00) |
| Micronutrients or iron alone | Any STH deworming combination with praziquantel with iron or micronutrients | 0.00(0.00,0.00) | 0.00(0.00,0.00) | 0.00(0.00,0.00) |
| Micronutrients or iron alone | Any STH deworming with micronutrients or iron | 0.00(0.00,0.00) | 0.000.00,0.00) | 0.00(0.00,0.00) |

**Effect modifier**

***A Lumbricoides*  – no infection**

| treatment | comparison | weight | height | haemoglobin |
| --- | --- | --- | --- | --- |
| STH deworming with any drug | Placebo or control | 0.00(-0.11,0.12) | 0.07(-0.14,0.27) | 0.48(-0.69,1.66) |
| Any STH deworming combination with praziquantel | Placebo or control | 0.10(-0.09,0.29) | -0.10(-0.39,0.18) | 1.86(0.35,3.38) |
| Any STH deworming combination with praziquantel with iron or micronutrients | Placebo or control | 0.05(-0.21,0.31) | -0.03(-0.39,0.33) | 2.50(0.75,4.24) |
| Any STH deworming with micronutrients or iron | Placebo or control | 0.02(-0.16,0.20) | -0.07(-0.40,0.26) | 2.43(0.72,4.15) |
| Micronutrients or iron alone | Placebo or control | -0.04(-0.21,0.14) | -0.12(-0.41,0.17) | 1.19(-0.24,2.61) |
| Any STH deworming combination with praziquantel | STH deworming with any drug | 0.09(-0.12,0.31) | -0.17(-0.50,0.16) | 1.38(-0.50,3.26) |
| Any STH deworming combination with praziquantel with iron or micronutrients | STH deworming with any drug | 0.05(-0.23,0.32) | -0.09(-0.48,0.29) | 2.01(0.05,3.98) |
| Any STH deworming with micronutrients or iron | STH deworming with any drug | 0.02(-0.18,0.22) | -0.14(-0.49,0.21) | 1.95(0.00,3.90) |
| Micronutrients or iron alone | STH deworming with any drug | -0.04(-0.24,0.16) | -0.19(-0.50,0.13) | 0.70(-1.06,2.47) |
| Any STH deworming combination with praziquantel with iron or micronutrients | Any STH deworming combination with praziquantel | -0.05(-0.30,0.20) | 0.08(-0.29,0.44) | 0.63(-1.32,2.58) |
| Any STH deworming with micronutrients or iron | Any STH deworming combination with praziquantel | -0.08(-0.32,0.17) | 0.03(-0.38,0.45) | 0.57(-1.55,2.70) |
| Micronutrients or iron alone | Any STH deworming combination with praziquantel | -0.13(-0.35,0.08) | -0.02(-0.35,0.32) | -0.68(-2.36,1.00) |
| Any STH deworming with micronutrients or iron | Any STH deworming combination with praziquantel with iron or micronutrients | -0.03(-0.33,0.27) | -0.04(-0.51,0.42) | -0.06(-2.45,2.33) |
| Micronutrients or iron alone | Any STH deworming combination with praziquantel with iron or micronutrients | -0.08(-0.34,0.17) | -0.09(-0.47,0.29) | -1.31(-3.24,0.62) |
| Micronutrients or iron alone | Any STH deworming with micronutrients or iron | -0.06(-0.28,0.16) | -0.05(-0.42,0.33) | -1.25(-3.15,0.65) |

***A Lumbricoides*  – lighter infection**

| treatment | comparison | weight | height | haemoglobin |
| --- | --- | --- | --- | --- |
| STH deworming with any drug | Placebo or control | 0.07(-0.13,0.27) | 0.25(-0.14,0.63) | -0.10(-1.75,1.55) |
| Any STH deworming combination with praziquantel | Placebo or control | -0.10(-0.82,0.61) | -0.19(-1.36,0.97) | 1.89(-3.64,7.43) |
| Any STH deworming combination with praziquantel with iron or micronutrients | Placebo or control | -0.69(-1.58,0.19) | -0.82(-2.32,0.68) | 1.69(-6.67,10.06) |
| Any STH deworming with micronutrients or iron | Placebo or control | -0.19(-0.48,0.10) | -0.02(-0.55,0.50) | 1.70(-0.86,4.26) |
| Micronutrients or iron alone | Placebo or control | 0.00(-0.31,0.31) | 0.01(-0.55,0.57) | 2.50(-0.48,5.48) |
| Any STH deworming combination with praziquantel | STH deworming with any drug | -0.17(-0.89,0.55) | -0.44(-1.57,0.69) | 1.99(-3.48,7.46) |
| Any STH deworming combination with praziquantel with iron or micronutrients | STH deworming with any drug | -0.76(-1.68,0.16) | -1.07(-2.61,0.48) | 1.80(-6.59,10.18) |
| Any STH deworming with micronutrients or iron | STH deworming with any drug | -0.26(-0.56,0.04) | -0.27(-0.83,0.28) | 1.80(-0.76,4.36) |
| Micronutrients or iron alone | STH deworming with any drug | -0.07(-0.40,0.27) | -0.23(-0.82,0.35) | 2.60(-0.28,5.48) |
| Any STH deworming combination with praziquantel with iron or micronutrients | Any STH deworming combination with praziquantel | -0.59(-1.53,0.35) | -0.63(-2.36,1.11) | -0.20(-8.50,8.11) |
| Any STH deworming with micronutrients or iron | Any STH deworming combination with praziquantel | -0.08(-0.85,0.69) | 0.17(-1.05,1.39) | -0.19(-6.05,5.66) |
| Micronutrients or iron alone | Any STH deworming combination with praziquantel | 0.11(-0.63,0.84) | 0.21(-1.00,1.42) | 0.61(-5.20,6.42) |
| Any STH deworming with micronutrients or iron | Any STH deworming combination with praziquantel with iron or micronutrients | 0.51(-0.44,1.45) | 0.80(-0.76,2.35) | 0.01(-8.74,8.75) |
| Micronutrients or iron alone | Any STH deworming combination with praziquantel with iron or micronutrients | 0.70(-0.18,1.57) | 0.83(-0.68,2.35) | 0.80(-7.67,9.28) |
| Micronutrients or iron alone | Any STH deworming with micronutrients or iron | 0.19(-0.16,0.54) | 0.04(-0.57,0.65) | 0.80(-2.60,4.20) |

***A Lumbricoides*  – high infection**

| treatment | comparison | weight | height | haemoglobin |
| --- | --- | --- | --- | --- |
| STH deworming with any drug | Placebo or control | 0.08(-0.13,0.29) | 0.04(-0.22,0.30) | 0.03(-2.08,2.14) |
| Any STH deworming combination with praziquantel | Placebo or control | -0.17(-0.53,0.19) | 0.00(-0.45,0.45) | 1.73(-1.41,4.87) |
| Any STH deworming combination with praziquantel with iron or micronutrients | Placebo or control | -0.52(-1.34,0.30) | -0.40(-1.43,0.63) | 7.37(-0.24,14.98) |
| Any STH deworming with micronutrients or iron | Placebo or control | 0.06(-0.18,0.30) | -0.06(-0.36,0.24) | 2.85(0.49,5.21) |
| Micronutrients or iron alone | Placebo or control | 0.04(-0.26,0.34) | -0.17(-0.55,0.20) | 0.84(-1.74,3.43) |
| Any STH deworming combination with praziquantel | STH deworming with any drug | -0.25(-0.67,0.16) | -0.04(-0.54,0.46) | 1.70(-2.09,5.49) |
| Any STH deworming combination with praziquantel with iron or micronutrients | STH deworming with any drug | -0.60(-1.39,0.20) | -0.44(-1.53,0.65) | 7.34(-0.41,15.09) |
| Any STH deworming with micronutrients or iron | STH deworming with any drug | -0.02(-0.27,0.23) | -0.10(-0.45,0.24) | 2.82(0.44,5.20) |
| Micronutrients or iron alone | STH deworming with any drug | -0.04(-0.33,0.25) | -0.22(-0.62,0.19) | 0.81(-2.02,3.65) |
| Any STH deworming combination with praziquantel with iron or micronutrients | Any STH deworming combination with praziquantel | -0.34(-1.15,0.46) | -0.40(-1.44,0.65) | 5.64(-2.64,13.93) |
| Any STH deworming with micronutrients or iron | Any STH deworming combination with praziquantel | 0.23(-0.17,0.63) | -0.06(-0.59,0.47) | 1.12(-2.82,5.07) |
| Micronutrients or iron alone | Any STH deworming combination with praziquantel | 0.21(-0.22,0.64) | -0.17(-0.73,0.38) | -0.88(-4.80,3.03) |
| Any STH deworming with micronutrients or iron | Any STH deworming combination with praziquantel with iron or micronutrients | 0.58(-0.23,1.39) | 0.34(-0.72,1.39) | -4.52(-12.36,3.32) |
| Micronutrients or iron alone | Any STH deworming combination with praziquantel with iron or micronutrients | 0.56(-0.26,1.37) | 0.23(-0.82,1.27) | -6.53(-14.40,1.35) |
| Micronutrients or iron alone | Any STH deworming with micronutrients or iron | -0.02(-0.30,0.26) | -0.11(-0.51,0.29) | -2.01(-4.86,0.84) |

**Effect modifier**

**Hookworm – no infection**

| treatment | comparison | weight | height | haemoglobin |
| --- | --- | --- | --- | --- |
| STH deworming with any drug | Placebo or control | 0.02(-0.09,0.13) | 0.06(-0.13,0.26) | 0.07(-0.92,1.06) |
| Any STH deworming combination with praziquantel | Placebo or control | 0.00(-0.34,0.33) | -0.13(-0.61,0.35) | 0.59(-1.72,2.89) |
| Any STH deworming combination with praziquantel with iron or micronutrients | Placebo or control | -0.04(-0.41,0.33) | -0.14(-0.67,0.39) | 1.98(-0.66,4.63) |
| Any STH deworming with micronutrients or iron | Placebo or control | -0.04(-0.19,0.11) | -0.09(-0.37,0.18) | 1.94(0.59,3.29) |
| Micronutrients or iron alone | Placebo or control | 0.00(-0.16,0.17) | -0.10(-0.38,0.18) | 0.80(-0.56,2.16) |
| Any STH deworming combination with praziquantel | STH deworming with any drug | -0.02(-0.38,0.33) | -0.20(-0.70,0.31) | 0.52(-1.94,2.98) |
| Any STH deworming combination with praziquantel with iron or micronutrients | STH deworming with any drug | -0.06(-0.45,0.33) | -0.21(-0.75,0.34) | 1.92(-0.74,4.58) |
| Any STH deworming with micronutrients or iron | STH deworming with any drug | -0.06(-0.22,0.10) | -0.16(-0.45,0.13) | 1.88(0.45,3.30) |
| Micronutrients or iron alone | STH deworming with any drug | -0.02(-0.19,0.15) | -0.17(-0.47,0.14) | 0.74(-0.72,2.19) |
| Any STH deworming combination with praziquantel with iron or micronutrients | Any STH deworming combination with praziquantel | -0.03(-0.39,0.33) | -0.01(-0.58,0.56) | 1.40(-1.44,4.24) |
| Any STH deworming with micronutrients or iron | Any STH deworming combination with praziquantel | -0.03(-0.40,0.33) | 0.04(-0.49,0.57) | 1.36(-1.21,3.92) |
| Micronutrients or iron alone | Any STH deworming combination with praziquantel | 0.01(-0.35,0.37) | 0.03(-0.47,0.53) | 0.22(-2.23,2.67) |
| Any STH deworming with micronutrients or iron | Any STH deworming combination with praziquantel with iron or micronutrients | 0.00(-0.40,0.40) | 0.05(-0.52,0.61) | -0.04(-2.93,2.84) |
| Micronutrients or iron alone | Any STH deworming combination with praziquantel with iron or micronutrients | 0.04(-0.35,0.43) | 0.04(-0.50,0.58) | -1.18(-3.93,1.56) |
| Micronutrients or iron alone | Any STH deworming with micronutrients or iron | 0.04(-0.14,0.22) | -0.01(-0.33,0.31) | -1.14(-2.74,0.46) |

**Hookworm – lighter infection**

| treatment | comparison | weight | height | haemoglobin |
| --- | --- | --- | --- | --- |
| STH deworming with any drug | Placebo or control | 0.05(-0.12,0.23) | 0.20(-0.16,0.55) | -0.04(-1.84,1.77) |
| Any STH deworming combination with praziquantel | Placebo or control | 0.03(-0.22,0.27) | -0.12(-0.49,0.24) | 2.06(-0.34,4.46) |
| Any STH deworming combination with praziquantel with iron or micronutrients | Placebo or control | -0.12(-0.44,0.20) | 0.02(-0.44,0.47) | 2.75(-0.01,5.51) |
| Any STH deworming with micronutrients or iron | Placebo or control | 0.10(-0.19,0.40) | 0.15(-0.38,0.68) | 1.43(-1.42,4.28) |
| Micronutrients or iron alone | Placebo or control | -0.05(-0.30,0.20) | -0.03(-0.42,0.35) | 1.76(-0.56,4.08) |
| Any STH deworming combination with praziquantel | STH deworming with any drug | -0.03(-0.30,0.25) | -0.32(-0.74,0.10) | 2.10(-0.57,4.76) |
| Any STH deworming combination with praziquantel with iron or micronutrients | STH deworming with any drug | -0.17(-0.53,0.19) | -0.18(-0.71,0.35) | 2.78(-0.36,5.93) |
| Any STH deworming with micronutrients or iron | STH deworming with any drug | 0.05(-0.26,0.36) | -0.05(-0.58,0.48) | 1.46(-1.55,4.48) |
| Micronutrients or iron alone | STH deworming with any drug | -0.10(-0.39,0.19) | -0.23(-0.63,0.17) | 1.80(-0.69,4.29) |
| Any STH deworming combination with praziquantel with iron or micronutrients | Any STH deworming combination with praziquantel | -0.15(-0.47,0.18) | 0.14(-0.32,0.60) | 0.69(-2.08,3.46) |
| Any STH deworming with micronutrients or iron | Any STH deworming combination with praziquantel | 0.07(-0.28,0.43) | 0.27(-0.32,0.87) | -0.63(-4.00,2.74) |
| Micronutrients or iron alone | Any STH deworming combination with praziquantel | -0.08(-0.37,0.22) | 0.09(-0.32,0.49) | -0.30(-2.77,2.17) |
| Any STH deworming with micronutrients or iron | Any STH deworming combination with praziquantel with iron or micronutrients | 0.22(-0.19,0.63) | 0.13(-0.55,0.82) | -1.32(-4.83,2.19) |
| Micronutrients or iron alone | Any STH deworming combination with praziquantel with iron or micronutrients | 0.07(-0.23,0.37) | -0.05(-0.52,0.42) | -0.99(-3.77,1.80) |
| Micronutrients or iron alone | Any STH deworming with micronutrients or iron | -0.15(-0.49,0.19) | -0.18(-0.73,0.36) | 0.33(-2.64,3.31) |

**Hookworm – high infection**

| Treatment | comparison | weight | height | haemoglobin |
| --- | --- | --- | --- | --- |
| STH deworming with any drug | Placebo or control | 0.16(-0.13,0.46) | 0.20(-0.13,0.52) | 3.58(0.13,7.02) |
| Any STH deworming combination with praziquantel | Placebo or control | 0.05(-0.27,0.37) | 0.00(-0.43,0.43) | 2.81(0.32,5.30) |
| Any STH deworming combination with praziquantel with iron or micronutrients | Placebo or control | 0.23(-0.51,0.98) | -0.16(-1.01,0.68) | 4.63(-0.30,9.56) |
| Any STH deworming with micronutrients or iron | Placebo or control | 0.09(-0.55,0.73) | -0.12(-1.02,0.77) | 5.46(-0.21,11.14) |
| Micronutrients or iron alone | Placebo or control | -0.04(-0.80,0.71) | -0.36(-1.35,0.63) | 2.05(-3.86,7.96) |
| Any STH deworming combination with praziquantel | STH deworming with any drug | -0.11(-0.51,0.29) | -0.20(-0.71,0.32) | -0.77(-4.59,3.05) |
| Any STH deworming combination with praziquantel with iron or micronutrients | STH deworming with any drug | 0.07(-0.69,0.84) | -0.36(-1.27,0.55) | 1.06(-4.76,6.87) |
| Any STH deworming with micronutrients or iron | STH deworming with any drug | -0.08(-0.77,0.62) | -0.32(-1.25,0.61) | 1.89(-4.37,8.14) |
| Micronutrients or iron alone | STH deworming with any drug | -0.20(-1.00,0.59) | -0.55(-1.56,0.45) | -1.52(-8.00,4.95) |
| Any STH deworming combination with praziquantel with iron or micronutrients | Any STH deworming combination with praziquantel | 0.19(-0.53,0.90) | -0.16(-1.03,0.70) | 1.82(-3.18,6.83) |
| Any STH deworming with micronutrients or iron | Any STH deworming combination with praziquantel | 0.04(-0.67,0.75) | -0.12(-1.08,0.84) | 2.66(-3.42,8.74) |
| Micronutrients or iron alone | Any STH deworming combination with praziquantel | -0.09(-0.84,0.66) | -0.36(-1.39,0.67) | -0.76(-6.70,5.19) |
| Any STH deworming with micronutrients or iron | Any STH deworming combination with praziquantel with iron or micronutrients | -0.15(-1.22,0.93) | 0.04(-1.20,1.28) | 0.83(-6.88,8.54) |
| Micronutrients or iron alone | Any STH deworming combination with praziquantel with iron or micronutrients | -0.28(-1.28,0.73) | -0.19(-1.44,1.05) | -2.58(-9.34,4.18) |
| Micronutrients or iron alone | Any STH deworming with micronutrients or iron | -0.13(-1.01,0.75) | -0.23(-1.51,1.05) | -3.41(-11.23,4.41) |

**Effect Modifier**

***T. Trichiura*– no infection**

| treatment | comparison | weight | height | haemoglobin |
| --- | --- | --- | --- | --- |
| STH deworming with any drug | Placebo or control | -0.01(-0.12,0.10) | 0.02(-0.18,0.22) | 0.17(-1.01,1.35) |
| Any STH deworming combination with praziquantel | Placebo or control | 0.03(-0.20,0.25) | -0.26(-0.62,0.11) | 1.15(-0.68,2.98) |
| Any STH deworming combination with praziquantel with iron or micronutrients | Placebo or control | 0.09(-0.19,0.36) | -0.08(-0.48,0.33) | 1.27(-0.82,3.36) |
| Any STH deworming with micronutrients or iron | Placebo or control | -0.04(-0.22,0.13) | -0.12(-0.44,0.20) | 1.85(0.23,3.46) |
| Micronutrients or iron alone | Placebo or control | -0.09(-0.26,0.09) | -0.17(-0.47,0.13) | 0.37(-1.11,1.85) |
| Any STH deworming combination with praziquantel | STH deworming with any drug | 0.04(-0.22,0.29) | -0.28(-0.68,0.12) | 0.98(-1.08,3.03) |
| Any STH deworming combination with praziquantel with iron or micronutrients | STH deworming with any drug | 0.10(-0.19,0.38) | -0.10(-0.54,0.34) | 1.10(-1.18,3.37) |
| Any STH deworming with micronutrients or iron | STH deworming with any drug | -0.03(-0.22,0.16) | -0.14(-0.48,0.20) | 1.67(-0.11,3.46) |
| Micronutrients or iron alone | STH deworming with any drug | -0.08(-0.27,0.12) | -0.19(-0.52,0.13) | 0.19(-1.57,1.96) |
| Any STH deworming combination with praziquantel with iron or micronutrients | Any STH deworming combination with praziquantel | 0.06(-0.21,0.33) | 0.18(-0.25,0.61) | 0.12(-2.13,2.37) |
| Any STH deworming with micronutrients or iron | Any STH deworming combination with praziquantel | -0.07(-0.35,0.21) | 0.14(-0.33,0.60) | 0.70(-1.71,3.10) |
| Micronutrients or iron alone | Any STH deworming combination with praziquantel | -0.11(-0.35,0.13) | 0.09(-0.32,0.49) | -0.78(-2.78,1.22) |
| Any STH deworming with micronutrients or iron | Any STH deworming combination with praziquantel with iron or micronutrients | -0.13(-0.44,0.18) | -0.05(-0.55,0.46) | 0.58(-1.98,3.14) |
| Micronutrients or iron alone | Any STH deworming combination with praziquantel with iron or micronutrients | -0.17(-0.45,0.10) | -0.10(-0.52,0.33) | -0.90(-3.12,1.32) |
| Micronutrients or iron alone | Any STH deworming with micronutrients or iron | -0.04(-0.26,0.18) | -0.05(-0.43,0.32) | -1.48(-3.42,0.46) |

***T. Trichiura*– lighter infection**

| treatment | comparison | weight | height | haemoglobin |
| --- | --- | --- | --- | --- |
| STH deworming with any drug | Placebo or control | 0.04(-0.11,0.20) | 0.30(-0.04,0.64) | 0.24(-1.23,1.71) |
| Any STH deworming combination with praziquantel | Placebo or control | 0.00(-0.26,0.26) | 0.18(-0.26,0.62) | 1.97(-0.12,4.06) |
| Any STH deworming combination with praziquantel with iron or micronutrients | Placebo or control | -0.33(-0.74,0.08) | -0.21(-0.83,0.41) | 4.23(1.20,7.25) |
| Any STH deworming with micronutrients or iron | Placebo or control | 0.06(-0.17,0.30) | 0.09(-0.34,0.53) | 2.05(0.00,4.10) |
| Micronutrients or iron alone | Placebo or control | 0.11(-0.12,0.35) | 0.08(-0.33,0.49) | 2.10(-0.10,4.31) |
| Any STH deworming combination with praziquantel | STH deworming with any drug | -0.05(-0.33,0.23) | -0.12(-0.58,0.34) | 1.73(-0.57,4.04) |
| Any STH deworming combination with praziquantel with iron or micronutrients | STH deworming with any drug | -0.38(-0.83,0.08) | -0.51(-1.17,0.14) | 3.99(0.46,7.52) |
| Any STH deworming with micronutrients or iron | STH deworming with any drug | 0.02(-0.22,0.26) | -0.21(-0.65,0.23) | 1.81(-0.23,3.85) |
| Micronutrients or iron alone | STH deworming with any drug | 0.07(-0.18,0.31) | -0.22(-0.62,0.19) | 1.87(-0.15,3.88) |
| Any STH deworming combination with praziquantel with iron or micronutrients | Any STH deworming combination with praziquantel | -0.33(-0.79,0.13) | -0.39(-1.03,0.24) | 2.26(-1.00,5.51) |
| Any STH deworming with micronutrients or iron | Any STH deworming combination with praziquantel | 0.07(-0.27,0.41) | -0.09(-0.64,0.47) | 0.08(-2.59,2.74) |
| Micronutrients or iron alone | Any STH deworming combination with praziquantel | 0.11(-0.20,0.43) | -0.10(-0.58,0.39) | 0.13(-2.33,2.60) |
| Any STH deworming with micronutrients or iron | Any STH deworming combination with praziquantel with iron or micronutrients | 0.40(-0.08,0.87) | 0.31(-0.42,1.04) | -2.18(-5.87,1.51) |
| Micronutrients or iron alone | Any STH deworming combination with praziquantel with iron or micronutrients | 0.44(0.03,0.86) | 0.30(-0.37,0.96) | -2.12(-5.86,1.61) |
| Micronutrients or iron alone | Any STH deworming with micronutrients or iron | 0.05(-0.22,0.31) | -0.01(-0.46,0.44) | 0.05(-2.15,2.26) |

***T. Trichiura*– high infection**

| treatment | comparison | weight | height | haemoglobin |
| --- | --- | --- | --- | --- |
| STH deworming with any drug | Placebo or control | 0.17(-0.06,0.41) | 0.07(-0.20,0.34) | 1.33(-1.14,3.81) |
| Any STH deworming combination with praziquantel | Placebo or control | 0.15(-0.24,0.54) | -0.11(-0.64,0.43) | 3.05(-0.48,6.59) |
| Any STH deworming combination with praziquantel with iron or micronutrients | Placebo or control | 0.23(-0.66,1.13) | 0.27(-0.95,1.50) | 6.76(-1.27,14.80) |
| Any STH deworming with micronutrients or iron | Placebo or control | 0.09(-0.30,0.48) | -0.06(-0.57,0.46) | 3.57(0.12,7.02) |
| Micronutrients or iron alone | Placebo or control | -0.04(-0.46,0.39) | -0.21(-0.79,0.38) | 3.12(-0.67,6.91) |
| Any STH deworming combination with praziquantel | STH deworming with any drug | -0.02(-0.45,0.41) | -0.18(-0.75,0.40) | 1.72(-2.42,5.86) |
| Any STH deworming combination with praziquantel with iron or micronutrients | STH deworming with any drug | 0.06(-0.85,0.98) | 0.21(-1.04,1.45) | 5.43(-2.89,13.75) |
| Any STH deworming with micronutrients or iron | STH deworming with any drug | -0.08(-0.48,0.31) | -0.12(-0.67,0.42) | 2.24(-1.27,5.75) |
| Micronutrients or iron alone | STH deworming with any drug | -0.21(-0.64,0.22) | -0.27(-0.87,0.33) | 1.79(-2.10,5.67) |
| Any STH deworming combination with praziquantel with iron or micronutrients | Any STH deworming combination with praziquantel | 0.08(-0.85,1.02) | 0.38(-0.84,1.60) | 3.71(-4.26,11.68) |
| Any STH deworming with micronutrients or iron | Any STH deworming combination with praziquantel | -0.06(-0.59,0.47) | 0.05(-0.67,0.77) | 0.52(-4.45,5.49) |
| Micronutrients or iron alone | Any STH deworming combination with praziquantel | -0.19(-0.75,0.37) | -0.10(-0.86,0.66) | 0.07(-5.01,5.14) |
| Any STH deworming with micronutrients or iron | Any STH deworming combination with praziquantel with iron or micronutrients | -0.14(-1.11,0.83) | -0.33(-1.63,0.97) | -3.19(-11.92,5.54) |
| Micronutrients or iron alone | Any STH deworming combination with praziquantel with iron or micronutrients | -0.27(-1.25,0.71) | -0.48(-1.78,0.82) | -3.64(-12.45,5.16) |
| Micronutrients or iron alone | Any STH deworming with micronutrients or iron | -0.13(-0.58,0.32) | -0.15(-0.78,0.48) | -0.45(-4.40,3.49) |

**Effect modifier**

**Anyworm – no infection**

| treatment | comparison | weight | height | haemoglobin |
| --- | --- | --- | --- | --- |
| STH deworming with any drug | Placebo or control | 0.02(-0.10,0.15) | 0.06(-0.16,0.29) | 0.21(-1.08,1.51) |
| Any STH deworming combination with praziquantel | Placebo or control | 0.07(-0.44,0.57) | -0.27(-0.94,0.40) | 0.78(-2.50,4.07) |
| Any STH deworming combination with praziquantel with iron or micronutrients | Placebo or control | 0.10(-0.38,0.58) | 0.01(-0.68,0.71) | 1.48(-1.96,4.91) |
| Any STH deworming with micronutrients or iron | Placebo or control | 0.06(-0.16,0.27) | -0.13(-0.53,0.26) | 2.75(0.68,4.82) |
| Micronutrients or iron alone | Placebo or control | 0.09(-0.16,0.34) | -0.14(-0.58,0.30) | 0.24(-1.94,2.42) |
| Any STH deworming combination with praziquantel | STH deworming with any drug | 0.04(-0.49,0.58) | -0.34(-1.03,0.36) | 0.57(-2.78,3.92) |
| Any STH deworming combination with praziquantel with iron or micronutrients | STH deworming with any drug | 0.08(-0.43,0.58) | -0.05(-0.76,0.66) | 1.26(-2.21,4.74) |
| Any STH deworming with micronutrients or iron | STH deworming with any drug | 0.04(-0.20,0.27) | -0.19(-0.62,0.23) | 2.54(0.23,4.84) |
| Micronutrients or iron alone | STH deworming with any drug | 0.07(-0.20,0.33) | -0.20(-0.66,0.26) | 0.02(-2.34,2.39) |
| Any STH deworming combination with praziquantel with iron or micronutrients | Any STH deworming combination with praziquantel | 0.03(-0.40,0.47) | 0.29(-0.49,1.06) | 0.69(-2.98,4.36) |
| Any STH deworming with micronutrients or iron | Any STH deworming combination with praziquantel | -0.01(-0.53,0.51) | 0.14(-0.61,0.89) | 1.97(-1.78,5.72) |
| Micronutrients or iron alone | Any STH deworming combination with praziquantel | 0.02(-0.47,0.51) | 0.13(-0.55,0.82) | -0.55(-3.94,2.84) |
| Any STH deworming with micronutrients or iron | Any STH deworming combination with praziquantel with iron or micronutrients | -0.04(-0.55,0.47) | -0.15(-0.92,0.63) | 1.27(-2.76,5.31) |
| Micronutrients or iron alone | Any STH deworming combination with praziquantel with iron or micronutrients | -0.01(-0.48,0.46) | -0.15(-0.86,0.56) | -1.24(-4.66,2.18) |
| Micronutrients or iron alone | Any STH deworming with micronutrients or iron | 0.03(-0.26,0.32) | -0.01(-0.52,0.50) | -2.51(-5.25,0.23) |

**Anyworm – lighter infection**

| treatment | comparison | weight | height | haemoglobin |
| --- | --- | --- | --- | --- |
| STH deworming with any drug | Placebo or control | 0.01(-0.14,0.15) | 0.16(-0.13,0.44) | 0.63(-0.63,1.89) |
| Any STH deworming combination with praziquantel | Placebo or control | 0.04(-0.17,0.24) | -0.04(-0.36,0.29) | 1.66(-0.06,3.39) |
| Any STH deworming combination with praziquantel with iron or micronutrients | Placebo or control | -0.06(-0.34,0.22) | -0.10(-0.51,0.30) | 2.28(0.22,4.34) |
| Any STH deworming with micronutrients or iron | Placebo or control | -0.10(-0.31,0.11) | -0.01(-0.36,0.34) | 1.78(0.14,3.42) |
| Micronutrients or iron alone | Placebo or control | -0.08(-0.27,0.11) | -0.07(-0.38,0.25) | 1.77(0.05,3.49) |
| Any STH deworming combination with praziquantel | STH deworming with any drug | 0.03(-0.20,0.26) | -0.19(-0.56,0.18) | 1.04(-0.85,2.92) |
| Any STH deworming combination with praziquantel with iron or micronutrients | STH deworming with any drug | -0.07(-0.38,0.24) | -0.26(-0.73,0.21) | 1.65(-0.67,3.97) |
| Any STH deworming with micronutrients or iron | STH deworming with any drug | -0.11(-0.32,0.11) | -0.17(-0.55,0.21) | 1.15(-0.63,2.93) |
| Micronutrients or iron alone | STH deworming with any drug | -0.09(-0.30,0.12) | -0.23(-0.56,0.11) | 1.14(-0.60,2.89) |
| Any STH deworming combination with praziquantel with iron or micronutrients | Any STH deworming combination with praziquantel | -0.10(-0.37,0.17) | -0.06(-0.48,0.35) | 0.62(-1.60,2.84) |
| Any STH deworming with micronutrients or iron | Any STH deworming combination with praziquantel | -0.14(-0.41,0.14) | 0.03(-0.42,0.47) | 0.12(-2.16,2.39) |
| Micronutrients or iron alone | Any STH deworming combination with praziquantel | -0.12(-0.36,0.12) | -0.03(-0.40,0.34) | 0.11(-1.75,1.97) |
| Any STH deworming with micronutrients or iron | Any STH deworming combination with praziquantel with iron or micronutrients | -0.04(-0.38,0.30) | 0.09(-0.43,0.61) | -0.50(-2.95,1.95) |
| Micronutrients or iron alone | Any STH deworming combination with praziquantel with iron or micronutrients | -0.02(-0.30,0.26) | 0.03(-0.41,0.48) | -0.51(-2.77,1.75) |
| Micronutrients or iron alone | Any STH deworming with micronutrients or iron | 0.02(-0.22,0.26) | -0.06(-0.43,0.32) | -0.01(-2.00,1.98) |

**Anyworm – high infection**

| treatment | comparison | weight | height | haemoglobin |
| --- | --- | --- | --- | --- |
| STH deworming with any drug | Placebo or control | 0.12(-0.08,0.32) | 0.02(-0.21,0.26) | -0.55(-2.89,1.80) |
| Any STH deworming combination with praziquantel | Placebo or control | -0.05(-0.41,0.32) | -0.06(-0.50,0.38) | 3.08(0.12,6.05) |
| Any STH deworming combination with praziquantel with iron or micronutrients | Placebo or control | -0.28(-1.12,0.55) | -0.12(-1.11,0.88) | 3.63(0.02,7.25) |
| Any STH deworming with micronutrients or iron | Placebo or control | 0.05(-0.25,0.36) | -0.05(-0.41,0.31) | 9.21(2.89,15.53) |
| Micronutrients or iron alone | Placebo or control | 0.07(-0.30,0.44) | -0.14(-0.59,0.31) | 9.76(3.15,16.37) |
| Any STH deworming combination with praziquantel | STH deworming with any drug | -0.16(-0.58,0.25) | -0.08(-0.56,0.40) | 6.13(-0.51,12.76) |
| Any STH deworming combination with praziquantel with iron or micronutrients | STH deworming with any drug | -0.40(-1.24,0.44) | -0.14(-1.14,0.86) | 2.43(-0.24,5.10) |
| Any STH deworming with micronutrients or iron | STH deworming with any drug | -0.06(-0.39,0.26) | -0.07(-0.46,0.32) | 2.98(0.09,5.87) |
| Micronutrients or iron alone | STH deworming with any drug | -0.05(-0.42,0.33) | -0.16(-0.63,0.31) | -0.65(-4.62,3.32) |
| Any STH deworming combination with praziquantel with iron or micronutrients | Any STH deworming combination with praziquantel | -0.24(-1.12,0.65) | -0.06(-1.07,0.95) | -6.78(-13.60,0.04) |
| Any STH deworming with micronutrients or iron | Any STH deworming combination with praziquantel | 0.10(-0.37,0.57) | 0.01(-0.55,0.57) | 0.83(-2.26,3.92) |
| Micronutrients or iron alone | Any STH deworming combination with praziquantel | 0.12(-0.41,0.64) | -0.08(-0.69,0.53) | 1.38(-1.82,4.58) |
| Any STH deworming with micronutrients or iron | Any STH deworming combination with praziquantel with iron or micronutrients | 0.34(-0.60,1.28) | 0.07(-0.99,1.13) | -2.25(-6.42,1.92) |
| Micronutrients or iron alone | Any STH deworming combination with praziquantel with iron or micronutrients | 0.35(-0.59,1.29) | -0.02(-1.09,1.04) | -8.38(-15.13,-1.63) |
| Micronutrients or iron alone | Any STH deworming with micronutrients or iron | 0.02(-0.33,0.36) | -0.09(-0.56,0.38) | -1.60(-4.74,1.54) |

**Effect modifier**

**Anaemic**

| treatment | comparison | weight | height | haemoglobin |
| --- | --- | --- | --- | --- |
| STH deworming with any drug | Placebo or control | -0.01(-0.12,0.11) | 0.08(-0.16,0.32) | 0.66(-0.67,1.98) |
| Any STH deworming combination with praziquantel | Placebo or control | 0.07(-0.12,0.27) | -0.15(-0.48,0.19) | 2.66(0.88,4.45) |
| Any STH deworming combination with praziquantel with iron or micronutrients | Placebo or control | -0.05(-0.36,0.27) | -0.05(-0.51,0.41) | 3.57(1.03,6.12) |
| Any STH deworming with micronutrients or iron | Placebo or control | -0.01(-0.19,0.17) | -0.07(-0.39,0.25) | 2.54(0.82,4.25) |
| Micronutrients or iron alone | Placebo or control | -0.04(-0.24,0.15) | -0.15(-0.46,0.16) | 1.80(0.03,3.57) |
| Any STH deworming combination with praziquantel | STH deworming with any drug | 0.08(-0.13,0.30) | -0.23(-0.58,0.13) | 2.01(-0.14,4.15) |
| Any STH deworming combination with praziquantel with iron or micronutrients | STH deworming with any drug | -0.04(-0.36,0.29) | -0.13(-0.61,0.34) | 2.92(0.21,5.63) |
| Any STH deworming with micronutrients or iron | STH deworming with any drug | 0.00(-0.19,0.19) | -0.16(-0.50,0.18) | 1.88(0.07,3.69) |
| Micronutrients or iron alone | STH deworming with any drug | -0.03(-0.23,0.16) | -0.23(-0.55,0.09) | 1.15(-0.76,3.06) |
| Any STH deworming combination with praziquantel with iron or micronutrients | Any STH deworming combination with praziquantel | -0.12(-0.44,0.20) | 0.09(-0.36,0.55) | 0.91(-1.82,3.64) |
| Any STH deworming with micronutrients or iron | Any STH deworming combination with praziquantel | -0.08(-0.33,0.17) | 0.07(-0.35,0.50) | -0.13(-2.56,2.31) |
| Micronutrients or iron alone | Any STH deworming combination with praziquantel | -0.12(-0.37,0.13) | 0.00(-0.40,0.39) | -0.86(-3.15,1.43) |
| Any STH deworming with micronutrients or iron | Any STH deworming combination with praziquantel with iron or micronutrients | 0.04(-0.30,0.38) | -0.02(-0.54,0.49) | -1.04(-3.94,1.86) |
| Micronutrients or iron alone | Any STH deworming combination with praziquantel with iron or micronutrients | 0.00(-0.30,0.31) | -0.10(-0.57,0.37) | -1.77(-4.67,1.13) |
| Micronutrients or iron alone | Any STH deworming with micronutrients or iron | -0.03(-0.24,0.17) | -0.08(-0.43,0.28) | -0.73(-2.79,1.33) |

**Nonanaemic**

| treatment | comparison | weight | height | haemoglobin |
| --- | --- | --- | --- | --- |
| STH deworming with any drug | Placebo or control | 0.06(-0.07,0.20) | 0.09(-0.15,0.34) | -0.18(-1.31,0.95) |
| Any STH deworming combination with praziquantel | Placebo or control | -0.02(-0.26,0.23) | 0.02(-0.36,0.40) | 0.96(-0.93,2.86) |
| Any STH deworming combination with praziquantel with iron or micronutrients | Placebo or control | -0.05(-0.36,0.27) | -0.07(-0.53,0.39) | 1.14(-0.89,3.18) |
| Any STH deworming with micronutrients or iron | Placebo or control | 0.00(-0.19,0.19) | 0.00(-0.32,0.33) | 2.07(-0.08,4.23) |
| Micronutrients or iron alone | Placebo or control | 0.00(-0.21,0.22) | -0.03(-0.34,0.29) | 2.25(-0.14,4.64) |
| Any STH deworming combination with praziquantel | STH deworming with any drug | -0.08(-0.35,0.19) | -0.07(-0.50,0.35) | 1.11(-1.16,3.37) |
| Any STH deworming combination with praziquantel with iron or micronutrients | STH deworming with any drug | -0.11(-0.44,0.23) | -0.16(-0.66,0.34) | 1.67(0.11,3.23) |
| Any STH deworming with micronutrients or iron | STH deworming with any drug | -0.06(-0.27,0.15) | -0.09(-0.46,0.28) | 1.85(0.15,3.54) |
| Micronutrients or iron alone | STH deworming with any drug | -0.06(-0.29,0.17) | -0.12(-0.48,0.25) | 0.71(-1.62,3.03) |
| Any STH deworming combination with praziquantel with iron or micronutrients | Any STH deworming combination with praziquantel | -0.03(-0.35,0.29) | -0.09(-0.58,0.40) | -0.40(-3.01,2.20) |
| Any STH deworming with micronutrients or iron | Any STH deworming combination with praziquantel | 0.02(-0.28,0.32) | -0.01(-0.50,0.47) | 0.92(-0.65,2.49) |
| Micronutrients or iron alone | Any STH deworming combination with praziquantel | 0.02(-0.27,0.30) | -0.04(-0.47,0.38) | 1.10(-0.63,2.83) |
| Any STH deworming with micronutrients or iron | Any STH deworming combination with praziquantel with iron or micronutrients | 0.05(-0.31,0.40) | 0.07(-0.47,0.62) | -0.05(-2.10,2.01) |
| Micronutrients or iron alone | Any STH deworming combination with praziquantel with iron or micronutrients | 0.05(-0.26,0.36) | 0.04(-0.45,0.53) | -1.15(-3.45,1.14) |
| Micronutrients or iron alone | Any STH deworming with micronutrients or iron | 0.00(-0.24,0.24) | -0.03(-0.42,0.36) | -0.75(-2.56,1.06) |

Additional tables 17: Deworming characterisitics table by comparisons

Placebo vs. STH

| Variable | | Placebo | | | STH | | |
| --- | --- | --- | --- | --- | --- | --- | --- |
|  |  | (n) | (%) | C.I (95%) | (n) | (%) | C.I (95%) |
| BMI for Age |  |  |  |  |  |  |  |
|  | < = -2 | 520 | 13 | (12 – 14) | 797 | 15 | (14 – 16) |
|  | > - 2 | 3568 | 87 | (86 – 88) | 4358 | 85 | (84 – 86) |
| Height for Age |  |  |  |  |  |  |  |
|  | < = -2 | 1350 | 33 | (32 – 34) | 1892 | 37 | (35 – 38) |
|  | > - 2 | 2738 | 67 | (66 – 68) | 3263 | 63 | (62 – 65) |
| Hookworm |  |  |  |  |  |  |  |
|  | 0 – No | 2411 | 59 | (57 – 60) | 2394 | 46 | (45 – 48) |
|  | 1 – 384 | 876 | 21 | (20 – 23) | 1102 | 21 | (20 – 22) |
|  | > 384 | 800 | 20 | (18 – 21) | 1659 | 32 | (31 – 33) |
| Trichuris |  |  |  |  |  |  |  |
|  | 0 – No | 1933 | 47 | (46 – 39) | 1891 | 37 | (35 – 38) |
|  | 1 – 288 | 1025 | 25 | (24 – 26) | 1321 | 26 | (24 – 27) |
|  | > 288 | 1124 | 28 | (26 – 29) | 1939 | 38 | (36 – 39) |
| *A Lumbricoides* |  |  |  |  |  |  |  |
|  | 0 - No | 2128 | 52 | (51 – 54) | 2404 | 47 | (45 – 48) |
|  | 1 – 1776 | 1109 | 27 | (26 – 28) | 1441 | 28 | (27 – 29) |
|  | > 1776 | 851 | 21 | (20 – 22) | 1309 | 25 | (24 – 27) |
| Anyworm |  |  |  |  |  |  |  |
|  | 0 | 1521 | 37 | (36 - 39) | 1487 | 29 | (28 – 30) |
|  | 1 | 1697 | 42 | (40 - 43) | 2192 | 43 | (41 – 44) |
|  | 2 | 870 | 21 | (20 – 23) | 1476 | 29 | (27 - 30) |
| Anemia |  |  |  |  |  |  |  |
|  | No | 2222 | 54 | (53 – 56) | 3048 | 59 | (58 – 60) |
|  | Yes | 1866 | 46 | (44 – 47) | 2107 | 41 | (40 – 42) |
| Age |  |  |  |  |  |  |  |
|  | < = 5 | 1123 | 27 | (26 – 29) | 1113 | 22 | (20 – 23) |
|  | > 5 | 2965 | 73 | (71 – 74) | 4042 | 78 | (77 – 80) |
| Sex |  |  |  |  |  |  |  |
|  | Male | 2171 | 53 | (52 – 55) | 2643 | 51 | (50 – 53) |
|  | Female | 1917 | 47 | (45 – 48) | 2512 | 49 | (47 – 50) |
| Total (n ) |  | 4168 |  |  | 5155 |  |  |
| Number of studies | 9 | | | | | | |

Placebo vs PZQ ± STH

| Variable | | Placebo | | | PZQ ± STH | | |
| --- | --- | --- | --- | --- | --- | --- | --- |
|  |  | (n) | (%) | C.I (95%) | (n) | (%) | C.I (95%) |
| BMI for Age |  |  |  |  |  |  |  |
|  | < = -2 | 41 | 6 | (4 - 8) | 65 | 9 | (7 - 11) |
|  | > - 2 | 602 | 94 | (92 - 96) | 686 | 91 | (89 - 93) |
| Height for Age |  |  |  |  |  |  |  |
|  | < = -2 | 169 | 26 | (23 - 30) | 215 | 29 | (25 - 32) |
|  | > - 2 | 474 | 74 | (70 - 77) | 536 | 71 | (68 - 75) |
| Hookworm |  |  |  |  |  |  |  |
|  | 0 | 177 | 28 | (24 - 31) | 192 | 26 | (22 - 29) |
|  | 1 – 384 | 253 | 39 | (36 - 43) | 298 | 40 | (36 - 43) |
|  | > 384 | 213 | 33 | (29 - 37) | 261 | 35 | (31 - 38) |
| Trichuris |  |  |  |  |  |  |  |
|  | 0 | 302 | 47 | (43 - 51) | 325 | 43 | (40 - 47) |
|  | 1 – 288 | 228 | 35 | (32 - 39) | 280 | 37 | (34 - 41) |
|  | > 288 | 113 | 18 | (15 - 21) | 146 | 19 | (17 - 22) |
| *A Lumbricoides* |  |  |  |  |  |  |  |
|  | 0 | 474 | 74 | (70 - 77) | 547 | 73 | (70 - 76) |
|  | 1 – 1776 | 33 | 5 | (3 - 7) | 47 | 6 | (5 - 8) |
|  | > 1776 | 136 | 21 | (18 - 24) | 157 | 21 | (18 - 24) |
| Anyworm |  |  |  |  |  |  |  |
|  | 0 | 93 | 14 | (12 - 17) | 97 | 13 | (11 - 15) |
|  | 1 | 384 | 60 | (56 - 64) | 477 | 65 | (60 - 67) |
|  | 2 | 166 | 26 | (22 - 29) | 177 | 24 | (21 - 27) |
| Anemia |  |  |  |  |  |  |  |
|  | No | 346 | 54 | (50 - 58) | 434 | 58 | (54 - 61) |
|  | Yes | 297 | 46 | (42 - 50) | 317 | 42 | (39 - 46) |
| Age |  |  |  |  |  |  |  |
|  | < = 5 | 1 | 0.2 | (0) | 1 | 0.1 | (0) |
|  | > 5 | 642 | 99.8 | (100) | 750 | 99.9 | (100) |
| Sex |  |  |  |  |  |  |  |
|  | Male | 347 | 54 | (50 - 58) | 416 | 55 | (52 - 59) |
|  | Female | 296 | 45 | (42 - 50) | 335 | 45 | (41 - 48) |
| Total (n) |  | 643 |  |  | 751 |  |  |
| Number of studies | 5 | | | | | | |

Placebo vs PZQ ± STH + MCN/iron

| Variable | | Placebo | | | PZQ + STH + MCN/Iron | | |
| --- | --- | --- | --- | --- | --- | --- | --- |
|  |  | (n) | (%) | C.I (95%) | (n) | (%) | C.I (95%) |
| BMI for Age |  |  |  |  |  |  |  |
|  | < = -2 | 28 | 8 | (5 - 10) | 22 | 6 | (3 - 8) |
|  | > - 2 | 345 | 92 | (90 - 95) | 352 | 94 | (92 - 97) |
| Height for Age |  |  |  |  |  |  |  |
|  | < = -2 | 73 | 20 | (16 - 24) | 71 | 19 | (15 - 23) |
|  | > - 2 | 300 | 80 | (76 - 84) | 303 | 81 | (77 - 85) |
| Hookworm |  |  |  |  |  |  |  |
|  | 0 - No | 151 | 41 | (35 - 45) | 155 | 41 | (36 - 46) |
|  | 1 – 384 | 161 | 43 | (38 - 48) | 168 | 45 | (40 - 50) |
|  | > 384 | 61 | 16 | (13 - 20) | 51 | 14 | (10 - 17) |
| Trichuris |  |  |  |  |  |  |  |
|  | 0 – No | 229 | 61 | (56 - 66) | 242 | 65 | (60 - 70) |
|  | 1 – 288 | 121 | 32 | (28 - 37) | 111 | 30 | (25 - 34) |
|  | > 288 | 23 | 6 | (4 - 9) | 21 | 6 | (3 - 8) |
| *A Lumbricoides* |  |  |  |  |  |  |  |
|  | 0 - No | 326 | 87 | (84 - 91) | 325 | 87 | (83 - 90) |
|  | 1 – 1776 | 19 | 5 | (3 - 7) | 23 | 6 | (4 - 9) |
|  | > 1776 | 28 | 8 | (5- 10) | 26 | 7 | (4 - 10) |
| Anyworm |  |  |  |  |  |  |  |
|  | 0 | 87 | 23 | (19 - 28) | 95 | 25 | (21 - 30) |
|  | 1 | 248 | 67 | (62 - 71) | 248 | 66 | (61 - 71) |
|  | 2 | 38 | 10 | (7 - 13) | 31 | 8 | (5 - 11) |
| Anemia |  |  |  |  |  |  |  |
|  | No | 158 | 42 | (37 - 47) | 178 | 48 | (43 - 53) |
|  | Yes | 215 | 58 | (53 - 63) | 196 | 52 | (47 - 57) |
| Age |  |  |  |  |  |  |  |
|  | < = 5 | 0 | 0 | (0) | 0 | 0 | (0) |
|  | > 5 | 373 | 100 | (100) | 374 | 100 | (100) |
| Sex |  |  |  |  |  |  |  |
|  | Male | 192 | 51 | (46 - 57) | 199 | 53 | (48 - 58) |
|  | Female | 181 | 49 | (43 - 54) | 175 | 47 | (42 - 52) |
| Total (n) |  | 373 |  |  | 374 |  |  |
| Number of studies | 3 | | | | | | |

Placebo vs STH + MCN/Iron

| Variable | | Placebo | | | STH + MCN/Iron | | | |
| --- | --- | --- | --- | --- | --- | --- | --- | --- |
|  |  | (n) | (%) | C.I (95%) | | (n) | (%) | C.I (95%) |
| BMI for Age |  |  |  |  | |  |  |  |
|  | < = -2 | 383 | 29 | (27 - 32) | | 405 | 28 | (26 - 31) |
|  | > - 2 | 918 | 71 | (68 - 73) | | 1019 | 72 | (69 - 74) |
| Height for Age |  |  |  |  | |  |  |  |
|  | < = -2 | 424 | 33 | (30 - 35) | | 454 | 32 | (29 - 34) |
|  | > - 2 | 877 | 67 | (65 - 70) | | 970 | 68 | (66 - 71) |
| Hookworm |  |  |  |  | |  |  |  |
|  | 0 – No | 1074 | 83 | (80 - 85) | | 1186 | 83 | (81 - 85) |
|  | 1 – 384 | 176 | 14 | (12 - 15) | | 178 | 13 | (11 - 14) |
|  | > 384 | 51 | 4 | (3 - 5) | | 60 | 4 | (3 - 5) |
| Trichuris |  |  |  |  | |  |  |  |
|  | 0 – No | 929 | 71 | (69 - 74) | | 954 | 67 | (65 - 69) |
|  | 1 – 288 | 273 | 21 | (19 - 23) | | 324 | 23 | (21 - 25) |
|  | > 288 | 99 | 8 | (6 - 9) | | 146 | 10 | (9 - 12) |
| *A Lumbricoides* |  |  |  |  | |  |  |  |
|  | 0 - No | 791 | 61 | (58 - 63) | | 850 | 60 | (57 - 62) |
|  | 1 – 1776 | 225 | 17 | (15 - 19) | | 237 | 17 | (15 - 19) |
|  | > 1776 | 285 | 22 | (20 - 24) | | 337 | 24 | (21 - 26) |
| Anyworm |  |  |  |  | |  |  |  |
|  | 0 | 657 | 50 | (48 - 53) | | 688 | 48 | (46 - 51) |
|  | 1 | 436 | 34 | (31 - 36) | | 468 | 33 | (30 - 35) |
|  | 2 | 208 | 16 | (14 - 18) | | 268 | 19 | (17 - 21) |
| Anemia |  |  |  |  | |  |  |  |
|  | No | 493 | 38 | (35- 41) | | 511 | 36 | (33 - 38) |
|  | Yes | 808 | 62 | (59 - 65) | | 913 | 64 | (62 - 67) |
| Age |  |  |  |  | |  |  |  |
|  | < = 5 | 117 | 9 | (7 - 11) | | 107 | 8 | (6 - 9) |
|  | > 5 | 1184 | 91 | (89 - 93) | | 1317 | 92 | (91 - 94) |
| Sex |  |  |  |  | |  |  |  |
|  | Male | 666 | 51 | (48 - 54) | | 729 | 51 | (49 - 54) |
|  | Female | 635 | 49 | (46 - 52) | | 695 | 49 | (46 - 51) |
| Total (n) |  | 1301 |  |  | | 1424 |  |  |
| Number of studies | 5 | | | | | | | |

Placebo vs MCN/iron

| Variable | | Placebo | | | MCN/Iron | | |
| --- | --- | --- | --- | --- | --- | --- | --- |
|  |  | (n) | (%) | C.I (95%) | (n) | (%) | C.I (95%) |
| BMI for Age |  |  |  |  |  |  |  |
|  | < = -2 | 98 | 12 | (9 - 14) | 104 | 12 | (10 - 14) |
|  | > - 2 | 744 | 88 | (86 - 91) | 750 | 88 | (86 - 90) |
| Height for Age |  |  |  |  |  |  |  |
|  | < = -2 | 249 | 30 | (26 - 33) | 252 | 30 | (26 - 33) |
|  | > - 2 | 593 | 70 | (67 - 74) | 602 | 70 | (67 - 74) |
| Hookworm |  |  |  |  |  |  |  |
|  | 0 – No | 546 | 65 | (62 - 68) | 533 | 62 | (59 - 66) |
|  | 1 – 384 | 264 | 31 | (28 - 34) | 284 | 33 | (30 - 36) |
|  | > 384 | 32 | 4 | (3 - 5) | 37 | 4 | (3 - 6) |
| Trichuris |  |  |  |  |  |  |  |
|  | 0 – No | 449 | 53 | (50 - 57) | 437 | 51 | (48 - 55) |
|  | 1 – 288 | 295 | 35 | (32 - 38) | 327 | 38 | (35 - 42) |
|  | > 288 | 98 | 12 | (9 - 14) | 90 | 11 | (8 - 13) |
| *A Lumbricoides* |  |  |  |  |  |  |  |
|  | 0 - No | 493 | 59 | (55 - 62) | 512 | 60 | (57 - 63) |
|  | 1 – 1776 | 170 | 20 | (17 - 23) | 172 | 20 | (17 - 23) |
|  | > 1776 | 179 | 21 | (18 - 24) | 170 | 20 | (17 - 23) |
| Anyworm |  |  |  |  |  |  |  |
|  | 0 | 218 | 26 | (23 - 29) | 218 | 26 | (23 - 28) |
|  | 1 | 497 | 59 | (56 - 62) | 501 | 59 | (55 - 62) |
|  | 2 | 127 | 15 | (13 - 18) | 135 | 16 | (13 - 18) |
| Anemia |  |  |  |  |  |  |  |
|  | No | 424 | 50 | (47 - 54) | 436 | 51 | (48 - 54) |
|  | Yes | 418 | 50 | (46 - 53) | 418 | 49 | (46 - 52) |
| Age |  |  |  |  |  |  |  |
|  | < = 5 | 117 | 14 | (12 - 16) | 109 | 13 | (11 - 15) |
|  | > 5 | 725 | 86 | (84 - 88) | 745 | 87 | (85 - 89) |
| Sex |  |  |  |  |  |  |  |
|  | Male | 416 | 49 | (46 - 53) | 444 | 52 | (49 - 55) |
|  | Female | 426 | 51 | (47 - 54) | 410 | 48 | (45 - 51) |
| Total (n) |  | 842 |  |  | 854 |  |  |
| Number of studies | 6 | | | | | | |

Head to Head (STH vs PZQ ± STH)

| Variable | | STH | | | PZQ ± STH | | |
| --- | --- | --- | --- | --- | --- | --- | --- |
|  |  | (n) | (%) | C.I (95%) | (n) | (%) | C.I (95%) |
| BMI for Age |  |  |  |  |  |  |  |
|  | < = -2 | 10 | 11 | (4 - 17) | 24 | 13 | (8 - 18) |
|  | > - 2 | 82 | 89 | (83 - 96) | 164 | 87 | (82 - 92) |
| Height for Age |  |  |  |  |  |  |  |
|  | < = -2 | 27 | 29 | (20 - 39) | 56 | 30 | (23 - 36) |
|  | > - 2 | 65 | 71 | (61 - 80) | 132 | 70 | (64 - 77) |
| Hookworm |  |  |  |  |  |  |  |
|  | 0 – No | 12 | 13 | (6 - 20) | 28 | 15 | (10 - 20) |
|  | 1 – 384 | 46 | 50 | (40 - 60) | 85 | 45 | (38 - 52) |
|  | > 384 | 34 | 37 | (27 - 47) | 75 | 40 | (33 - 47) |
| Trichuris |  |  |  |  |  |  |  |
|  | 0 – No | 19 | 21 | (12 - 29) | 39 | 21 | (15 - 27) |
|  | 1 – 288 | 42 | 46 | (35 - 56) | 91 | 48 | (41 - 56) |
|  | > 288 | 31 | 34 | (24 - 44) | 58 | 31 | (24 - 38) |
| *A Lumbricoides* |  |  |  |  |  |  |  |
|  | 0 - No | 64 | 70 | (60 - 79) | 120 | 64 | (57 - 71) |
|  | 1 – 1776 | 13 | 14 | (7 - 21) | 23 | 12 | (8 - 17) |
|  | > 1776 | 15 | 16 | (9 - 24) | 45 | 24 | (18 - 30) |
| Anyworm |  |  |  |  |  |  |  |
|  | 0 | 5 | 5 | (1 - 10) | 12 | 6 | (3 - 10) |
|  | 1 | 64 | 70 | (60 - 79) | 114 | 61 | (54 - 68) |
|  | 2 | 23 | 25 | (16 - 34) | 62 | 33 | (26 - 40) |
| Anemia |  |  |  |  |  |  |  |
|  | No | 63 | 68 | (59 - 78) | 137 | 73 | (66 - 79) |
|  | Yes | 29 | 31.5 | (22 - 41) | 51 | 27 | (21 - 34) |
| Age |  |  |  |  |  |  |  |
|  | < = 5 | 0 | 0 | (0) | 0 | 0 | (0) |
|  | > 5 | 92 | 100 | (100) | 188 | 100 | (100) |
| Sex |  |  |  |  |  |  |  |
|  | Male | 55 | 60 | (50 - 70) | 117 | 62 | (55 - 69) |
|  | Female | 37 | 40 | (30 - 50) | 71 | 38 | (31 - 45) |
| Total (n) |  | 92 |  |  | 188 |  |  |
| Number of studies | 1 | | | | | | |

Head to Head (STH vs STH + MCN/iron)

| Variable | | STH | | | STH + MCN/Iron | | |
| --- | --- | --- | --- | --- | --- | --- | --- |
|  |  | (n) | (%) | C.I (95%) | (n) | (%) | C.I (95%) |
| BMI for Age |  |  |  |  |  |  |  |
|  | < = -2 | 86 | 16 | (13 - 19) | 86 | 14 | (11 - 17) |
|  | > - 2 | 447 | 84 | (81 - 87) | 524 | 86 | (83 - 89) |
| Height for Age |  |  |  |  |  |  |  |
|  | < = -2 | 203 | 38 | (38 - 42) | 214 | 35 | (31 - 39) |
|  | > - 2 | 330 | 62 | (58 - 66) | 396 | 65 | (61 - 69) |
| Hookworm |  |  |  |  |  |  |  |
|  | 0 – No | 411 | 77 | (74 - 81) | 473 | 78 | (74 - 81) |
|  | 1 – 384 | 113 | 21 | (18 - 25) | 122 | 20 | (17 - 23) |
|  | > 384 | 9 | 2 | (1 - 3) | 15 | 2 | (1 - 4) |
| Trichuris |  |  |  |  |  |  |  |
|  | 0 – No | 245 | 46 | (42 - 50) | 256 | 42 | (38 - 46) |
|  | 1 – 288 | 207 | 39 | (35 - 43) | 230 | 38 | (34 - 42) |
|  | > 288 | 81 | 15 | (12 - 18) | 124 | 20 | (17 - 24) |
| *A Lumbricoides* |  |  |  |  |  |  |  |
|  | 0 - No | 232 | 44 | (39 - 48) | 250 | 41 | (37 - 45) |
|  | 1 – 1776 | 145 | 27 | (23 -31) | 164 | 27 | (23 - 30) |
|  | > 1776 | 156 | 29 | (25 - 33) | 196 | 32 | (28 - 36) |
| Anyworm |  |  |  |  |  |  |  |
|  | 0 | 138 | 26 | (22 - 30) | 137 | 22 | (19 - 26) |
|  | 1 | 285 | 53 | (49 - 58) | 310 | 51 | (47 - 55) |
|  | 2 | 110 | 21 | (17 - 24) | 163 | 27 | (23 - 30) |
| Anemia |  |  |  |  |  |  |  |
|  | No | 300 | 56 | (52 - 61) | 353 | 58 | (54 - 62) |
|  | Yes | 233 | 44 | (39 - 48) | 257 | 42 | (38 - 46) |
| Age |  |  |  |  |  |  |  |
|  | < = 5 | 101 | 19 | (16 - 22) | 107 | 18 | (15 - 21) |
|  | > 5 | 432 | 81 | (78 - 84) | 503 | 82 | (79 - 85) |
| Sex |  |  |  |  |  |  |  |
|  | Male | 271 | 51 | (47 - 55) | 314 | 51 | (47 - 55) |
|  | Female | 262 | 49 | (45 - 53) | 296 | 49 | (45 - 53) |
| Total (n) |  | 533 |  |  | 610 |  |  |
| Number of studies | 4 | | | | | | |

Head to Head (STH vs MCN/Iron)

| Variable | | STH | | | MCN/Iron | | |
| --- | --- | --- | --- | --- | --- | --- | --- |
|  |  | (n) | (%) | C.I (95%) | (n) | (%) | C.I (95%) |
| BMI for Age |  |  |  |  |  |  |  |
|  | < = -2 | 86 | 16 | (13 - 19) | 74 | 14 | (11 - 17) |
|  | > - 2 | 447 | 84 | (81 - 87) | 469 | 86 | (83 - 89) |
| Height for Age |  |  |  |  |  |  |  |
|  | < = -2 | 203 | 38 | (38 - 42) | 203 | 37 | (33 - 41) |
|  | > - 2 | 330 | 62 | (58 - 66) | 340 | 63 | (59 - 67) |
| Hookworm |  |  |  |  |  |  |  |
|  | 0 – No | 411 | 77 | (74 - 81) | 409 | 75 | (72 - 79) |
|  | 1 – 384 | 113 | 21 | (18 - 25) | 126 | 23 | (20 - 27) |
|  | > 384 | 9 | 2 | (1 - 3) | 8 | 2 | (0 - 2) |
| Trichuris |  |  |  |  |  |  |  |
|  | 0 – No | 245 | 46 | (42 - 50) | 243 | 45 | (41 - 49) |
|  | 1 – 288 | 207 | 39 | (35 - 43) | 220 | 41 | (36 - 45) |
|  | > 288 | 81 | 15 | (12 - 18) | 80 | 15 | (12 - 18) |
| *A Lumbricoides* |  |  |  |  |  |  |  |
|  | 0 - No | 232 | 44 | (39 - 48) | 243 | 45 | (41 - 49) |
|  | 1 – 1776 | 145 | 27 | (23 -31) | 150 | 28 | (24 - 31) |
|  | > 1776 | 156 | 29 | (25 - 33) | 150 | 28 | (24 - 31) |
| Anyworm |  |  |  |  |  |  |  |
|  | 0 | 138 | 26 | (22 - 30) | 139 | 26 | (22 - 29) |
|  | 1 | 285 | 53 | (49 - 58) | 286 | 53 | (48 - 57) |
|  | 2 | 110 | 21 | (17 - 24) | 118 | 22 | (18 - 25) |
| Anemia |  |  |  |  |  |  |  |
|  | No | 300 | 56 | (52 - 61) | 301 | 55 | (51 - 60) |
|  | Yes | 233 | 44 | (39 - 48) | 242 | 45 | (40 - 49) |
| Age |  |  |  |  |  |  |  |
|  | < = 5 | 101 | 19 | (16 - 22) | 108 | 20 | (17 - 23) |
|  | > 5 | 432 | 81 | (78 - 84) | 435 | 80 | (77 - 83) |
| Sex |  |  |  |  |  |  |  |
|  | Male | 271 | 51 | (47 - 55) | 280 | 52 | (47 - 56) |
|  | Female | 262 | 49 | (45 - 53) | 263 | 48 | (44 - 53) |
| Total (n) |  | 533 |  |  | 543 |  |  |
| Number of studies | 4 | | | | | | |

Head to Head (PZQ ± STH vs PZQ ± STH + MCN/Iron)

| Variable | | PZQ ± STH | | | PZQ + STH + MCN/Iron | | |
| --- | --- | --- | --- | --- | --- | --- | --- |
|  |  | (n) | (%) | C.I (95%) | (n) | (%) | C.I (95%) |
| BMI for Age |  |  |  |  |  |  |  |
|  | < = -2 | 31 | 8 | (5 - 11) | 22 | 6 | (3 - 8) |
|  | > - 2 | 354 | 92 | (89 - 95) | 352 | 94 | (92 - 97) |
| Height for Age |  |  |  |  |  |  |  |
|  | < = -2 | 74 | 19 | (15 - 23) | 71 | 19 | (15 - 23) |
|  | > - 2 | 311 | 81 | (77 - 85) | 303 | 81 | (77 - 85) |
| Hookworm |  |  |  |  |  |  |  |
|  | 0 – No | 151 | 39 | (34 - 44) | 155 | 41 | (36 - 46) |
|  | 1 – 384 | 175 | 45 | (40 - 50) | 168 | 45 | (40 - 50) |
|  | > 384 | 59 | 15 | (12 - 19) | 51 | 14 | (10 - 17) |
| Trichuris |  |  |  |  |  |  |  |
|  | 0 – No | 235 | 61 | (56 - 66) | 242 | 65 | (60 - 70) |
|  | 1 – 288 | 126 | 33 | (28 - 37) | 111 | 30 | (25 - 34) |
|  | > 288 | 24 | 6 | (4 - 9) | 21 | 6 | (3 - 8) |
| *A Lumbricoides* |  |  |  |  |  |  |  |
|  | 0 - No | 340 | 88 | (85 - 92) | 325 | 87 | (83 - 90) |
|  | 1 – 1776 | 18 | 5 | (3 - 7) | 23 | 6 | (4 - 9) |
|  | > 1776 | 27 | 7 | (4 - 10) | 26 | 7 | (4 - 10) |
| Anyworm |  |  |  |  |  |  |  |
|  | 0 | 85 | 22 | (18 - 26) | 95 | 25 | (21 - 30) |
|  | 1 | 271 | 70 | (66 - 75) | 248 | 66 | (61 - 71) |
|  | 2 | 29 | 8 | (5 - 10) | 31 | 8 | (5 - 11) |
| Anemia |  |  |  |  |  |  |  |
|  | No | 174 | 45 | (40 - 50) | 178 | 48 | (43 - 53) |
|  | Yes | 211 | 55 | (50 - 60) | 196 | 52 | (47 - 57) |
| Age |  |  |  |  |  |  |  |
|  | < = 5 | 0 | 0 | (0) | 0 | 0 | (0) |
|  | > 5 | 385 | 100 | (100) | 374 | 100 | (100) |
| Sex |  |  |  |  |  |  |  |
|  | Male | 203 | 53 | (48 - 58) | 199 | 53 | (48 - 58) |
|  | Female | 182 | 47 | (42 - 52) | 175 | 47 | (42 - 52) |
| Total (n) |  | 385 |  |  | 374 |  |  |
| Number of studies | 3 | | | | | | |

Head to Head (PZQ ± STH vs MCN/Iron)

| Variable | | PZQ ± STH | | | MCN/Iron | | |
| --- | --- | --- | --- | --- | --- | --- | --- |
|  |  | (n) | (%) | C.I (95%) | (n) | (%) | C.I (95%) |
| BMI for Age |  |  |  |  |  |  |  |
|  | < = -2 | 23 | 7 | (5 - 10) | 30 | 10 | (6 - 13) |
|  | > - 2 | 284 | 93 | (90 - 95) | 281 | 90 | (87 - 94) |
| Height for Age |  |  |  |  |  |  |  |
|  | < = -2 | 52 | 17 | (13 -21) | 49 | 16 | (12 - 20) |
|  | > - 2 | 255 | 83 | (79 - 87) | 262 | 84 | (80 - 88) |
| Hookworm |  |  |  |  |  |  |  |
|  | 0 – No | 139 | 45 | (40 - 51) | 124 | 40 | (34 - 45) |
|  | 1 – 384 | 147 | 48 | (42 - 54) | 158 | 51 | (45 - 56) |
|  | > 384 | 21 | 7 | (4 - 10) | 29 | 9 | (6 - 13) |
| Trichuris |  |  |  |  |  |  |  |
|  | 0 – No | 187 | 61 | (55 - 66) | 194 | 62 | (57 - 68) |
|  | 1 – 288 | 111 | 36 | (31 - 42) | 107 | 34 | (29 - 40) |
|  | > 288 | 9 | 3 | (1 - 5) | 10 | 3 | (1 - 5) |
| *A Lumbricoides* |  |  |  |  |  |  |  |
|  | 0 – No | 270 | 88 | (84 - 92) | 269 | 87 | (83 - 90) |
|  | 1 – 1776 | 16 | 5 | (3 - 8) | 22 | 7 | (4 - 10) |
|  | > 1776 | 21 | 7 | (4 - 10) | 20 | 6 | (4 - 9) |
| Anyworm |  |  |  |  |  |  |  |
|  | 0 | 85 | 28 | (23 - 33) | 79 | 25 | (21 - 30) |
|  | 1 | 207 | 67 | (62 - 73) | 215 | 69 | (64 - 74) |
|  | 2 | 15 | 5 | (2 - 7) | 17 | 6 | (3 - 8) |
| Anemia |  |  |  |  |  |  |  |
|  | No | 125 | 41 | (35 - 46) | 135 | 43 | (38 - 49) |
|  | Yes | 182 | 59 | (54 - 65) | 176 | 57 | (51 - 62) |
| Age |  |  |  |  |  |  |  |
|  | < = 5 | 0 | 0 | (0) | 1 | 0.3 | (0) |
|  | > 5 | 307 | 100 | (100) | 310 | 99.7 | (100) |
| Sex |  |  |  |  |  |  |  |
|  | Male | 163 | 53 | (47 - 59) | 164 | 53 | (47 - 58) |
|  | Female | 144 | 47 | (41 - 53) | 147 | 47 | (42 - 53) |
| Total (n) |  | 307 |  |  | 311 |  |  |
| Number of studies | 2 | | | | | | |

Head to Head (PZQ ± STH + MCN/Iron vs MCN/Iron)

| Variable | | PZQ + STH + MCN/Iron | | | MCN/Iron | | |
| --- | --- | --- | --- | --- | --- | --- | --- |
|  |  | (n) | (%) | C.I (95%) | (n) | (%) | C.I (95%) |
| BMI for Age |  |  |  |  |  |  |  |
|  | < = -2 | 19 | 6 | (3 - 9) | 30 | 10 | (6 - 13) |
|  | > - 2 | 291 | 94 | (91 - 97) | 281 | 90 | (87 - 94) |
| Height for Age |  |  |  |  |  |  |  |
|  | < = -2 | 52 | 17 | (13 - 21) | 49 | 16 | (12 - 20) |
|  | > - 2 | 258 | 83 | (79 - 87) | 262 | 84 | (80 - 88) |
| Hookworm |  |  |  |  |  |  |  |
|  | 0 – No | 145 | 47 | (41 - 52) | 124 | 40 | (34 - 45) |
|  | 1 – 384 | 148 | 48 | (42 - 53) | 158 | 51 | (45 - 56) |
|  | > 384 | 17 | 6 | (3 - 8) | 29 | 9 | (6 - 13) |
| Trichuris |  |  |  |  |  |  |  |
|  | 0 – No | 211 | 68 | (63 - 73) | 194 | 62 | (57 - 68) |
|  | 1 – 288 | 89 | 29 | (24 - 34) | 107 | 34 | (29 - 40) |
|  | > 288 | 10 | 3 | (1 - 5) | 10 | 3 | (1 - 5) |
| *A Lumbricoides* |  |  |  |  |  |  |  |
|  | 0 - No | 274 | 88 | (85 - 92) | 269 | 87 | (83 - 90) |
|  | 1 – 1776 | 21 | 7 | (4 - 10) | 22 | 7 | (4 - 10) |
|  | > 1776 | 15 | 5 | (2 - 7) | 20 | 6 | (4 - 9) |
| Anyworm |  |  |  |  |  |  |  |
|  | 0 | 95 | 31 | (25 - 38) | 79 | 25 | (21 - 30) |
|  | 1 | 202 | 65 | (60 - 70) | 215 | 69 | (64 - 74) |
|  | 2 | 13 | 4 | (2 - 6) | 17 | 6 | (3 - 8) |
| Anemia |  |  |  |  |  |  |  |
|  | No | 133 | 43 | (37 - 48) | 135 | 43 | (38 - 49) |
|  | Yes | 177 | 57 | (52 - 63) | 176 | 57 | (51 - 62) |
| Age |  |  |  |  |  |  |  |
|  | < = 5 | 0 | 0 | (0) | 1 | 0.3 | (0) |
|  | > 5 | 310 | 100 | (100) | 310 | 99.7 | (100) |
| Sex |  |  |  |  |  |  |  |
|  | Male | 157 | 51 | (45 - 56) | 164 | 53 | (47 - 58) |
|  | Female | 153 | 49 | (44 - 55) | 147 | 47 | (42 - 53) |
| Total (n) |  | 310 |  |  | 311 |  |  |
| Number of studies | 2 | | | | | | |

Head to Head (STH + MCN/Iron vs MCN/Iron)

| Variable | | STH + MCN/Iron | | | MCN/Iron | | |
| --- | --- | --- | --- | --- | --- | --- | --- |
|  |  | (n) | (%) | C.I (95%) | (n) | (%) | C.I (95%) |
| BMI for Age |  |  |  |  |  |  |  |
|  | < = -2 | 86 | 14 | (11 - 17) | 74 | 14 | (11 - 17) |
|  | > - 2 | 524 | 86 | (83 - 89) | 469 | 86 | (83 - 89) |
| Height for Age |  |  |  |  |  |  |  |
|  | < = -2 | 214 | 35 | (31 - 39) | 203 | 37 | (33 - 41) |
|  | > - 2 | 396 | 65 | (61 - 69) | 340 | 63 | (59 - 67) |
| Hookworm |  |  |  |  |  |  |  |
|  | 0 – No | 473 | 78 | (74 - 81) | 409 | 75 | (72 - 79) |
|  | 1 – 384 | 122 | 20 | (17 - 23) | 126 | 23 | (20 - 27) |
|  | > 384 | 15 | 2 | (1 - 4) | 8 | 2 | (0 - 2) |
| Trichuris |  |  |  |  |  |  |  |
|  | 0 – No | 256 | 42 | (38 - 46) | 243 | 45 | (41 - 49) |
|  | 1 – 288 | 230 | 38 | (34 - 42) | 220 | 41 | (36 - 45) |
|  | > 288 | 124 | 20 | (17 - 24) | 80 | 15 | (12 - 18) |
| *A Lumbricoides* |  |  |  |  |  |  |  |
|  | 0 - No | 250 | 41 | (37 - 45) | 243 | 45 | (41 - 49) |
|  | 1 – 1776 | 164 | 27 | (23 - 30) | 150 | 28 | (24 - 31) |
|  | > 1776 | 196 | 32 | (28 - 36) | 150 | 28 | (24 - 31) |
| Anyworm |  |  |  |  |  |  |  |
|  | 0 | 137 | 22 | (19 - 26) | 139 | 26 | (22 - 29) |
|  | 1 | 310 | 51 | (47 - 55) | 286 | 53 | (48 - 57) |
|  | 2 | 163 | 27 | (23 - 30) | 118 | 22 | (18 - 25) |
| Anemia |  |  |  |  |  |  |  |
|  | No | 353 | 58 | (54 - 62) | 301 | 55 | (51 - 60) |
|  | Yes | 257 | 42 | (38 - 46) | 242 | 45 | (40 - 49) |
| Age |  |  |  |  |  |  |  |
|  | < = 5 | 107 | 18 | (15 - 21) | 108 | 20 | (17 - 23) |
|  | > 5 | 503 | 82 | (79 - 85) | 435 | 80 | (77 - 83) |
| Sex |  |  |  |  |  |  |  |
|  | Male | 314 | 51 | (47 - 55) | 280 | 52 | (47 - 56) |
|  | Female | 296 | 49 | (45 - 53) | 263 | 48 | (44 - 53) |
| Total (n) |  | 610 |  |  | 543 |  |  |
| Number of studies | 4 | | | | | | |

Note: There are no direct head to head comparisons for: 2 vs 4, 3 vs 5, 4 vs 5

Additional tables 18: the interaction effects used to test for effect size modification

| **EM_ Baz -2_ Any STH vs placebo** | | |
| --- | --- | --- |
| **Outcome or Subgroup** | **Effect Estimate** | **Test for subgroup differences** |
| 1 Weight | 0.00 (-0.08, 0.09) | Chi² = 0.06, df = 1 (P = 0.80), I² = 0% |
| 1.1 >-2 | 0.01 (-0.09, 0.11) |  |
| 1.2 <-2 | -0.02 (-0.24, 0.20) |  |
| 2 Height | 0.07 (-0.12, 0.25) | Chi² = 0.55, df = 1 (P = 0.46), I² = 0% |
| 2.1 >-2 | 0.09 (-0.11, 0.29) |  |
| 2.2 <-2 | -0.13 (-0.68, 0.42) |  |
| 3 Hemoglobin | 0.25 (-0.62, 1.12) | Chi² = 0.67, df = 1 (P = 0.41), I² = 0% |
| 3.1 >-2 | 0.40 (-0.54, 1.34) |  |
| 3.2 <-2 | -0.63 (-2.90, 1.64) |  |
| **EM_BAZ -2_ Any_STH + PZQ vs placebo** | | |
| **Outcome or Subgroup** | **Effect Estimate** | **Test for subgroup differences** |
| 1 Weight | 0.03 (-0.12, 0.18) | Chi² = 0.25, df = 1 (P = 0.62), I² = 0% |
| 1.1 >-2 | 0.04 (-0.12, 0.20) |  |
| 1.2 <-2 | -0.10 (-0.63, 0.43) |  |
| 2 Height | -0.09 (-0.35, 0.17) | Chi² = 0.64, df = 1 (P = 0.42), I² = 0% |
| 2.1 >-2 | -0.05 (-0.32, 0.22) |  |
| 2.2 <-2 | -0.39 (-1.17, 0.39) |  |
| 3 Hemoglobin | 1.82 (0.51, 3.13) | Chi² = 0.00, df = 1 (P = 1.00), I² = 0% |
| 3.1 >-2 | 1.82 (0.45, 3.19) |  |
| 3.2 <-2 | 1.82 (-2.71, 6.35) |  |
| **EM_BAZ_2_ Any STH + PZQ + iron or MCN vs placebo** | | |
| **Outcome or Subgroup** | **Effect Estimate** | **Test for subgroup differences** |
| 1 Weight | -0.03 (-0.26, 0.20) | Chi² = 0.42, df = 1 (P = 0.52), I² = 0% |
| 1.1 >-2 | -0.01 (-0.25, 0.23) |  |
| 1.2 <-2 | -0.30 (-1.14, 0.54) |  |
| 2 Height | -0.02 (-0.36, 0.31) | Chi² = 0.00, df = 1 (P = 0.96), I² = 0% |
| 2.1 >-2 | -0.02 (-0.37, 0.33) |  |
| 2.2 <-2 | -0.05 (-1.21, 1.11) |  |
| 3 Hemoglobin | 2.71 (1.00, 4.43) | Chi² = 0.04, df = 1 (P = 0.85), I² = 0% |
| 3.1 >-2 | 2.76 (0.98, 4.54) |  |
| 3.2 <-2 | 2.12 (-4.33, 8.57) |  |
| **EM_BAZ-2_ Any STH +MCN or iron vs placebo** | | |
| **Outcome or Subgroup** | **Effect Estimate** | **Test for subgroup differences** |
| 1 Weight | -0.01 (-0.13, 0.10) | Chi² = 0.05, df = 1 (P = 0.82), I² = 0% |
| 1.1 >-2 | -0.02 (-0.16, 0.12) |  |
| 1.2 <-2 | 0.01 (-0.21, 0.23) |  |
| 2 Height | -0.02 (-0.26, 0.22) | Chi² = 0.33, df = 1 (P = 0.56), I² = 0% |
| 2.1 >-2 | 0.02 (-0.25, 0.29) |  |
| 2.2 <-2 | -0.15 (-0.66, 0.36) |  |
| 3 Hemoglobin | 1.96 (0.81, 3.11) | Chi² = 0.55, df = 1 (P = 0.46), I² = 0% |
| 3.1 >-2 | 2.23 (0.88, 3.58) |  |
| 3.2 <-2 | 1.26 (-0.92, 3.44) |  |
| **EM_BAZ-2_ MCN or iron vs placebo** | | |
| **Outcome or Subgroup** | **Effect Estimate** | **Test for subgroup differences** |
| 1 Weight | -0.02 (-0.15, 0.11) | Chi² = 1.40, df = 1 (P = 0.24), I² = 28.8% |
| 1.1 >-2 | 0.01 (-0.13, 0.15) |  |
| 1.2 <-2 | -0.23 (-0.60, 0.14) |  |
| 2 Height | -0.07 (-0.31, 0.16) | Chi² = 0.80, df = 1 (P = 0.37), I² = 0% |
| 2.1 >-2 | -0.03 (-0.28, 0.22) |  |
| 2.2 <-2 | -0.33 (-0.94, 0.28) |  |
| 3 Hemoglobin | 1.32 (0.14, 2.50) | Chi² = 0.70, df = 1 (P = 0.40), I² = 0% |
| 3.1 >-2 | 1.12 (-0.15, 2.39) |  |
| 3.2 <-2 | 2.58 (-0.60, 5.76) |  |
| **EM_HAZ 2_STH vs placebo** | | |
| **Outcome or Subgroup** | **Effect Estimate** | **Test for subgroup differences** |
| 1 Weight | 0.02 (-0.06, 0.11) | Chi² = 0.91, df = 1 (P = 0.34), I² = 0% |
| 1.1 >-2 | 0.05 (-0.05, 0.15) |  |
| 1.2 <-2 | -0.04 (-0.20, 0.12) |  |
| 2 Height | 0.06 (-0.10, 0.23) | Chi² = 3.06, df = 1 (P = 0.08), I² = 67.3% |
| 2.1 >-2 | -0.03 (-0.23, 0.17) |  |
| 2.2 <-2 | 0.30 (-0.01, 0.61) |  |
| 3 Hemoglobin | 0.40 (-0.49, 1.29) | Chi² = 0.81, df = 1 (P = 0.37), I² = 0% |
| 3.1 >-2 | 0.09 (-1.03, 1.21) |  |
| 3.2 <-2 | 0.94 (-0.53, 2.41) |  |
| **EM_HAZ 2_ Any_STH + PZQ vs placebo** | | |
| **Outcome or Subgroup** | **Effect Estimate** | **Test for subgroup differences** |
| 1 Weight | 0.05 (-0.11, 0.21) | Chi² = 1.11, df = 1 (P = 0.29), I² = 10.0% |
| 1.1 >-2 | -0.01 (-0.21, 0.19) |  |
| 1.2 <-2 | 0.18 (-0.11, 0.47) |  |
| 2 Height | 0.10 (-0.14, 0.34) | Chi² = 0.12, df = 1 (P = 0.73), I² = 0% |
| 2.1 >-2 | 0.12 (-0.15, 0.39) |  |
| 2.2 <-2 | 0.02 (-0.47, 0.51) |  |
| 3 Hemoglobin | 1.92 (0.56, 3.29) | Chi² = 0.16, df = 1 (P = 0.69), I² = 0% |
| 3.1 >-2 | 2.08 (0.51, 3.65) |  |
| 3.2 <-2 | 1.44 (-1.32, 4.20) |  |
| **EM_HAZ 2_ Any STH + PZQ + iron or MCN vs placebo** | | |
| **Outcome or Subgroup** | **Effect Estimate** | **Test for subgroup differences** |
| 1 Weight | -0.03 (-0.26, 0.19 | Chi² = 0.20, df = 1 (P = 0.65), I² = 0% |
| 1.1 >-2 | -0.06 (-0.31, 0.19) |  |
| 1.2 <-2 | 0.07 (-0.44, 0.58) |  |
| 2 Height | 0.11 (-0.21, 0.43) | Chi² = 0.00, df = 1 (P = 1.00), I² = 0% |
| 2.1 >-2 | 0.11 (-0.24, 0.46) |  |
| 2.2 <-2 | 0.11 (-0.62, 0.84) |  |
| 3 Hemoglobin | 2.72 (1.02, 4.41) | Chi² = 0.02, df = 1 (P = 0.88), I² = 0% |
| 3.1 >-2 | 2.78 (0.90, 4.66) |  |
| 3.2 <-2 | 2.45 (-1.45, 6.35) |  |
| **EM_HAZ 2_ Any STH +MCN or iron vs placebo** | | |
| **Outcome or Subgroup** | **Effect Estimate** | **Test for subgroup differences** |
| 1 Weight | -0.03 (-0.15, 0.09) | Chi² = 0.02, df = 1 (P = 0.88), I² = 0% |
| 1.1 >-2 | -0.02 (-0.18, 0.14) |  |
| 1.2 <-2 | -0.04 (-0.24, 0.16) |  |
| 2 Height | -0.00 (-0.23, 0.23) | Chi² = 0.00, df = 1 (P = 0.97), I² = 0% |
| 2.1 >-2 | 0.00 (-0.27, 0.27) |  |
| 2.2 <-2 | -0.01 (-0.42, 0.40) |  |
| 3 Hemoglobin | 2.04 (0.89, 3.19) | Chi² = 0.67, df = 1 (P = 0.41), I² = 0% |
| 3.1 >-2 | 1.71 (0.32, 3.10) |  |
| 3.2 <-2 | 2.74 (0.70, 4.78) |  |
| **EM_HAZ 2_ MCN or iron vs placebo** | | |
| **Outcome or Subgroup** | **Effect Estimate** | **Test for subgroup differences** |
| 1 Weight | -0.03 (-0.17, 0.11) | Chi² = 0.04, df = 1 (P = 0.84), I² = 0% |
| 1.1 >-2 | -0.02 (-0.20, 0.16) |  |
| 1.2 <-2 | -0.05 (-0.29, 0.19) |  |
| 2 Height | 0.12 (-0.11, 0.35) | Chi² = 0.06, df = 1 (P = 0.81), I² = 0% |
| 2.1 >-2 | 0.14 (-0.13, 0.41) |  |
| 2.2 <-2 | 0.08 (-0.33, 0.49) |  |
| 3 Hemoglobin | 1.35 (0.20, 2.50) | Chi² = 0.00, df = 1 (P = 0.96), I² = 0% |
| 3.1 >-2 | 1.37 (0.06, 2.68) |  |
| 3.2 <-2 | 1.30 (-1.09, 3.69) |  |
| **EM_Sex_Any STH vs placebo** | | |
| **Outcome or Subgroup** | **Effect Estimate** | **Test for subgroup differences** |
| 1 Weight | 0.01 (-0.08, 0.10) | Chi² = 0.29, df = 1 (P = 0.59), I² = 0% |
| 1.1 female | 0.04 (-0.10, 0.18) |  |
| 1.2 male | -0.01 (-0.13, 0.11) |  |
| 2 Height | 0.08 (-0.08, 0.24) | Chi² = 0.31, df = 1 (P = 0.58), I² = 0% |
| 2.1 female | 0.04 (-0.18, 0.26) |  |
| 2.2 male | 0.13 (-0.11, 0.37) |  |
| 3 Hemoglobin | 0.30 (-0.53, 1.13) | Chi² = 0.00, df = 1 (P = 0.96), I² = 0% |
| 3.1 female | 0.32 (-0.84, 1.48) |  |
| 3.2 male | 0.28 (-0.92, 1.48) |  |
| **EM_Sex_ Any_STH + PZQ vs placebo** | | |
| **Outcome or Subgroup** | **Effect Estimate** | **Test for subgroup differences** |
| 1 Weight | 0.06 (-0.10, 0.21) | Chi² = 3.01, df = 1 (P = 0.08), I² = 66.8% |
| 1.1 female | 0.19 (-0.03, 0.41) |  |
| 1.2 male | -0.08 (-0.30, 0.14) |  |
| 2 Height | -0.07 (-0.32, 0.17) | Chi² = 0.35, df = 1 (P = 0.56), I² = 0% |
| 2.1 female | 0.00 (-0.35, 0.35) |  |
| 2.2 male | -0.15 (-0.50, 0.20) |  |
| 3 Hemoglobin | 1.82 (0.51, 3.12) | Chi² = 0.06, df = 1 (P = 0.81), I² = 0% |
| 3.1 female | 1.64 (-0.30, 3.58) |  |
| 3.2 male | 1.96 (0.20, 3.72) |  |
| **EM_Sex_ Any STH + PZQ + iron or MCN vs placebo** | | |
| **Outcome or Subgroup** | **Effect Estimate** | **Test for subgroup differences** |
| 1 Weight | -0.01 (-0.25, 0.23) | Chi² = 1.87, df = 1 (P = 0.17), I² = 46.6% |
| 1.1 female | -0.21 (-0.58, 0.16) |  |
| 1.2 male | 0.13 (-0.18, 0.44) |  |
| 2 Height | -0.06 (-0.37, 0.26) | Chi² = 0.00, df = 1 (P = 0.97), I² = 0% |
| 2.1 female | -0.06 (-0.49, 0.37) |  |
| 2.2 male | -0.05 (-0.50, 0.40) |  |
| 3 Hemoglobin | 2.78 (1.09, 4.46) | Chi² = 0.14, df = 1 (P = 0.71), I² = 0% |
| 3.1 female | 3.11 (0.70, 5.52) |  |
| 3.2 male | 2.46 (0.11, 4.81) |  |
| **EM_Sex_ Any STH +MCN or iron vs placebo** | | |
| **Outcome or Subgroup** | **Effect Estimate** | **Test for subgroup differences** |
| 1 Weight | -0.02 (-0.14, 0.11) | Chi² = 0.30, df = 1 (P = 0.58), I² = 0% |
| 1.1 Female | 0.02 (-0.16, 0.20) |  |
| 1.2 male | -0.05 (-0.23, 0.13) |  |
| 2 Height | -0.03 (-0.25, 0.19) | Chi² = 0.33, df = 1 (P = 0.57), I² = 0% |
| 2.1 female | -0.09 (-0.38, 0.20) |  |
| 2.2 male | 0.04 (-0.29, 0.37) |  |
| 3 Hemoglobin | 2.16 (1.02, 3.29) | Chi² = 0.04, df = 1 (P = 0.85), I² = 0% |
| 3.1 female | 2.04 (0.39, 3.69) |  |
| 3.2 male | 2.26 (0.69, 3.83) |  |
| **EM_Sex_ MCN or iron vs placebo** | | |
| **Outcome or Subgroup** | **Effect Estimate** | **Test for subgroup differences** |
| 20.1 Weight | -0.02 (-0.16, 0.12) | Chi² = 0.08, df = 1 (P = 0.78), I² = 0% |
| 20.1.1 female | -0.04 (-0.24, 0.16) |  |
| 20.1.2 male | 0.00 (-0.20, 0.20) |  |
| 20.2 Height | -0.07 (-0.29, 0.14) | Chi² = 0.30, df = 1 (P = 0.58), I² = 0% |
| 20.2.1 female | -0.13 (-0.42, 0.16) |  |
| 20.2.2 male | -0.01 (-0.32, 0.30) |  |
| 20.3 Hemoglobin | 1.36 (0.19, 2.53) | Chi² = 0.06, df = 1 (P = 0.80), I² = 0% |
| 20.3.1 female | 1.50 (-0.11, 3.11) |  |
| 20.3.2 male | 1.20 (-0.51, 2.91) |  |
|  | | |
| **EM_Age_STH vs placebo** | | |
| **Outcomes and subgroup** | **Effect Estimates** | **Test for subgroup differences** |
| 1 Weight | 0.01 (-0.07, 0.09) | Chi² = 0.86, df = 1 (P = 0.35), I² = 0% |
| 1.1≥ 5 years | 0.06 (-0.08, 0.20) |  |
| 1.2 < 5 years | -0.02 (-0.12, 0.08) |  |
| 2 Height | 0.08 (-0.10, 0.26) | Chi² = 0.60, df = 1 (P = 0.44), I² = 0% |
| 2.1 ≥ 5 years | 0.03 (-0.19, 0.25) |  |
| 2.2 < 5 years | 0.18 (-0.13, 0.49) |  |
| 3 Hemoglobin | 0.35 (-0.54, 1.23) | Chi² = 0.00, df = 1 (P = 0.97), I² = 0% |
| 3.1 ≥ 5 years | 0.33 (-0.79, 1.45) |  |
| 3.2 < 5 years | 0.37 (-1.06, 1.80) |  |
| **EM_Age_Any STH +PZQ vs placebo** | | |
| **Outcome or Subgroup** | **Effect Estimate** | **Test for subgroup differences** |
| 1 Weight | 1.12 (0.95, 1.31) | Chi² = 0.00, df = 1 (P = 0.99), I² = 0% |
| 1.1 ≥ 5 years | 1.12 (0.95, 1.31) |  |
| 1.2 < 5 years | 1.08 (0.02, 61.41) |  |
| 2 Height | -0.08 (-0.32, 0.15) | Chi² = 0.29, df = 1 (P = 0.59), I² = 0% |
| 2.1 ≥ 5 years | -0.08 (-0.32, 0.16) |  |
| 2.2 < 5 years | -2.04 (-9.17, 5.09) |  |
| 3 Hemoglobin | 1.94 (0.62, 3.25) | Chi² = 0.19, df = 1 (P = 0.67), I² = 0% |
| 3.1 ≥ 5 years | 1.93 (0.62, 3.24) |  |
| 3.2 > 5 years | 14.19 (-41.53, 69.91) |  |
| **EM_Age_Any STH + PZQ + iron or MCN vs placebo** | | |
| **Outcome or Subgroup** | **Effect Estimate** | **Test for subgroup differences** |
| 1 Weight | 0.05 (-0.20, 0.30) | Not applicable |
| 1.1 ≥ 5 years | 0.05 (-0.20, 0.30) |  |
| 1.2 < 5 years | Not estimable |  |
| 2 Height | -0.05 (-0.34, 0.24) | Not applicable |
| 2.1 ≥ 5 years | -0.05 (-0.34, 0.24) |  |
| 2.2 < 5 years | Not estimable |  |
| 3 Hemoglobin | 2.83 (1.16, 4.50) | Not applicable |
| 3.1 ≥ 5 years | 2.83 (1.16, 4.50) |  |
| 3.2 < 5 years | Not estimable |  |
| **EM_Age_Any STH +MCN or iron vs placebo** | | |
| **Outcome or Subgroup** | **Effect Estimate** | **Test for subgroup differences** |
| 1 Weight | -0.02 (-0.15, 0.11) | Chi² = 0.00, df = 1 (P = 1.00), I² = 0% |
| 1.1 ≥ 5 years | -0.02 (-0.18, 0.14) |  |
| 1.2 < 5 years | -0.02 (-0.26, 0.22) |  |
| 2 Height | -0.06 (-0.28, 0.17) | Chi² =0.01,df = 1(P =0.92), I² = 0% |
| 2.1 ≥ 5 years | -0.06 (-0.30, 0.18) |  |
| 2.2 < 5 years | -0.02 (-0.76, 0.72) |  |
| 3 Hemoglobin | 2.06 (0.82, 3.30) | Chi² =0.03,df = 1(P =0.85), I² = 0% |
| 3.1 ≥ 5 years | 2.10 (0.79, 3.41) |  |
| 3.2 < 5 years | 1.73 (-1.99, 5.45) |  |
| **EM_Age_MCN or iron vs placebo** | | |
| **Outcome or Subgroup** | **Effect Estimate** | **Test for subgroup differences** |
| 1 Weight | -0.03 (-0.16, 0.10) | Chi² = 2.33, df = 1 (P = 0.13), I² = 57.0% |
| 1.1 ≥ 5 years | 0.04 (-0.12, 0.20) |  |
| 1.2 < 5 years | -0.18 (-0.42, 0.06) |  |
| 2 Height | -0.08 (-0.30, 0.15) | Chi² = 0.00, df = 1 (P = 0.98), I² = 0% |
| 2.1 ≥ 5 years | -0.08 (-0.32, 0.16) |  |
| 2.2 < 5 years | -0.07 (-0.83, 0.69) |  |
| 3 Hemoglobin | 1.37 (0.20, 2.55) | Chi² = 0.05, df = 1 (P = 0.82), I² = 0% |
| 3.1 ≥ 5 years | 1.33 (0.10, 2.56) |  |
| 3.2 < 5years | 1.79 (-2.07, 5.65) |  |
| **EM_Ascaris_Any STH vs placebo** | | |
| **Outcome or Subgroup** | **Effect Estimate** | **Test for subgroup differences** |
| 1 Weight | 0.03 (-0.06, 0.12) | Chi² = 0.66, df = 2 (P = 0.72), I² = 0% |
| 1.1 ascaris0 | 0.00 (-0.12, 0.12) |  |
| 1.2 ascaris1 | 0.07 (-0.13, 0.27) |  |
| 1.3 ascaris2 | 0.08 (-0.12, 0.28) |  |
| 2 Height | 0.09 (-0.06, 0.23) | Chi² = 0.90, df = 2 (P = 0.64), I² = 0% |
| 2.1 ascaris0 | 0.07 (-0.13, 0.27) |  |
| 2.2 ascaris1 | 0.25 (-0.12, 0.62) |  |
| 2.3 ascaris2 | 0.04 (-0.21, 0.29) |  |
| 3 Hemoglobin | 0.25 (-0.60, 1.10) | Chi² = 0.38, df = 2 (P = 0.83), I² = 0% |
| 3.1 ascaris0 | 0.48 (-0.66, 1.62) |  |
| 3.2 ascaris1 | -0.10 (-1.73, 1.53) |  |
| 3.3 ascaris2 | 0.03 (-2.05, 2.11) |  |
| **EM_Ascaris_ Any_STH + PZQ vs placebo** | | |
| **Outcome or Subgroup** | **Effect Estimate** | **Test for subgroup differences** |
| 1 Weight | 0.03 (-0.14, 0.20) | Chi² = 1.85, df = 2 (P = 0.40), I² = 0% |
| 1.1 ascaris0 | 0.10 (-0.10, 0.30) |  |
| 1.2 ascaris1 | -0.10 (-0.81, 0.61) |  |
| 1.3 ascaris2 | -0.17 (-0.52, 0.18) |  |
| 2 Height | -0.08 (-0.32, 0.17) | Chi² = 0.17, df = 2 (P = 0.92), I² = 0% |
| 2.1 ascaris0 | -0.10 (-0.39, 0.19) |  |
| 2.2 ascaris1 | -0.19 (-1.35, 0.97) |  |
| 2.3 ascaris2 | 0.00 (-0.45, 0.45) |  |
| 3 Hemoglobin | 1.84 (0.52, 3.16) | Chi² = 0.01, df = 2 (P = 1.00), I² = 0% |
| 3.1 ascaris0 | 1.86 (0.35, 3.37) |  |
| 3.2 ascaris1 | 1.89 (-3.62, 7.40) |  |
| 3.3 ascaris2 | 1.73 (-1.41, 4.87) |  |
| **EM_Ascaris_ Any STH + PZQ + iron or MCN vs placebo** | | |
| **Outcome or Subgroup** | **Effect Estimate** | **Test for subgroup differences** |
| 1 Weight | -0.05 (-0.28, 0.18) | Chi² = 3.93, df = 2 (P = 0.14), I² = 49.1% |
| 1.1 ascaris0 | 0.05 (-0.20, 0.30) |  |
| 1.2 ascaris1 | -0.69 (-1.57, 0.19) |  |
| 1.3 ascaris2 | -0.52 (-1.32, 0.28) |  |
| 2 Height | -0.11 (-0.43, 0.22) | Chi² = 1.38, df = 2 (P = 0.50), I² = 0% |
| 2.1 ascaris0 | -0.03 (-0.38, 0.32) |  |
| 2.2 ascaris1 | -0.82 (-2.31, 0.67) |  |
| 2.3 ascaris2 | -0.40 (-1.42, 0.62) |  |
| 3 Hemoglobin | 2.71 (1.05, 4.37) | Chi² = 1.62, df = 2 (P = 0.44), I² = 0% |
| 3.1 ascaris0 | 2.50 (0.76, 4.24) |  |
| 3.2 ascaris1 | 1.69 (-6.46, 9.84) |  |
| 3.3 ascaris2 | 7.37 (-0.08, 14.82) |  |
| **EM_Ascaris_ Any STH +MCN or iron vs placebo** | | |
| **Outcome or Subgroup** | **Effect Estimate** | **Test for subgroup differences** |
| 1 Weight | -0.01 (-0.13, 0.12) | Chi² = 1.89, df = 2 (P = 0.39), I² = 0% |
| 1.1 ascaris0 | 0.02 (-0.16, 0.20) |  |
| 1.2 acaris1 | -0.19 (-0.48, 0.10) |  |
| 1.3 ascaris2 | 0.06 (-0.18, 0.30) |  |
| 2 Height | -0.06 (-0.26, 0.15) | Chi² = 0.02, df = 2 (P = 0.99), I² = 0% |
| 2.1 ascaris0 | -0.07 (-0.40, 0.26) |  |
| 2.2 ascaris1 | -0.02 (-0.55, 0.51) |  |
| 2.3 acaris2 | -0.06 (-0.35, 0.23) |  |
| 3 Hemoglobin | 2.38 (1.17, 3.59) | Chi² = 0.43, df = 2 (P = 0.81), I² = 0% |
| 3.1 ascaris0 | 2.43 (0.72, 4.14) |  |
| 3.2 ascaris1 | 1.70 (-0.85, 4.25) |  |
| 3.3 ascaris2 | 2.85 (0.52, 5.18) |  |
| **EM_Ascaris_ MCN or iron vs placebo** | | |
| **Outcome or Subgroup** | **Effect Estimate** | **Test for subgroup differences** |
| 1 Weight | -0.02 (-0.15, 0.12) | Chi² = 0.22, df = 2 (P = 0.90), I² = 0% |
| 1.1 ascaris0 | -0.04 (-0.22, 0.14) |  |
| 1.2 ascaris1 | 0.00 (-0.31, 0.31) |  |
| 1.3 ascaris2 | 0.04 (-0.25, 0.33) |  |
| 2 Height | -0.12 (-0.33, 0.10) | Chi² = 0.27, df = 2 (P = 0.87), I² = 0% |
| 2.1 ascaris0 | -0.12 (-0.41, 0.17) |  |
| 2.2 ascaris1 | 0.01 (-0.56, 0.58) |  |
| 2.3 ascaris2 | -0.17 (-0.54, 0.20) |  |
| 3 Hemoglobin | 1.32 (0.17, 2.47) | Chi² = 0.78, df = 2 (P = 0.68), I² = 0% |
| 3.1 ascaris0 | 1.19 (-0.24, 2.62) |  |
| 3.2 ascaris1 | 2.50 (-0.44, 5.44) |  |
| 3.3 ascaris2 | 0.84 (-1.75, 3.43) |  |
| **EM_Hookworm_STH vs placebo** | | |
| **Outcome or Subgroup** | **Effect Estimate** | **Test for subgroup differences** |
| 1 Weight | 0.04 (-0.04, 0.12) | Chi² = 0.81, df = 2 (P = 0.67), I² = 0% |
| 1.1 Hookworm0 | 0.02 (-0.08, 0.12) |  |
| 1.2 hookworm1 | 0.05 (-0.13, 0.23) |  |
| 1.3 hookworm2 | 0.16 (-0.13, 0.45) |  |
| 2 Height | 0.12 (-0.03, 0.27) | Chi² = 0.83, df = 2 (P = 0.66), I² = 0% |
| 2.1 hookworm0 | 0.06 (-0.14, 0.26) |  |
| 2.2 hookworm1 | 0.20 (-0.13, 0.53) |  |
| 2.3 hookworm2 | 0.20 (-0.11, 0.51) |  |
| 3 Hemoglobin | 0.25 (-0.58, 1.08) | Chi² = 3.81, df = 2 (P = 0.15), I² = 47.6% |
| 3.1 hookworm0 | 0.07 (-0.91, 1.05) |  |
| 3.2 hookworm1 | -0.04 (-1.80, 1.72) |  |
| 3.3 hookworm2 | 3.58 (0.13, 7.03) |  |
| **EM_Hookworm_ Any_STH + PZQ vs placebo** | | |
| **Outcome or Subgroup** | **Effect Estimate** | **Test for subgroup differences** |
| 1 Weight | 0.03 (-0.14, 0.19) | Chi² = 0.05, df = 2 (P = 0.98), I² = 0% |
| 1.1 hookworm0 | 0.00 (-0.33, 0.33) |  |
| 1.2 hookworm1 | 0.03 (-0.21, 0.27) |  |
| 1.3 hookworm2 | 0.05 (-0.26, 0.36) |  |
| 2 Height | -0.08 (-0.33, 0.16) | Chi² = 0.22, df = 2 (P = 0.90), I² = 0% |
| 2.1 hookworm0 | -0.13 (-0.62, 0.36) |  |
| 2.2 hookworm1 | -0.12 (-0.49, 0.25) |  |
| 2.3 hookworm2 | 0.00 (-0.43, 0.43) |  |
| 3 Hemoglobin | 1.77 (0.39, 3.14) | Chi² = 1.73, df = 2 (P = 0.42), I² = 0% |
| 3.1 hookworm0 | 0.59 (-1.72, 2.90) |  |
| 3.2 hookworm1 | 2.06 (-0.29, 4.41) |  |
| 3.3 hookworm2 | 2.81 (0.32, 5.30) |  |
| **EM_Hookworm_ Any STH + PZQ + iron or MCN vs placebo** | | |
| **Outcome or Subgroup** | **Effect Estimate** | **Test for subgroup differences** |
| 1 Weight | -0.05 (-0.28, 0.17) | Chi² = 0.80, df = 2 (P = 0.67), I² = 0% |
| 1.1 hookworm0 | -0.04 (-0.39, 0.31) |  |
| 1.2 hookworm1 | -0.12 (-0.43, 0.19) |  |
| 1.3 hookworm2 | 0.23 (-0.48, 0.94) |  |
| 2 Height | -0.06 (-0.38, 0.25) | Chi² = 0.26, df = 2 (P = 0.88), I² = 0% |
| 2.1 hookworm0 | -0.14 (-0.67, 0.39) |  |
| 2.2 hookworm1 | 0.02 (-0.43, 0.47) |  |
| 2.3 hookworm2 | -0.16 (-1.00, 0.68) |  |
| 3 Hemoglobin | 2.64 (0.87, 4.41) | Chi² = 0.88, df = 2 (P = 0.64), I² = 0% |
| 3.1 hookworm0 | 1.98 (-0.65, 4.61) |  |
| 3.2 hookworm1 | 2.75 (0.01, 5.49) |  |
| 3.3 hookworm2 | 4.63 (-0.29, 9.55) |  |
| **EM_Hookworm_ Any STH +MCN or iron vs placebo** | | |
| **Outcome or Subgroup** | **Effect Estimate** | **Test for subgroup differences** |
| 1 Weight | -0.00 (-0.14, 0.13) | Chi² = 0.76, df = 2 (P = 0.68), I² = 0% |
| 1.1 hookworm0 | -0.04 (-0.20, 0.12) |  |
| 1.2 hookworm1 | 0.10 (-0.19, 0.39) |  |
| 1.3 hookworm2 | 0.09 (-0.56, 0.74) |  |
| 2 Height | 0.96 (0.76, 1.21) | Chi² = 0.65, df = 2 (P = 0.72), I² = 0% |
| 2.1 hookworm0 | 0.91 (0.69, 1.20) |  |
| 2.2 hookworm1 | 1.16 (0.68, 1.97) |  |
| 2.3 hookworm2 | 0.89 (0.37, 2.14) |  |
| 3 Hemoglobin | 2.00 (0.81, 3.20) | Chi² = 1.60, df = 2 (P = 0.45), I² = 0% |
| 3.1 hookworm0 | 1.94 (0.59, 3.29) |  |
| 3.2 hookworm1 | 1.43 (-1.39, 4.25) |  |
| 3.3 hookworm2 | 5.46 (-0.20, 11.12) |  |
| **EM_Hookworm_ MCN or iron vs placebo** | | |
| **Outcome or Subgroup** | **Effect Estimate** | **Test for subgroup differences** |
| 1 Weight | -0.01 (-0.15, 0.12) | Chi² = 0.11, df = 2 (P = 0.95), I² = 0% |
| 1.1 hookworm0 | 0.00 (-0.16, 0.16) |  |
| 1.2 hookworm1 | -0.05 (-0.30, 0.20) |  |
| 1.3 hookworm2 | -0.04 (-0.78, 0.70) |  |
| 2 Height | -0.09 (-0.31, 0.13) | Chi² = 0.39, df = 2 (P = 0.82), I² = 0% |
| 2.1 hookworm0 | -0.10 (-0.37, 0.17) |  |
| 2.2 hookworm1 | -0.03 (-0.42, 0.36) |  |
| 2.3 hookworm2 | -0.36 (-1.34, 0.62) |  |
| 3 Hemoglobin | 1.09 (-0.06, 2.23) | Chi² = 0.61, df = 2 (P = 0.74), I² = 0% |
| 3.1 hookworm0 | 0.80 (-0.55, 2.15) |  |
| 3.2 hookworm1 | 1.76 (-0.53, 4.05) |  |
| 3.3 hookworm2 | 2.05 (-3.83, 7.93) |  |
| **EM_Trichuris_STH vs placebo** | | |
| **Outcome or Subgroup** | **Effect Estimate** | **Test for subgroup differences** |
| 1 Weight | 0.03 (-0.06, 0.12) | Chi² = 1.82, df = 2 (P = 0.40), I² = 0% |
| 1.1 trichuris0 | -0.01 (-0.13, 0.11) |  |
| 1.2 trichuris1 | 0.04 (-0.12, 0.20) |  |
| 1.3 trichuris2 | 0.17 (-0.07, 0.41) |  |
| 2 Height | 0.09 (-0.06, 0.23) | Chi² = 2.03, df = 2 (P = 0.36), I² = 1.6% |
| 2.1 trichuris0 | 0.02 (-0.18, 0.22) |  |
| 2.2 trichuris1 | 0.30 (-0.03, 0.63) |  |
| 2.3 trichuris2 | 0.07 (-0.20, 0.34) |  |
| 3 Hemoglobin | 0.33 (-0.51, 1.17) | Chi² = 0.72, df = 2 (P = 0.70), I² = 0% |
| 3.1 trichuris0 | 0.17 (-0.97, 1.31) |  |
| 3.2 trichuris1 | 0.24 (-1.21, 1.69) |  |
| 3.3 trichuris2 | 1.33 (-1.14, 3.80) |  |
| **EM_Trichuris_ Any_STH + PZQ vs placebo** | | |
| **Outcome or Subgroup** | **Effect Estimate** | **Test for subgroup differences** |
| 1 Weight | 0.04 (-0.12, 0.20) | Chi² = 0.40, df = 2 (P = 0.82), I² = 0% |
| 1.1 trichuris0 | 0.03 (-0.21, 0.27) |  |
| 1.2 trichuris1 | 0.00 (-0.25, 0.25) |  |
| 1.3 trichuris2 | 0.15 (-0.24, 0.54) |  |
| 2 Height | -0.08 (-0.33, 0.17) | Chi² = 2.31, df = 2 (P = 0.32), I² = 13.3% |
| 2.1 trichuris0 | -0.26 (-0.63, 0.11) |  |
| 2.2 trichuris1 | 0.18 (-0.25, 0.61) |  |
| 2.3 trichuris2 | -0.11 (-0.64, 0.42) |  |
| 3 Hemoglobin | 1.71 (0.43, 2.99) | Chi² = 0.98, df = 2 (P = 0.61), I² = 0% |
| 1 trichuris0 | 1.15 (-0.67, 2.97) |  |
| 3.2 trichuris1 | 1.97 (-0.13, 4.07) |  |
| 3.3 trichuris2 | 3.05 (-0.48, 6.58) |  |
| **EM_Trichuris_ Any STH + PZQ + iron or MCN vs placebo** | | |
| **Outcome or Subgroup** | **Effect Estimate** | **Test for subgroup differences** |
| 1 Weight | -0.02 (-0.24, 0.20) | Chi² = 3.09, df = 2 (P = 0.21), I² = 35.3% |
| 1.1 trichuris0 | 0.09 (-0.18, 0.36) |  |
| 1.2 trichuris1 | -0.33 (-0.74, 0.08) |  |
| 1.3 trichuris2 | 0.23 (-0.67, 1.13) |  |
| 2 Height | -0.09 (-0.42, 0.24) | Chi² = 0.48, df = 2 (P = 0.79), I² = 0% |
| 2.1 trichuris0 | -0.08 (-0.49, 0.33) |  |
| 2.2 trichuris1 | -0.21 (-0.84, 0.42) |  |
| 2.3 trichuris2 | 0.27 (-0.95, 1.49) |  |
| 3 Hemoglobin | 2.44 (0.75, 4.12) | Chi² = 3.67, df = 2 (P = 0.16), I² = 45.6% |
| 3.1 trichuris0 | 1.27 (-0.83, 3.37) |  |
| 3.2 trichuris1 | 4.23 (1.21, 7.25) |  |
| 3.3 trichuris2 | 6.76 (-1.22, 14.74) |  |
| **EM_Trichuris_ Any STH +MCN or iron vs placebo** | | |
| **Outcome or Subgroup** | **Effect Estimate** | **Test for subgroup differences** |
| 1 Weight | 0.01 (-0.13, 0.14) | Chi² = 0.64, df = 2 (P = 0.73), I² = 0% |
| 1.1 trichuris0 | -0.04 (-0.22, 0.14) |  |
| 1.2 trichuris1 | 0.06 (-0.18, 0.30) |  |
| 1.3 trichuris2 | 0.09 (-0.30, 0.48) |  |
| 2 Height | -0.05 (-0.28, 0.18) | Chi² = 0.60, df = 2 (P = 0.74), I² = 0% |
| 2.1 trichuris0 | -0.12 (-0.43, 0.19) |  |
| 2.2 trichuris1 | 0.09 (-0.34, 0.52) |  |
| 2.3 trichuris2 | -0.06 (-0.57, 0.45) |  |
| 3 Hemoglobin | 2.12 (0.94, 3.31) | Chi² = 0.60, df = 2 (P = 0.74), I² = 0% |
| 3.1 trichuris0 | 1.85 (0.24, 3.46) |  |
| 3.2 trichuris1 | 2.05 (0.01, 4.09) |  |
| 3.3 trichuris2 | 3.57 (0.12, 7.02) |  |
| **EM_Trichuris_ MCN or iron vs placebo** | | |
| **Outcome or Subgroup** | **Effect Estimate** | **Test for subgroup differences** |
| 1 Weight | -0.02 (-0.15, 0.11) | Chi² = 1.79, df = 2 (P = 0.41), I² = 0% |
| 1.1 trichuris0 | -0.09 (-0.27, 0.09) |  |
| 1.2 trichuris1 | 0.11 (-0.13, 0.35) |  |
| 1.3 trichuris2 | -0.04 (-0.47, 0.39) |  |
| 2 Height | -0.10 (-0.32, 0.12) | Chi² = 1.09, df = 2 (P = 0.58), I² = 0% |
| 2.1 trichuris0 | -0.17 (-0.46, 0.12) |  |
| 2.2 trichuris1 | 0.08 (-0.33, 0.49) |  |
| 2.3 trichuris2 | -0.21 (-0.80, 0.38) |  |
| 3 Hemoglobin | 1.14 (-0.02, 2.31) | Chi² = 2.85, df = 2 (P = 0.24), I² = 29.9% |
| 3.1 trichuris0 | 0.37 (-1.12, 1.86) |  |
| 3.2 trichuris1 | 2.10 (-0.04, 4.24) |  |
| 3.3 trichuris2 | 3.12 (-0.66, 6.90) |  |
| **EM_Anyworm_STH vs placebo** | | |
| **Outcome or Subgroup** | **Effect Estimate** | **Test for subgroup differences** |
| 1 Weight | 0.03 (-0.05, 0.11) | Chi² = 0.91, df = 2 (P = 0.63), I² = 0% |
| 1.1 anyworm0 | 0.02 (-0.10, 0.14) |  |
| 1.2 anyworm1 | 0.01 (-0.13, 0.15) |  |
| 1.3 anyworm2 | 0.12 (-0.08, 0.32) |  |
| 2 Height | 0.07 (-0.07, 0.21) | Chi² = 0.59, df = 2 (P = 0.74), I² = 0% |
| 2.1 anyworm0 | 0.06 (-0.16, 0.28) |  |
| 2.2 anyworm1 | 0.16 (-0.11, 0.43) |  |
| 2.3 anyworm2 | 0.02 (-0.22, 0.26) |  |
| 3 Hemoglobin | 0.30 (-0.53, 1.13) | Chi² = 0.79, df = 2 (P = 0.67), I² = 0% |
| 3.1 anyworm0 | 0.21 (-1.04, 1.46) |  |
| 3.2 anyworm1 | 0.63 (-0.62, 1.88) |  |
| 3.3 anyworm2 | -0.55 (-2.90, 1.80) |  |
| **EM_Anyworm_ Any_STH + PZQ vs placebo** | | |
| **Outcome or Subgroup** | **Effect Estimate** | **Test for subgroup differences** |
| 1 Weight | 0.02 (-0.14, 0.19) | Chi² = 0.23, df = 2 (P = 0.89), I² = 0% |
| 1.1 anyworm0 | 0.07 (-0.42, 0.56) |  |
| 1.2 anyworm1 | 0.04 (-0.16, 0.24) |  |
| 1.3 anyworm2 | -0.05 (-0.40, 0.30) |  |
| 2 Height | -0.08 (-0.32, 0.17) | Chi² = 0.38, df = 2 (P = 0.83), I² = 0% |
| 2.1 anyworm0 | -0.27 (-0.94, 0.40) |  |
| 2.2 anyworm1 | -0.04 (-0.37, 0.29) |  |
| 2.3 anyworm2 | -0.06 (-0.49, 0.37) |  |
| 3 Hemoglobin | 1.81 (0.46, 3.16) | Chi² = 1.11, df = 2 (P = 0.57), I² = 0% |
| 3.1 anyworm0 | 0.78 (-2.51, 4.07) |  |
| 3.2 anyworm1 | 1.66 (-0.05, 3.37) |  |
| 3.3 anyworm2 | 3.08 (0.12, 6.04) |  |
| **EM_Anyworm_ Any STH + PZQ + iron or MCN vs placebo** | | |
| **Outcome or Subgroup** | **Effect Estimate** | **Test for subgroup differences** |
| 1 Weight | -0.04 (-0.27, 0.19) | Chi² = 0.70, df = 2 (P = 0.70), I² = 0% |
| 1.1 anyworm0 | 0.10 (-0.37, 0.57) |  |
| 1.2 anyworm1 | -0.06 (-0.33, 0.21) |  |
| 1.3 anyworm2 | -0.28 (-1.08, 0.52) |  |
| 2 Height | -0.08 (-0.41, 0.26) | Chi² = 0.08, df = 2 (P = 0.96), I² = 0% |
| 2.1 anyworm0 | 0.01 (-0.68, 0.70) |  |
| 2.2 anyworm1 | -0.10 (-0.51, 0.31) |  |
| 2.3 anyworm2 | -0.12 (-1.10, 0.86) |  |
| 3 Hemoglobin | 2.59 (0.89, 4.29) | Chi² = 4.72, df = 2 (P = 0.09), I² = 57.6% |
| 3.1 anyworm0 | 1.48 (-1.95, 4.91) |  |
| 3.2 anyworm1 | 2.28 (0.22, 4.34) |  |
| 3.3 anyworm2 | 9.21 (2.90, 15.52) |  |
| **EM_Anyworm_ Any STH +MCN or iron vs placebo** | | |
| **Outcome or Subgroup** | **Effect Estimate** | **Test for subgroup differences** |
| 1 Weight | -0.01 (-0.14, 0.13) | Chi² = 1.23, df = 2 (P = 0.54), I² = 0% |
| 1.1 anyworm0 | 0.06 (-0.16, 0.28) |  |
| 1.2 anyworm1 | -0.10 (-0.32, 0.12) |  |
| 1.3 amyworm2 | 0.05 (-0.24, 0.34) |  |
| 2 Height | -0.06 (-0.27, 0.15) | Chi² = 0.20, df = 2 (P = 0.90), I² = 0% |
| 2.1 anyworm0 | -0.13 (-0.52, 0.26) |  |
| 2.2 anyworm1 | -0.01 (-0.36, 0.34) |  |
| 2.3 anyworm2 | -0.05 (-0.40, 0.30) |  |
| 3 Hemoglobin | 2.21 (1.05, 3.37) | Chi² = 0.55, df = 2 (P = 0.76), I² = 0% |
| 3.1 anyworm0 | 2.75 (0.69, 4.81) |  |
| 3.2 anyworm1 | 1.78 (0.13, 3.43) |  |
| 3.3 anyworm2 | 2.43 (-0.24, 5.10) |  |
| **EM_Anyworm_ MCN or iron vs placebo** | | |
| **Outcome or Subgroup** | **Effect Estimate** | **Test for subgroup differences** |
| 1 Weight | -0.00 (-0.15, 0.14) | Chi² = 1.25, df = 2 (P = 0.53), I² = 0% |
| 1.1 anyworm0 | 0.09 (-0.16, 0.34) |  |
| 1.2 anyworm1 | -0.08 (-0.28, 0.12) |  |
| 1.3 anyworm2 | 0.07 (-0.30, 0.44) |  |
| 2 Height | -0.10 (-0.33, 0.12) | Chi² = 0.09, df = 2 (P = 0.95), I² = 0% |
| 2.1 anyworm0 | -0.14 (-0.59, 0.31) |  |
| 2.2 anyworm1 | -0.07 (-0.38, 0.24) |  |
| 2.3 anyworm2 | -0.14 (-0.59, 0.31) |  |
| 3 Hemoglobin | 1.14 (-0.08, 2.36) | Chi² = 1.23, df = 2 (P = 0.54), I² = 0% |
| 3.1 anyworm0 | 0.24 (-1.94, 2.42) |  |
| 3.2 anyworm1 | 1.77 (0.08, 3.46) |  |
| 3.3 anyworm2 | 0.83 (-2.25, 3.91) |  |
| **EM_Anaemic_STH vs placebo** | | |
| **Outcome or Subgroup** | **Effect Estimate** | **Test for subgroup differences** |
| 1 Weight | 0.02 (-0.07, 0.11) | Chi² = 0.58, df = 1 (P = 0.45), I² = 0% |
| 1.1 Anaemic | -0.01 (-0.13, 0.11) |  |
| 1.2 Nonanaemic | 0.06 (-0.08, 0.20) |  |
| 2 Height | 0.09 (-0.08, 0.25) | Chi² = 0.00, df = 1 (P = 0.95), I² = 0% |
| 2.1 Anaemic | 0.08 (-0.16, 0.32) |  |
| 2.2 Nonanaemic | 0.09 (-0.15, 0.33) |  |
| 3 Hemoglobin | 0.19 (-0.65, 1.03) | Chi² = 0.94, df = 1 (P = 0.33), I² = 0% |
| 3.1 Anaemic | 0.66 (-0.61, 1.93) |  |
| 3.2 Nonanaemic | -0.18 (-1.30, 0.94) |  |
| **EM_Anaemic_STH + PZQ vs placebo** | | |
| **Outcome or Subgroup** | **Effect Estimate** | **Test for subgroup differences** |
| 1 Weight | 0.04 (-0.12, 0.19) | Chi² = 0.30, df = 1 (P = 0.58), I² = 0% |
| 1.1 Anaemic | 0.07 (-0.13, 0.27) |  |
| 1.2 nonanaemic | -0.02 (-0.27, 0.23) |  |
| 2 Height | -0.07 (-0.32, 0.17) | Chi² = 0.44, df = 1 (P = 0.50), I² = 0% |
| 2.1 anaemic | -0.15 (-0.48, 0.18) |  |
| 2.2 nonanaemic | 0.02 (-0.35, 0.39) |  |
| 3 Hemoglobin | 1.86 (0.56, 3.15) | Chi² = 1.65, df = 1 (P = 0.20), I² = 39.5% |
| 3.1 anaemic | 2.66 (0.88, 4.44) |  |
| 3.2 nonanaemic | 0.96 (-0.92, 2.84) |  |
| **EM_Anaemic_ Any STH + PZQ + iron or MCN vs placebo** | | |
| **Outcome or Subgroup** | **Effect Estimate** | **Test for subgroup differences** |
| 1 Weight | -0.05 (-0.27, 0.17) | Chi² = 0.00, df = 1 (P = 1.00), I² = 0% |
| 1.1 anaemic | -0.05 (-0.36, 0.26) |  |
| 1.2 nonanaemic | -0.05 (-0.36, 0.26) |  |
| 2 Height | -0.06 (-0.39, 0.27) | Chi² = 0.00, df = 1 (P = 0.95), I² = 0% |
| 2.1 anaemic | -0.05 (-0.50, 0.40) |  |
| 2.2 nonanaemic | -0.07 (-0.54, 0.40) |  |
| 3 Hemoglobin | 2.70 (1.05, 4.34) | Chi² = 0.78, df = 1 (P = 0.38), I² = 0% |
| 3.1 anaemic | 3.57 (1.02, 6.12) |  |
| 3.2 nonanaemic | 2.07 (-0.09, 4.23) |  |
| **EM_Anaemic_ Any STH +MCN or iron vs placebo** | | |
| **Outcome or Subgroup** | **Effect Estimate** | **Test for subgroup differences** |
| 1 Weight | -0.01 (-0.14, 0.13) | Chi² = 0.01, df = 1 (P = 0.94), I² = 0% |
| 1.1 anaemic | -0.01 (-0.19, 0.17) |  |
| 1.2 nonanaemic | 0.00 (-0.20, 0.20) |  |
| 2 Height | -0.04 (-0.27, 0.19) | Chi² = 0.09, df = 1 (P = 0.76), I² = 0% |
| 2.1 anaemic | -0.07 (-0.38, 0.24) |  |
| 2.2 nonanaemic | 0.00 (-0.33, 0.33) |  |
| 3 Hemoglobin | 2.06 (0.91, 3.21) | Chi² = 0.54, df = 1 (P = 0.46), I² = 0% |
| 3.1 anaemic | 2.54 (0.82, 4.26) |  |
| 3.2 nonanaemic | 1.67 (0.12, 3.22) |  |
| **EM_Anaemic_ Any MCN or iron vs placebo** | | |
| **Outcome or Subgroup** | **Effect Estimate** | **Test for subgroup differences** |
| 1 Weight | -0.02 (-0.17, 0.12) | Chi² = 0.07, df = 1 (P = 0.79), I² = 0% |
| 1.1 anaemic | -0.04 (-0.24, 0.16) |  |
| 1.2 nonanaemic | 0.00 (-0.22, 0.22) |  |
| 2 Height | -0.09 (-0.31, 0.13) | Chi² = 0.28, df = 1 (P = 0.60), I² = 0% |
| 2.1 anaemic | -0.15 (-0.46, 0.16) |  |
| 2.2 nonanaemic | -0.03 (-0.34, 0.28) |  |
| 3 Hemoglobin | 1.31 (0.14, 2.48) | Chi² = 0.53, df = 1 (P = 0.46), I² = 0% |
| 3.1 anaemic | 1.80 (0.04, 3.56) |  |
| 3.2 nonanaemic | 0.92 (-0.65, 2.49) |  |

Head to head comparisons

| **EM_ Baz _ Any STH + PZQ vs Any STH** | | |
| --- | --- | --- |
| **Outcome or Subgroup** | **Effect Estimate** | **Test for subgroup differences** |
| 1 Weight | 0.02 (-0.15, 0.19) | Chi² = 0.14, df = 1 (P = 0.71), I² = 0% |
| 1.1 >-2 | 0.03 (-0.15, 0.21) |  |
| 1.2 <-2 | -0.08 (-0.63, 0.47) |  |
| 2 Height | -0.15 (-0.43, 0.13) | Chi² = 0.06, df = 1 (P = 0.81), I² = 0% |
| 2.1 >-2 | -0.14 (-0.43, 0.15) |  |
| 2.2 <-2 | -0.26 (-1.20, 0.68) |  |
| 3 Hemoglobin | 1.51 (0.00, 3.02) | Chi² = 0.16, df = 1 (P = 0.69), I² = 0% |
| 3.1 >-2 | 1.41 (-0.18, 3.00) |  |
| 3.2 <-2 | 2.45 (-2.39, 7.29) |  |
| **EM_BAZ _ Any STH + PZQ + iron or MCN vs any STH** | | |
| **Outcome or Subgroup** | **Effect Estimate** | **Test for subgroup differences** |
| 1 Weight | -0.04 (-0.29, 0.20) | Chi² = 0.32, df = 1 (P = 0.57), I² = 0% |
| 1.1 >-2 | -0.02 (-0.27, 0.23) |  |
| 1.2 <-2 | -0.28 (-1.14, 0.58) |  |
| 2 Height | -0.09 (-0.43, 0.24) | Chi² = 0.10, df = 1 (P = 0.75), I² = 0% |
| 2.1 >-2 | -0.11 (-0.46, 0.24) |  |
| 2.2 <-2 | 0.09 (-1.09, 1.27) |  |
| 3 Hemoglobin | 2.39 (0.48, 4.31) | Chi² = 0.01, df = 1 (P = 0.91), I² = 0% |
| 3.1 >-2 | 2.36 (0.36, 4.36) |  |
| 3.2 <-2 | 2.75 (-3.82, 9.32) |  |
| **EM_BAZ_Any STH + MCN or iron vs any STH** | | |
| **Outcome or Subgroup** | **Effect Estimate** | **Test for subgroup differences** |
| 1 Weight | -0.02 (-0.16, 0.12) | Chi² = 0.09, df = 1 (P = 0.77), I² = 0% |
| 1.1 >-2 | -0.03 (-0.19, 0.13) |  |
| 1.2 <-2 | 0.02 (-0.27, 0.31) |  |
| 2 Height | -0.05 (-0.30, 0.20) | Chi² = 0.01, df = 1 (P = 0.91), I² = 0% |
| 2.1 >-2 | -0.06 (-0.33, 0.21) |  |
| 2.2 <-2 | -0.02 (-0.63, 0.59) |  |
| 3 Hemoglobin | 1.84 (0.54, 3.14) | Chi² = 0.00, df = 1 (P = 0.96), I² = 0% |
| 3.1 >-2 | 1.82 (0.35, 3.29) |  |
| 3.2 <-2 | 1.90 (-0.86, 4.66) |  |
| **EM_BAZ_ MCN or iron alone vs any STH** | | |
| **Outcome or Subgroup** | **Effect Estimate** | **Test for subgroup differences** |
| 1 Weight | -0.03 (-0.18, 0.12) | Chi² = 1.04, df = 1 (P = 0.31), I² = 4.1% |
| 1.1 >-2 | 0.00 (-0.16, 0.16) |  |
| 1.2 <-2 | -0.22 (-0.61, 0.17) |  |
| 2 Height | -0.13 (-0.37, 0.10) | Chi² = 0.06, df = 1 (P = 0.81), I² = 0% |
| 2.1 >-2 | -0.12 (-0.37, 0.13) |  |
| 2.2 <-2 | -0.20 (-0.81, 0.41) |  |
| 3 Hemoglobin | 1.10 (-0.21, 2.42) | Chi² = 1.79, df = 1 (P = 0.18), I² = 44.2% |
| 3.1 >-2 | 0.72 (-0.71, 2.15) |  |
| 3.2 <-2 | 3.21 (-0.14, 6.56) |  |
| **EM_BAZ_ Any STH + PZQ + iron or MCN vs Any STH + PZQ** | | |
| **Outcome or Subgroup** | **Effect Estimate** | **Test for subgroup differences** |
| 1 Weight | -0.06 (-0.29, 0.17) | Chi² = 0.10, df = 1 (P = 0.75), I² = 0% |
| 1.1 >-2 | -0.05 (-0.29, 0.19) |  |
| 1.2 <-2 | -0.20 (-1.08, 0.68) |  |
| 2 Height | 0.05 (-0.28, 0.39) | Chi² = 0.23, df = 1 (P = 0.63), I² = 0% |
| 2.1 >-2 | 0.03 (-0.32, 0.38) |  |
| 2.2 <-2 | 0.34 (-0.88, 1.56) |  |
| 3 Hemoglobin | 0.89 (-0.98, 2.76) | Chi² = 0.03, df = 1 (P = 0.86), I² = 0% |
| 3.1 >-2 | 0.94 (-1.00, 2.88) |  |
| 3.2 <-2 | 0.30 (-6.68, 7.28) |  |
| **EM_Baz_ Any STH with MCN or iron vs Any STH + PZQ** | | |
| **Outcome or Subgroup** | **Effect Estimate** | **Test for subgroup differences** |
| 1 Weight | -0.04 (-0.24, 0.16) | Chi² = 0.28, df = 1 (P = 0.59), I² = 0% |
| 1.1 >-2 | -0.06 (-0.28, 0.16) |  |
| 1.2 <-2 | 0.10 (-0.45, 0.65) |  |
| 2 Height | 0.10 (-0.23, 0.43) | Chi² = 0.10, df = 1 (P = 0.75), I² = 0% |
| 2.1 >-2 | 0.08 (-0.27, 0.43) |  |
| 2.2 <-2 | 0.24 (-0.68, 1.16) |  |
| 3 Hemoglobin | 0.29 (-1.43, 2.02) | Chi² = 0.13, df = 1 (P = 0.72), I² = 0% |
| 3.1 >-2 | 0.41 (-1.43, 2.25) |  |
| 3.2 <-2 | -0.56 (-5.54, 4.42) |  |
| **EM_Baz_ MCN or iron alone vs Any STH + PZQ** | | |
| **Outcome or Subgroup** | **Effect Estimate** | **Test for subgroup differences** |
| 1 Weight | -0.04 (-0.21, 0.13) | Chi² = 0.12, df = 1 (P = 0.73), I² = 0% |
| 1.1 >-2 | -0.03 (-0.21, 0.15) |  |
| 1.2 <-2 | -0.14 (-0.73, 0.45) |  |
| 2 Height | 0.02 (-0.26, 0.30) | Chi² = 0.01, df = 1 (P = 0.94), I² = 0% |
| 2.1 >-2 | 0.02 (-0.27, 0.31) |  |
| 2.2 <-2 | 0.06 (-0.86, 0.98) |  |
| 3 Hemoglobin | -0.57 (-2.06, 0.93) | Chi² = 0.30, df = 1 (P = 0.58), I² = 0% |
| 3.1 >-2 | -0.70 (-2.27, 0.87) |  |
| 3.2 <-2 | 0.76 (-4.18, 5.70) |  |
| **EM_Baz_ Any STH with MCN or iron vs Any STH+PZQ + iron/MCN** | | |
| **Outcome or Subgroup** | **Effect Estimate** | **Test for subgroup differences** |
| 1 Weight | 0.02 (-0.24, 0.28) | Chi² = 0.50, df = 1 (P = 0.48), I² = 0% |
| 1.1 >-2 | -0.01 (-0.28, 0.26) |  |
| 1.2 <-2 | 0.31 (-0.53, 1.15) |  |
| 2 Height | 0.03 (-0.36, 0.42) | Chi² = 0.05, df = 1 (P = 0.83), I² = 0% |
| 2.1 >-2 | 0.04 (-0.37, 0.45) |  |
| 2.2 <-2 | -0.10 (-1.32, 1.12) |  |
| 3 Hemoglobin | -0.57 (-2.62, 1.48) | Chi² = 0.01, df = 1 (P = 0.93), I² = 0% |
| 3.1 >-2 | -0.54 (-2.70, 1.62) |  |
| 3.2 <-2 | -0.86 (-7.50, 5.78) |  |
| **EM_Baz_ MCN or iron alone vs Any STH + PZQ + iron/MCN** | | |
| **Outcome or Subgroup** | **Effect Estimate** | **Test for subgroup differences** |
| 1 Weight | 0.02 (-0.20, 0.25) | Chi² = 0.01, df = 1 (P = 0.91), I² = 0% |
| 1.1 >-2 | 0.02 (-0.22, 0.26) |  |
| 1.2 <-2 | 0.07 (-0.75, 0.89) |  |
| 2 Height | -0.03 (-0.37, 0.31) | Chi² = 0.19, df = 1 (P = 0.67), I² = 0% |
| 2.1 >-2 | -0.01 (-0.36, 0.34) |  |
| 2.2 <-2 | -0.28 (-1.46, 0.90) |  |
| 3 Hemoglobin | -1.47 (-3.34, 0.41) | Chi² = 0.36, df = 1 (P = 0.55), I² = 0% |
| 3.1 >-2 | -1.64 (-3.60, 0.32) |  |
| 3.2 <-2 | 0.46 (-6.07, 6.99) |  |
| **EM_Baz_ MCN or iron alone vs Any STH with MCN or iron** | | |
| **Outcome or Subgroup** | **Effect Estimate** | **Test for subgroup differences** |
| 1 Weight | -0.02 (-0.18, 0.15) | Chi² = 1.52, df = 1 (P = 0.22), I² = 34.0% |
| 1.1 >-2 | 0.03 (-0.15, 0.21) |  |
| 1.2 <-2 | -0.24 (-0.63, 0.15) |  |
| 2 Height | -0.07 (-0.34, 0.20) | Chi² = 0.13, df = 1 (P = 0.72), I² = 0% |
| 2.1 >-2 | -0.05 (-0.34, 0.24) |  |
| 2.2 <-2 | -0.18 (-0.83, 0.47) |  |
| 3 Hemoglobin | -0.70 (-2.08, 0.68) | Chi² = 1.62, df = 1 (P = 0.20), I² = 38.3% |
| 3.1 >-2 | -1.10 (-2.61, 0.41) |  |
| 3.2 <-2 | 1.31 (-2.08, 4.70) |  |
| **EM_Haz_ Any STH + PZQ vs Any STH** | | |
| **Outcome or Subgroup** | **Effect Estimate** | **Test for subgroup differences** |
| 1 Weight | 0.02 (-0.15, 0.19) | Chi² = 0.14, df = 1 (P = 0.71), I² = 0% |
| 1.1 >-2 | 0.03 (-0.15, 0.21) |  |
| 1.2 <-2 | -0.08 (-0.63, 0.47) |  |
| 2 Height | -0.15 (-0.43, 0.13) | Chi² = 0.06, df = 1 (P = 0.81), I² = 0% |
| 2.1 >-2 | -0.14 (-0.43, 0.15) |  |
| 2.2 <-2 | -0.26 (-1.20, 0.68) |  |
| 3 Hemoglobin | 1.51 (0.00, 3.02) | Chi² = 0.16, df = 1 (P = 0.69), I² = 0% |
| 3.1 >-2 | 1.41 (-0.18, 3.00) |  |
| 3.2 <-2 | 2.45 (-2.39, 7.29) |  |
| **EM_Haz_ Any STH + PZQ + iron or MCN vs any STH** | | |
| **Outcome or Subgroup** | **Effect Estimate** | **Test for subgroup differences** |
| 1 Weight | -0.04 (-0.29, 0.20) | Chi² = 0.32, df = 1 (P = 0.57), I² = 0% |
| 1.1 >-2 | -0.02 (-0.27, 0.23) |  |
| 1.2 <-2 | -0.28 (-1.14, 0.58) |  |
| 2 Height | -0.09 (-0.43, 0.24) | Chi² = 0.10, df = 1 (P = 0.75), I² = 0% |
| 2.1 >-2 | -0.11 (-0.46, 0.24) |  |
| 2.2 <-2 | 0.09 (-1.09, 1.27) |  |
| 3 Hemoglobin | 2.39 (0.48, 4.31) | Chi² = 0.01, df = 1 (P = 0.91), I² = 0% |
| 3.1 >-2 | 2.36 (0.36, 4.36) |  |
| 3.2 <-2 | 2.75 (-3.82, 9.32) |  |
| **EM_Haz_ Any STH + MCN or iron vs any STH** | | |
| **Outcome or Subgroup** | **Effect Estimate** | **Test for subgroup differences** |
| 1 Weight | -0.02 (-0.16, 0.12) | Chi² = 0.09, df = 1 (P = 0.77), I² = 0% |
| 1.1 >-2 | -0.03 (-0.19, 0.13) |  |
| 1.2 <-2 | 0.02 (-0.27, 0.31) |  |
| 2 Height | -0.05 (-0.30, 0.20) | Chi² = 0.01, df = 1 (P = 0.91), I² = 0% |
| 2.1 >-2 | -0.06 (-0.33, 0.21) |  |
| 2.2 <-2 | -0.02 (-0.63, 0.59) |  |
| 3 Hemoglobin | 1.84 (0.54, 3.14) | Chi² = 0.00, df = 1 (P = 0.96), I² = 0% |
| 3.1 >-2 | 1.82 (0.35, 3.29) |  |
| 3.2 <-2 | 1.90 (-0.86, 4.66) |  |
| **EM_Haz_ MCN or iron alone vs any STH-** | | |
| **Outcome or Subgroup** | **Effect Estimate** | **Test for subgroup differences** |
| 1 Weight | -0.03 (-0.18, 0.12) | Chi² = 1.04, df = 1 (P = 0.31), I² = 4.1% |
| 1.1 >-2 | 0.00 (-0.16, 0.16) |  |
| 1.2 <-2 | -0.22 (-0.61, 0.17) |  |
| 2 Height | -0.13 (-0.37, 0.10) | Chi² = 0.06, df = 1 (P = 0.81), I² = 0% |
| 2.1 >-2 | -0.12 (-0.37, 0.13) |  |
| 2.2 <-2 | -0.20 (-0.81, 0.41) |  |
| 3 Hemoglobin | 1.10 (-0.21, 2.42) | Chi² = 1.79, df = 1 (P = 0.18), I² = 44.2% |
| 3.1 >-2 | 0.72 (-0.71, 2.15) |  |
| 3.2 <-2 | 3.21 (-0.14, 6.56) |  |
| **EM_Haz_ Any STH + PZQ + iron or MCN vs Any STH + PZQ** | | |
| **Outcome or Subgroup** | **Effect Estimate** | **Test for subgroup differences** |
| 1 Weight | -0.06 (-0.29, 0.17) | Chi² = 0.10, df = 1 (P = 0.75), I² = 0% |
| 1.1 >-2 | -0.05 (-0.29, 0.19) |  |
| 1.2 <-2 | -0.20 (-1.08, 0.68) |  |
| 2 Height | 0.05 (-0.28, 0.39) | Chi² = 0.23, df = 1 (P = 0.63), I² = 0% |
| 2.1 >-2 | 0.03 (-0.32, 0.38) |  |
| 2.2 <-2 | 0.34 (-0.88, 1.56) |  |
| 3 Hemoglobin | 0.89 (-0.98, 2.76) | Chi² = 0.03, df = 1 (P = 0.86), I² = 0% |
| 3.1 >-2 | 0.94 (-1.00, 2.88) |  |
| 3.2 <-2 | 0.30 (-6.68, 7.28) |  |
| **EM_Haz_ Any STH with MCN or iron vs Any STH + PZQ** | | |
| **Outcome or Subgroup** | **Effect Estimate** | **Test for subgroup differences** |
| 1 Weight | -0.04 (-0.24, 0.16) | Chi² = 0.28, df = 1 (P = 0.59), I² = 0% |
| 1.1 >-2 | -0.06 (-0.28, 0.16) |  |
| 1.2 <-2 | 0.10 (-0.45, 0.65) |  |
| 2 Height | 0.10 (-0.23, 0.43) | Chi² = 0.10, df = 1 (P = 0.75), I² = 0% |
| 2.1 >-2 | 0.08 (-0.27, 0.43) |  |
| 2.2 <-2 | 0.24 (-0.68, 1.16) |  |
| 3 Hemoglobin | 0.29 (-1.43, 2.02) | Chi² = 0.13, df = 1 (P = 0.72), I² = 0% |
| 3.1 >-2 | 0.41 (-1.43, 2.25) |  |
| 3.2 <-2 | -0.56 (-5.54, 4.42) |  |
| **EM_Haz_ MCN or iron alone vs Any STH + PZQ** | | |
| **Outcome or Subgroup** | **Effect Estimate** | **Test for subgroup differences** |
| 1 Weight | -0.04 (-0.21, 0.13) | Chi² = 0.12, df = 1 (P = 0.73), I² = 0% |
| 1.1 >-2 | -0.03 (-0.21, 0.15) |  |
| 1.2 <-2 | -0.14 (-0.73, 0.45) |  |
| 2 Height | 0.02 (-0.26, 0.30) | Chi² = 0.01, df = 1 (P = 0.94), I² = 0% |
| 2.1 >-2 | 0.02 (-0.27, 0.31) |  |
| 2.2 <-2 | 0.06 (-0.86, 0.98) |  |
| 3 Hemoglobin | -0.57 (-2.06, 0.93) | Chi² = 0.30, df = 1 (P = 0.58), I² = 0% |
| 3.1 >-2 | -0.70 (-2.27, 0.87) |  |
| 3.2 <-2 | 0.76 (-4.18, 5.70) |  |
| **EM_Haz_ Any STH with MCN or iron vs Any STH+PZQ + iron/MCN** | | |
| **Outcome or Subgroup** | **Effect Estimate** | **Test for subgroup differences** |
| 1 Weight | 0.02 (-0.24, 0.28) | Chi² = 0.50, df = 1 (P = 0.48), I² = 0% |
| 1.1 >-2 | -0.01 (-0.28, 0.26) |  |
| 1.2 <-2 | 0.31 (-0.53, 1.15) |  |
| 2 Height | 0.03 (-0.36, 0.42) | Chi² = 0.05, df = 1 (P = 0.83), I² = 0% |
| 2.1 >-2 | 0.04 (-0.37, 0.45) |  |
| 2.2 <-2 | -0.10 (-1.32, 1.12) |  |
| 3 Hemoglobin | -0.57 (-2.62, 1.48) | Chi² = 0.01, df = 1 (P = 0.93), I² = 0% |
| 3.1 >-2 | -0.54 (-2.70, 1.62) |  |
| 3.2 <-2 | -0.86 (-7.50, 5.78) |  |
| **EM_Haz_ MCN or iron alone vs Any STH + PZQ + iron/MCN** | | |
| **Outcome or Subgroup** | **Effect Estimate** | **Test for subgroup differences** |
| 1 Weight | 0.02 (-0.20, 0.25) | Chi² = 0.01, df = 1 (P = 0.91), I² = 0% |
| 1.1 >-2 | 0.02 (-0.22, 0.26) |  |
| 1.2 <-2 | 0.07 (-0.75, 0.89) |  |
| 2 Height | -0.03 (-0.37, 0.31) | Chi² = 0.19, df = 1 (P = 0.67), I² = 0% |
| 2.1 >-2 | -0.01 (-0.36, 0.34) |  |
| 2.2 <-2 | -0.28 (-1.46, 0.90) |  |
| 3 Hemoglobin | -1.47 (-3.34, 0.41) | Chi² = 0.36, df = 1 (P = 0.55), I² = 0% |
| 3.1 >-2 | -1.64 (-3.60, 0.32) |  |
| 3.2 <-2 | 0.46 (-6.07, 6.99) |  |
| **EM_Haz_ MCN or iron alone vs Any STH with MCN or iron** | | |
| **Outcome or Subgroup** | **Effect Estimate** | **Test for subgroup differences** |
| 1 Weight | -0.02 (-0.18, 0.15) | Chi² = 1.52, df = 1 (P = 0.22), I² = 34.0% |
| 1.1 >-2 | 0.03 (-0.15, 0.21) |  |
| 1.2 <-2 | -0.24 (-0.63, 0.15) |  |
| 2 Height | -0.07 (-0.34, 0.20) | Chi² = 0.13, df = 1 (P = 0.72), I² = 0% |
| 2.1 >-2 | -0.05 (-0.34, 0.24) |  |
| 2.2 <-2 | -0.18 (-0.83, 0.47) |  |
| 3 Hemoglobin | -0.70 (-2.08, 0.68) | Chi² = 1.62, df = 1 (P = 0.20), I² = 38.3% |
| 3.1 >-2 | -1.10 (-2.61, 0.41) |  |
| 3.2 <-2 | 1.31 (-2.08, 4.70) |  |
| **EM_Sex_ Any STH + PZQ vs Any STH** | | |
| **Outcome or Subgroup** | **Effect Estimate** | **Test for subgroup differences** |
| 1 Weight | 0.02 (-0.15, 0.18) | Chi² = 1.52, df = 1 (P = 0.22), I² = 34.2% |
| 1.1 female | 0.14 (-0.11, 0.39) |  |
| 1.2 male | -0.07 (-0.29, 0.15) |  |
| 2 Height | -0.15 (-0.42, 0.12) | Chi² = 0.76, df = 1 (P = 0.38), I² = 0% |
| 2.1 female | -0.04 (-0.41, 0.33) |  |
| 2.2 male | -0.28 (-0.67, 0.11) |  |
| 3 Hemoglobin | 1.50 (-0.01, 3.01) | Chi² = 0.05, df = 1 (P = 0.82), I² = 0% |
| 3.1 female | 1.32 (-0.84, 3.48) |  |
| 3.2 male | 1.68 (-0.44, 3.80) |  |
| **EM_Sex_ Any STH + PZQ + iron or MCN vs any STH** | | |
| **Outcome or Subgroup** | **Effect Estimate** | **Test for subgroup differences** |
| 1 Weight | -0.01 (-0.27, 0.24) | Chi² = 2.08, df = 1 (P = 0.15), I² = 52.0% |
| 1.1 female | -0.25 (-0.66, 0.16) |  |
| 1.2 male | 0.14 (-0.19, 0.47) |  |
| 2 Height | -0.14 (-0.47, 0.19) | Chi² = 0.07, df = 1 (P = 0.79), I² = 0% |
| 2.1 female | -0.10 (-0.55, 0.35) |  |
| 2.2 male | -0.19 (-0.68, 0.30) |  |
| 3 Hemoglobin | 2.48 (0.62, 4.33) | Chi² = 0.10, df = 1 (P = 0.75), I² = 0% |
| 3.1 female | 2.79 (0.12, 5.46) |  |
| 3.2 male | 2.18 (-0.41, 4.77) |  |
| **EM_Sex_ Any STH + MCN or iron vs any STH** | | |
| **Outcome or Subgroup** | **Effect Estimate** | **Test for subgroup differences** |
| 1 Weight | -0.03 (-0.17, 0.11) | Chi² = 0.02, df = 1 (P = 0.89), I² = 0% |
| 1.1 female | -0.02 (-0.22, 0.18) |  |
| 1.2 male | -0.04 (-0.24, 0.16) |  |
| 2 Height | -0.12 (-0.35, 0.12) | Chi² = 0.02, df = 1 (P = 0.90), I² = 0% |
| 2.1 female | -0.13 (-0.44, 0.18) |  |
| 2.2 male | -0.10 (-0.45, 0.25) |  |
| 3 Hemoglobin | 1.86 (0.60, 3.12) | Chi² = 0.04, df = 1 (P = 0.84), I² = 0% |
| 3.1 female | 1.72 (-0.12, 3.56) |  |
| 3.2 male | 1.98 (0.26, 3.70) |  |
| **EM_Sex_ MCN or iron alone vs any STH** | | |
| **Outcome or Subgroup** | **Effect Estimate** | **Test for subgroup differences** |
| 1 Weight | -0.04 (-0.18, 0.11) | Chi² = 0.45, df = 1 (P = 0.50), I² = 0% |
| 1.1 Female | -0.09 (-0.31, 0.13) |  |
| 1.2 male | 0.01 (-0.19, 0.21) |  |
| 2 Height | -0.16 (-0.39, 0.07) | Chi² = 0.03, df = 1 (P = 0.86), I² = 0% |
| 2.1 female | -0.18 (-0.49, 0.13) |  |
| 2.2 male | -0.14 (-0.47, 0.19) |  |
| 3 Hemoglobin | 1.06 (-0.25, 2.37) | Chi² = 0.04, df = 1 (P = 0.85), I² = 0% |
| 3.1 female | 1.18 (-0.60, 2.96) |  |
| 3.2 male | 0.92 (-1.02, 2.86) |  |
| **EM_Sex_ Any STH + PZQ + iron or MCN vs Any STH + PZQ** | | |
| **Outcome or Subgroup** | **Effect Estimate** | **Test for subgroup differences** |
| 1 Weight | -0.03 (-0.27, 0.22) | Chi² = 5.67, df = 1 (P = 0.02), I² = 82.4% |
| 1.1 female | -0.40 (-0.79, -0.01) |  |
| 1.2 male | 0.21 (-0.10, 0.52) |  |
| 2 Height | 0.02 (-0.31, 0.34) | Chi² = 0.23, df = 1 (P = 0.63), I² = 0% |
| 2.1 female | -0.06 (-0.51, 0.39) |  |
| 2.2 male | 0.10 (-0.37, 0.57) |  |
| 3 Hemoglobin | 0.96 (-0.86, 2.77) | Chi² = 0.27, df = 1 (P = 0.60), I² = 0% |
| 3.1 female | 1.47 (-1.18, 4.12) |  |
| 3.2 male | 0.50 (-1.99, 2.99) |  |
| **EM_Sex_ Any STH with MCN or iron vs Any STH + PZQ** | | |
| **Outcome or Subgroup** | **Effect Estimate** | **Test for subgroup differences** |
| 1 Weight | -0.06 (-0.25, 0.12) | Chi² = 1.10, df = 1 (P = 0.30), I² = 8.7% |
| 1.1 female | -0.17 (-0.44, 0.10) |  |
| 1.2 male | 0.03 (-0.22, 0.28) |  |
| 2 Height | 0.04 (-0.27, 0.35) | Chi² = 0.72, df = 1 (P = 0.40), I² = 0% |
| 2.1 female | -0.09 (-0.52, 0.34) |  |
| 2.2 male | 0.18 (-0.27, 0.63) |  |
| 3 Hemoglobin | 0.35 (-1.31, 2.01) | Chi² = 0.00, df = 1 (P = 0.95), I² = 0% |
| 3.1 female | 0.40 (-1.99, 2.79) |  |
| 3.2 male | 0.30 (-2.01, 2.61) |  |
| **EM_Sex_ MCN or iron alone vs Any STH + PZQ** | | |
| **Outcome or Subgroup** | **Effect Estimate** | **Test for subgroup differences** |
| 1 Weight | -0.05 (-0.23, 0.13) | Chi² = 2.83, df = 1 (P = 0.09), I² = 64.6% |
| 1.1 female | -0.23 (-0.50, 0.04) |  |
| 1.2 male | 0.08 (-0.16, 0.32) |  |
| 2 Height | -0.00 (-0.29, 0.28) | Chi² = 0.87, df = 1 (P = 0.35), I² = 0% |
| 2.1 female | -0.13 (-0.52, 0.26) |  |
| 2.2 male | 0.14 (-0.27, 0.55) |  |
| 3 Hemoglobin | -0.46 (-1.98, 1.06) | Chi² = 0.16, df = 1 (P = 0.69), I² = 0% |
| 3.1 female | -0.14 (-2.32, 2.04) |  |
| 3.2 male | -0.76 (-2.88, 1.36) |  |
| **EM_Sex_ Any STH with MCN or iron vs Any STH+PZQ + iron/MCN** | | |
| **Outcome or Subgroup** | **Effect Estimate** | **Test for subgroup differences** |
| 1 Weight | -0.01 (-0.27, 0.26) | Chi² = 2.20, df = 1 (P = 0.14), I² = 54.5% |
| 1.1 female | 0.23 (-0.18, 0.64) |  |
| 1.2 male | -0.18 (-0.53, 0.17) |  |
| 2 Height | 0.03 (-0.34, 0.39) | Chi² = 0.10, df = 1 (P = 0.75), I² = 0% |
| 2.1 female | -0.03 (-0.54, 0.48) |  |
| 2.2 male | 0.09 (-0.44, 0.62) |  |
| 3 Hemoglobin | -0.63 (-2.59, 1.33) | Chi² = 0.19, df = 1 (P = 0.66), I² = 0% |
| 3.1 female | -1.07 (-3.85, 1.71) |  |
| 3.2 male | -0.20 (-2.96, 2.56) |  |
| **EM_Sex_ MCN or iron alone vs Any STH + PZQ + iron/MCN** | | |
| **Outcome or Subgroup** | **Effect Estimate** | **Test for subgroup differences** |
| 1 Weight | -0.11 (-0.38, 0.16) | Chi² = 0.03, df = 1 (P = 0.86), I² = 0% |
| 1.1 female | -0.08 (-0.53, 0.37) |  |
| 1.2 male | -0.13 (-0.46, 0.20) |  |
| 2 Height | -0.02 (-0.35, 0.30) | Chi² = 0.13, df = 1 (P = 0.72), I² = 0% |
| 2.1 female | -0.08 (-0.53, 0.37) |  |
| 2.2 male | 0.04 (-0.43, 0.51) |  |
| 3 Hemoglobin | -1.44 (-3.24, 0.37) | Chi² = 0.04, df = 1 (P = 0.85), I² = 0% |
| 3.1 female | -1.61 (-4.16, 0.94) |  |
| 3.2 male | -1.26 (-3.83, 1.31) |  |
| **EM_Sex_ MCN or iron alone vs Any STH with MCN or iron** | | |
| **Outcome or Subgroup** | **Effect Estimate** | **Test for subgroup differences** |
| 1 Weight | -0.00 (-0.16, 0.16) | Chi² = 0.46, df = 1 (P = 0.50), I² = 0% |
| 1.1 female | -0.06 (-0.30, 0.18) |  |
| 1.2 male | 0.05 (-0.17, 0.27) |  |
| 2 Height | -0.04 (-0.30, 0.21) | Chi² = 0.00, df = 1 (P = 0.97), I² = 0% |
| 2.1 female | -0.04 (-0.39, 0.31) |  |
| 2.2 male | -0.05 (-0.42, 0.32) |  |
| 3 Hemoglobin | -0.79 (-2.16, 0.58) | Chi² = 0.14, df = 1 (P = 0.71), I² = 0% |
| 3.1 female | -0.54 (-2.44, 1.36) |  |
| 3.2 male | -1.06 (-3.04, 0.92) |  |
| **Em_Age_ Any STH + PZQ vs Any STH** | | |
| **Outcomes and subgroup** | **Effect Estimates** | **Test for subgroup differences** |
| 1 Weight | 0.05 (-0.15, 0.25) | Chi² = 0.00, df = 1 (P = 0.98), I² = 0% |
| 1.1 age ≥ -2 | 0.05 (-0.15, 0.25) |  |
| 1.2 age < -2 | 0.10 (-3.96, 4.16) |  |
| 2 Height | -0.10 (-0.38, 0.17) | Chi² = 0.34, df = 1 (P = 0.56), I² = 0% |
| 2.1 age ≥ -2 | -0.10 (-0.37, 0.17) |  |
| 2.2 age < -2 | -2.22 (-9.35, 4.91) |  |
| 3 Hemoglobin | 1.62 (0.03, 3.21) | Chi² = 0.19, df = 1 (P = 0.67), I² = 0% |
| 3.1 age ≥ -2 | 1.61 (0.02, 3.20) |  |
| 3.2 age < -2 | 13.83 (-41.48, 69.14) |  |
| **EM_Age_ Any STH + PZQ + iron or MCN vs any STH** | | |
| **Outcome or Subgroup** | **Effect Estimate** | **Test for subgroup differences** |
| 1 Weight | -0.02 (-0.29, 0.25) | Not applicable |
| 1.2 age > -2 | -0.02 (-0.29, 0.25) |  |
| 2 Height | -0.08 (-0.43, 0.27) | Not applicable |
| 2.1 age >-2 | -0.08 (-0.43, 0.27) |  |
| 3 Hemoglobin | 2.50 (0.60, 4.40) | Not applicable |
| 3.1 age >-2 | 2.50 (0.60, 4.40) |  |
| **Em_Age_ Any STH + MCN or iron vs any STH** | | |
| **Outcome or Subgroup** | **Effect Estimate** | **Test for subgroup differences** |
| 1 Weight | -0.05 (-0.18, 0.09) | Chi² = 0.32, df = 1 (P = 0.57), I² = 0% |
| 1.1 age ≥ -2 | -0.08 (-0.26, 0.10) |  |
| 1.2 age < -2 | 0.00 (-0.22, 0.22) |  |
| 2 Height | -0.09 (-0.33, 0.15) | Chi² = 0.09, df = 1 (P = 0.77), I² = 0% |
| 2.1 age ≥ -2 | -0.08 (-0.33, 0.17) |  |
| 2.2 age < -2 | -0.20 (-0.94, 0.54) |  |
| 3 Hemoglobin | 1.72 (0.38, 3.05) | Chi² = 0.04, df = 1 (P = 0.84), I² = 0% |
| 3.1 age ≥ -2 | 1.77 (0.34, 3.20) |  |
| 3.2 age < -2 | 1.36 (-2.38, 5.10) |  |
| **EM_Age_ MCN or iron alone vs any STH** | | |
| **Outcome or Subgroup** | **Effect Estimate** | **Test for subgroup differences** |
| 1 Weight | -0.07 (-0.22, 0.07) | Chi² = 1.00, df = 1 (P = 0.32), I² = 0.0% |
| 1.1 age ≥ -2 | -0.02 (-0.20, 0.16) |  |
| 1.2 age < -2 | -0.17 (-0.41, 0.07) |  |
| 2 Height | -0.12 (-0.37, 0.12) | Chi² = 0.12, df = 1 (P = 0.73), I² = 0% |
| 2.1 age ≥ -2 | -0.11 (-0.36, 0.14) |  |
| 2.2 age < -2 | -0.25 (-1.01, 0.51) |  |
| 3 Hemoglobin | 1.05 (-0.27, 2.37) | Chi² = 0.04, df = 1 (P = 0.84), I² = 0% |
| 3.1 age ≥ -2 | 1.00 (-0.41, 2.41) |  |
| 3.2 age < -2 | 1.42 (-2.42, 5.26) |  |
| **EM_Age_ Any STH + PZQ + iron or MCN vs Any STH + PZQ** | | |
| **Outcome or Subgroup** | **Effect Estimate** | **Test for subgroup differences** |
| 1 Weight | -0.06 (-0.31, 0.19) | Not applicable |
| 1.2 age > -2 | -0.06 (-0.31, 0.19) |  |
| 2 Height | 0.02 (-0.29, 0.33) | Not applicable |
| 2.1 age >-2 | 0.02 (-0.29, 0.33) |  |
| 3 Hemoglobin | 0.90 (-0.92, 2.72) | Not applicable |
| 3.1 age >-2 | 0.90 (-0.92, 2.72) |  |
| **EM_Age_ Any STH with MCN or iron vs Any STH + PZQ** | | |
| **Outcome or Subgroup** | **Effect Estimate** | **Test for subgroup differences** |
| 1 Weight | -0.13 (-0.35, 0.09) | Chi² = 0.00, df = 1 (P = 0.99), I² = 0% |
| 1.1 age ≥ -2 | -0.13 (-0.35, 0.09) |  |
| 1.2 age < -2 | -0.10 (-4.18, 3.98) |  |
| 2 Height | 0.02 (-0.29, 0.34) | Chi² = 0.30, df = 1 (P = 0.58), I² = 0% |
| 2.1 age ≥ -2 | 0.02 (-0.29, 0.33) |  |
| 2.2 age < -2 | 2.02 (-5.13, 9.17) |  |
| 3 Hemoglobin | 0.15 (-1.64, 1.93) | Chi² = 0.20, df = 1 (P = 0.66), I² = 0% |
| 3.1 age ≥ -2 | 0.16 (-1.62, 1.94) |  |
| 3.2 age < -2 | -12.46 (-68.14, 43.22) |  |
| **EM_Age_ MCN or iron alone vs Any STH + PZQ** | | |
| **Outcome or Subgroup** | **Effect Estimate** | **Test for subgroup differences** |
| 1 Weight | -0.07 (-0.27, 0.13) | Chi² = 0.01, df = 1 (P = 0.92), I² = 0% |
| 1.1 age ≥ -2 | -0.07 (-0.27, 0.13) |  |
| 1.2 age < -2 | -0.27 (-4.29, 3.75) |  |
| 2 Height | 0.00 (-0.27, 0.28) | Chi² = 0.29, df = 1 (P = 0.59), I² = 0% |
| 2.1 age ≥ -2 | 0.00 (-0.27, 0.27) |  |
| 2.2 age < -2 | 1.97 (-5.24, 9.18) |  |
| 3 Hemoglobin | -0.62 (-2.13, 0.89) | Chi² = 0.17, df = 1 (P = 0.68), I² = 0% |
| 3.1 age ≥ -2 | -0.61 (-2.12, 0.90) |  |
| 3.2 age < -2 | -12.41 (-68.41, 43.59) |  |
| **EM_Age_ Any STH with MCN or iron vs Any STH+PZQ + iron/MCN** | | |
| **Outcome or Subgroup** | **Effect Estimate** | **Test for subgroup differences** |
| 1 Weight | -0.06 (-0.33, 0.21) | Not applicable |
| 1.2 age > -2 | -0.06 (-0.33, 0.21) |  |
| 2 Height | 0.00 (-0.37, 0.37) | Not applicable |
| 2.1 age >-2 | 0.00 (-0.37, 0.37) |  |
| 3 Hemoglobin | -0.74 (-2.76, 1.28) | Not applicable |
| 3.1 age >-2 | -0.74 (-2.76, 1.28) |  |
| **EM_Age_ MCN or iron alone vs Any STH + PZQ + iron/MCN** | | |
| **Outcome or Subgroup** | **Effect Estimate** | **Test for subgroup differences** |
| 1 Weight | -0.01 (-0.25, 0.23) | Not applicable |
| 1.2 age > -2 | -0.01 (-0.25, 0.23) |  |
| 2 Height | -0.03 (-0.34, 0.28) | Not applicable |
| 2.1 age >-2 | -0.03 (-0.34, 0.28) |  |
| 3 Hemoglobin | -1.51 (-3.33, 0.31) | Not applicable |
| 3.1 age >-2 | -1.51 (-3.33, 0.31) |  |
| **EM_Age_ MCN or iron alone vs Any STH with MCN or iron** | | |
| **Outcome or Subgroup** | **Effect Estimate** | **Test for subgroup differences** |
| 1 Weight | -0.01 (-0.17, 0.16) | Chi² = 1.49, df = 1 (P = 0.22), I² = 32.9% |
| 1.1 age ≥ -2 | 0.06 (-0.14, 0.26) |  |
| 1.2 age < -2 | -0.16 (-0.45, 0.13) |  |
| 2 Height | -0.03 (-0.29, 0.23) | Chi² = 0.00, df = 1 (P = 0.97), I² = 0% |
| 2.1 age ≥ -2 | -0.03 (-0.30, 0.24) |  |
| 2.2 age < -2 | -0.05 (-0.91, 0.81) |  |
| 3 Hemoglobin | -0.68 (-2.06, 0.71) | Chi² = 0.14, df = 1 (P = 0.71), I² = 0% |
| 3.1 age ≥ -2 | -0.77 (-2.24, 0.70) |  |
| 3.2 age < -2 | 0.06 (-4.10, 4.22) |  |
| **EM_Ascaris_ Any STH + PZQ vs Any STH** | | |
| **Outcome or Subgroup** | **Effect Estimate** | **Test for subgroup differences** |
| 1 Weight | 0.00 (-0.18, 0.19) | Chi² = 2.31, df = 2 (P = 0.32), I² = 13.3% |
| 1.1 ascaris0 | 0.09 (-0.13, 0.31) |  |
| 1.2 ascaris1 | -0.17 (-0.88, 0.54) |  |
| 1.3 ascaris2 | -0.25 (-0.66, 0.16) |  |
| 2 Height | -0.15 (-0.41, 0.12) | Chi² = 0.46, df = 2 (P = 0.80), I² = 0% |
| 2.1 ascaris0 | -0.17 (-0.50, 0.16) |  |
| 2.2 ascaris1 | -0.44 (-1.58, 0.70) |  |
| 2.3 ascaris2 | -0.04 (-0.53, 0.45) |  |
| 3 Hemoglobin | 1.49 (-0.09, 3.07) | Chi² = 0.06, df = 2 (P = 0.97), I² = 0% |
| 3.1 ascaris0 | 1.38 (-0.46, 3.22) |  |
| 3.2 ascaris1 | 1.99 (-3.46, 7.44) |  |
| 3.3 ascaris2 | 1.70 (-2.04, 5.44) |  |
| **EM_Ascaris_ Any STH + PZQ + iron or MCN vs any STH** | | |
| **Outcome or Subgroup** | **Effect Estimate** | **Test for subgroup differences** |
| 1 Weight | -0.08 (-0.32, 0.17) | Chi² = 4.64, df = 2 (P = 0.10), I² = 56.9% |
| 1.1 ascaris0 | 0.05 (-0.22, 0.32) |  |
| 1.2 ascaris1 | -0.76 (-1.68, 0.16) |  |
| 1.3 ascaris2 | -0.60 (-1.38, 0.18) |  |
| 2 Height | -0.17 (-0.52, 0.17) | Chi² = 1.75, df = 2 (P = 0.42), I² = 0% |
| 2.1 ascaris0 | -0.09 (-0.46, 0.28) |  |
| 2.2 ascaris1 | -1.07 (-2.60, 0.46) |  |
| 2.3 ascaris2 | -0.44 (-1.52, 0.64) |  |
| 3 Hemoglobin | 2.31 (0.47, 4.16) | Chi² = 1.79, df = 2 (P = 0.41), I² = 0% |
| 3.1 ascaris0 | 2.01 (0.05, 3.97) |  |
| 3.2 ascaris1 | 1.80 (-6.39, 9.99) |  |
| 3.3 ascaris2 | 7.34 (-0.26, 14.94) |  |
| **EM_Ascaris_ Any STH + MCN or iron vs any STH** | | |
| **Outcome or Subgroup** | **Effect Estimate** | **Test for subgroup differences** |
| 1 Weight | -0.05 (-0.19, 0.08) | Chi² = 2.50, df = 2 (P = 0.29), I² = 20.1% |
| 1.1 ascaris0 | 0.02 (-0.18, 0.22) |  |
| 1.2 ascaris1 | -0.26 (-0.55, 0.03) |  |
| 1.3 ascaris2 | -0.02 (-0.27, 0.23) |  |
| 2 Height | -0.14 (-0.37, 0.08) | Chi² = 0.27, df = 2 (P = 0.87), I² = 0% |
| 2.1 ascaris0 | -0.14 (-0.49, 0.21) |  |
| 2.2 ascaris1 | -0.27 (-0.82, 0.28) |  |
| 2.3 ascaris2 | -0.10 (-0.43, 0.23) |  |
| 3 Hemoglobin | 2.17 (0.88, 3.46) | Chi² = 0.42, df = 2 (P = 0.81), I² = 0% |
| 3.1 ascaris0 | 1.95 (0.03, 3.87) |  |
| 3.2 ascaris1 | 1.80 (-0.75, 4.35) |  |
| 3.3 ascaris2 | 2.82 (0.45, 5.19) |  |
| **EM_Ascaris_ MCN or iron alone vs any STH** | | |
| **Outcome or Subgroup** | **Effect Estimate** | **Test for subgroup differences** |
| 1 Weight | -0.05 (-0.19, 0.10) | Chi² = 0.03, df = 2 (P = 0.99), I² = 0% |
| 1.1 ascaris0 | -0.04 (-0.24, 0.16) |  |
| 1.2 acaris1 | -0.07 (-0.40, 0.26) |  |
| 1.3 ascaris2 | -0.04 (-0.33, 0.25) |  |
| 2 Height | -0.21 (-0.44, 0.02) | Chi² = 0.02, df = 2 (P = 0.99), I² = 0% |
| 2.1 ascaris0 | -0.19 (-0.50, 0.12) |  |
| 2.2 ascaris1 | -0.23 (-0.82, 0.36) |  |
| 2.3 acaris2 | -0.22 (-0.63, 0.19) |  |
| 3 Hemoglobin | 1.13 (-0.19, 2.44) | Chi² = 1.30, df = 2 (P = 0.52), I² = 0% |
| 3.1 ascaris0 | 0.70 (-1.04, 2.44) |  |
| 3.2 ascaris1 | 2.60 (-0.26, 5.46) |  |
| 3.3 ascaris2 | 0.81 (-2.01, 3.63) |  |
| **EM_Ascaris_ Any STH + PZQ + iron or MCN vs Any STH + PZQ** | | |
| **Outcome or Subgroup** | **Effect Estimate** | **Test for subgroup differences** |
| 1 Weight | -0.11 (-0.34, 0.13) | Chi² = 1.54, df = 2 (P = 0.46), I² = 0% |
| 1.1 ascaris0 | -0.05 (-0.30, 0.20) |  |
| 1.2 ascaris1 | -0.59 (-1.53, 0.35) |  |
| 1.3 ascaris2 | -0.34 (-1.12, 0.44) |  |
| 2 Height | 0.01 (-0.32, 0.33) | Chi² = 1.28, df = 2 (P = 0.53), I² = 0% |
| 2.1 ascaris0 | 0.08 (-0.27, 0.43) |  |
| 2.2 ascaris1 | -0.63 (-2.35, 1.09) |  |
| 2.3 ascaris2 | -0.40 (-1.44, 0.64) |  |
| 3 Hemoglobin | 0.85 (-0.99, 2.69) | Chi² = 1.48, df = 2 (P = 0.48), I² = 0% |
| 3.1 ascaris0 | 0.63 (-1.31, 2.57) |  |
| 3.2 ascaris1 | -0.20 (-8.43, 8.03) |  |
| 3.3 ascaris2 | 5.64 (-2.38, 13.66) |  |
| **EM_Ascaris0_ Any STH with MCN or iron vs Any STH + PZQ** | | |
| **Outcome or Subgroup** | **Effect Estimate** | **Test for subgroup differences** |
| 1 Weight | 0.01 (-0.20, 0.21) | Chi² = 1.74, df = 2 (P = 0.42), I² = 0% |
| 1.1 ascaris0 | -0.08 (-0.33, 0.17) |  |
| 1.2 ascaris1 | -0.08 (-0.82, 0.66) |  |
| 1.3 ascaris2 | 0.23 (-0.16, 0.62) |  |
| 2 Height | 0.01 (-0.31, 0.32) | Chi² = 0.14, df = 2 (P = 0.93), I² = 0% |
| 2.1 ascaris0 | 0.03 (-0.38, 0.44) |  |
| 2.2 ascaris1 | 0.17 (-1.05, 1.39) |  |
| 2.3 ascaris2 | -0.06 (-0.59, 0.47) |  |
| 3 Hemoglobin | 0.61 (-1.16, 2.39) | Chi² = 0.14, df = 2 (P = 0.93), I² = 0% |
| 3.1 ascaris0 | 0.57 (-1.55, 2.69) |  |
| 3.2 ascaris1 | -0.19 (-6.03, 5.65) |  |
| 3.3 ascaris2 | 1.12 (-2.80, 5.04) |  |
| **EM_Ascaris_ MCN or iron alone vs Any STH + PZQ** | | |
| **Outcome or Subgroup** | **Effect Estimate** | **Test for subgroup differences** |
| 1 Weight | -0.05 (-0.24, 0.14) | Chi² = 2.11, df = 2 (P = 0.35), I² = 5.3% |
| 1.1 ascaris0 | -0.13 (-0.35, 0.09) |  |
| 1.2 ascaris1 | 0.11 (-0.62, 0.84) |  |
| 1.3 ascaris2 | 0.21 (-0.22, 0.64) |  |
| 2 Height | -0.05 (-0.32, 0.23) | Chi² = 0.40, df = 2 (P = 0.82), I² = 0% |
| 2.1 ascaris0 | -0.02 (-0.35, 0.31) |  |
| 2.2 ascaris1 | 0.21 (-0.99, 1.41) |  |
| 2.3 ascaris2 | -0.17 (-0.72, 0.38) |  |
| 3 Hemoglobin | -0.62 (-2.12, 0.87) | Chi² = 0.20, df = 2 (P = 0.91), I² = 0% |
| 3.1 ascaris0 | -0.68 (-2.37, 1.01) |  |
| 3.2 ascaris1 | 0.61 (-5.17, 6.39) |  |
| 3.3 ascaris2 | -0.88 (-4.78, 3.02) |  |
| **EM_Ascaris_ Any STH with MCN or iron vs Any STH+PZQ + iron/MCN** | | |
| **Outcome or Subgroup** | **Effect Estimate** | **Test for subgroup differences** |
| 1 Weight | 0.08 (-0.18, 0.35) | Chi² = 2.90, df = 2 (P = 0.23), I² = 31.1% |
| 1.1 ascaris0 | -0.03 (-0.32, 0.26) |  |
| 1.2 ascaris1 | 0.51 (-0.43, 1.45) |  |
| 1.3 ascaris2 | 0.58 (-0.20, 1.36) |  |
| 2 Height | 0.08 (-0.34, 0.49) | Chi² = 1.31, df = 2 (P = 0.52), I² = 0% |
| 2.1 ascaris0 | -0.04 (-0.51, 0.43) |  |
| 2.2 ascaris1 | 0.80 (-0.75, 2.35) |  |
| 2.3 ascaris2 | 0.34 (-0.72, 1.40) |  |
| 3 Hemoglobin | -0.42 (-2.61, 1.77) | Chi² = 1.19, df = 2 (P = 0.55), I² = 0% |
| 3.1 ascaris0 | -0.06 (-2.43, 2.31) |  |
| 3.2 ascaris1 | 0.01 (-8.50, 8.52) |  |
| 3.3 ascaris2 | -4.52 (-12.22, 3.18) |  |
| **EM_Ascaris_ MCN or iron alone vs Any STH + PZQ + iron/MCN** | | |
| **Outcome or Subgroup** | **Effect Estimate** | **Test for subgroup differences** |
| 1 Weight | 0.03 (-0.20, 0.26) | Chi² = 4.60, df = 2 (P = 0.10), I² = 56.6% |
| 1.1 ascaris0 | -0.08 (-0.33, 0.17) |  |
| 1.2 ascaris1 | 0.70 (-0.18, 1.58) |  |
| 1.3 ascaris2 | 0.56 (-0.24, 1.36) |  |
| 2 Height | -0.01 (-0.35, 0.33) | Chi² = 1.57, df = 2 (P = 0.46), I² = 0% |
| 2.1 ascaris0 | -0.09 (-0.46, 0.28) |  |
| 2.2 ascaris1 | 0.83 (-0.68, 2.34) |  |
| 2.3 ascaris2 | 0.23 (-0.81, 1.27) |  |
[truncated: 55,486 more chars]
